# Correction: Generation of a Transcriptome in a Model Lepidopteran Pest, *Heliothis virescens*, Using Multiple Sequencing Strategies for Profiling Midgut Gene Expression

**DOI:** 10.1371/journal.pone.0133948

**Published:** 2015-07-24

**Authors:** The PLOS ONE Staff

## Notice of Republication

This article was republished on July 2, 2015 to correct errors in figures that were introduced during the typesetting process as well as errors in the Author Contributions. The publisher apologizes for the errors. Please download this article again to view the correct version. The originally published, uncorrected article and the republished, corrected article are provided here for reference.

## Supporting Information

S1 File Originally published, uncorrected article. (PDF)

S2 File Republished, corrected article. (PDF)
