# Supplementary material for: Generation of a Transcriptome in a Model Lepidopteran Pest, Heliothis virescens, Using Multiple Sequencing Strategies for Profiling Midgut Gene Expression
Source: PLoS One. 2015 Jun 5;10(6):e0128563. doi: 10.1371/journal.pone.0128563 (PMC4457788; doi:10.1371/journal.pone.0128563)
Supplement: S2 Table — (PDF) [file pone.0128563.s002.pdf]

Open reading frame sequences used for codon usage analysis

1. ***Table S2A Open reading frames used for codon usage analysis***
    a. ***Heliothis virescens*** Page 1
    b. ***Bombyx mori*** Page 27
    c. ***Helicoverpa armigera*** Page 56

2. **Table S2B. Codon usage data for *H. virescens*, *H. armigera*, and *B.mori*.** Page 82

Fifty open reading frames selected from *Heliothis virescens* for codon usage analysis

>Hv_Contig_104|annexin IX [Helicoverpa armigera]
atgaacactattattactaatctcgctgaactgagtcaagatcaatatgtcacaagggatcacagtgctaacggatgtttctattaccccgtatttgat
acacctgtatccgaaatcgtactgccaggatcttgcgaagccattacaggagtttcaaatttcaacgctcaagcttatcttggaacatggtacgagatc
gccagataccctcaaccaacgcaacagggccaatgtaacagtgctacatacgcaaacgctgctaacggagctgtctccgtcctgaacagccaagttctc
aatgaggctttggcttcaatatctggttcagctgtagtttcaagcacagacaacacaggaaaattaacagttacttttgtcgttgctggtgaggtcaga
aaccaggacttatacgtactcgccacggactatctcaactatgctcttgtatacgcctgtactaatcttcccaatggagacagagttgttggaagttgg
aaactgagtagaacgaaatctttaagcgcgaatgccttaacagtcatggatagtgtcatcgctaacacacaagcacttcacgaacaatattaccagcag
acactccaaactgatgcagcttgcttctattatcccgtgttcgatggtacggaaaccactattgatttgcctggaagttgcgcgacagcagcagttgcc
ggtgttcccaactttaacttagcaagctacgctggattatggtacgaagttgccagatacccgcaaccgactcagcaagggcagtgtaaccgtgctaat
tacgaatcaactgccaacggcgtatctgtccagaacagccaagttattaatgaggcattggctagtataactggcgaagctagggtaataagcacagac
aattccggagtattagaagtgaccctcaacgttggtggtgtgcccacaagtaccaccttatacgtactcgcaactgactataacagctactctgtggta
tacacatgtactgatctacctaatggaggacaggagagttggcagttggaaactcaggtagaagaagggaagcctgaatgctaa

>Hv_Contig_369|NADPH cytochrome P450 reductase [Helicoverpa armigera]
atgtcagacagcgcacaggacgttctaaaggatgcggcgaccggcgctgcagctgctgctgccgctgggggatcgcttttcagcacatttgatattatt
gtgctagtcatcttattgggcggcaccatttggtggctgtacaactcgaaaaaggaaaataaaaaagatgaaattcttcttagtaaatattcaattcag
gctgcgggttccatccaagttacagaaaattcttttataaacaaattaaagtcgtctggaagaagtttagttgtattttatggatcccaaactggtact
ggtgaggagtttgctggtcgtcttgccaaggaaggcatacgatacaagatgaaaggatggttgctgatcctgaagaatgtgatatggaagaactcatg
aaactccaagacattccgaattctttagctgtgttctgtatggcgacatatggcgagggagatcccacagataactccatggagttttatgaatggata
aagaacggcgaacccgatttgactggtttgaattatgctgtgtttggccttggcaacaaaacatatgaacattacaatgctgttgcaatttatctagac
aagcgtcttgaagaacttggcgctactcgagtgtatgaacttggtcttggggatgatgatgcaaatattgaagatgacttcattacatggaaagacaaa
ttttggccagcagtttgtgagaaattcaatatcgaaagcactggagaagaagaactgatacgtcaattcagacttgtgacacatgccccaggggatatc
cagccaaacaatgtgttcactggtgaaattgctaggctacactctttacaagttcaaagaccgccttacgatgctaagaatcctttcttagcccaaatc
acagtaaacagagaactacacaaaggtggtgataggtcctgcattcatgtggaacttgatatttcagactcgaaaatgagatatgaagcaggtgaccat

gtggctgtatacccaataaatgatagcaaccttgttgaacgattgggtcagttaacaggcgcaaatcttgatgagattttctcgctcatcaatactgat
caagaaagtagcaagaaacatcctttcccctgtccaacatcctaccgcactgctctgtcccactatgtggaaatcactgcattaccccgtactcacata
ttgcgtgagctcgttgaatattgctctgatgaagaagataaaaagaagttgatgttgatggcaactaactcgcaagagggcaaggctctatatcagtca
ttcattgtggatgcttgtagaaatattgtacacattttagaagatataccatcttgtaaacctcatctggatcatctttgtgaactgttaccacgtctg
caaccaagatattattcgatttcatccagtcccaagatgtacccagagactgtgcatattactgctgtgattgttaagtataaacaccaactggtcgc
gttaacaaaggcgtaactaccacatggctggcagaaaacaaaccagaacctggaaagccacttcctcgtgtacccgtgtacatcagaaaatcgcagttc
aggttaccattgcagacgcaaactccgataataatggtcggcccaggtactggtctagcaccgttccgtggtttcttacaagagcgtgcattcgctcgt
gcaaatgggaaggaagtcggggaaagtattctttactttggatgcaggcatcgtgatcaggacttcatttatcaggatgaacttgaaaagtatgagcaa
aatggtgatgttaaattaaaccttgcattctctcgtgaccaaaaagagaaagtttacgttactcatttattagaaaaggatatggaccttttatgggac
gtgattggtaatcgcaatggtcacttctacatctgcggtgatgccaagaatatggccgttgacgtaaggaacatagttctcaaagctatccaagacaag
ggcgggcgcacagaatccgaggcagtgcaatttatcaagaagttggaatctatgaagaaatattcagcagatgtatggagttaa

>Hv_Contig_753|3-hydroxy-3-methylglutaryl coenzyme A reductase [Helicoverpa armigera]
atgaaagtctggggagctcacggcgagttctgcgctcgccaccaatgggaggtgatcgtagccacgctagccctactcgcctgcgcagccagcgtcgac
aggaacggccctggcaacaggtccgaacactgcgcaggatgggctagagcctgtcctggcttagaagccgagtaccaagccgcagatgctgtcatcatg
accttcgtccgctgcgcagctctcctatacgcctactaccaaatctcaaaccttcacaaaatagcttccaaatatcttctcatcattgccggggtgttt
tccacgtttgcaagcttcatattcacatcggctgttgccagtttgttttggagcgaactggcgagtattaaggatgccccccttcctgtttctgttggtc
gctgatgtggctagggggcgagaatggcgaaggcgggatggtcggccggagaagaccagggaaagagggttgggagggcgctggctttgctggggcca
acagcaacccttgatactctgctagcagtcttattagtcggagttggagccttatctggggttcccaggctagaacacatgtgcaccttcgcctgtcta
gctctgttagtagactacctggtcttcgtgacgttctacccagcctgcctctccttagtcgcggatttcgcttcgggacgcaaagaaatgtctccagac
agtccgttttctgaagccgatctcaaaccgaacccagttgttcagagagtgaagatgataatggcggcagggctgctgtgtgtacatctcaccagcagg
tggccttggagcaacgatagcggcatcatcgaaggtccaatagacacattaactcctaccccaaatgataatattttgttacattcgtacgtcaaatgg
ttttccgtttctgcggattacatagtcatagcgactctgctctgtgccttgatcataaagtttgtattctttgaggagcaacgaaactgggttattgac
atgaacgatatgacggtcaaagaagtagtacaagagaaagctcggagtaagcctaagttctcagtaggagatgactcgaactcagaagtatccacgcaa
acggagggagtattagaagatgagtggcctacgctttccccgagctcgtccgctgctaaattaaactccaaaaagcgtccgatggccgaatgccttgaa
atatacagatctgaaggagcctgcaactcgttaagtgacgaagaagtcgtcatgttagtcgaacagtcccatattccactacacagactcgaagcggtc
ctaggagatccattacgcggcgtaagactgcgcagaaaagttgtcggatccaggttccagactgaattagctataaaacaactgccttacttaaattat
gattattctaaagttttgaacgcgtgttgcgagaatgtgatcggatatgtcggtgtaccagtcgggtatgctgggcctttagtcgttgatgggaagccg
tatatgattccgatggctactacggaggggctctggtcgcttcgacaaacagaggagctaaagccataggttcccgtggagttacgagcgtagttgaa
gatgtgggcatgacgagagcaccagctataaagctgcctaacgttgtgagagcccacgagtgcaggcagtggatagacaataaagataactacgcgctt
atcaaagaagccttcgactccacatcgcgattcgctcgactccaagaaattcacataggagttgacggtgcaacactatacttaagattcagggctacc
acgggcgacgcgatgggtatgaacatggtgtcaaaaggcgcagaaaatgccctcaaactcctcaaaaactacttccctgacatggaagttatcagcttg
tctggtaactactgttcagacaaaaaagccgcagccattaactgggttaaggggagagggaaacgagtcgtttgtgagacgacgatatcccatgatagt
ttaaggacgatcttcaaaactgatgccaaaactctggctcggtgcaacaagataaagaatctctcgggatctgctctcgccggttccataggcgggaat
aacgcccacgcggcaaatatggtgacggctatttacatagctacgggccaagatccggctcagaacgtcaccagctcgaattgttcaacaaacatggag
gtttgtggcgaaaatggggaggatttgtatgtgacctgtaccatgccttctttagaagttggtacggtaggcggagggactatcctgacaggccagggt
gcctgtttggacatcttaggagtgaagggggcgggggcacgtcccgcagagaattctgcgcgactagcttccttgatttgcgctacggttttagcggga

gagctcagtttgatggcggctctcgtgaattcagatcttgttaaatcacatatgaggcataacaggtctacggtgaacgtgcaagcgagttgcaactta
caaactgacaacatcactctgaaagtgccgactttataa

>Hv_Contig_786|midgut aminopeptidase N2 [Helicoverpa armigera]
atgcaattcatcagcttcatactcctggcgctggtcgccatcatttcggccgacttcccgcttccaccagaatttgatgaaccggagttccgtaacact
gctgacccagaagtctaccgacttcccgaagatctggaccccatacattacgttgtggaaatcaccccttacttcactgctacaactaccaatgaagcg
tttacttttgatggtattgttaccatcactgtcagggctctcaaagctgatctgaatgcactcttcattcaagaaaatgtaagagaaattgtgtctgta
gctcttactacagaagccggtgccaatgttccacttcatgctactactcccttcgaacgcataagagattaccatttcttgaaagtcaatttacctgtt
ggtgctaccttggacaacggagcagtttacaaactgactgtcacttatatcgggaacattaatgaaactccactatccagaggtgtgttcagaggcaac
tacatagataacgagggaaatatacgctggtacgcagctacccatttgcagccaaccaattcaagacaagcattccccagcttcgatgagcctggtttc
aagtctaccttcgacatcatcatcaatagaccagttaactttgctgagacctactccaacatgaggatttctacaagtactttagtcggcggtcgcatt
agggaagtattcgagactacccccaggatgtcggcctacttggtcaccttccacattagcgaagacttcacggtgattgccgataatcagaaccaagcg
agatcttacagaatcttagccagacccaacgctgccggccagggagcatatgctttagaagtcggaccaccgttgactacttggcttagtgaatacttt
ggcattgattactatagcatgcagccaaatattaagaacgatcaaatcgcttctccttattgggcttcaggagctactgaaaactggggattagtgaca
tacagggaattgcgtctgctttacgaagaaggagagaccaatgctttggacaagatgtacattggtactattactgctcacgagctcgctcacaagtgg
ttcggcaacctcatcacctgcagatggtgggataacgtctggatcaatgaaggcttcgccagctacttcgaatatttcgccatggatggagtagacaaa
tcaatggaattagaagaccagttcaacataatgtacgttcagagcgctctctctgctgacgcgtcccttgcgactagggcacttcagcacacagtgaac
agcccagccgaagtcacgggacacttcagtggcatcagttactcgaagggagcttccttactcctcatgttgaagcatttcctcaccgaaaatactttc
aagaaggctttgaactacttcttggtcgagaggtcatatgaacacgctttcccatcacacctctacagtgcgtttgccaaagcagttcaggaagatggt
gtaccatccaccaccttcgatattgcatcattcatgcaatactgggtagatgaaccaggatatccagttcttgaagttaatgtcaattcggcaaatggt
cgcattgagctgtcccagaaacgtttcttagtcagtgctactgccacaaccaccgaccaagtgtggcccctgcctcttacttacacgaccgaaagcaat
cctgactggaacaaccttttgcctagtaaagtgatgaccacgaagactgacttcattgaaaggaatgtaggagccaatgaatgggtcattttcaacgtt
cagcagaaaggtatctaccgcgtgaactatgacactaacaactggcagttgcttgcaaacgctctgagcagggaccacaccgctatccatcatttgaac
agggctcagatcgttgacgacgtttttgctctgatgagatctggacaaatgacgtatcgtcttggattccgagtgctggacttcttgaagaaggacacg
agctactactcgtggtatccagctatcactggttttacctggctgaggaaccgccttctccacattcctgcgtccttggctgcatttgacgaaattctc
tacggcttcctagacgctgtgataactgaccttggatatgacgtggttgcaaatgaacctcttacaagaactttgaacagattcttcaccctgacattt
gcatgcaacatcggacacagaggctgtatcgacaacgctgtgcaaaaattcgtggctttaagagataacgcagtcgctgtgaaccccaacttgaggcgc
cacgtgttctgtgagggtcttcgagctggtggcctgaacgattggcagttcctatacaatcgccgtcaagcttctaacaaccagggagacgaagtcgct
atgctcagatctttgggatgcaccaccaacgagcaagctaggcagttgtacttgcaaatgattctgtctgacgatgacgtgaaggctcaggatcgagtg
aacgcattcacgttcttctacatgggagaccgcagcaacgctaacgttggtctgcaatatatcaagggcaatgttgatgccattagaaaggagtcgtc
cttccagcatggttcaacagcgttctcagcaacaccgccgcctatttagatgaagctggtttgaaagatatggaagaatggctattggccaaccaaaac
gctgttcctgaattcgcagtgggcatcagcgctattaactcagccagagctaacatgcagtggggttcagacaatgctgctactataatagctgcagcc
aatgatgaagatcctccagcgccagaagaagatggttcaggagacgaaggttcaggagacgatggttcaggagacgatggttcgggtgacaatggatct
ggagaagattcagatgacacaatgaccaccgcacctgtaacaacaacaaccccaagtgaaccagtagatgaagtgacgacgacagaagcaagtgccaca
acaacaactggcgcagatgaagaatcaactacaacaactgtaagttcaacgacttcaagtacaacagctgcaccaggatctgatgaaagcgaagaagat
gaacctagttcagcagcaatattcctgccaaccactgtactgttatcatggacagtactcgcaatgttgctataa

>Hv_Contig_879|relish [Helicoverpa armigera]
atgtctacgagcagtgaccacgatatgagcgatgcctccagctctcctatgtcttttaacatatactctcctcatagttctccgtcgcagcaggttcct
cagctggcgactgacctcactgagctgacttgcgctgacaataagcgacaatttcttagacacgccttgaaaccatgcctgcgcatcacggaacagccc
cagaatcacttcagatttcggtatgtcagcgaaatggttggcacacacggatgtcttctgggaaaatcttatggctccaacaagacaaagactcatcct
accgttgagctactaaattacagtggaaaggctctgataaggtgtcgtttggctcaacacaataaccctgacgaacacccacacaagttgcttgaagat
gaacaggacagagacatgagttacctagtgcctgatcagggaagctataaagtcgggttcggcgggattggcattatacacaccgctaaaaaagacgta
gctgcacttctattcaagaagttttccgaaagatgtaagaacaccaatgtgaacttgaaagaccttcaagttcaatgcgagaatatggccaaaactata
aatttgaatattgttcgcttgaaattcagtgctcatgacactgttactggtcaagagatatgtgaacctgtgttctcggaacctattcataacatgaaa
agtgccgctactaatgatttgaagatatgccgtataagccggtcatccggcagtgcttctggtggagaagatgttttcatactggtcgagaaagttaac
aaaaagaacataatgattcgcttcttcgagatggacgagaatggtgaaagaggttggaccagtgtcggccagtttatgcagagcgatgtccatcaccaa
tacgccattgtgttcaggactcctccatacaaaaatcccgagacgccagtcgatgttcacgtgtttatagaactagtgcgtccctctgacggtcgcact
agtgaacctaaagagttcaagtacaaagcgaatcaagcgtacaaacagatcaagaaaaggaaaaccggatcgtctttctgctctatcggcagttcatct
agtggatctctgaaaagtggctgcgacattcccatatcagttgtcaatcatcaagctgaagaaattatggagcgcgaccccgacccgacttcgatgtat
atcttgcctcaggtgcacaacccgacatcgccgaaccaatgcgacctcggcagcgcgttatactcggcccccgggtcggagtcgagcctgtcgccgatg
tccagccccatgtggagcgagccccacagcgtgatgcttcccgaaccacccatcgcagacttacaactcaattccgcagactttgaacagatcaccgcc
aacaacgatcccgcgaacaatctgataccagaagaagttaaaagcttttgaatgaatatttgaaaagtcacggagaaactcttcctgaagacaaaaat
agtatggagtttattcgctctttgctagtcgccgactcgggacgaccaaagttacagaaagaggaaaagttgcgttaccagggaccaaaaacaggcgag
gtctatcccaaaactgaggacgaactcaaaaagcccaagaacgagtaccccgcattctacaaaacggaggatggcatcgaagttaagaagattgtgaaa
gacttgtgtgaaatgatcagaaacaaaaagggattcaagaaggctgaagtcagaagccgcttggagaggttatttgaaataagactgtcaaatggagat
acattcctccacatggcgttgtgcagtaaccagccaagtcttgagtacatcgtgaaaataatacatagtgtgaaagcgacgcacctcctagactgtgcc
aatgataaacaacagacgccactacacctcgccgtcgtcaatgatatgccgaaattggtgtcgttgtttgttgcaaaaggatcgaatccaatgctgaag
gatgatgacgacctaaatgtgatccactacgccgtaaagtacaagtcgtgcctggaagtccttctggactccattaagaagaacgatgtgccctgtgac
ttgaacgactacaatggtgagaagcagtcggcgctgcacatggcggtggtgtcgggctgggcaggcggcgcgcggctgctgctgcagcacggcgccagc
tacagcgtgcgcgacgccgccggccgcacgccgctgcacctcgccgcctacgacgaccgcctgcccgtcctgaggacgctgctcgacttcataccaccg
agcgaaattgacgtcatggatgacgctgggaatacggcactacaaatcgtctgtggcggcacgaccgtcagggaaaattctgttgaaatagcaagactg
ctgcttgaaaagaaggcgtaccctctaaaacacgaggattgcaacgaatccgcgtggagactcgtcaggaagaagccggaactcaaagagttgatgaaa
gcttacgtcacgtcgcccgactgcatagacgaagacgacatcaagtccgaaccggaggacgagttcgagtcggctgacgaaggcgagtacatggaggcg
gggctgcaggagctggggtcgtacgcgcgcgaggtgtcggcgctggtggacggcgcggcggcgtggcgcgcgctggcgcagcgcctgcgcctggacgcg
ctgctggactggtacgccgcgcagcccagccccacgctcacgctgctcaaacatctcaaggattctcgagacgacatatcttccaagtcgttggccttg
attttggaagacatgggtcaaattgaggctgcacgagtcataaggaggtatatcgactga

>Hv_Contig_920|aminopeptidase N5 [Helicoverpa armigera]
atgtatttactggcactactcactctcttcgggtcggcattcgccggtcccatctcaacc
caagtggagagcagaaacctagcctccacctacgtcctgcctaccgacaccatccccaccttctacgatgtcagcctgttcctcaaccctgacaacgag
gagagcttcagaggatccgtctccatccgtattctgccaaaagtgaccaccagtgaaatcgtcatccatgctatggctatgcagatagattcgaacatt
cgagtttctagcgacagaaatccagctaataatctatacgtttcccacacattggccactgatgacacccattttctaaggattcagttgagtgaaccg
atgacaatactccagccgtacactgtagaaatcagctacgttggccagttcgctgaaaatatgttcggtatttacttatcgaaatatcaaaacaacggt

caggaggagagacttatcacatcccaactgcagcctacgtttgctcgtcgcgcrttcccctgctatgatgaaccgggtcttaaagccgtcttcagaacc
accatatacgccccaccatcgtaccccatcgtcaggagtaacatgcctttgaggagcgacttgctgaaagaaaacgtagctggttacacgaaacacgag
ttccaggatactctcattatgtccacctacctcatcgcctacctggtctccaacttcgttcacgtcgaaaacagtgccaaccccatctaccgcatccca
ttcagggtctactccagacctggtattcaggacaccgctgaattcgccatgatcttcggacaggaaaatatggcagcattggagaaatacaccgagttc
gattacgagttcccgaagatggataaggtggctgttcctgactttgctgctggtgctatggagaattggggtctggttatctacagagaagtggcactc
ctagtaacggacggagtgacaacaacagcgaccaaacagaatgtaggcagaattatttgccacgagaacgtgcatcagtggttcggcaacgaagtcagc
cctgtgtcctggacttatacttggctcaacgaaggttttgcgaacttcttcgagaactttgccactgacctggtgagaccagactggcgtatgatggac
cagtttgtgctggccctgcagaacgtctttcagtctgatgctgtcgccagcgtgaaccctatgactcatgcggtgtacacgccttccgagatcctcggc
actttcaacgctgttgcttatcagaagtctggctccgtgattcgtatgatgcaacacttcctcacacctgaagtattcagacaaggctcgtcatttac
atacgcagcaacacccgtgaggcggccacacctttgaacctatacgcagctctccagcaagctctggaccagtcctcccacagtatcaacttcccagtg
aacaccatcatgcagcgatgggccaaccagggtggtttcccagtcctgacagtcaccaggagcgctgcctctgcccagtctattgtcgttgaacaggaa
cgtttcctcatggacagaagccagagacttcccgaccgttggcacgtacccgtcaactgggtgctatctaccaaccctgacttcagcgacactagtcct
cagggttgggtccctcctaccttccccgcgcagtcctttaatattccgggattgtccaacgctgagtggtacatcattaacaagcagcagacaggttac
tacagagtgaattacgagccttccaactgggcggccttagccagggtcctcaacagctcccacgagagcatccatgtgctgaatcgtgcgcagatctta
gacgactccttcaacctggccaggaatggacgtttgaactacgattaccccttcactatctcaagctacctggtgaaggagagtgactacataccctgg
ggtgctgccaaccctgccttcacttacctggataccgtgctcagttcatctcctgtatacaatctcttccagagatatcttctacacttatcagctcct
ctttatgagcaacttggcttccaagcagttgaaggcgaaccattcgtgactccttaccacaggaatattatcctggatctgaactgccgtcacggcaat
cccgagtgccttaacactgcccaatcgttgcttgaaggattcagaaatgacgacagtcaacccttgaatgccgacatccaaaccctggtcttctgctcc
ggtcttcgcggtggtagtgcagagaacttcaacttcctctgggacagatatctgggaacctcagactctagtgaacagtctatcctgctgaacgctctt
ggatgcacttctaatgaagaaaggcgtgctttctacatgaaccaggtgatcagcgatgactccccagtgagggagcaagataggcacaccatcttggtg
tcagtcatcaacgccagtcccaacagcactcaggcagctttcgaattcattattgagaactttgcggcgatccaaccaagagtacaaggcctgactgga
acaacgaacatcttgaatgctctcgccagaagacttacaagccaggctgattttgatcaggtcgagtcattcgaagctcgataccagaatatcttcaca
gccggtgaactggcttccattgccggtattaaggagaacatcgcatcttccattacttggagcacgcaaaatgcaccaatcgttgaagcgtggctcaga
gcgaactacgatgacagcggtgcacctgctctcgtttctggcttcctagtacttatttctatctttgtgacaatatacaatcattaa

>Hv_Contig_1178|heat shock protein [Helicoverpa armigera]
atgctcaaaatgcggtggagtattctagcgttggcctgcgtggtcgtggcggtgtgcgccgacgacaagaaggataaggagaaggacattggcaccgtc
attggtatcgacttgggtaccacctactcctgtgtgggtgtgtacaagaacggccgtgtggagatcattgctaacgaccagggtaaccgtatcaccccc
tcatatgtagcgttcacagccgacggcgagcgtctgattggtgacgccgccaagaatcagctcaccactaacccagaaaacaccgtcttcgacgccaag
cgtctcattggtcgtgaatggggagattctacagtccaacatgatgtcaaattcttccccttcaaggttgttgagaagaacagcaaaccccatgtttcc
gtcatgacctcccaaggtgacaagatcttcgcccctgaagaaatctctgctatggtcctcatcaagatgaaggaaaccgctgaggcttacctcggtaag
aaggtgactcacgccgtcgtcacagtcccggcttacttcaacgacgctcagcgtcaagccaccaaggacgccggtgccattgctggtctccaggtcatg
agaatcatcaacgagcctactgcagccgctatcgcttacggtcttgacaagaaggaaggcgagaagaacgtgttggtattcgacttgggtggtggtacc
tttgacgtgtctctgctcaccatcgacaatggagtctttgaagtagtagctaccaatggtgacactcacttgggtggtgaggacttcgatcagagagtc
atggaacacttcatcaagctgtacaagaagaagaagggcaaggacatcaggaaagacaaccgtgccgtacagaaactgcgtcgtgaagtcgagaaggct
aagaggctctgtcttccagccaccaggtcaagatcgaaattgaatcgttcttcgaaggtgaagatttctccgaaactcttaccagggctaagttcgag
gaattgaacatggacttgttcagatctaccctcaagcctgtgcagaaggtattggaagacgccgacatgaacaagaaggacgtcgacgagatcgtactt

gtcggaggttccacccgtatccccaaggttcagcagctcgtcaaggaattcttcaacggcaaggagccttcccgaggtatcaaccccgacgaagccgtc
gcttacggtgccgccgtccaggctggagtgctcagcggtgaacaagacactgacgctattgtgctgttggacgtcaaccctctgaccatgggtatcgag
accgtcggaggtgtcatgaccaagcttatcccccgtaacaccgtcatccccaccaagaagtcccagatcttctccaccgccagcgacaaccagcacacc
gtcaccatccaggtgtacgagggtgagcgcccaatgaccaaggacaaccacttgctcggaaagttcgatctgactggcattcctcctgcaccccgtggt
attcctcagattgaagtaaccttcgaaatcgacgctaacggtatcttacaagtgtctgccgaggacaagggaactggaaaccgcgaaaagatcgtgatc
accaacgaccagaacagactgacacctgaagacattgagaggatgatcaaggatgccgagaggttcgcagatgaggacaagaggctcaaggagcgcgtt
gaggccaggaacgagctggaaagttatgcttactccatcaagaaccagctccaggataaggagaaacttggtgctaagttgagtgatgacgagaaatcg
aagatggaggaggcgatcgatgccgccatcaagtggctcgaggacaaccaagacgtcgactcagaagaatacaagaaacagaagaagtcccttgaggac
gtagtccagcccatcatcgccaagttgtaccagggacaaggtggcgtgcccccgcccggtgcgggaggtgacgaggaagacttcaaggacgagttgtaa

>Hv_Contig_1196|inhibitor of apoptosis protein [Helicoverpa armigera]
atgtggtcgtgttgctcaccttgtgtaaatactcaaaaaactggattacaaatggatataaccaaagtggcatccaatggccccgcctcaccattaacg
ctattcaagagcgctccgcgcgaggctaagattcgacctctggttgggccgctagtactgccgacgccgagctacgactcaaacgccggctctccggcc
tcgtcaccctctacaccttcctgttcctcttcattcagcaccgacaagaccgacaaccatgatacctttggcttcagcacagacacaattgatatgcgc
cgagaagatgaaagaataaaaacattcgaaaaatggccagtcacctttctacccggagaacagctcgctcggaatggattttattaccttggtcgcgga
gatgaagtacgctgcgcgttctgcaaagttgaaataatgagatgggtcgaaggcgacgatcctgccaaggaccatcagcgttgggcgccacaatgcccg
ttcgttcgcaagctgggcggcggcgccagcacggactccagcagcgcgggccgggacgagtgcggcgcgcgagcggccgcctccggcaccactcccccg
cgcatggccggccccgcgcaccctcgctacgcttccgaagccgcgcgtttgcgcagttttaaagactggccgcgatgcatgcggcagaaaccagaggaa
ctcgccgaggccggtttcttctacaccggtcaaggggataagaccaagtgtttctattgcgatggcggtttgaaagactgggaaaatgatgatgtcccc
tgggagcagcacgcgcgctggtttgaccgctgcgcctacgtgcagctggtgaagggtcgcgagtacgtacagaaggtgatgtcggaggcgtgcgtggtg
ccggcggccgaggcggggaaggacgtggcgccggcgcggctgcccagccagcccagcacgcccaactcgcaacccgaaacgccggagaactccgtagac
gactccaagttgtgtaaaatctgttacgcggaggagcgcaacgtgtgcttcgtgccgtgcggacacgtggtggcgtgcgccaagtgcgcg
ttggcggccgacaagtgcccgatgtgccgcaggacgtttcagaacgcagtgcgattgtatttctcgtga

>Hv_Contig_1359|dorsal [Helicoverpa armigera]
atggcgcgccgcgaccagccctacgtggagatcgtcgagcagccggccagcaaggcgctcagatttcgctacgagtgtgaaggccggtcagcaggttcg
atccccggcgtgaacagcacgtcagacaacaagacttaccctactattaagatatgcggctacaccggacaggtcgtcatcgtcgtgtcctgtgtcacc
aaagatgagccttacagggcccacccccacaacttagtgggtcgcgaacgctgcgagcgaggagtgtgcacgatcccgctccgcgtcacggcagagaca
tgcgaataccagttcaagaacctcggcatccagtgcgtgaaacggcgcgacattgccgaggcactcaccgtcagggagaagcttagagtcgaccctttt
agaaaaaacttcgaccacaagaaccaccccaaagcatcgacctgaacgcagttcggctatgcttccaagtgttcctaccggacgagtcggggcggctg
cggcggcccctggcgcccgtggtctccgatgtcatctacgacaagaaggccatgagcgacctgctcatcatgaggtccagccactgttctggcaccgct
aggggtggcacgcaggtcattctgctgtgtgagaaggtgacccgagaggacatagaagtggtgttctaccaggaggagaacggcatcatggtgtgggag
gagatggccatccgcatcctcgtgcacaagcaggtggccatcgccttcgagacgccccccctacaagttccccaacaccacggaccatgtgcatgtgcac
ttccagctgaagcgcctgtcagacaacgctcgcagcaactctctgccgttcgagtacatccccgagttcacagatgtgagtaataagaggcggaaggtg
gttagcgacatgttacgaaactacgaaatgcatgaccgaatttattcaccggagcagatcaagtcggaacctagagaccggactccgccacaccataac
attagcagtccgccactccacggctacgcgccgccctacgaacagaattggaacatggacaatatgcaaggcggtatagcagtcccagggccgagccat
gtcagcccggtaccagccggtcatggcaacctgttcgtccaggagagcatgtgggccaaccagcagtacggccaggtgtcccctggccatatgcagcct

ggcatgccccagaacctgcaggtgttacagcccaatatgcaggccatgtctcccagtatgcagcccatgtcacccaacatgcagcccctgtcacccaac
atggcgtacgccgagcgcatgtcgcccaacgtgggcgtcatgtcgccgcaccagggcatgtcgcccgccatgcagggcatgtcgcccataggccgcgtg
tcacccaacgtacagccgaacatggggcacatatcgcctaacctgcagcagcagccggcctatgtgcagcaggccccacagctagcgcagccggcgggc
acgctgatggagacggagacacacagcacgacgtcgctgagccacctgctgctggaccgcgcggagcactcgctgctgctgaactcgggcgagctggcg
ggcctgtccgcgctgctcggcgaccgcagcgccgagtcgcagcccgcgctagactactaa

>Hv_Contig_1426|cytochrome p450 6B2 [Helicoverpa armigera]
atgtgggtgttttatctgccagtagtgatatcagtgttagctgtagcactttattattatttcacacggacatttaattactggaagaaacgaaatgtt
cgcggaccggaacctactgtacttttcgggaacttgacggattcagttctccgaagaaaaaacataggagtagtgatggaagaaatgtacaacatgttt
ccagatgaaaaagttattggaatttataggatgacctcaccttgcctactcgtacgagatttggatgtgattaagcacatcatgatcaaagacttcgaa
gtgttcagcgatcgtggtgtggaattcagcaaagagggattgggacaaaacttattccacgccgacggagaaacgtggagagccttaagaaataggttt
acacctattttcacatctggtaaattgaaaaacatgttctatctcatgcacgaaggtgctgataacttcattgaccatgtgacagcagagtgtgaaaaa
aaacaagagtttgagattcactcccttctccagacttataccatgtctacgatctcctcgtgtgcatttggagttacttatgacagtataagcgataag
gtaaatactttagcaattgttgataagattatttctgaaccaagttatgctatagaattggattacatgtaccccaaattactggctaaactcaatctt
tcaatttttcctactgttgtacataaattcttcaaaaatcttgtaaataacattgtcactcaaagaaatggcaaaccttcaggtcgtaatgactttatg
gatctcatactagaactccgtgaaatgggagaaatcactagcaacaaatatggtaacaaccttacatctcttgaaataacagacacagttatgtgtgcc
caagcttttgtatttacgtagctggatatgaaactagtgctactactatggcctacttgacatatcaattggcacttaatcaagatatacaaaayaag
ttgatagcagaaatagatgaagcaatacaagctagtggtggaaaagtaacatacgacaccgtgaaagaaatgaaatacatgaacaaagtttttgacgaa
actcttcgtatgtactcaatagtagaaccactgcaaagaaaagctataagagattacaaaattcctggaactgatatcgtcattgaaaaagacaccatg
gtattaatatcaccaagaggtattcactacgaccccaaatattacgacaatcccacccaattcaaccctgatagatttgacgcggaggaagtggcaag
cgtcacccgtgcgcgtacttaccgtttggacttggacaaaggaattgcattggtatgcggtttggcagacttcagtccctactgtgcatcacgaagctt
ttgtcaaagtttaggatagagccatcgacaaagaccgacagaaacttgcaagttgaaccacgccgagttatcataggaccgaaaggaggaatacatgta
aaaattgtgcctagaaagattgtatcctaa

>Hv_Contig_1509|flavin-dependent monooxygenase FMO2A [Helicoverpa armigera]
atgttttttttttttttttcaagcagaagacggcatacgagcatgtataaatatttaagaacaaacctgccaaaagctccaatggaattgagaggcttt
cccttacctgattacctgccatcgtaccctactggggaagatttctaccactatctagaagagtgtgtggagcgcttagacatttcgaaatatatcaag
ttcttacactccgtacttgaagtaagaagaataaatgactcgtggaaagtcaaatacgaaaacgttgtaacaaaggatacatttgaggaagactttgat
tacgtcattgttgggaatggacactttaacaaacctagcttacctaatatccccggtgaagatctttttagaggcacaataatgcatagccatgactac
aaagcaccagaaccgttcacgaatcgtcgcgtattagtcgtgggcgccggcccgtctggcatggacatcgggctcgaagtggctgacgtagccagtgcc
ctcatccacagccatcactcccggatcaactggaccacgcccttcccgcctcactacgttaggaaacctgacatcaaagagttcaatgaaactggagcc
atattccaggatggcacctttgaagaagtcgatgttgttctttattgtacagggttctattacgactttccgttcttggatgagtcgagtggcctgacc
atggagcctaggagcgtggtgcccctgtacagctacaccgtgaacatcaaccagcccaccatgttcatcatgggagccttcatcatagcctgcttggta
gtcgctttggatgctcaggcacgctacgcaacagcttatatcatagggaacttcagtttgccatctcgagacgagatgatgctggagtggcagaagaga
atggacaccatccgctccaagggactgcctacttcttacatacacattctaggagaaaaagaggacgaattctacgccgacctaacaagagaatcaggt
atcgagagagttcccccagtgatgttcaagatccgcaccctggacaccgaagccaagatcgagaacctgtacacctacaggaactacgagtacaccgtc
attgatgaccacaccttcgtcaggaagttagaagacccggccgtcagaaaaggcatgcatgtatatgggctagcgtatgttaattaa

>Hv_Contig_1534|carboxyl/choline esterase CCE014a [Helicoverpa armigera]
atggcaatgatatcggctgtcaacgacttttttagatgacctgcgagggggcaggatgacagaagcaccggttgtgaccgtggaacagggtcagctccaa
ggcaaagtggtgaacagccccacagggaaggcgtactacagcttccaaggaatcccttatgctaaaccccctctagggtccttgaggttcaaggcacca
caacccccagagccatgggacggcatccgcgacgcgacttcagaaggcagcgtaagtgcacaaatagacttcatggcaaccaaacagttcactggtgac
gaaaactgcctcttcctgaacatttccacccctaacctagacggagagttcctcccagtaatggtattcatccacggagggagtttctcgttcggatca
ggcaattccgacttctacggagcagattatttagtagaaaaagatgtagttgtagtcacgataaactacagatgcggtgtccttggattcttgagtttg
aataccccctgaagtgcctggtaacgccggtcttaaagacatggtacaagcgatcaggtggatcaagagtaacattcacaattttggaggtaacagtggg
aatgttaccattttcggagaaagcgccggaggtgtggcggtatctctgcttactgctagtcctttaacgaaaaacttgatcagtaaggctatcgtgcag
tctggtagtgcactttcgaactgggccatacagaggaatcctctagaatcggccagaaacatcgcgaagatcttaggatgcgagtctaatgatcctgaa
gagattttggacatccttaacgctacgtcaacgaaggacctggttgaagcccatcataaacttagtcctgaggaagaattcccggacacaataaacgct
ttcggttacgtcgttgaaaaggagttcccgggagtagaagccgttattacagaatcgtttttagacctattaacatctggacgagttgctgacatccca
atcatgattgggtcaacatccctcgagtttgctttagagaggaaaacagacgatttgcaagtttttatcccagatgagctaaatatcgaaaggaattca
gaggaatcgctggcgatcgctgaacaattaagactttatacttcaagggcagtcatactggggtggaaagtctacctgagtattttgaacttctgtca
gacgctatcatcaatgtagacacgcatagacatgtccaatacttgataaacgtatcaaagaaacctatttattactacaagttcgattatgtaggcgag
ttgaacatttcgaagaaaatttttgaacagctttggtctgaaaaaagctatgcatatggatgaattagggtatctgttcaagaatgaccttcagaaggat
gttgaacctactaccctggacgtgaagatgagggagaggatgctaaggctttggacgaactttgctaagagtggaaacccaacccccgacgagaaccac
tacttaaccgtcacctggttgccagtaaccaaggacaacctctactacctgaacttaggccaagagctatctctggcaaccaatcctgataaggagaag
atggaattctgggacgaactctacagcaaatactatcgcatctgggaacagcctaggacgaacgttgtagaaacagcagtcatatattctgaaccgaaa
atcgttgagtctatctcagaaagtgggaccgtatatacttactccgaaacgacagaatcccatcacaccttcatatctgaatcccacgaaattatcact
gagaacaaccaaataatttccgaaacccacgaagtcattactaacaatgacgacgacgtcccccaaatacaagaagtcgttacgcaaaataacgaagtc
gtaacggagaataacgaagtcgtaacggagaataacgaagtcgtcccggagaggaacgaagtcgataacgtagtggaaattaaacctgaacctgttaaa
attgaggatgtggagcccgtttctattattggggctgttacaaacggtgatcagaagccgaagccaaggctttcgaacgagattaagatggtcactcgc
tcgaatggggcacctaaagatgttataagggccaatgacccgccagaagatgatttacctaaaaatattggagtcaataagtttgttaacttctttgaa
tcgcttggggctaagaagtaa

>Hv_Contig_1598|molt-regulating transcription factor HaHR3 [Helicoverpa armigera]
atgatgaacagcaatcagttccacgatttatttgggtcgcagtggcctccagaccagcacggaggtcactcgtccgcgtcgacgatgctgcaccagtcc
cagggtctacctcagggtatgcagctgaagagagaacctcatacggatgtgcagccggtgatgcacaaccagatgggcatggacatcacgtccgggtcc
gtggcggatagtacatcccccccgcctggcagcagtgatgggatgttcggctcatcgatatctggcatgtttatggataagaaagccgctaattctata
agagctcaaatcgaaataataccatgcaaagtgtgtggggacaagtcatcaggggttcattatggagtcatcacctgcgaaggatgcaagggcttcttc
agacgatcacaaagcacagtggtgaactaccaatgtccacggaataaagcctgcgtggtggaccgcgtcaacaggaatcgctgccagtactgcagattg
cagaaatgcctcaaacttggaatgagccgtgatgcggtgaagtttggacggatgtcgaaaaagcaacgtgagaaggtggaagatgaggtgagattccat
cgtgcacagatgagagcgcagactgacacagcgcccgattcagtatacgacgcccaacaacagacgccgagctcaagcgaccagttccatggtcactac
aatggctacccgggctacggatccccattgtcctcatacgggtacaacaacgcgggcccagcattacagtccaacatgggcggcatacagccccaaccg
ccccaacagcagccctacgacgtgtctgctgactacgtggactccaccacagcttacgagccgaaacagaccgggggattcttggatcctgattttatt

agtcatgcggagggtgacatcagcaaagttctggtgaagagtttggcagaagcacatgcgaataccaaccctaaactggaattcattcacgagatgttc
agaaagccacaagacgtctccaagcttctatactatagttctatgacgtacgaggagatgtggctggactgtgctgacaaattgactgggatgattcag
aatataattgagtttgctaaacttatacctggcttcatgaagttgacccaggatgatcagattttgctcttgaaatctggctcgttcgagctcgccata
gtccggctgtcgcgactgatagacgtcaatcgtgaccaagtgttgtatggagacgcggttctatccatcagagaatgtgtgcatgcacgtgatccccgc
gacgtggcgctagtggttggtatcttcgacgcggcgaagacgatcgcgcgactcaaactcactgagactgaactggcgctataccagagccttgtgcta
ttgtggccagagcggcacggcgtccgcggcaaccctgagatccagtgcctcttcaacatgtcaatggcggcgatgcggcacgagatcgagaccaaccac
gcccccctcaagggtgatgtcaccgtgctggacacgctgctggccaagatacccaccttcagagaactatcgctgatgcacctggaggctctgtgccgc
ttcaaggccgcgcacccgcaccacgtgttcccggcgctgtacaaggagctgttctcactcgacagcgtgctcgactacacccacggctaa

>Hv_Contig_1704|heat shock protein 90 [Helicoverpa armigera]
atgcccgaagaaatgcagaccgatgtcgcggaggtggagaccttcgccttccaggcggagatcgcccagctcatgtccctgatcatcaacacattctac
tccaacaaagaaatcttcctccgtgagctgatttccaactcttctgacgctttggacaagatccggtatgagtcactcaccgacccatcaaagctggac
agcggcaaggagctgtacatcaagatcatccccaacaagagcgaaggtacactcacaattattgataccggtattggtatgacaaaggctgacctcgtc
aacaaccttggtaccatcgccaagtctggaacaaaggcgttcatggaggctcttcaggctggcgctgacatcagcatgattggtcagttcggtgtgggt
ttctactcgtgctacttggtggccgaccgcgtgacagttcactcaaagcacaatgatgacgagcagtacatgtgggagtcgtcagcgggaggctcattc
acagtccgccccgaccacggcgagcccctcggacgcggtaccatgatcgtactacacatcaaggaagacctcactgagtatcttgaagagcacaagatc
aaagagatcgtgaagaagcactcacagttcatcggctaccccatcaaactgatggtggagaaggaacgcgagaaggagctgtctgatgacgaggctgag
gaggagaagaaagaggacgagaaggaggacgacaagcccaagatcgaggatgtcggcgaggacgacgaggaggataagaaggacaagaagaagaagaag
accatcaaggagaagtacacggaggatgaggagctcaacaagaccaagcccatctggacccgcaacgctgatgacatcacacaggaggagtacggtgac
ttctacaaatccctcaccaacgactgggaagatcacctggccgtcaagcacttctctgttgaaggtcagctggagttcagagcacttctgtttgtgccc
cgccgtgctcccttcgacctgtttgaaaacaagaagcgcaagaacaacatcaagctgtacgtgcgcagggtgttcatcatggacaactgcgaggacctc
atcccagagtacctgaacttcatcaagggtgtggtcgacagtgaggacctgcccctcaacatctctcgtgagatgctccaacagaacaaaatcctcaaa
gtaattaggaagaacttagttaagaaatgcttagaactcttcgaggagttggctgaggacaaggagaactacaagaagtattatgaacagttcagcaag
aacctgaagcttggtatccacgaggactctcagaacaggtcgaagctggctgacttgctccgctaccacacatctgcctctggtgatgaggcatgctcc
ctcaaggagtatgtatcccgcatgaaggagaaccagaaacacatctactacatcactggtgagaaccgtgaccaggtagccaactcttcatttgttgag
cgagtcaagaagcgtggttatgaagttgtatacatgactgagcccattgatgagtatgtagtacagcagatgagggagtatgacggcaagacccttgtc
tcagtcacaaaggagggtctggaactccctgaggatgaggaggaaaagaagaagcgtgaggaagataaggtcaagttcgaaggcctctgcaaggtcatg
aagaacatcctcgacaacaaagttgagaaggttgtcgtgtctaaccgtctagtcgagtcaccatgctgcatcgtcactgctcagtatggttggtctgcc
aacatggagcgtatcatgaaggctcaggctcttcgtgacacatccaccatgggttacatggcagctaagaagcaccttgagatcaaccctgaccattcc
attgttgagactctgaggcagaaggccgaggctgacaagaacgacaaggctgtgaaggacttggtcatcctgttgtacgagactgcgctgctgtcatct
ggcttcaccctggacgagccccaggtgcacgcgtcccgcatctaccgcatgatcaagctgggtctcggcatcgacgaggacgagcccatccaggtggag
gagaccagcgccggcgacgtgcccccactagagggcgacgccgacgatgcgtcgcgcatggaggaagtagattaa

>Hv_Contig_1708|hexamerine [Helicoverpa armigera]
atggctcgtttggtgttgtgtgttctggcgctcctagtggccgggggcctctcggaccccgtgaggagcaacccctcgcagaaagctgcggacccagtg
ttcctgcaacgccaattggatttgatggccttgtacttccacattcatgaaccgattcatgttgattcttgcatgactatcgctaggtcctggagtctc
gagaaaaacatcgaccactacagcaatgtgactgctgtaactacttacatcgagatgttggagcaccagtggatactacctcgtgcggtaccgttctcg

cttctggaatccgagcacagatttgaagcagtcaccctgtacaatgtgctggtctctgccaaggactacgacacattctacaagaccgccgtttacttg
agaaatatcgtcaacgagaacctcttcgtttacgtcctcagtgttgcaattctgaaccgccctgatactcaaggaatctacatccctcgcctccctgag
gtctttccttcatacttcaacaacggtgaaattatgacaaccgcccagaggatcaacactcacggcttcaacatgcttgagcattacccttctacctac
aaatggacaactatgtagtgatcagatggaatgctactatctggccgtaccacaacagcgaatccacacctgtcgcctacttcactaatgacttcagt
ctaaacaccatctactacaaccttcagcttgctcaacctagttggctgcacagtgaagttcttccattgaacaaacacaggcgtggagaatggttctgg
tacttgcacaagcagctcctcgcccgttattatatggagagattgtctaatggccttggtgaaatccctgagcttggccacgagattgttaagcagggt
tactcttcaggtcttttataccacaatggagtgccgttccctgtaaggcccaattacttcaacttggatcagccccagttcgtaaatgaaatccaagaa
atcttagactacgagcgccgtattcgtgacgccattgaccagggttacgttgttaaccaccttggtgaacacattgacatctgcgctccagaggctatc
gaaatcttgggtaatcttattgaggctaacgttgactctcctaatggcaaatattacaaggacttcatcagcatctggaagaagcttttggcaactcc
attgttcaagaacagcagtaccacaacaattacgtccccctggtcgtcccctcggtcttggaacactatcaaactgctcttcgtgatcctgccttctac
atgatctggaagcgtgtcttgggactgttccaaatgtggcaggagaaacttcctctgtacaagaaagaagaacttgctatgccccaagtggccatccag
aaggtcgatgtagacaagctgatgacatacttcgaacacacttacttgaacgtgtcgtctcacctgcatatgaacgaggacgaagttaaggaagtccac
gaccaagtccgcgtgttggtacaacaccccgtcttgaaccacaagaagtaccaagttcgcgtacacgttaagagcgaggtcgccaagaccgtcctcgtc
aagttcttcttggcacccaaatacgacagccaaggccaagagatccctctccacctcaacacccaaaacttcatgcagctggatgagttcctatatgac
cttccttctggcgaatgcgtcatttctcgtgattctgttgacacttctggcaagaaattgatgtctggcaacgaagtctatgaagcggtagtaaatgcc
atgcaaggcaagggtcattacaccatcgacgagaacctggagaaactcgccgatcatcttatgctgcccaagggtcgcgtcggcggcatgcctttcgtc
ctgatggtctacatctcggaataccgcgcaccgaaggttgctcctgaagccgtctcttacccggcttggtctcttggcctgtctcccaccattcgtgca
ctgaccgacgagccattaggcttcccagtcaacaggcctcttcacccatggcagttggagggagtcaagaacttgtacctccaagatgtgttgatctac
cacaagcataccccccgaaatcgaggttccccacatggaataa

>Hv_Contig_1816|c-Jun NH2-terminal kinase [Helicoverpa armigera]
atggttcagttttactcagtcacactcggtgacagcgtgttcacgatacccacgcggtacactgagctagtgagccgcggcgcgggcgcacaggggatg
gtgtgcgccgcatacgacacggtgacgcagcagaatgtcgccatcaagaagttgtcgcggccgttccaaaacgtcacgcacgccaagcgcgcgtaccgc
gagttcaaacttatgaaactcgtcaatcataaaaatataattggtcttctaaacgcgttcacgccccagaagagcctggaggagttccaggacgtgtac
ctggtgatggagctgatggacgcgaacctgtgccaggtcatccagatggacctcgaccacgagcgcatgagctacctgctctaccagatgctgtgcggc
atcaagcatctgcatcttgctgggattatacatcgggacctgaagccgtccaacatagtggtgaagagcgactgcacgctgaagatcctggacttcggg
ctggcgcgcacggccggcaccaccttcatgatgacgccctacgtcgtcacccgctactaccgcgcgcctgaggtgatcctgggcatggggtacacggag
aacgtggacatctggtcggtgggctgcatcatgggcgagatgatccgcggcggcgtgctcttccccggcaccgaccacatcgaccagtggaacaagatt
attgagcaactaggcactccgtcggcggcgttcatggcgcggctgcagccgacggtgcgcaactacgtggagaaccggccgcgctacaccggctacagc
ttcgagcgcctgttccccgacatcctgttcccgtccgactcctccgagcacaaccgcctcaaggcgtcgcaggcgcgcgacctgctgtcccgcatgctg
gtcatcgaccccgagcgccgcatctccgtcgacgaggcgctgctgcacccctacatcaacgtctggtacgacgaggggaggtcaacgcgcctgcgccg
gcgtcgtacgaccactcggtggacgagcgcgagcacacggtggagcagtggaagcagctcatctaccaggaggtggtggagtacgccgcgccgccgccg
ccgcgcgcgtgctggagcagctggagcgcggcagtgcgctgcgccgcctcgcgcgacaccaccacgcgcacaccaagcatgccatgcaccacgggggtg
aactga

>Hv_Contig_1950|alpha-amylase [Helicoverpa armigera]

atgttccggctcatccttttgcttgcggccgtgtccctggcactggcttacaagaaccctcactatgcatcaggccgcacgaccatggtccacttgttc
gaatggaagtgggatgatatcgctgctgagtgcgagaggttcttaggacctagaggatttggtggtatccagatctctcctcctaatgagaacttggct
atctggtccgctaaccgcccctggtgggaacgctaccagcctatctcctaccgcctcatcacccgttctggtaacgaacaacagttcgctagcatggtg
cgaagatgtaacgatgctggtgtcaggatctacgtcgacgctatcatcaaccacatgactggcacatggaacgagaacaccggtactggtggcagcacc
gccaatttcggcgactggcactaccccgccgttccttatggcagaaacgacttcaactggccccattgtgtcatctctggcaatgattacggctgctgt
cctgatagggtacgtaactgcgagctctccggtcttaaggatttgaaccagggtactgaatacgttcgtcaaatgatcgtcaactacatgaaccatctc
atcggcttgggtgtcgctggattcagaattgacgctgccaaacacatgtggcccggagacatgcgcgtcatcttcgaccgtctacacaacctcaatacc
gatcacggtttcccgtccggcgctcgtccctacatctaccaggaagtcattgaccttggtggcgaagctatcacccgtgacgaatacaccccacttgct
gcagtcactgaattcaaatttggaatggaacttagccgtgccttcaaccgcggaaaccaactcaggtggctggtcaactggggacctgcatggggtctt
cttgcttcggacgatgctctcaccttcattgataaccacgacaaccaaagaggtcacggtgctggtggcaacatcctcacttacaaacaggctaagcaa
tacaagggtgcgattgctttcatgttggcccatccttatggctggcctcagctcatgagcagtttcgacttccacaacactgaagctggtcccccaatg
gacagcagtggcaacatcatctctccttctatcaactcggaccagacatgtggtaacggatgggtctgcgagcaccgttggcgtcaaatctacagcatg
gttgccttcagaaaccgcgccggtaactccgccgtcaccaactggtgggacaacggcagcaaccagatcgccttctgcagaggaaaccagggcttcgtt
gctttcaacaatgactactgggacttgaaccagactcttcagacctgcctgcccgctggtacctactgtgacgtgatctccggcgagaagagcggtaac
aactgcaccggcaaacgcatcacagtcggcagcgacggtcgcgctagcatcagcctaggagccaatgactacgacatggtgttggctattcacgctggt
gatgaatcgagactgtga

>Hv_Contig_2013|arylphorin [Helicoverpa armigera]
atgaagactgtcttgttcctagcagggctcgtcgctctggcgatgggcggcgccgtcccccaccatgagatgaaaatgaagccggttgatgctaagttc
attggctaccagaagaagattctgtcactcttcgaacactctgaacaactggacctgcactccgaatactacaaagttggcaaggattacaatgttgag
gctaacattgacaactacagcaacaagcaagctgtccaagagttcctcgtgttgtacagaactggattcctgccaaaataccacaagttttcgatcttc
tatgaacggatgagggatgaagctattgccctattccaccttatgtactacgctaaggactttgaaaccttctggcaaaccgcggcctgggccaaggtt
tacttgaacgaggagcagttcttatatgcttactacattgctgtggtccagaggcctgacttgaatggtatcgttctcccagcgccttatgaagtctat
cctcaattcttcttcaacaaggaggttttgatcaggatgtaccgcaccaagatgcagaatggcttgatgactcctgaagttggcgctcaatacggcatt
gtcaaggagaacgactactacgtgtactatgccaactactccaactcgttgacttaccccaaccaggaacaacaactgtcatactttactgaagatatt
ggcttgaacgcttactacttctacttccactcacagatgcctttctggtggaagtccgagaaattcaacgtgttgaaagaacgtatgggcgaaattttc
ttctactactaccagcaacttctagctcgttactacttggagcgtctgcctcatggtttgggagacattcctgaattctcttggtactctgaattcaag
actggatactatccccaactgactggaaacttcctgccttacgcgcaaaggagtaacaattacaatatccattctgagaagaactacgaatacattcgt
ttcctggacacctatgagaagacattcttccagttcttgcagaagggagatttcaagactcctgagaaggaaatgaactacgtcggcaactactggcac
atgaaccaagatctgtactccgagcatagcaacaaggaattgcaccagtattcctatgagatcatcgcccgtcacgtgctcggcggcagtcccaagcct
ttcgacaaatacgcattcatgcccaccgctctcgacttctaccagacttctctccgtgaccctgcattctaccagctctaccagagaatcgtcgactac
ctcatcgcctacaaagagtacgtcaaaccttattctcacaacgaccttcacttcgtcggtgttaagatcaatgacgtgaaagtcagcgaattggttact
tacttcgatttcttcgacttcaatgccacgagcagtgtgttctacagccaggaagagcttacatcttacccaactggattcgttgttcgtcaacctcgc
ctgaaccacaaaccattcactgtttctgttgaccttaagtctgatgtagcgtctgatgccgttttcaagatcttcattggacccaaataccacgccaac
ggttacccagtaaacattgaagaggactggatgaaattctacgaactcgactggttcgtgcagaagcttgtccccggcgaaaacaagattgagcgcaag
tccagcgaattcgctttcttcaaggatgactcaatccccatcaacgagatctacaagtggttagaccaaggcaaggtaccttacgacatgtctgttgtg
cccgacagcatgcctaggagactgatgctgccaaagggtacccctggtggatatccattccaaatgttcgtgttcgtataccattcaacggagttaaga

agggagaagacgtgttccagaactaccttgctgacaacaaacccttcggttatccattcgaccgccccgtgcaggaagcttactacagacaacccaaca
tgtacttcgaggatgtacagatctaccacaaggacgcgtatttaccgtatgagatga

>Hv_Contig_2041|carboxyl/choline esterase CCE016a [Helicoverpa armigera]
atggcgtcggtgataaatatcgtggcgcttctagcagtcctgtcagtgcaggcgcttgctgtgcgagtgagagtgagtgatggcttgctggagggagag
cgagtgttcaacgagtacggcggcggtcagtactacagcttcaaaggaatcccatatgcacagcctccgctaggaaacctcagattcaaggctccacaa
cctccaacaccatggagcaatgttcgcagcgctaaggcatttggttccaattgtctgcagtacgatttgttcatggacaagggaagacgaagtggcaat
gaagattgtctgtacctcaacgtgtacacaccagagatcactcctagcgagcctctgccagtcatggtctggatccacggaggaggctttgtctccggc
agtggtgatgacgatgtatacggacctaaattccttgtaagacacggagttattgtagtcacgattaactaccgacttgaagctcttggtttcctttgc
ttagacacagaagaagtccctggcaacgcaggtatgaaagatcaagtggccgcgctcagatgggtcaacaggaacatcgcccaatttggtggaaatcca
aacaacgttactattttcggtgaaagtgccggtggagtcagtgtctcctaccaagtaatctcaccaatgtctaagggattattcaagagagccatagct
cagagcggtgtcagtgtcggttattgggcacaggcgtataggccaagagaaagaggctttgctttaaccagaaatttaggattacatactgacaatgtt
actgaagtttacgagttcctaaaagtccaaccagctgaatcacttgtacaagtcaaagctccagtaacttactctgaaaacgaaaggcccaacgttgaa
gtttactttagcgtagctgatgaaaaacaattcggcaacaacgagagatttttctacggagatatggtggatgcagtgtcaaacaatgtccatgaagga
gtagatattatgacaggatacacggctgatgaaggactcatgggtgttgccattttcggtcaagtcaattccagtttggaacaagctaggaagttccca
caattctttgtgtcctacccaatgtcgctcaatttgccagtagatgatcaattggaacttggagcaagaatcagagattattatttcaaaaaccagatt
tcaattcctgatgattgggagggtttgtcgagatactatggcatggacatattctcattccccactttaaggtggattaagctgattgctcgcactaat
aagaacaatgcctatctgtacaaattcaccgtcacatcggaactgaacaaggctgctcatatgatgggactagctgacatcctcggagacaaggaggtg
gttgcccacagcgacgatttgatttacttgttcaattcgcgacacttgcctattctcaacatgaccacgacccccttcatatacatcgagcatgttact
aagctgtggaccaatttcgcgaaatatgggtga

>Hv_Contig_2119|protein phosphatase 2 regulatory subunitalpha isoform [Helicoverpa armigera]
atggcagcaagcgactctgggacggatgagtcattgtatcctattgcggtgctcatcgacgagttgaaaaacgaggatgtccagcttcgtttgaactct
atcaagaagttatccaccatcgcgttggccctcggagtggagaggaccaggtctgagctcatcccgttcctgacagagaccatttacgacgaagatgag
gttttactcgccctcgccgaacaactcggcaacttcatcaacctggttggtggtggtgaattcgctcactgtctcctgcctcctctggagtctctggcc
accgtagaagagaccgtagtccgtgacaaggctgtagcctcactccgtgccgtagctgctcatcacactcctcaagctttggagcagcactttgtaccg
ctagtccagcgtcttgctggtggtgactggtttacttcccgtgcctccgcttgtggattgttcagtgtatgctacccccgagtatcagccccccgtgaag
gcggagctccggcaacatttccgctccctctgccaagatgacacgcccatggtccgccgcgccgccgcctacaagctgggggagttcgcccgcgttgtg
gaagtggagtacgtcaagagcgacctgatacctatgttcgttactttggctcaggatgagcaagactcagtccggctgttggcagcagaagcgtgcgcc
gcagtagccgcgctgctgccgcccgaggacatggagcagctggtgatgccgacggtgcggcgcgggccggcgacacctcctggcgcgtgcggtacatg
gtcgctgacaagttcgtggagctgcagcaggcagtaggacctgagctggcgcggtcggacctggcgcagatcttccaggcgctgctcaaggacaccgag
gctgaagtacgcgccgccgccgctggcaaggtgaaggacttctgcatgaacttggacaaggcgcatcaagagcacatcatcatgacaatgatcttaccg
caaatcaaggatctagtctgcgacgcgaaccagcacgtcaagtccgcgctcgcctccgtcatcatgggtctgagccccatcgtcggccgtcagaacacc
atcgaacacctcctgcctttgttcctcactcaactgaaggacgagtgccctgaagtcaggctcaacattatctccaaccttgagtgtgtcaacgaggtc
atcggaatccaacaattagtgcaatctctcctccccgctattgtggaactcgctgaagacacaaaatggcgtgtccgtctagccatcatcgaacacatg
cctttactcgctggccagctaggccaggagttcttcgatgaaaagctgaccggtttatgcatgtcttggctcatcgaccacgtttatgctatccgtgaa
gctgccaccctgaaccttaagaaattagtagaacaatacggatcacagtgggccgagactaatgtcatccccaaagtactggctatgtcccgcgaacag

aattacctgcacagaatgacgtacttattctgcattaatgtgctgtctgaagtgtgcggcaaggatatcaccaccagggtactgctgcccactgttctg
tccatggcagatgacaatgttgccaacgtgagattcaacgtcgccaagactctgcagaaaatggcgccattcttagaccccgcggtcatccagccacaa
gtgaagccagttctcgagaaactgaacgtagatcctgacgtggatgtcaagtacttcgcctccgaagccattgccggcatcgctggctaa

>Hv_Contig_2353|cathepsin B-like cysteine proteinase [Helicoverpa armigera]
atggccgcttcgcgtgcaacgtttgttgcgctcgtgtgcgcactcgcgctggcctccgccaacctgcagaatccgctcagcgacgatttcatcaatctc
atcaacacaaagcaaaactcctggaaagctggtagaaacttcccagagcacacgcccttcgcacacatcaagagattagcgggcgtcctggaagattac
catttggcaaaattaaccaaggttgaacatgaagatgaattgattgcaagtttaccagagaactttgacccgagggacaaatggccgaactgccctaca
ttgaacgaagtaagggaccagggatcgtgtggtagctgctgggcgtttggtgctgtggaggcaatgaccgatcggtattgtacttactcaaatggaaca
caacatttccatttctccgctgaagatcttttgagctgctgcccaatctgtggtctcggatgtaatggaggtatgccaacgctagcttgggagtactgg
aagcatttcggacttgtgtctggtggtagctacaactccacccagggttgcagaccgtacgagattcccccgtgtgaacatcatgttcctggcaataga
atgccctgcaatggtgattctaagaccccaaaatgcctaaaaacctgcgaatcaaactacaacgttgactaccacaaagataagcgctacggcaaacat
gtttctctgtgtcaagtaaggaagaccatatcagggctgagttatttaagaatggaccagttgaaggtgcgtttacagtatactcggacttgctgaat
tacaagacaggtgtttacaagcacacgattggcgacgctctcggtgggcacgctgttaagatcttgggctggggagtagagaacggcaacaagtattgg
ctcatcgctaactcatggaacagtgactggggagacaatggattctttaaaatccttcgcggtgaagaccactgcggaattgaaagctctattgtagcc
ggtgagcctctctttgttcaaaattag

>Hv_Contig_2390|serine/threonine protein kinase Akt [Helicoverpa armigera]
atggcggaggcggcgcccgggaacatcgtgaaggagggctggctgcagaaacgcggcgagcacattcgcaactggcgcgaccgctacttcatcctgttc
gacaacggcgacctggtgggcttcaagacgcagccggagcggaacaactaccgcgacccgctcaacaagttcacggtgcgggactgccagatcatggcc
gtggacaagccgcgcccctacacgttcaccatccgcggcctgcagtggaccaccgtcatcgagcgcaacttctccgtggacaacgagaaggaacgcgag
gagtgggtggcggccatccgctacgtgtcgtcgcagctcagcgcgggcgggcccagcgccgccgcgctgcccgagagcgacgaccgcgacatggcacag
ctcggcaccagcttccgcgaccctcgtcgcatcacgctggagaagttcgagttcgtcaaggtgctgggcaagggcacgttcggcaaggtggtgctgagc
cgcgagaagggcacgggtaagctgtacgccatgaagatcctcaagaagaacctcatcatacagaaggacgaggtggcgcacaccatcaccgagaaccgc
gtgctcaagaagaccaagcaccccttcctcacggcgctgcgctactcgttccagacggcggaccgcgtgtgcttcgtgatggagtacgccaacggcggc
gagctgttcttccacctgtcgcgcgagcgctccttctccgaggagcgcacgcgcttctacggcgccgagatcgtgtccgcgctcggctacctgcacgcc
gagggcatcatctaccgcgacctcaagctcgagaacctgctgctcgacaaggacggccacatcaagatcgccgacttcggcctgtgcaaggtgaacatc
acgtacgggcgcaccacgaagacgttctgcggcacgcccgagtacctggcgccggaggtgctggaggacacggactacgggccggccgtggactggtgg
ggcacgggcgtggtgatgtacgagatggcgtgcggccgcctgcccttctacaaccgcgaccacgacgtgctgttctcgctgatcctgagcgaggaggtg
cggttcccgcgcgcgctgtcggcggcgtgccgcgcgctgctggcggggctgctgaccaaggagccggcgtcgcgcctgggcgccgggcccgacgacgcg
cacgagatcatgaaccaccccttcttcgcgtccgtcaactgggccgacctggtcgccaagaagatcccgccgcccttcaagccgcaggtggagtcggag
accgacacgcgctacttcgactcggagttcacgggcgagtccgtcgagctcacgccgcccgagagcgacgccagcctcgccaggatacaggaggagcag
ttcccccagttctcctaccaggacatctgttcgtcggcgcactcggcgctgtcgcagcactcggcgctggccgacaagcgccagtag

>Hv_Contig_2546|receptor for activated protein kinase C [Helicoverpa armigera]
atggctctctgtgtttgtaaatgcctaaatgtaacgcttgaaggtgataaacttgaagacaatgtcgatattggaaactggagttatcatccatggaa
caaaggatattttttcagtgagaaactgatgtcttgcactctgagcagcttgaagagtcaggtggctcagcctgcactggtggaccagcgagcactg

ggcaggtggaccatacactcctgcctggcttgtggacagcccacgcatgccatatcacatgacaaaaacggaccttatgcattgcttgtttgtaaatct
aatcagaccacacaagatcggataaacaatctgaaaaagtcaaataactattcgccagtattcaacttattagtgccagaagtaacaaatgatatagaa
atgaaggaaaatgtggacacaaacaacttcataaacagtgacaggaatgtgtggctaccaactcagcaggtgataggcactctcagtaaacagttgaat
caaactctgcagtctcagctggaagcggttgaggagtctgtcaggcagttcagagatcagaagtatgctgagtttgaggcttacagagaragagcacat
agggatcacaaaattttagctagtatcataagcaaggcacgcagcacagctgacagggacagctggcggatcgacacaaacattgacaatggacyaccs
gtctttttactttgagacggaggctagtgtgttggggcttagccgctcatcaagtttttgtgaaaatgactgaaactttaaagcttagaggaaccctc
tgtggccacaatggctgggttacccaaatcgcgaccaaccctaaataccctgacatgattttgtcttcttctcgagacaaaaccctcatcgtatggaag
ctgaccagagacgagactaactacggtgtcccgcagaagcgtctgtacggtcactctcacttcatctcggacgttgtgctctctagcgacggcaactac
gctctgtctggctcttgggacaagaccctgcgtctgtgggatctcgctgccggcaagaccaccaggcgttttgaagaccatactaaggatgtcctctcc
gtggcgttctcagttgacaaccgtcaaatcgtgtctggctctcgggacaagaccatcaagctgtggaacacactggctgagtgcaagtacaccatccag
gatgatggccacagtgactgggtgtcctgcgtccgcttctcccccaaccatgccaacccgatcattgtgtctgctggttgggaccgcactgttaaggtc
tggcaccttaccaactgcaaactcaagatcaaccaccttggtcactctggctacctgaacacagtcaccgtttctcctgacggttctctctgcgcttcc
ggtgggaaggacatgaaggccatgctctgggacctgaatgatggcaagcatctgcacaccctggaccacaatgacatcatcacatcattgtgcttctca
cccaacagatactggctgtgcgccgccttcggaccttccatcaagatctgggatctggaaagcaaggagatggttgaagagctcaggcctgagatcatt
aaccagacccagacctccaagtcagacccaccccagtgcctgtctctggcgtggtccacagacggccagaccctcttcgctggctactccgacaacatc
atcagagtctggcaggtgtcagtctcagcacgataa

>Hv_Contig_2626|serine proteinase-like protein 1 [Helicoverpa armigera]
atgttcggtccccctccatccactaagaaccctagccagggcttgctttcaaccgcagctccaatcggcgacaagacgcaagggtccactatagtacca
ccaataaacaataatgcagctgaagtggacctgtcgtgcactatgcctgatggtcaggcgggagagtgcgtcagctattacctttgtaacgtgaacaat
aacacggtcatcgttgacggcgctggcgtcatcgacattcgtgtccgagacggtccctgtgagtcgtatatcgacacctgttgtctggtgcctgacaag
agacctaaagaccagcccatcaaacctcagcctaagcctgatcagccacagcgcaaaggatgcggttgggcgaaccctgacggcgcagatctgcggaca
actggtgaaaccaatggcgagaccaaatttggcgagttcccatggatggttgccatcctcaagatcgaaccagtcaacgaaaatgatcccgaaggtcag
aaactcaacgtgtacgtcggaggagggtccctcatccaccccagcgtggttctgactgcagctcactacgtggccagcgctaaagccctgaggatcaga
gccggcgagtgggacacacagaatactaaggagatctaccccttccaagaccgggatgtcgcgtccgtggttacccatcaagattttaacaaaggaaac
ctgttctacgatgtagcagtactgttccttgcgcagccagtggacctggctcctaacgtaggagtagcgtgtctgccaccagcgaaggttcgtcaggag
gatggcacccggtgcatcgctaccggctggggtaaagacaagttcggcaaggagggacgctaccaggtcattttgaagaagatcgagttaccagtagtg
aagcacgacaaatgtcagacagctctccgtaggacccgcctgggcagattcttcgagctgcactcgaccttcatgtgcgctggaggagaacccggcaag
gatacctgcaagggagacggtggatcacccctgtcttgtcctattgagtacgaaaaagaccgttacgttcaaagcggtatcgtcgcgtggggcatcggc
tgcggcgaggacggcactcctggagtctacgtcgacgtctcagtcctcagggactggatcgatgacaaagtagccggcaagggctatgaccctaagact
tacacttactaa

>Hv_Contig_2658|carboxyl/choline esterase CCE017a [Helicoverpa armigera]
atggaacatgacaacccaatagtgaccgttaaacagggtaaattgaagggagcggtaaagaaatgtttggacggaagcccttactacagcttcaaagga
atacgctatgcagaaccacctgttggagagttacggtttagggcgccagtaccaataaagccatgggaaggcataaaggatgctatagaacacggcca
gtgtgcccgcagtttgacatggccatcttagactatgtggaaggcaacgaggactgtctcagcttaaacgtctacacaaagtctttacaacccagctca
aaactacccgtcatggtctacatacacggtggagcttttctatcagggtcaggtaactcagagacctacggacctgaatttttattcttacatgatgtc

atactagtcactattaattataggctcgaagccctaggtttcctttgcctagacactcctgaagtaccaggcaacgctggtatgaaagaccaagttgta
gccatgcgttggattaaagaaaatattagcacttttggaggcgaccctgacaatatcacgttgtttggagaaagtgctggtggagcctgcgttaactat
cacatgatctcaccgatgtctgaaggactgttccacaaggcaatagctcaaagtggagtttgtttacaatattgggcgcaagcacatcaacctgtcgaa
agagcgtttagatctggaaaagttctaggcaaagacactaaggatccaaaagaacttctggaccatttaagaggactccctgctcttgatttggccagg
cttacgataaaaacgagaactccggacgagaaatatagagggctacccatctactacactccaacggtggaaaagaaatttgaagggcaggaacagttt
ttgactgaagacccactagaaatgattatggcaggcaaggtgaagaaaatacctttttatgtgcggttacaatactgctgaaggaatattaatagctggt
gatcatctgaaaaagttgaatgttctgaataaagaaccatcctaccttgtcccgagagacatagcaacgatagtttctaaggaaaaattggaggagttc
ggtcaaaggattaaagatttttatttcggaggtagagatgcgactgaggctgatttacggccgatggtagacttccatacagatattaatttcatgtac
caggctcacagacttgtgcatctgtaccataagatgggtcctacatatatgtaccgatttaactactgcactgaattgaatataatgaagggcgtgttt
ggtgaaactagcgtggaaggcgcgtgtcatgtagatgatctattttatatgttccatacaccgatgactcaaagtatgtatgaagagaaggaggaaata
agaaaagctgtgtataatgttaccaaattatgggctgattttgctaagactggaaatccaacaccaacaaagggcagcatagactggcggccttacaat
tcaacaaaagagtatttactgatagacaaagaaatcaaaatgtccggtaacttggagaaggaaagaactgaattctggaacaaaatgtacgctgaggtt
ggtttgcctgccatcactaaatctaatttatag

>Hv_Contig_2722|carboxyl/choline esterase CCE002a [Helicoverpa armigera]
atgtttgcgtacgctttgtttgtgttgctagttgcgtacgcgcatggtttgcaagtgagcactattgaaggcaccgtggaaggcgggaaggcgtctgat
ggagattattatgctttctacggtattccttacgctgggtggacgacaggtcgaagccggtttaaggcacccgttctaccttcgtggtatccaggagtg
ttccacgccacagaaaacaccctgatctgcgcacagccaacgtccaggggtctcgtcggttccgagaactgcctcacccttaacgtgttcacgaacaat
gtcacaagcccgaaaccagtcatcgtgtggatcaacgcagaggagtacaccaacacgaacactgaaatattctcatatcgaacatttgtcgaaaataat
gttgtctttgtgtctatgaacttcagactgtcgattttcggattcctatgcttgggcgtccaagaagcacccggtaacgcgggtttgaaagacatcatt
caaggactgaaatgggtcaaggacaacattagcaattttggaggagatcctaagaatgttattttaatgggtcatgcatctggagcagcgatggtggac
ttgataacgctttcacctcaaagtgaaggtcttgttcataaggctattattctgagtggttcaagtctagctccatgggcggtatcttacgatcctgtc
cagtcggcccaacttgtgggagataaacttggttacggaggcaagacaagagatgacttagcaaaacatttagtcaaaaccgatattaaccttttacat
gcagccttggacacctccaaatacttcaataatacaccgcaatttgcaccatgtattgaggatcccaaagttagttctaacttcacagtcctttccgaa
gctcctatcaatattctgagatctgaaaaataccctcaaatccccgtcatatacggatatacaaatagggaaggaacaatgagagcagcgcaggcagac
tatggcaactggctgaactacatgcagacaaactttacaaacttccttccagtcgacatggacttcggtaaaaatagaacagctgtagcggcggccatc
cgtgaccactactttgccagcaaggcgatcagcatggaaaccattgaagacttcctggattatgaaggagacaccctcgtactcctgtctgtaattaaa
gcagctaaggaaagggctttaagctctaagagtgaagtaaggttgttggagttcagttatttgggtactatgaactctcattgggtgcacaaccagatt
cctttaagtggagctaaacacggatccttcctaaacttcttgtttggttatgacctacgtcccgttgacgaggcagtcagcaaatcactcgtgaagcgc
ttcgttgacttcgcgtataacaagaacagcgcgaccgccagtgcagctagtaacatcaacattgttaacgtagcacctactgtcaattctaccattagc
aatgcaacagccaacctcaccaggatcaacgggacatctacagcagcaataactaatggaacctccaatggaactgttgttgccaataatacaaataat
gccatccccgtcaccgtaactaatgattggcccacagttagaccgaatctcaccaactacctcttctatggtggcaatggggctcccactgttaacaac
ggctttatctacgttgaggagcccaggtttaaccctcacgtgcaacgaatgtccttctgggataatctttatgctaaatattatattactcctacactt
cctagttcttctagtgttacaattagttgtttcgtgtctatcgtcctgtgtcagttagtcgttcatttgttttaa

>Hv_Contig_2820|ADP/ATP translocase [Helicoverpa armigera]

atgtcgaacctcgctgatccggtcgcgttcgcgaaagatttcctcgctggcggtatctccgccgcggtctccaagactgccgtggcacccatcgagcgt
gtcaagctgctgctccaggtccagcacgtcagcaaacagatcgccgccgaccagcgctacaagggtatcatcgatgccttcgtccgcattcccaaggag
cagggtcccctgtccttctggcgtggtaacctcgccaacgtcatcaggtacttccccacccaggcgctgaacttcgccttcaaggacaagtacaagcag
gtgttcctcggaggcgtagacaagaacacccagttctggcgctacttcgcgggtaaccttgcgtccggtggtgccgccggcgcgacctccctgtgcttc
gtgtacccgctcgacttcgcgcgtacccgtctcgccgccgacgtcggcaaaggcgacggccagcgtgaattcaagggactgggagactgcttgggcaag
atcttcaagtctgacggtctcaccggtctgtacagaggcttcggcgtatccgtgcagggtatcatcatctaccgtgcctcctacttcggattctacgac
accgcccgtggcatgctgcccgaccccaagaacacaccactcgtcatcagctgggccatcgcgcagactgtcaccaccgtcgccggtatcatctcgtat
cccttcgacacggtccgtaggcgcatgatgatgcagtctggccgtgccaagggcgacatcttgtacaggagcaccctccactgctgggccaccatcgcc
aagaccgagggaactggcgccttcttcaagggagccttctccaacgtcctcagaggaaccggaggcgccttcgtgcttgtgttatacgatgagatcaag
aagcttctctaa

>Hv_Contig_2827|cytochrome p450 CYP4S1 [Helicoverpa armigera]
atgctgctgtcactggcactggtgggcttcataaccttcatgctgtggaaactcctggctgaggaggagaatcctctggacaagctgccgggaccgaag
cgctatcctatcataggatctgcccttgaattcttcaggatgaatccacgagacctgtttttgaaaatgcgctatttcgccaaagtatatggcgataga
tacctcatcaagatcttggggagacggatcctgcatgttaataatatcaaggacgttgaggtcgtgttatctcactcgcgtaatataaagaagagcaag
ccgtacacatttctggaagcctggctcggtacgggcctattgctgagtaatggtgcaaaatggcacaagcgccgcaagatcctgactccgaccttccac
ttcaacatcctgaagagtttctccattgtgatgaaggagagaagtaagggcctggtggagaagatcaagggtttggagaacacagacgtcaatctactg
cccctgatcagtgattatacactgtacattatttgtgaaactgccatgggcacccaactggactcagacaatcagcaaaaaccttagaatacaagagc
gccatcttggagatcgggagcctcctcttctctcgtctaacaagagtatggctccacaacgaatacctcttccgaatgaacccacttggtagaaggttc
gagaaaacgctagaaaaagtccactcattcgccgatgacgtcatcacggagagaaagaagaattggaagccgggacagagtgatttcacagcagaagat
tcggttgggggaagaagagactggctatgctggatttgctgttggaggcggagagtaaaggagaaatagatttagaggggattagagaagaagttaat
acttttatgtttgagggtcacgacaccaccgccatggcgttagtccttggcctgatgctgatagcagaccacgaggaagtccaggaccgtatattcgaa
gagtgtcagaagatcttccctgatgctgagagcacgccatcgatgtcagacctggctgatatgaagtatctggaggcagttatcaaggagaccttgagg
ctgtaccctagcgtgcccttcatagccagggagatcactgaggacttcatgttagatgacctaaaagtaaagaaaggtgctgaagtgtctatccacatc
tacgacctgcaccgtcgagctgacctgttccctgaacctgaagccttcaagccggagcggttcctcagtggggagcctttgcatccatacgccttcgta
ccatttagcgctgggcctcggaattgcatcggtcaaaggttcgcgatgttggaaatgaaatgcgtgctgagtggaatttgtcgcaacttcaagctgcag
ccgagtgtcaaaggagccaggcctgcgctcctagctgacatgttgattcgcccggctgagcctgtctgtgtgaaatttattcgtagatag

>Hv_Contig_2829|cytochrome P450 [Helicoverpa armigera]
atgattctagtcctggtatgggtggcggtgctgatcgccgtggtggtgctgtacctccgccaggtctactccaagttctctcggtatggcgtgaagcac
ttcaagccggtgcccctattcggcaacatggccaggatcttcttccgactggatcacttcattgatgatgtcatgaggctctataacagctttcctgaa
gagaggtttgtgggcagatttgagttcgtcaatgagttggtgatgatccgcgacattgagctggtgaagaagatcaccgtcaaagactttgaatacttc
ctcgatcaccgctctatcttcagttccagtgactcattcttctccagaaacttattctctttgaaaggccaagaatggaaagacatgcgatcgacttta
agcccagctttcaccagttccaagatgcgtctaatggtgcccttcatggtggaagttggcgaccagatgatggcttcgcttagaaagaagattaaggag
tccgaaaacggctacatagacatcgaatgcaaggatttgacgacacgttacgccaacgacgtgatagcctcctgtgccttcggtctgaaggtggactct
cacaatgacactgacaacgagttctatgccatgggcaagctgtcttccactttcaacttccgtcagatgctcatgttctttctacttataaatgcccct
aaagttgctaagttcttcaagctggactttctttccgaggcatcgaagaaattctttaggaatttagttcttgataccatgaagaatcgtgagatgaac

catatcattagacctgatatgattcatttgcttatggaagctaagaaaggtaaactgacccatgaagaaatcaaatctgatgatgtaactgctggattt
gcgacagttgaagaatctgctgtgggacagaaagaaattactagagtgtggtctgaagatgatctcatcgctcaagcagtactattcttcatcgccggt
tttgagaccgtgtcagctggtatgtcattcctgctgtatgagctggctatcaaccctgaggttcaggagcgactggcacaggagatcaaggagaacgat
gccaagaatggtggcaagtttgacttcaactccattcagaatatgcagtacatggatatggttgtgtctgagctgctgcgcctgtggccacccgggata
gcattagacagagtctgcactaaggactacaatttgggaaaacctaacaagaacgctgaacatgattttattatccgcaaaggcacaggtatcggtata
ccagcgtacgccttccaccgcgaccctcagttcttcccgaatccagacaagttcgaccctgagaggttctccgaggagaacaagcacaccatccagagt
tttgcttacatgcccttcggtattggacctaggaattgcataggttccagatttgctctctgcgagatcaaagtgatggcataccagattctgcaacac
atggagctctctccctgtgagaggacctgcataccagccaagcttgataaagagaccttcaacattcgtctgaaaggaggacactggctgagatttaga
cctaggcagtag

>Hv_Contig_2846|carboxyl/choline esterase CCE001h [Helicoverpa armigera]
atgaagcggttgttggtagttttatgtgtagtagtgttaagtaactgtgatgataatgagtggcggcaagtgcgaactgcgcaaggacacgtgcgcgga
cgcagggaccccgagggtgggctttacgccttctataatataccctacgctattgctccacagggaagagagaggtttaaggcaccttatcctccacca
gtatggctacaacccctggacgctgtagacaaaggcatcatttgtatgcaaagtccttcaccattcattgacaccaagtccaaaattatgcaagaagac
tgtcttattgccaatgtctatgtccccgatacggaagataaaaatctaccagtcatagtgtacatccatggtggggcgttcgaagtaggatatggagac
atggcaaaacctacaaaattggtgaagaccaaaaaagttatagtagtcaacttcaattatagacttggagtacatgggttcctgtgcctgggatccaat
aacgctcctggcaacgctggtctaaaggaccaggtggcattactccgctgggtgaaaaaaaacatcgctaatttcggaggcaaccccaatgacgtcaca
atagctggttacagtgctggttcggtttctgcagacctcttgatgctttccaaatctgctgagggtctatttaacaaagtgattctagaaagtggggca
agcgtaggctcaattgcagttcaaatggatcctctggaaaacgctaaagcctacgcaaaattattgaactttactgacgttgatgactttcacgcctta
gaagagttttatagaacagctcctttagattcaatactttgaatacatttcaaaaagaaaagatagtaccatttctttccgccctgcattgaacgc
gaaggaaaaggagtatttctagaagattctccagtcaacataataaagaaccgtaaatacaaaaaggtgcctattttgataggtacaagcaacttggaa
ggatcattatttatacctaactttggaaaatggaaagaaaacttgaatcaaaaattttcagatttcctcccagctgatttgcaattcgaaaatgatgca
gaaagggaagaaattagtaaaaaaattaaagaatttatttcgatgacaagcctatagctgaagaaactattctatcgtaccttgattactttggcgat
attacttttggttacccaacctggaaccacgttaagctacatgtagaagctggtcatgatcaaatatacttgtatgaatattcttttgtggatgacagt
actcctgaggtaccgttcacgaaggttcgccgatccgggcactgtgctcagactatggcagtacttgatggaggtagccttatggtgcctgatgaaagt
aatatatctgaagaatacaaacgaatgaaagcttcaatacgtgaaatgtggttcaacttcgcagtcaatggaaaccccgtaccagaagaatcaaacctg
ccagcatggcctgtggctaacgcaaatgggccccatacatgtctttgggagatgttattgaactgggaggagtttacctaggaaaacgaggacaattc
tgggaagaattataccagaaatactataaagcgccggtggctccaccacagccgccgccggagaaacacacagaattgtga

>Hv_Contig_2992|cathepsin 1-like protease [Helicoverpa armigera]
atgactaagttagtctggttattgtttgggctggcggtagccacacaggctttaagtgtgaaaagggaaatcaaagaaaagaagaatctcgaaggttac
aagaaactccaatggcctagtcaataccagttaaaagcgacgcgtatgtcgcttactgacaacatcattgaaaactatgccgtatggagaacgtcaaag
caatcgagaattgattacaatgatggccacgtgaaatcaattgtgagagccaagaacaatcagttccgttatggtatagagtatcagatccatcctgaa
gcgtcaaaggaccaagaagtggaactggtatgctatgctatggaaggtaacagatggcacaggccatcaccctcgactattctacccgatactgatgag
ttcgagtatgatgatgaggagctgatgaatgataaagactgctacaaattcgttaataacgtgaaggatgaacaaactgactcacagaagaccctttgg
gctgagtacaatgaagatgacaaagcttgggttcctgtcagatacgaagtcttggaattcaactcctggttgggatcgaaggacaaacacgaaatctgg
gatttctctgattacgctaaagctttctccttccgcagtgttttcgatgttgaagactacggttgtaatgacgctgttactcacacccaagatagtgag

gaagtaactaaacatttgttgttcatggatgctgagaatgacaaacatgtcgaccatgtctttaaagcattcaaaaatacccacaacaggaaatactct
gatgccaacgagcacgctatgagaaggagtctattcaagaggtccatgaggcaagtaattgagaacaaccgtcaaaacaaaggcttcaaactcgccatc
aaccagttcgctgacaagacacctgaagaaatggaaaggtacatgggtctgatgaggcgccctgaaggaaaagttggaaccatacctttcccttacgat
gaagcaaaactgaatgagatctccagtgatatacctaaagaatatgacgccaggcttgagggtcttgtgtcaaacattaaaaatcaagaggactgcggt
tcttgctggaccttcggaactaccgctgcggtggaaggagccctggcgcgtatcaatggtggaaaactgctggctttgagcaaccaggctctcgtggac
tgcgcttgggctttcggctccgctggctgcgaaggcggcactgacaacgcggcctacgagtggatgatggagtacggactgccgactgtagtagaatat
ggtccatataccaacaaggatggcgagtgtaacatcttgaacatgacaaagacttaccctattcgtggatacgtggatgtcacccccccagagtattgga
gctttaaaggttgccctagtgaaccatggtcctctgtcggtatctgttgacgtcaccgatgcattcgctaaatactcttctggaattttctacgatcct
gagtgcacacaacaacgcctgaaccacgaggtgactctggtagggttcggcgagcgtgatggagacaccttctggatcctgaagaactcctggggaccc
caatggggtatcgacggctacatgtacatctcctctagggacaacgcctgtggtgttgctacggaacccacctacgttgtattctaa

>Hv_Contig_2996|glucose oxidase-like enzyme [Helicoverpa armigera]
atgattctggcggaacagctagattgcggttgcccaatcgtagtagaagggccgtcgatcctgaactccacagcatgcagcgggacctatttgttcatg
atgctcctacaaggatatcagtggggacgctgcgatatcgtcactccttgcaagagaatcgagtcgatagatgaaacggaaccagaatatgacttcata
gtcgtgggagctggatcagcaggatctattgtagctgggagactaagcgaaaacacttcgtataacgtcctacttttggaagctggagggcctgaaccg
ctgggtgcccgtgtcccatcattttacaaaacgttttggggtaacgatgaggtagactggcaagcgcgaactgtacctagtcccaacttctgcctcgat
caacaaggacttgggtgccaatggccgctaggaaaaagcttaggaggaaccagtctcctcaacggtatgatgtaccatcgaggccaccccgctgactac
gacacctgggtgaaagaaggcgcaactgggtggtcctgggacgagatcaaaccattcatggacttggcggaaggcaatagacaagtaggaagccttgtc
gatgggaagtaccactcggaaactggacgcatgccaattcaaacgtttaactaccagcccccgaaaaaaaggatttaatagaagcaataaaccaggcg
ggcttgccggtcatcaaggacatgaacgacccgaacacacccgacggattcgtagtggcacaaacctttaatgataatggacagcgatacaccacagcc
cgtgcatacttggtaccgaaatctgagcgacccaacctgagcgtcaaactctatgcccacgtgactaaaatcctcttcgatgggaacaaagcagtggga
gtggaatacgttgaagccaatggcaatacgaaaactgtgaaggctactaaagaggtaatcgtatcagcaggagcgttaaccagtccaaaaatcctaatg
cactccggagttggacctaaagaagtcttagaaccattgggcatccctatggtagccgacctgcccgttggcaagaccctgaagaaccactgcggagcc
acgctttacttcatgctgaagagcaccaacaacaccgaagctctggactggagtgcactgaccgagtacttgctaaaactagacggtcctatgtcttct
actggactaactcagctcactggtctcctatactcaagctacgccaacaaggattgcaagcagcctgacctacagttcttcttcaacggtctgtatgcc
gactgctccaagactggtgttattcgcgaacctgctgacgactgcagcaatggctaccaagtctcagcgaacgccgtagccctactcccgcgcagcgta
ggcaacgtgaccatcaactcgacagaccccttcaagcaggcgctgtactaccccaacttcttctcccaccctgacgacatgaacatcgtgatggaaggc
cttgattacctgagacagatctttgagagtgaggtgctgcaagagaaatacaaagtagagctagacccggaatacacaacacagtgtgacgactacgaa
acctggtcaagagactggaaggagtgcatgattcgcctacacacagacccacagaaccatcagctcgctaccaacgctattggcaaggtcatagaccca
cagcttagggtttaa

>Hv_Contig_3083|protein disulfide isomerase [Helicoverpa armigera]
atgttgcacacatatttcttaggtgttttattatgtgtggggtccgggctcgccttgtacgattcgagttcaaatgtcgtggatctgacacccagtaat
ttcgacaaactagtcacagatagtaatgatgtgtggttagtggaattctacgctccgtggtgcggccactgtaagaacttagttcctgaatacaagaaa
gctgccgatgcattgaagggcattgcaaaagtaggagctatggacgctgagcagcacagaagctacgccaagaactatggagttaccggcttccctacc
ctgaagatcttcacaggcagcaagcacactccatacaaaggtgcgaggacagcaacagccatggtcgacgcctgtcttgaagccctgaagaacaaggca
tacggtcgtctcggtgccaaacccgaccgctcatcagaaaagtcggactctgacgtgatcacgctgacggatgagaacttcaagaagctggtgctggac

agcgaagacctgtggctggttgagttctacgcgccctggtgcggccactgcaagaacttggagccccagtgggccaaagctgccaaagaacttaagggc
aaggtgaaactcggagcattagacgccacagtccaccaagcgatggcttcccgctaccaagtgcaaggctaccccaccatcaagctgttcccatctggc
aagaagtccagcgactctgcagaggactacaaaggaggcaggacagccagcgacatcgtgacttatgctcttgacaagctcgctgaaaatgtgcccgct
cctgagattgttcaggttatcgacgaagcgtcaatgcaggcgtgcagtgaaaaaccgctgtgcgtggtatcggttctgccgcacatcttggactgcaac
gcggcctgtcgcaacgaatacctggcgatactcgcacgacttggagacaagtacaagagcaagatgtggggatgggtgtgggccgaagctggcgcgcag
atatctttggaagaatcactggagctgggcggtttcggctaccccgccatggctgtcgtcaacgctaagaaactcaagttctcaaccctcagggatcc
ttctccgagactggcatcaatgaattccttagggatctatcattcggtcgcggccagactgccccagtgaagggcgcagagatgccgaagatcgtgtcc
accgaccccctgggacggcaaggacggtgaactgccacaagaagaggacattgacctctctgacgtagacctcgagaaggacgagttataa

>Hv_Contig_3271|gossypol-induced cytochrome P450 [Helicoverpa armigera]
atgatcctattacttctgttgaccgcgatattttgataatatccgcgatatacctcgtgtcgaagaagaagtttcaatattgggagaagaagaaagta
ccatatttacctccggttcctctcttaggaaactttgccaagtatatattgcagaagcaatttctcggagatactgtaaggaaaatatgtgacaaattc
ccgaatgcgccggtcatcggtgcttactacgccactgaaccagctctgatcgttcaagatcctgaactcatcaagctcgtcgtcaccaaagacttctac
ttcttcagtaaccgtgatgtatccgagttcgccaccagggaaaagttaacacagaacctcttctccacctccgggaaccagtggaaggtgctacgtcag
aatctgacgccagtatttacctctgcgaagatgaagaacatgtttcatttaatcgaaaagtgttctcacgtgtttgaagattttctcgacaaggaagcc
aaaacaagcaaggaagctgaaatgaggtctctcatagcaagatacactatggactgcatcggcacctgtgcatttggtgttgaaacaagcacaatgaac
aagacggaaaataatccgtttgtaaatgttggtaacagcatctttatctttgactcgtatcaatcatttaagttcttgctgaggactatctacccttca
ttattttatgcactgagatttacaacacttccaccagtagttgaggcattcttcactaatttaatgactggagtttttaaaggccgccaatatacgccc
acatctcgaaatgacttcgtcgattttgtcctaaaatggaagcataataaaccatttcaggggacagtctggcgaacttaaaatctaatacacagaca
aaagtgagtttagacgtcgatgatgacctgttagtggcgcaatgctttatatttttcgctgcgggatacgaaacttcggccaccacgttgagtttcact
ttgtatgagttggcgaaacacccagaagctcagaagagagctatagccgaggtggacgactatctgcggcgtcacaacaacgagctgaagtacgagtgc
ctttcggagatgccatatgtagaagcttgcattgatgaaactcttcgactgtatccagttctcagtgtgctaactcgcgaagtggcagaagattacact
ttcccttctggactgaaggtagagaaaggcctccgcatattcctgccactgtatcagttgcaccataacccggagtatttcccggatccggaggagtat
cggccggagcggttcctgcctgagaataagcaaaatataaagtcgtatacttacatgccctttggtgatggccccagaatctgtatcggcatgaggttc
gcaaaaatgcaaatgacagccggaataataactttgctgaagaagtaccgtttggaacttgctccagggatgaaaacgaagttgaacttcgaaccgaag
acggtgatcacgcaggctataggaggcatcaatctaaaaatgatagaaagggaaggatgggaggaaaggttattgaagaaattgtaa

>Hv_Contig_3294|chitin deacetylase 1 [Helicoverpa armigera]
atggcgcgctacgcccgtgtcgctactctggccgcgtgtctcttgttcgcctgcgcggttgccgatgggcaccgatggcggcggcaggctgacgatcgt
gaagcgccgaagaaagacgacagcttagaggtagagctatgcaaggacaaggacgccggcgaatggttccgcctggtggccggcgagggcgacaactgt
cgcgacgtcatccagtgcaccgcctcgggcattcaagctatccgttgtccagctggtttatacttcgatatcgagaagcagacctgcgactggaaagac
gctgtcaaaaactgtaaattgaaaaataaggaacgtaaagtaaagccgttgttgtacaccgaagagcctttgtgccaagatggattcctggcgtgcggt
gactccaactgcatcgagcgcggactcttctgtaacggcgagaaggactgcgcagacggctccgacgaaaactcttgcgatatc
gacaacgaccctaacagggctccgccttgcgatgcgtcgcagtgtgtgctgcctgactgcttctgctctgaggacggcacggtgatccccggcgacctg
cccgccaaggacgtgccccagatgatcaccatcacgtttgacgatgctatcaacaacaacaacatcgaactttacaaagagatcttcaacggaaaacgt
aaaaaccccaacggttgcgacattaaggctacatactttatctcgcacaagtacaccaactactcggctgttcaggaaactcacagaaagggtcatgaa
atcgctgtacattcaatcacgcacaacgatgacgaacgcttctggagcaacgccaccgttgacgactggggtaaggagatggctggtatgagagtcatc

atcgagaagttctctaacatcactgacaacagtgtcgtgggtgtgcgagcgccttatctccgtgttggtggtaacaaccaattcaccatgatggaagaa
caagctttcttgtacgacagcaccatcactgctcccctgtcaaacccgccgctatggccatacactatgtacttcagaatgcctcaccgttgtcacgga
aacctgcagagctgccccaccaggagccacgccgtatgggagatggtgatgaacgagcttgaccgtcgtgaggaccccaccaacgatgagtatttgcct
ggatgcgctatggttgactcttgttctaacatttgacaggagatcagttctacaacttcctgaaccacaactttgacaggcattatgaacaaaaccgt
gctccattgggtctctacttccacgctgcatggcttaagaacaaccccgaattcttagaggctttcttgtactggatcgacgaaattctccaaagtcac
aatgacgtatactttgtaacaatgactcaagtaattcaatggatccaaaaccctcgcactatcaccgaggctaagaacttcgagccctggagggagaag
tgctccgttgagggataccaggcctgctgggtaccccactcttgcaaactcacctccaaggaggttcccggtgaaaccatcaacttgcagacgtgcgtg
agatgccctgtcaactaccccctggctgaacgaccccacgggtgacggccattactag

>Hv_Contig_3618|glyceraldehyde-3-phosphate dehydrogenase [Helicoverpa armigera]
atgtccaaaatcggtatcaacggtttcggccgcattggccgtctggtcctccgtgccgccgtcgagaagggcgctcaggtcgttgccatcaacgacccc
ttcatcggcctggattacatggtctacctcttcaagtacgactccacccacggtcgcttcaagggctccgttgatatccaagatggccaccttgtcgtc
aatggaaacaaaatcgccgtcttctccgagagggaccccaaggccatcccatggggcaaggctggtgccgagtacgtcgttgaatccactggtgttttc
accaccacagagaaggcctctgctcatttagagggtggtgctaagaaggtcatcatctccgctcccagcgctgacgcacccatgttcgtcgttggtgtc
aaccttgaagcgtatgacccctcttacaaggtcatctccaacgcttcctgcacaaccaactgcctcgctcctctcgctaaggtcatccatgacaacttc
gagatcattgaaggtctgatgaccactgtacacgccaccactgccacccagaagacagtggatggaccctctggcaaactgtggcgtgatggccgtggt
gctcagcagaatatcattcccgcctctaccggtgctgccaaggccgtcggcaaggtcattcccgctctgaacggaaagctgactggtatggctttccgt
gtccccgtcccgaacgtgtctgtcgttgatctgaccgttcgccttggcaagcccgccagctacgatgccatcaagcaaaaggtcaaggaggccgctcag
ggtcctctgaagggcatcctcgactacaccgaggagcaggtcgtgtcatccgacttcatcggtgactctcactcatccatcttcgatgctgctgccggt
atctctctgaacgacaacttcgtcaagctcatcagctggtacgacaacgagttcggctactccaaccgtgtcatcgatctcatcaagtacatccagacc
aaggattaa

>Hv_Contig_3763|cathepsin L-like protease [Helicoverpa armigera]
atgaagagtatcgcggtactgctgtgcgtggtgggcgccgcgtgcgccgtctcgctgctggacctggtgcgcgaggagtggagcgccttcaagctggag
cacagcaagcgctacgacagcgaggtggaggacaagttccgcatgaagatctacctggagaacaagcaccgcatcgccaagcacaaccagcgcttcgag
cagggcgccgtcagctacaagctgcgcccgaacaagtacgccgacatgctcaaccacgagttcgtgcacgtcatgaacggcttcaacaagaccgccaag
caccctaagaacgtgcacggcaagggccgcgagtcccgcgccgccacgttcatcgcgccggcgcatgtctcctaccccgaccacgtggactggcgcaag
aagggcgcagtcaccgaggtcaaggaccagggcaagtgcggctcctgctgggccttcagtaccactggcgcgctggaaggccagcacttccgcaagact
gggtacctggtgtcgctgtccgagcagaacctgatcgactgctcggccgcgtacggtaacaacggctgcaacggcggcctcatggacaacgccttcaag
tacatcaaggacaacggcggcatcgacaccgagaaggcctacccctatgagggcgtcgatgacaagtgcaggtacaacgcgaagaactctggcgcggac
gacgtcggcttcgtggatattcctcagggtgacgaggagaagctgatgcaagccgtggctaccgtggggcccgtgtccgtcgccatcgacgcctcgcag
gagagcttccagttctactctgacggcgtgtactacgacgagaactgctcgtccaccgacctcgaccacggagtgatggtggtgggatacggcacggac
gagcagggcggcgactactggctggtgaagaactcgtggggccgcacgtggggagacctcggctacatcaagatggcgcgcaacaagaacaaccactgc
ggcatcgcctcctccgcctcctacccgctcgtgtag

>Hv_Contig_3816|cryptochrome [Helicoverpa armigera]

atgcttggtggaagtgtcctctggttcaggcacgggctgcgtctccatgacaacccgtctcttcactcggcgctggaagagaagggattccctttcttc
cctatcttcatcttcgatggagaaaccgctggaacaaaactggttggctacaaccgtatgcggtatctcctggaagccctcgatgacctggacagccag
ttcaagaagtttggaggcaggctgatcatgctgaagggcaagcctaatgtcgtgttccgaagactttgggaggaattcggcatccgtaaactgtgcttc
gagcaagactgcgagccagtatggcgcgctcgcgatgacagcgtgaagagtgcgtgcaaggagatcggcgtggtgtgccgggaacatgtctcccatact
ctgtgggagcccgagactgtcatcaaagctaacgggggattccgccgcttacttaccagatgtttttgcatactgtagcaactatcggcgatccyccg
cgccctgtcggcaacatcgacttcacgggagtcaagttcggcagcttgcccgagtgtttctatcaagagtttacggtctacgataagactcccaaacct
gaagacctaggcgtgttccttgagaacgaagacatcaggatgatccgctgggtgggtggtgagaccaccgcgcttaaacaaatgcaacaacgcctgtcc
gtcgagtacgagacattcctcaggggttcgtatttgccgactcatggcaaccctgacttattgggaccaccgatatcgttgagccctgcgctgcggttt
ggatgtctttcagtgagaagcttctactgggctgtgcaagatcttttc
cgtcaagtacatcagggccgtctcaccactaactctgcttctcatttcattacaggccagctgatatggcgtgaatatttctacacgatgagcgtgaac
aaccctaactatggccagatggctggcaaccccatctgcttggatatcccctggaagaatccagaaggcgatgagttgcagagatgggtagagggccgc
actggtttcccgttcgtggacgcggcgatgcgtcaactgcgcaccgaaggctggttacaccatgcggcccgtaacactgtggcctcgttccttactaga
ggcacgctctggctgtcgtgggaacacggccttaatcacttcttgaagtatctgctcgatgctgattggtcggtgtgcgccggcaactggatgtgggtg
tcgtcgtctgcgttcgaggcgctgctggactcgggcgagtgcgcgtgcccggtgcggctgggccagcgcctcgaccccagcggcgagtacgtgcgccgc
tacgtgcccgagctcgcgcgcatgcccgtcgactacatttacgagccatggaaggcccctatcgacgtacaagagcgcgctacttgcgtcatcggcaag
gactaccccgcgcccgtagtcaaccacttggtagccgcgcagaggaacaaaaacgctatgaaggaactccgtcatatgttacaaaaggctccccccac
tgctgcccttcctcagaagatgaaatccggcaattcatgtggctcaacgagtaa

>Hv_Contig_3928|beta-1,3-galactosyltransferase [Helicoverpa armigera]
atgaaaagaagacacttgaaatatttttgtgtgcgtgtgcgatagtgtggatatactatttttgggtgttagtgactatgtacgttcgaaaagcttt
gaacatgaattcgactaccctcttaatataaatattagaagcgtcgtggaagacgttttatcaggcaaaaaggttgcagtgacgcctatcaattattac
ccctacaggtttctgactaactcggggatatgttctaccacagacaagttagacctgttcatcgtggtgaagtctgcgatgagccattttagtcacaga
gacgccgtcaggcaaacatacggcaaagcgaatgtgccaggacgcactgtaaagtccttattcttcttaggaacagacggcaaacagaagtcagagcta
cagaaacaaatagacaaagaaatg
gccgagttcagggacataatacagatagactttatcgacaactactacaacaacactagaaagacgatgatgtcgttccgctgggtgtacgagcactgc
ccgacggctgactactacttgttcaccgacgacgacatgtacatctccgccaacaacctgctcgggtacttacacgaccgggaggcgacgcgcaagccg
gacacgtctgcggcgaacgacaagccctccactgcctctgcggagtccgacgactgcctctacgctggatacgtcttcgattcaattccacagaggttc
aggtcgagcaaatggagggtttctcttgaagaatacccgtgggataggtggcccgcctacgtcacagcgggcgcttacgtcgtatctaacaaagctatg
aaggtcatgtacgtagctagtctctttatcaaacatttccgattcgatgatgtttatttaggcatagtggcgaaaaaggccggtattataccgacgcat
tgtccggaattccatttctacaagaaggaatttagcgtggaaggttacaaggatgttatagcgtctcatggttacggagaccgtgacgaactgatcaga
gtatggtgcgagatgaatagtgggtag

>Hv_Contig_4027|eukaryotic initiation factor 5C [Helicoverpa armigera]
atgagtcagaaggtagaaaaaccagtattatcgggtcaacggatcaagaccagaaaaagagatgagaaagagaagtacgatccgaacggtttccgcgac
gcgctcgtgcagggtttggagcgcgccggcggcgacctggacgcggcctataagttcctagacgcggccggctccaagctcgactaccgaagatatggc
gaggtcatattcgacgtgctcatcgctgggggctgctgctccccggcggctcggtgtcgatggacggcgaatccccgaagaccaacacctgtatttc
accgcgaacgaggatatggagaccatgcgaaactttgagcaggtatttgtgaagctcatgagacgttacaagtacttggaaaagatgtttgaggaagaa

atgaaaaaggtgctggtgtacctaaaaggtttcgaacctctacagcgcatcaagctcgctcgtatgactgcactatggatcggcaacggctgcgtgcca
ccgtcggtgctgctggtgctggtgaacgagcacctgctgaaggataacctggcgctggagttcgtgctggaggtgttcgccaccgtcaaggccgagcgc
ggcgtcacctccctcgtcaccgcgctcaagcgcggacagcttgaaggcaggctgttggagttcctgccgctgaaccgtcgcagtgaggacgtgctggcc
tcctcgttcgcgtcgcgggggctcggcgagctgctgcgcctgcatcgcgcacaagcctcgcaggaggcgcgtcgcgagctgacgcaggcgctgctggac
gagctggcggaggagaagcccatccgcgacctcatccaggagctgcgcgacatggccgccaagcacgccatccccgaccacgaggtcgtcgccatcata
tggcaatgcgtgatgtcccgtggcgagtggaataagaaggaagagctgttagccgagcaagcggctaaacatttgcgccattacacgccgctgctggct
gccttcgcgcagtcggctaaggctgagatcgcgttactgactaaggtgcaagaatattgttacgagaacatgaacttcatgcgcgcgttcagcaagctg
gtcgtgatgctgtacaagaccaacgtgctttccgaggaggtgatcctgaagtggtaccgcgagcccaactccagcaagggcaagatgatgttcctcgac
cagatgaagaagttcgtcgagtggctacagagcgccgaagaagagtccgagagtggcgaagaagaagattag

>Hv_Contig_4247|NADPH cytochrome b5 reductase [Helicoverpa armigera]
atgagtaacatagaagtggctgtggatgattcgtttggaatcgcttcagttttacccatattcgttggcgttagtgccgctgtggtactagtgtccgtc
cttgccaactgtttgtggggcaaaaaagacaagaaggcaactcctaagaaragcacacaaccaataacattagtagaccctaatgtaaagtatgctcta
ccactcatagagagagaggagatcagtcatgatacaaggaggttccgatttggattgccatcatcggaacatgttttgggcttgcccatcggccagcac
attcacttatcggcgaagattgatgatgacctcgtcatcagatcttacacccctgtctcaagcgacgaagagaagggatatgtcgaacttgttattaag
gtatacttcaaaaatgtccaccctaaattccccgacggtggtaagatgtctcaacacttgaacaatctaaaaataaatgacacaatcgatgttcgagga
ccttcaggcagactgcagtacgcaggcaacggcctcttcctcatcaagaaaatgaggaaagacccacccgtagaattgcgggcgaaaaaacttaacatg
atcgcaggtggtactggcatcgcgccaatgctacagctcatcaggcagatatgcaaagatgccagtgaccccacagaaatgcgactgttgtttgccaac
cagactgaagaagacatcttgttgagaaacgaactggaaaagtaccaagctgaacatcccgaacagttcaaactgtggtacactctcgacaggcccaat
gaaggatggaaatacagcgtcggtttcatcaacgatgagatgatcaaagagcacttgttcccaccagcggacgatgttttagtactaatgtgtggacct
cctcccatgatcaacttcgcttgcaacccggcccttgaaaaactcggctatcccgaatcccaacgcttcgcctactaa

>Hv_Contig_4548|microsomal cytochrome P450 [Helicoverpa armigera]
atgatagtcgtactatggctcgtggtgctcatcgtagctctgacgctgtacctgcgccaggtctactccaggtttagcaactacggagtcaagcacttc
aagccagtcccactagtgggcaacctgggcacagtgcttatgcgtaaagaacatgcttctgcagacttcgacaaattatacaaggcttttcctgaagag
cggttcgtgggccgttatgatttcctgaggaacatcgtgatgattcgtgacctcgagctggtgaagaggatcaccgtcaaggactttgaacacttcctt
gatcaccgcatgttagctgatggtgacgttgagcctctattcggaagaaacctgttctccttaagagggcaagaatggaaggaaatgcgttccacgctg
agtccagcgttcactagctccaagatgagatcgatggtgcctttcatgatggaagtcagcgagcagatgataaactgcttaaaaacgaagatcaaagag
tctggagcaaactatgcagatgttgaatgcaaagacctgatgacccgatacgccaacgacgtaatagcatcctgtgccttcggcctgaaggtggactct
cacaatgacagagaaaatgaattctactccatcggcactgaaactgctaattttgacttcaaaaagatgttggtgatctttgggtattcttgctttcct
ggtattatgaggaaattcaacgtgaaaatgttttcggaactcatagcgacattcttcaggaatattgtaatcggtacaatgaggaaccgtcaaaagaac
aatatttaagacctgacatgatacatctactcatggaagccaagaaaggaaaactaacgcatgaagaaaaagcggctgaagccaatactggatttgca
acagtagaggaatctgacgttggaaaaattactgttaaaagagaatggacagacgatgacttgacagcccaagcggtactgttcttcgttgctggatac
gaaaccatatcatcagcgatggcgttcctcatctatgagctggcagttcaccctgaggtgcaggagaaactggcaaatgagatcaaggaacatgacgct
aagaacggcggcaagttcgacttcaattccatacagaacatgccttatttggatatggttatttcagaggtgctgagactatggcctccagcgatcggt
ttggacagagaatgcaccaaagattacaaccttggaaaacccaatgacaaagcagacaaggatttcatactccgtaaaggtgaggctgtggtgatcccg
acgtggtctgtccaccacgaccctcaatacttcccggacccttacaagtttgacccggagcgattctcggaggagaacaagcacaaaatcaagccattc

agttacatgccatttggacttggacctagaaattgtattggatcaagattcgctctctgcgaggtcaaggtcatgacataccagctgatccagcagatg
gagttgtctccctgcgagaagacttgcatccctgctgtactggctaaggacacatttaacttgaaggttgagggggaaattatgtcaggttcaagttg
cggcaatga

>Hv_Contig_4572|molting carboxypeptidase A [Helicoverpa armigera]
atggggtccttgatgaagacggtcgtgctcctgtgcgtcatatcctcggtgttatgtacgccgttccataaaaagctacagccrggacaagaatggcct
actcgtagttcagttaaacaaccgtatcatcaattcgatgagactgaaaatgggacaatatcagacacggttgctactgaagtcgcggcaaatgaagaa
aaaacacctgaaaataataaacctaagataaaagaggacgatgtagagagaatagactacagtggagcccaagtttggaaagtatcaacaaagaaatca
aacgcacaccgtgttattagtcaactcgcgtccgagaaactcataacatcatgggaggcaaccatacaagaatgtctattgatgtgaaaccgaaagct
ctagaaaacgtaaccgcaaccctaaaaaataaggaaattgcgtattacgttatgattgaagatctgcaacaaagaattgacgaggaaaacccacccttа
gatgaaaatgaactggaactgcaagacagacgaggtcatcgaatgacatggaagcagtatcatagactggaagatattcacggattttggactatttg
gcgaaaacttatccttcaatcgttagcgtcaatagtataggaaaatctttcgaaggccgcgacctcaaggtattacgcatttcggacggcaagaagacc
aacaaagcagtgtttattgacggaggcatccatgcgcgtgagtggatcagtcctgctgtcgttacatacttcattaaccaatttgcggaaaactttgat
gtggaatccgacgacattaggaatatcgactggtatttcttgccagtagtaaatcctgacggttacgaatatacgcacaagtccgaccgtctctggcgc
aagaacaggaaaggatatggtggatgtgccggcgcggatctcaacagaaactttggatatcagtggggaggcaaaggagcttcaaggtatccatgcagt
gaaatttaccgtggtagcgggccgttctctgaaccagaatctaaagcaattgctcaattctttaaaaatactgcagcagagttctcagcatacttgaca
taccatagttacggacagtacttgctctacccatggggctatgacaatgcattgccaccagaccacaagaacctagagactgtgggcaacctgatggca
caggctataaaaaagactggaggatcagaatacaaagtaggatcatcaagcgggctcctctatccagcagctggtggctccgatgactgggccaagtca
ttaaacatcaagtacacttacacaatcgaactaagcgacactggccgttatggttttgtgttgccagcgtcatacattgagccagtcgctagggagaac
ttggccggcttaagggttctagcaagccaattgaacaaagagtaa

>Hv_Contig_4643|beta-1,3-glucan recognition protein 1 [Helicoverpa armigera]
atggcagtttgtgttctgtttttaattttagtgtttagttttaattgtggcctgtgttatgaagtgccagcggctaaattggaagctatttaccctcga
ggattgaaggtctctattcctgatgacggcttctcactcttcgccttccatggcaatctgaacagagaaatgaacggcctcgaagctgggcagtggtcc
agagacatcaccaggccgaagaatggaaggtggaccttcagggacagggatgccgaacttaagatcggagatactgtctatttctggacgtacgtcatc
aaagatggtcttggatatcgacaggataatggagagtggaaggtcacaggttacgtcgacgatgctgaaagccctatcaacccaagcgaggttccctct
cttcccacttcttctgcctcccgaccctcaacctccaggccatctcttggagctggttctgctgcaccagcttcacaagtcaatgcggagtacccttgc
gagatttccgtatccaaggtcaatgtaccaggatttgtctgcaagggacagttgctcttcgaagataacttcaactctgggatagaaaagggcaagatt
tggactcctgagatcatgtttcctggagaaccggactttcctttcaacgtgtacctcaatgaccgcaatcttcatgtgagagatggacggctgctcatc
aagccgatcaccctggagtcgaagtacggagaggacttcgtgaggcagtcactggatatcacggctaggtgtactggtaccattggaactacggattgt
tcgagagtggcgtctggtcctcagatcctgccgcccatcatcacttccaagatcaccaccaagaacaaattcgcgttcaagtacggcaggattgagatc
agcgctagattgccgcttggggactggatttatccagagatccagctagaacctcgtgaccacgtctacggtgtcaggaactatgcatcaggactcatg
cgcatagccactgtcaaagggaacgtggaatctgccaagaaactgtacgcgggacccatcatgttcgattctgaaccttataggtctgtttatctgaag
gagaaggcaggttctgatctctggagcagggatttccacaattactcattggagtggagaccagatggcatatctctattcgtggatggtgagaaatat
agcgaagtgactcctcctgaggaaggtttctacaagactgcttctgagaaccaagtcgctgctgcttcacagtggcttaaaggaaccacgatagctccc
tttgatgaaatgttctacatatccctgggcctgaacgtgggaggagtgcacgagtttcctgacacggacagcaagccctggaagaaccgcgccaccaag

gccatgctgaacttctggaacgccagggaacagtggtactctacctggtacgatgacaccagcgccttgctcgtggattacgtcagggtatttgcttta
tag

>Hv_Contig_4654|seryl-tRNA synthetase [Helicoverpa armigera]
atggtgctcgacttagacttgttccgtgccgacaaagacggtaaccctgacaaaatccgtgaaaaccaacgaaaaaggttcaaagatgtggcccttgtt
gacaccgtggtggaacaggacaccttgtggaggagattgcgccacgaagctgacaattacaacaagctgaagaatgtgtgcagcaaggagattggtctt
aagatgaagagcaaggagcctgttggcccagaagaccagccggtaccagcagaaatagccgagaagttagttgacctgactggagatgacttgaagcct
ttaactgttaaccaaattaaaaaggtcagagtattaatagatgaagccataacaaagaatgaacagggtctcattgcggcagagaaggctcgttcagca
gctcttcgtgaagtgggcaaccacctccatgagtccgtccctgtggacgatgatgaggaccacaacaaagtggagaggacttacggagactgcactttt
aggaccaagtactcccatgtggatcttatttgtatgattgatggtatggacggcgaccgaggctcagccgtcgctggcggacgcgggtactacctcaag
ggcgcggcggtgttcctggagcaggcgctcatccagctgtccctcaggatactgctgaagaagggatacacgccactttatactccgttcttcatgagg
aaagaggtgatgcaagaagtagctcaactagcccaattcgacgaggaactatacaaggtggtgggcaagggatctgagaacaagggcgacgtggccatc
gaggagaaatatctcattgcgacctccgagcagccgatagccgcctaccacagggacgagtggctgcctgaggcttcattacccataaggtatgcaggt
ctctcaacg
tgtttccgtcaagaagtaggctcccacggacgtgatactcgcggtatcttcagagttcaccaatttgagaaggtagaacaattcgtcctgacctccccc
cacgacaacgcgtcctggcaaatgatggacgagatgatcacgaacgcagaggacttctacaaactgttgggcatcccttaccgcatcgtgaacatcgtg
tcaggggcgcttaaccatgcggccgctaagaaattggacttggaagcctggttcccgggctctggtgccttccgtgaactggtttcttgcagtaactgc
ttggagtaccaggctaggaggttgcttgtcagatacggtcaaacgaagaagatgaacgcagcgactgagtacgtgcacatgttgaacgcgaccatgtgc
gcgaccacccgcgtcatctgcgcggtgctcgaggtcaatcagactgaagagggcatcaaggtacctgaagccctaaaacaatggatgcctgagcagtac
caggaactgatcccattcgtgaagccagctcccatcgacgtggaagccgccgccgctaagaaaggcaagaataaagaaggaaagaaataa

>Hv_Contig_5387|voltage-dependent anion-selective channel [Helicoverpa armigera]
atggctcccccatactatgctgatcttggaaagaaggcgaacgatgtcttcagcaaaggctaccactttggcgtatttaaatttgacctgaagaccaag
agtgaaacaggtgttgaattcgccagtggaatcacctccaaccaagaaagtggaaaggtttttggtagcctttcatcaaatatgcagtgaaggactat
ggcctctcattcactgagaagtggaacacagacaacacacttgccactgacattacaatccaggacaagattgctgccggacttaaagtaaccctcgaa
ggaacatttgcgccacaaacgggtaccaagactggtaaattgaagacgtcttttgccaatgaaactgttgcggtaaacaccaacttagatttggacctg
gctggacccattgtggatatcgctgctgtcctctcctaccaggctggcttgccggtgtgcacggtcagtttgactcacagaagactaagttctccaag
aacaacttctctcttggttaccagtccaaggacttcaacctacacaccaacgttgacaacggaaaggacttcggtggttccatctaccagaaggtgtca
gaaaagatcgaatgcggtattagcatgaagtggaccgcgggctctgccgacacactgtttggcgtcggagccaagttcgcgctagacgccgacgcgtct
ctgcatgccaaggtcaacaacaaatctctcattggtctcggataccaacagaagttgcgcccaggtgtgattttgacactgtctgctgctatcgatggc
cagaacttcaacgctggtggacacaaagtgggtgtggctctggagctcgagccctaa

>Hv_Contig_5710|proteasome subunit beta 1 [Helicoverpa armigera]
atgctgagtgttagtggaaattttcctgaatatgctgttcctggcgccaaacaagttcgcttcgagccctacgctgacaacggaggaagcatcgttgct
attgccggtgatgatttttgcagtaattggcgctgatactcgtcttagtacgggattctcaatctacacaagggaacaaaagaaattgtttaagctgtcg
gacaaaactgtcctaggcgcgaccggctgctggtgcgacacgcttactctcaccagactgcttgctgcaaggatgcagatgtatgaacacgagcataac
aagtctatgtcaactcctgccgttgctcagatgctctccacgatgctctactacaaacgattcttcccttattatgtgtccaatgtgttagctggtctg

gatgctgacggaaaaggttgtgtctacagctacgacccaatcgggcactgcgaacgctccaactaccgcgcaggtggctcagcgggtgcacagctgcag
cctctgctggacaatcaaatagggctcaagaatatgcagaatgttacagaggcccccattcctaaagagaaggctttggccttactgaaggatgttttc
atcagtgctgcagagcgtgacatttatactggagactgcatctacatattgattattactgcaagcggtatccaggaggagaaatttgaattgcgtaaa
gattaa

>Hv_Contig_5788|receptor for activated protein kinase C [Helicoverpa armigera]
atgactgaaactttaaagcttagaggaaccctctgtggccacaatggctgggttacccaaattgcgaccaaccctaaataccctgacatgattttgtct
tcttctcgagacaaaaccctcatcgtatggaagctgaccagagacgagactaactacggtgtcccgcagaagcgtctgtacggtcactctcacttcatc
tcggacgttgtgctctccagcgacggcaactacgctctgtctggctcttgggacaagaccctgcgtctgtgggatctcgctgccggcaagaccaccagg
cgtttcgaagaccatactaaggatgtcctctccgtggcgttctcagttgacaaccgtcagatcgtgtctggctctcgggacaagaccatcaagctgtgg
aacacactggctgagtgcaagtacaccatccaggatgatggccacagtgactgggtgtcctgcgtccgcttctcccccaaccatgccaaccccatcatc
gtgtctgctggttgggaccgcactgttaaggtctggcaccttaccaactgcaagctcaagatcaaccaccttggtcactctggctacctgaacacagtc
actgtttctcctgacggttctctctgcgcttccggtggcaaggacatgaaggccatgctctgggatctgaatgatggcaagcatctgcacaccctggac
cacaatgacatcatcacatcattgtgcttctcacccaacagatactggctgtgcgctgccttcggaccttccatcaagatctgggatctggaaagcaag
gagatggttgaagagctcaggcctgagatcatcaaccagacccagacctccaagtcagacccaccccagtgcctgtctctggcgtggtccacagatggc
cagaccctcttcgctggctactccgacaacatcatcagagtctggcaggtgtcagtctcagcacgataa

>Hv_Contig_5821|juvenile hormone epoxide hydrolase [Helicoverpa armigera]
atggcgcgactcctattcatagcgcctatcctggccgtcatcctagttcctatatacttcgtattcctcaaaggaccccccgcccttgcctgacctggac
tacaacgagtggtggggaccagaggccctgaaagccaaacaggataccagcgtcagacctttcaaagttgcttttgatgatgtggccataaaagactta
aaagaccgtctcaaaagatcaaggtctttcactccgccgctagagggcgtgggctttgagtacggcttcaacagcggccagcttgactcctggctgaag
tactgggccaatgactaccagttcaaagaacgggagaagttctttaaccagtttcctcagttcaagactaatattcaaggccttgatatacatttcata
agggttacaccaaaggtgccagctggagtcgaagtcgttcctctactccttctccacggctggccaggttccgtcagggagttctacgaagctatcccc
ctcatcactgccgtcagtaaggacagagacttcgctatcgaagtcatcgtcccaagtctgcctggctacggattctcggacgcagctgtccgtccaggt
ctgggtgcccccccacgtcgccgtcgtcatgaggaacctgatgcaccgcctcggcttcaaacagttctacgtacagggggggtgactggggcagtctcatc
ggcagctgcatgactaccttcttccctaaggaagtcctaggctaccacacgaacttagctgcggttatgtctactaaagccaccttgatagagttgatc
gcatccgtctacccttcacttattgtggaacctgaacttgccgaccgaatgtatccaatgggtcagagatatgccactttggttgaagaaatggctac
atgcacatccaagccagcaagcctgatacagttggcgttgcactcacggattccccagctggtctcctggcgtatattctggagaaattctccacttgg
acgaggctcgagcaccgcttgaaggtggatggagccctaacgttccgcttcacaaaggaacagcttatagacaacttgatgatgtactgggcttcaagt
tctatcaccacctcgatgaggctgtacgctgagagttttaactccaaagtttatggaatgaagcttgacgagatccccacaccagtaccagtatgggtg
agccaagcgaagtatgagttggcgtaccagccgccttgcatcctcaagctcaagttccccaaccttcaaggagtaacagtcctggatgatggagggcat
ttcctggcgtttgaactgcccaagatattctccgaggatgttctgaaggccatgggggtattcagaaagttgtccaaaaataatgtgaagactgatttg
taa

>Hv_Contig_5932|fructosidase [Helicoverpa armigera]
atgttgtcaattgcaacaaagtttattctaatggctctgtttttaacagatgctgggccagtgatgtcaatcctcgctacgttccgcattaccatgtc
tatccaccatctggatggatgaacgatcccaacggcttctgtgtattcgatggcgaataccacttgttctatcagtacaacccttactccagccaagaa

gctggagtagcacattgggggcatgtgaagagccctgatctaatccattgggaacatctaccaaccgcaatgacaccagaccaaccgtacgacataaat
ggagtattctcaggaagtgcgatcatcgaaaatgggacaatgtatctcctttatacaggaaacgtgaacaatccaaccaacaagcaagtgcaagcttta
gctgcaagccaagatggaatatctgtggataaatatcctgggaatcctgtcatcgaaggagcagatcttcagccaaacataagagatccgaaagtctgg
aagcacagagatcttttctacatggtgctgggcaattctttcgatgataatactcgcggtcgagtactactttattcgtcgccagatttaatatcttgg
acacaggagtcagttctagatgagtctgacggctctctaggtaatgtgtgggaatgccctgatttcttcgaattggacggaaaatatgtactgctgttt
tctccacaaggtatgcaagctactggtgataaatacagaaatctattccaaactgggtacgtagttggaaatttcgattatgaaacgaaattgtttact
cctataaccgaattcagagaattggaccacggacatgattttatgctactcaaactattttggacaagtttggacgtagaattgttgtggcttggttt
gacatgtgggagcaagtttaccctgagcgcaacgatggttggactggccaaataactataccaagagagttttcgttgacagaagatttgagactcctg
caaaaacccgttaaagagctttctgctgcgcgtggcccacaattgtactcgggagaggctcaagctggttacaccctcgctttagaagccgcagccgct
gacatacgagtattggcacctagattgcaagattttgagctacttttagaatccgaaaattctacactttcaatcaaatatgactacaaaaaaggtaca
gtgacactcgatcgtggaggtgacgatggcgttcgtagaacaaagtggagacctgatggtaaacttgtctggcaagtcttggtagactcgagctccgtt
gaactattttgtggagaaggtgaagttactttctccagcagattcttccctaatggtggattgaaaatcagggtaggtgatggttctaatgctgatgat
ctaacagtgtacaaaatgattcgtacgattaaagcacccggtgacaactga

Bombyx mori sequences matching 50 open reading frames selected from *Heliothis virescens*

(Back to top)

>NM_001043376.1 [Annexin IX isoform A] ID:NP_001036841.1

ATGAGCGGACAACAATACTACCCTTACAAGTGCACCCCCACGGTGTACCCCGCGGAGCCGTTCGACCCGGCCGCGGATGCCGAGACCCTTCGCAAGGCT
ACGAAGGGCTTCGGCACCGATGAGAAGGCTATCATCGACGTGCTCTGCCGCCGCGGCATCGTGCAACGCCTCGAGATCGCGGAGACCTTCAAGACTAAC
TATGGCAAGGACTTGATTAGCGAACTCAAGAGTGAACTCACCGGCAACTTGGAAAATGTCATCGTCGCATTGATGACTCCCCTGCCCCACTTCTACGCT
AAGGAGCTCCACGATGCTGTCTCAGGAATTGGAACCGACGAAGAAGCCATCATCGAGATCCTGTGCACGCTTTCCAACTATGGTATCCGTACCATATCC
GCATTTTACGAACAACTGTACGGCAAGAGCCTGGAATCGGACTTAAAAGGCGACACGTCGGGACACTTCAAGAGATTGTGCGTGTCGTTGTGCATGGCC
AATCGCGATGAAAACCAGGGCATCGATGAAGGCTCAGCTAAAGCCGATGCTGAAGCACTGGCCGCCGCTGGTGAAGGTCAATGGGGAACCGACGAATCA
ATCTTCAACTCCATCCTTATCACTCGCTCCTATCAGCAGCTGAGACAGATCTTCGCCGAGTACGAAGCTTTGACCGGAAAAGACATTGAGGACTCGATC
AAGAAAGAATTCTCTGGTAGCATTGAGAAGGGCATGCTCGCTATTGCCAAATGCGTAAAGAGCAAGGTCGGTTTCTTCGCTGAGCGCCTGTACTACTCG
ATGAAGGGCATCGGCACCAACGACAAGACGCTCATCCGCATCGTGGTGAGCCGCTCCGAGATCGACCTAGGGGACATCAAGCAGGCATTCCTCGAGAAG
TACGGCAAGTCCCTGGAGACCTGGATCGCTGATGACACATCCGGAGATTACAAGAAGGCTTTGTTGACCCTTGTCGATAAATAA

>NM_001111364.1 [NADPH cytochrome P450 reductase] ID:NP_001104834.1

ATGTCAGAAAATGCACAAGACTTACTGAAAGATGCAGCTGCCGGTGCCGCCGCTGCAGCCGCTGCTGGTGGATCACTATTCAGCACATTTGACATAATT
GTGTTGGCATTGTTGTTGGGAGGTACTATATGGTGGTTTTACAATTCAAAAAAAGAAAGCAAGCGGGACAAAATACTTCTGGAAAAATACTCAATTCAA
GCAGCAGGATCTATACAAGTCACAGAAAACTCTTTTATAACAAAATTAAAGACTTCGGGAAGGAGTTTAGTAGTGTTCTATGGATCTCAGACGGGTACT
GCGGAAGAATTCGCTGGTCGACTTGCCAAAGAAGGCATGCGCTATAAAATGAAGGGTATGGTGGCTGATCCAGAAGAATGTGATATGGAAGAACTCACA
AAGCTACAAGAAATTGAAAACTCCCTGGCTGTTTTCTGTATGGCAACATACGGTGAGGGAGACCCCACAGATAATTCAATGGAATTTTATGAATGGTTA
AAAAATCAAGATCCAGATCTGACTGGCCTAAATTACGCAGTATTTGGGCTTGGTAATAAAACATATGAGCATTATAATGCAGTGGCCATTTATTTAGAT
AAAAGATTAGAAGAACTTGGAGCTACACGAGTATATGAATTGGGAATGGGTGATGATGATGCTAATATTGAAGATGACTTCATTACGTGGAAAGATAAA
TTTTGGCCAGCGGTATGTGAGAAATACAACATTGAAAGTGCAGGAGAAGAAGAACTCACGAGACAGTTCCGTCACGTGCTCCATCCACCTGATGAAGTT
TCTCCTAACAGTGTGTTCACTGGTGAAATTGCTAAGCTACACTCGCTACAAGTTCAAAGACCACCATATGATTCTAAAAATCCATTCTTGGCACAGATT
ACTGTCAACAGAGAACTACATCGTGGTGGTGACAGATCATGTTTGCATGTAGAATTAGACATATCTGATTCTAAAATGCGATATGAAGCTGGTGATCAT
GTAGCTGTGTATCCTATCAACGATATGGATTTAGTAGAGCGCTTGGGACAACTGACAAATGCCAATCTCGATGAAATATTTTCGCTTATCAACACTGAC
CAGGAAAGCAGCAAGAAACATCCATTCCCTTGTCCAACATCTTACAGAACTGCTCTCTCGCATTATGTAGAAATTACAGCATTACCGCGTACCCACATC
CTCCGAGAGTTGGTTGAATATTGTTCTAATGAAGAAGATAAGAAGAAGCTGCTTCTGATGGCCACAAACACTCAAGAGGGAAAATCCCTGTATCAGTCG
TTTGTAGTTGATGCTTGTAGAAATATTGTGCACATATTGGAAGACCTACCGTCATGTAAACCTCCCTTGGATCATATTTGTGAACTTTTGCCCCGGTTA
CAACCAAGATACTATTCGATTTCTTCTAGTCCTAAGCTTCATCCAGAGACGGTGCACATTACTGCGGTCGTCGTAAAGTACAAGACTCCGACCGGACGA
ATCAACAAAGGTGTCGCTACGACGTGGCTTGCTCAACATAAACCCGAGCCGGGGAAACCACTGCCTCGTGTCCCGGTTTATATAAGGAAATCTCAGTTC

AGGCTTCCAATGCAGACCCAAACACCAATAATAATGGTGGGACCGGGGACTGGATTGGCGCCCTTCCGAGGTTTTCTTCAAGACCGCGCATTCGTCCGC
GCCAGTGGGAAAGAAGTGGGCGAGAACATCCTTTACTTTGGATGCAGACATCGTGATGAGGATTACATCTACCAAGAGGAACTAGAGGAATACGAAAAG
AATGGTGACGTAAAACTGAACTTAGCATTTTCACGCGACCAAGCGAACAAAGTTTACGTTACACATTTACTCGAAAATAATATGGACGAACTATGGGAT
GTTATCGGAAATAGGAATGGAAATTTCTACATTTGCGGTGACGCGAAAAACATGGCTGTGGACGTAAGGAACATTGTGTTGAAAGCTATAAAAGAGAAA
GGCGGCCGCACAGAGACGGAGGCCGTGCAGTTCCTCAAGAAGTTAGAATCCGTCAAGAAATATTCTTCGGACGTTTGGAGTTAA

>NM_001099828.1 [3-hydroxy-3-methylglutaryl-CoA reductase precursor] ID:NP_001093298.1

ATGAAAGTGTGGGGAGCCCACGGGGAATTCTGCGCTAGACATCAATGGGAAGTCATCGTAGCCACCCTGGCTTTACTAGCATGTGCAGCTAGTGTCGAG
CGACATGGCCCAGGGAACAGATCTGAACACTGTGCGGGTTGGGCTCGAGCGTGTCCCGGCCTTGAAGCGGAGTATCAAGCAGCCGACGCGGTCATCATG
ACTTTTGTCCGCTGTGCCGCCCTGCTATATGCATACTACCAAGTTCTCAACCTTCACAAAATAGCCTCGAAATACCTCCTCATCATAGCCGGGGTGTTC
TCAACGTTTGCCAGTTTTATATTCACGTCGGCACTCGCTAGCCTGTTCTGGAGCGAATTGGCTAGTATAAAGGATGCGCCGTTTCTCTTTCTGTTGGTG
GCTGATGTCGCCAGAGGTGCTAGGATGGCGAAAGCGGGGTGGTCGGCCGGTGAAGACCAAGGGAAGGGGGTTGGGAGGGCCTTGTCGTTGTTAGGACCA
ACTGCAACTCTGGACACGCTACTCGCCATATTATTGGTCGGCGTCGGAGCTTTATCTGGTGTTCCCCGTCTCGAGCATATGTGTACTTTTGCTTGCCTA
GCCTTGTTAGTTGACTACATTGTGTTCATAACTTTCTACCCCGCTTGTTTGTCATTGGTAGCAGATTTCGCAACGAACAGAAAAGAAATAGCCCACGAC
AGTCCATTCTCGGAGGAGGACTTGAAACCCAACCCTGTGGTGCAGAGAGTCAAAATGATTATGGCCGCTGGCTTATTGTGCGTTCATTTGACGAGCAGG
TGGCCTTGGTCCGCTAATCACGGCATAATTGAGGGACCAATTGATGCATCCATACCTGTTCCCCACGACAATATACTCCTCCATTCATACGTGAAGTGG
TTCTCTGTGAGCGCAGACTACATCGTTATAGCGACGCTGCTCTGCGCTTTGATTATAAAATTCATTTTCTTTGAAGAGCAACGTAACTGGGTTTATGAT
ATGGACGACATGACTGTAAAGGAAGTGATAAATGACACCGATTTGAGCCGTAAACCCAAGTTTTCTGTCGGTGACGATTCCAATTCTGAAGTGTCTACA
CAGACCGATGAGGCTGGAAATGTCGAGGACATGGAGTGGCCCACTTTGTCCCCAAGCTCCTCGGCCTCTAAATTGAACGCCAAAAAGAGGCCTATGGTC
GAATGCTTAGAGCTATATCGATCTGGTGCTTGCACTTCTCTCAGCGATGAAGAAGTCATAATGCTAGTCGAGCAATCCCATATACCCATGCACAGATTG
GAAGCTGTGCTGGAGGATCCCTTGCGCGGTGTGAGACTGCGCCGGCGAGTGATCGCGTCGAGATTTAACAACGAAACCGCAATAAAACAACTTCCCTAC
TTAAACTACGATTATAGTAAAGTCTTAAACGCGTGCTGTGAGAATGTGATTGGCTACATTGGAGTTCCCGTCGGATATGCCGGCCCGTTGGTGGTAGAC
GGGAAGCCTTACATGATCCCGATGGCTACGACCGAGGGGGCTTTGGTGGCGTCAACAAACCGCGGAGCCAAAGCCATTGGCAGTAGGGGAGTGACGAGT
GTCGTCGAAGATGTCGGCATGACAAGAGCGCCGGCTGTCAAATTGCCAAACGTAGTGCGAGCCCATGAATGCCGACAATGGATCGACAATAAAGAGAAC
TACGCTCTGCTCAAAGAGGCGTTCGATTCGACTTCAAGATTCGCCCGGCTCCAAGAAATACACGTCGGAGTTGACGGAGCTACTCTCTATCTAAGGTTC
AGAGCGACAACTGGCGACGCTATGGGAATGAACATGGTCTCGAAAGGCGCGGAAAACGCTTTAAAACTATTAAAAACCTTCTTCCGTGATATGGAAGTT
ATTAGTTTGTCAGGGAATTATTGTTCGGACAAAAAGGCAGCGGCGATAAACTGGATCAAAGGTAGAGGTAAAAGGGTTGTCTGCGAGACTGTCATATCC
AGTGAAAATTTGCGCACGATCTTCAAGACCGACGCTAAAACTCTGTCTCGGTGCAACAAAATCAAGAATTTATCCGGTTCCGCTTTAGCCGGGTCCATA
GGCGGCAACAACGCGCACGCGGCTAACATGGTTACCGCTATCTTCATAGCTACCGGCCAAGATCCGGCTCAGAACGTCACTAGCAGCAACTGCTCGACA
AATATGGAAGCTTACGGGGAAAACGGCGAAGATCTATACGTTACCTGCACCATGCCCTCCTTGGAAGTCGGCACAGTAGGTGGGGGAACAGTTTTGACG
GGTCAGGGAGCGTGTCTAGAAATCTTAGGAGTCAAAGGAGCGGGTACGCGACCTGCCGAGAACTCCGCCAGGCTGGCGTCCCTAATATGCGCTACGGTT

TTAGCTGGCGAGCTTAGTCTAATGGCGGCCTTGGTTAATTCGGATCTGGTGAAATCGCACATGCGTCACAACAGATCGACGCTTAACGTTCAAACTGCC
AATGTCGAACCGTACACGGTGGCACTCAAAGTACCTCCATCATAA

>JQ061144.1 [aminopeptidase N-2] ID:AFK85018.1

ATGCATCATCTGACAAAATTCTGCCTTCTGGCCGCTTTGGCTCTTGTCAGAACTGATTTCCCACTGGACACAGACGAACCAGTCAATCGAAGTAATGTT
GATGAAACTATTTATCGAGTGCCCGAAGATTTAGATCCCATACATTTTGACGTGGAGATCACTCCTTATTTTGAAGCAACACAGACTGATGAAGCATTT
ACCTTTGATGGTATTGCCACGATTCGGCTTAGGGCTGTAAAAGACAATCTGAATGCACTCATCATTCAAGAGAACGTTCGACACATCGTCGCAGTCAGT
CTGACCAATACTGATGGAGTTCCGATTCGCTTGAATACGAGGAACCCGTTTGAACGCATAAGAGCTTACCACTTTTTGAAGATTAATCTTGCTGAAGGC
GTTACTTTAGTCAACGGTCAAATTTACATATTGACTATCGAATATATTGGTAACATCAACGAAACCCCACTTTCTCGAGGAGTATTCAGAGGGAATTAC
ATCGGTGATGATGGAAAATTGCATTGGTACGCGGCAACACACTTACAGCCAACAAACGCCAGACAAGCATTCCCGAGCTTCGACGAGCCCGGCTTTAAG
TCTACTTTCAATATCATCGTCAACAGACCTGCTCATTTCACCGAGACTTATTCCAACATGCCAATCAGGCAGACCATACCAATTGGCGACCGCGTTAAA
GAAATCTTTCACACTACTCCCAGAATGTCAGCCTATCTAGTTACATTCCATATAAGTGAAGAATTCACCGTTATTGCTGACAATAATGATCCTGTGAGG
TCGTATAGAATTCTTGCAAGACCGAACGCCAAAGGTCAAGGTGAATATGCCTTGGAAGTCGGGCCCCCGCTAACCAAGTGGTTAGAAGAATATTTGAAT
ATCGATTATTATAGTATGCAATCTTTTATGAAAAACGATCAGATTGCTTCGCCGTTTTGGGCTTCTGGAGCTACTGAGAATTGGGGCTTGGTTACTTAC
AGAGAATTTCGTCTCCTTTACGAACAAGGCGAAACAAATGCACTAGACAAAATGTACATCGGAACTATCACCGCTCATGAACTCGCTCACAAGTGGTTC
GGAAACTTGATCACGGCCAGGTGGTGGGATAACGTGTGGATTAACGAAGGTTTTGCCAGCTACTTTGAATACTTCGCTATGGATGGTGTTGATCCCGAA
TTAGAACTAGCTGATCAATTCAACTTGATGTACATGCAAAGCGCATTATCCACCGACGCTTCTGCATCCACCAGAGCTTTACGTCATACCGTGAACAGT
CCAGCTCAAGTCACTGGTCATTTTTCTGGAATCAGTTATTCTAAAGGAGCTTCCTTCCTGTTAATGATGAAGCACTTGGTTACAGAAGATACATTTAAG
AAGGCTTTGAATTATTTCCTCATAGATAGGTCATTTGAGTACGCCTCGCCGGAAAATTTATATTCTGCTTTTGTGAGAGCAGTACGCGAAGATAATACA
CTGGAAACTACTTTCGACGTTGAAGAATTCATGAAGTACTGGGTTGACGAGCCTGGATTTCCTCTTCTGGATGTTGCAGTCAATACAGAAACCGGAGTT
ATCAGCCTTAAGCAGGAGCGCTTCTTCATAAGTACCAGTGCAACACAAACTAATCAAATCTGGCCAATTCCGTTGACCTACACAACTGGTAGCAACCCT
AACTGGAACTCCCTGCGACCTTTGCACATAATGACCGCTCAGAATGATGAAATACGTATAACACCTGGAAACCAATGGGTCATCTTTAATGTACAACAA
AAAGGCATCTACAGAGTGAACTATAATCAAGAAAACTGGGAACGTCTCGCTAATGCTTTAAGCGAAGATCATACCAATATACATCATTTGAACAGAGCA
CAGATCGTCGATGATGTATTCGCGCTGATGAGATCAGAGAAGCTAAGTTTCGATCTTGGTTTTCGTGTTTTGGACTTCCTCAAGAAAGACACCAGTTAC
TATGTTTGGTATCCGGCCGTCACTGGATTCGGTTGGCTAAGGAACCGATTTCTGCATATGCCTGATGTGTTGGCCGAATTTAATACAATTCTCTACACC
TTCCTAGAAGCAGTCATTGCAGATCTTGGTTACGATGTAGTTGACGGCGAACCCCTCACAAGGACCTTGAATAGATTTTTCGTTTTGTCATTTGCGTGT
AATATTGGGCATGATGGTTGCATTTCTAATGCTATTCAGAAGTTCAATGCTTTGAGGACTAGTGGTACCAGCGTCAATCCTAACCTGAGACGCCATGTT
TTCTGCTCTGGTCTCCTTGAAGGTGGTTACAATGAGTGGCGATTCCTATATGAGCGCAGAAAGAACTCGAATAACCAAGGTGACGAGGTCGCTATGCTC
AGATCTCTTGGGTGCACCACGAACCCCCAGGCTCGACAGGAATACTTAAGCATGATTTTGAGTGACGATGTCAAAGCTCAAGATAGAGTGAATGCTCTC
ACATTCTTCTATATGGGAGACCGTTCCAACGCTAATGTAGCCCTGCAGTACTTGAAGGAGAACTTCGAGGAAATCAGACAAGGAGTCGTTCTCCCCGCT
TGGTTCGATAACGTTATTTCTAATTTGGCTTCCTATTTGAACGAAGAAGGACTCGAAGATATGGAATCTTGGCTTCGGGCCAACCAAAACATGATACCC
AACTTCAACGTTGGACTAAACGCTATAAACTCGGCAAGAACAAGCATGCAATGGGGAACTGACAGAGCACAGGAGATTCTGAAAGCAGCCAGAGGCTCT
GCTGTTACCGTATTGCCAACATTCATGCTGTTGGTCCCAACATTAGCCATGCTCGTTTTGAAGTAG

>NM_001102465.1[nuclear factor NF-kappa-B p110 subunit isoform 1] ID:NP_001095935.1

ATGTCTACAACTGCCAGTGATCAGGATAGTGATTCTTCCAGCAGATCCCCACAATCTTATTATAGTTATTGTTCACCCTCTCAACAAGTACCTCAGTTG
ACTCCAGATTTGAACAACCTAATATATCTAGACAATGGCTCAAAGAGAAACAGACCGTTTCTTCGCATAATTGAACAGCCACAGGACTACTTTAGATTT
CGCTATGCTAGTGAAATGGCTGGAACACATGGATGTTTGCTCGGAAAGTCATCTTCAACAAACAAGAACAAAGTTCACCCTACAGTTGAGCTTGTAAAC
TACACTGGCCAGGCTGTAATAAAATGTCAGTTGGCCCAACATAAGACACCTGAGGAGCATCCACACAAGCTATTCGAAGAAGAACAAGATAATGATGAC
AGAGAAGTCAGCTGCATTGTTCCTAAACAAGGCATTTACAAAGTCGGGTTCGGTGGAATGGGTATCATACACACAGCAAAAAAAAACGTACCAGGCTTA
CTGTTCCAGAAGTATGCAGAGAAGTGTAAGAACATCAGCAACAAAGAGCTCAAAATCCAATGCGAGAATATGGCTAAAAGCATAGATCTCAACATTGTG
CGTTTGAAGTTCAGCGCTCACGACATCAGCACCGATCGTGAGATATGCAAGCCTGTTTTCTCTGAACCGATACACAGCTTAAAAAGTGCTGCTTCGAAC
GATCTGAAGATATGTCGCATCAGTCGTTGTTCCGGGCGTCCAGTTGGAGGAGAAGACGTGTACATTTTCGTCGAGAAAGTTAACAAAAAAAATATACAG
ATACGCTTCTTCGAATTGGACGAGAATGGCCACCGCGTGTGGACGGCTAACGGGAGTTTCATGCCTGGCGATGTTCACCACCAGTACGCCATAATCTTC
AGGACCCCGGCCTACAAGGATACGAAGATATCAAAGAACGTGAACGTGTATATAGAACTGGCGCGGCCCTCGGACGGTCGCACGAGCGAACCTAAGGAG
TTCAAGTACATCGCCGAGCCCATATACTCGAACAGCAAGAAGAGAAGGATGAATGCCTCATGTTCTACTTCCCCTGCTTCGTCTGGGTCGTTGAAGAGT
ATCAGCGATCTGCCCGCCCTTGTGACTCTCAATAACAATTTCAACGATGAAAACAACATTGAAATGCCGAAAACTGAGCTGCCGGCCGCCGCGGCCGCC
GCCCCCTCTCTGGGACTGTGCGACCTCGCCGACGCGCTCATGGGCTCCGAGAGCGCCGTGGACCTACACTCGGTCGCCGGGAACGCGATGTGGAGCCCG
AGTGCGCCCGTCGAGACGATGAGCGTCGAGCAGTTCCCGAATCTGCAACTGAATTCGACGGAGTTCGAAAAGATAATAACGAACGCCCTTCCGCCGGAG
GAAAGGAACGACTTCACGGAATATGCCCTAAGTTCGTACGAATCGTTCGATGACATCGACGATGATCCAAATGGATGGAAGTTCATACGTACGTTACAG
ATGATGCAAACCGACTCTGCACGCAGTAGGCAGCAGACACCGCAGACTAGTAAACCCGCACTGAAAACTGTTGAACCCCAAGAGAGCTCAGACAGGACC
GAGCTGAATGTACCGTCTGATGCGAAAGATGCAAACGAATATAGCGCTTATTACAACGCCCAAGACGGCCTCGAAGTGAAACAGCTACTAAGAGAACTG
ACGCAGATAATACGCAACAAGAAGGTTCATAAGAAACAAGAACTGAAAGACAAACTAAAGAGACTTTTCGACATAAGACTTTCGAACGGCGACACGTAC
CTCCACATGATACTGTGCAGCAATCAATCCAGCATAGCGAACATGGTGACGATAATTGACAGCGTTAAAATGACCCACCTCTTGGACTACAGGAATTTG
AAACTACAGACGTTACTGCATACCGCCATAGCTAATGACTTGTCCCATTACGTGACATTACTAGTATCAAAAGGAAGCAGCGTGAGTTTGCAAGATGTA
GCCGGCGACACCGCGTTCCACTACGCGGCCAAAAGTCACAAGTGCTTGGAGCCGCTACTAGCCGCAGTCAAGAAGCATAACATACCGTTCGACTTGAAC
ACTTATAATTACGAAAAACAAGCGCCTCTGCACTTGGCGAGGACGGGACAGAACGCGCGCCATCTACTGGAGAGCGGCGCCGACCACCGCGTGCGTGAC
ATGCACGGCCGCACGCCGCTCCACCTGGCCGCCTACGACAGTCGCCTCGCGCTTGTCGCCGCCCTGCTGGACTACATACCCTCGAAAGAAATAGATGCA
ACCGATGAATCAGGCAACACAGCACTACAGATTGTTTGTGGCGGGCCCTTAAGACAGAACTCCGTGGAGATCGTCAAACTATTACTTGCGAAAAAGGCC
GATCCTAACTTTCACGGCGCTCAGAAAGAATCTGCCTGGAAAATGTCTGCCGATAAACCTGAACTAAGAGCCCTTCTCTTGGAAGCTATCGCCTTCGAA
GACGGCTTCGAGAATAATAGTGTGAAATCTGAACTCGATGACGAAATCAAGTCTGAACCTGATGACGAGATCAAGTCTGAACCCGAGGACGATTACGAT
TCAGCTGATGAAGGCGAGGAAACAAGTGGCAGCACGGCACTAGACGGCATGGGTCAGTACTTGGGCGAGGTGAGCTCGCTGCTGGACCGGTCGGGCAGC
TGGCAGGAGCTAGCGGGCAGGCTGCGTCTCGACTCGCTGCTCTCCTTGTACTGCGCGCAGCCCAGCCCCACGCACACGCTGCTGCTGCATCTCAAGGTA
TACCTTGTTTACTGTATGGCCTTGCAGCAACCCAGCTTATAA

>NM_001043369.1 [aminopeptidase N precursor] ID:NP_001036834.1

ATGTATTTACTATTCATCACCGCCCTTTTGGGCTCAGCTTACTCGTTCCCAACGAGCACTTTCAACAACGTCACAAGGAATACGGATCTGGCCTCCCAG
TATGTCCTACCCGGAGAATCATTCCCCACGTTCTACGATGTCAGTTTATTCATTGATCCTGCCAATACCGTCAGTTTCAACGGGAAGGTCAGCATCAGA
ATTATCCCCAGGATTGCCACAAACGTTATCGTTGTCCAAGCCATGGAGATGACCATTAGAAGCATTAGTGTTTTTACTGACAGGAACAGCAACGAAAAC
TTGTTTACTAGTTTCACTCTAGCAACTGATGATACGCATTTTTTGAGAATCAGTACCAGAACACAGCTCCTTCCAGACCAGCCGTATATTGTAAACATT
GATTATGAATCTAAATACGCGCCCAACATGTTCGGAGTATATGTATCTACATACCAACAAAATGGACGAACAGTAAATCTGGTAACTTCTCAACTTCAA
CCAACGTTTGCCCGCCGTGCATTCCCTTGCTACGATGAGCCGGCTATCAAAGCTATTTTCAGAACTACCATATACGCACCAGCAGCCTACACGGTTGTT
AGGCACAATACTCCCGAAAGAGCTGTCCCATTGAAGGAGGACGTGGCGGGTTACGTGAAGCACGAGTTTGAAGACACCCTCGTAATGTCGACGTACCTG
CTCGCCTATCTCGTGTCGAACTTCGAGCACGTCAGCCACGAGCAAAACCCCATCTACAGAGTCCCCTTCAGGGTATATTCTAGGCCAGGAACCCAGACC
AACGCAGCCTTCGCAATGGACTTCGGACAAAAGAACATGGTCGCTTTGGAAGCGTACAACGAATTCCCTTACGCTTTCCCTAAATTAGATAAAGCAGCC
GTTCCCGATTTTGCTGCGGGTGCCATGGAGAACTGGGGTCTTGTCATTTACAGGGAAGTGGCGTTGCTGGTGACGGAAGGTGTAACGACCACAGCGACC
AGGCAGAACATTGCCAGGATTATCTGCCACGAGAATGTGCACATGTGGTACGGCAATGAAGTGGGTCCGCTGTCCTGGACTTACACTTGGCTCAACGAA
GGTTTCGCAACGTTCTTCGAGAGTTTCGCCACTGATTTGGTACTTCCAGAATGGCGTATGATGGAGCAGTTTGTGGTGACCATGCAGAATGTGTTCCAA
TCGGATGCTGTTCTCACTATCAACCCCATGACGCACGCGGTCTACACTCCTTCTCAGATCATGGGACAATTTAACGCCATTGCCTATCAAAAATCCGGC
TCGGTGATCAGAATGTTGCATCATTTCCTGACACCGGAAATATTCAGACGAGGTCTCGTTATTTACATCATTAACAACTCCCGTCGTGCTGCCGGGCCG
TCAGATCTCTACTGCGCTCTCCAACAAGCCCTGGATGCGTCCGACCACAGCATCCCGTACTCGATCTCCAATGTGATGAACAGATGGGTCAACCAGGGA
GGGTTCCCAGTGTTGAACGTGAGAAAGAGCGCCCCGAACGCTAATTCGGTCTTTATTTCTCAGGAGCGATATCTCACAGACCGCTCTCTGACGTCAACG
GACCGTTGGCATGTGCCAGTCAACTGGGTGCTGTCTTCCAACATTGATTTCTCTGACACAAAACCCCAAGGATGGATCCCACCGTCGTTCCCTGCCACC
TCGATCGACATTCCTGGATTGGCAAACGCCGAATGGTTCATTTTTAACAAGCAACAGACTGGCTACTACCGCGTGAACTACGATCCGGAGAACTGGGCG
GCCCTCGCAAAGATCTTACAGACCAACCATGCTGTTATCCATCTCCTCAATCGCGCTCAGATTCTTGACGATTCCTTCAACATGGCCAGAAATGGGCGC
CTGAACTACAACCTTCCCTTCGAGATTTCTCGATACCTGATCAACGAGAAAGATTACATTCCCTGGGCTGCCATTAACCCTGCCTTCAATTACTTAGAT
ATCGTTCTAACCGGATCTTCAGTATACAATTTGTTTAGGGAGTATCTTCTGACACTGACTGCACCTCTATATGACGAAATTGGTTGGGAGGCGACTGCC
AACGAGGAGCATGTTATGGCTTACCACAGAAATATCATATTGGACATCAATTGTCGTCTCGGAAACCAGAGATGCGTCACCAGAGCCCAGGAGCTGTTG
GAGCAGTTCCGAAATAATCCAACTCAAAGACTGAACCCGGATTTACAAAACACAGTGTACTGTTCCGGTCTACGTGGCGGTGATAGAGATAATTTCAAT
TTCCTTTGGGAGCAATACTTGGCTACCTCTGATTCAAGCGGGCAAAACATCCTCCGCAATGCTCTGGGTTGTTCTTCGAACCCTGAACTGCGTCCCTTC
TACATGAACCAAGTTTTCGATGCCCACTCCCCAGTGGGGGACAAGACCGGCACACAATTCTCGTCTCTGTCATCAACTCTAGTCCAGAGAACATGGAC
GCTGCCTTAGAATTCGTCATTGAAAACTTCCACAGGATCCAACCGAGGGTGCAAGGCCTCACCGGGACCACTAACATATTAAATGCCTTCGCAAGAAGA
CTGACCACAGAAACGCATGCTGAAAGGATAAACCAGCTGATCAGTCGTCACCAAGCCATCTTAACAGCTGGAGAACAAGCATCCATCTCAGCTATCAGA
GAGCACATCGCAGCTTCGATAGCTTGGGGTAAAGACAATGCTGCTGTTGTAGAGGACTGGCTTGAAGACAACTACGGAGAACCCAAGCCGGAGGAACCA
TCCGCAGCACATTCTACGACGGCTGGTTTTATTGTTTTGCTATCTGCTTTTGTCGCTTTCTTTAACATTCATTAA

>AY155274.1 [inhibitor of apoptosis protein] ID:AAN46650.1

ATGGAGTTGACGAAAGTTGCTAAAAATGGAGCTGCCGCCACGTTGGTGATGTTAAAAAATGCGCGGGATGCAAAAATGCGACCTTTCATTGGTCCGCTC
ATGTTATCCTCGTGCGAGTCTTCAACGACATCCACACTCCCGTCACCTTCGTCGTCAGCTGATAAAACGGATAATCACGACACATTCAACTTCCTTCCT
GATATGCCCGACATGCGTCGTGAAGAGGAACGTCTGAAAACATTTGATCAGTGGCCCGTTACGTTTTTGACGCCGGAACAATTGGCCCGCAACGGATTC
TACTACCTCGGTCGCGGCGACGAAGTGTGCTGTGCTTTCTGTAAGGTAGAAATTATGAGGTGGGTCGAAGGCGACGATCCTGCCGCCGATCATCGGAGA
TGGGCGCCCCAGTGTCCCTTTGTACGAAAACAAATGTATGCCAACGCTGGGGGAGAGGCGGCCGCTGTCGGTAGAGACGAATGTGGGGCCAGTGCGGCC
ACGCAGCCTTCCCGCATGCCCGGCCCCGTGCACGCGCGGTACTCCACCGAGGCCGCGCGGCTCGCCACCTTCAAGGACTGGCCGAGATGTATGCGCCAA
AAACCCGAGGAACTGGCAGAGGCCGGATTCTTCTATACAGGCCAAGGTGACAAAACGAAATGCTTCTATTGCGACGGAGGGCTAAAAGATTGGGAAAGC
GATGACGTTCCGTGGGAACAGCACGCCAGATGGTTCGACCGCTGCGCGTACGTGCAATTGGTGAAAGGACGTGACTACATTCAGAAGGTGAAGTCGGAG
GCCACTGCGATATCTGCTAGCGAAGAAGAACAGGCCGCCACCAATGATTCGACTAAGAACGTCGCTCAAGAGGGCGAGAAACATTTGGATGACTCTAAA
ATATGTAAAATATGTTATTCCGAGGAGCGTAACGTGTGCTTCGTGCCGTGCGGCCACGTGGTGGCGTGCGCCAAGTGCGCGCTGTCGACGGACAAGTGC
CCGATGTGTCGCAGGACGTTCACGAATGCGGTGCGGCTCTACTTCTCGTGA

>NM_001043431.1 [embryonic polarity protein dorsal isoform B] ID:NP_001036896.1

ATGGACATCGGCGGAGACGCCGGTCTACCGATAGGTATTGCAGAACAAAATGACCCCAATCAACCTAACGAATTGAATATCAATGATGTTTTTGAGGCC
ATATCGCTCGCGGATCCCACGTTCGGCGAGGGCCCTCCTCCTGTTGCCTTAGAGAACATGGCGAGTCAATCCGCGCCCCCGCGAGCACCAAGCGTGGTA
ATAGTCGAGCAACCAGCCAGCAAAGCCCTCAGATTCCGGTACAAATGCGAGGGCCGGTCGGCGGGCTCGCTCCCGGGCGTGTCCAGTACTCCGGAGAAC
AAAACTTATCCTACAATCAAGATATGCTCTTACAAGGGACCGGCCACCATAGTCGTGTCCTGCGTCAACAAGGATCCCCCGTTTAAACCGCACCCGCAC
AACCTGGTGGGGCGGGAGCGCTGCGACCGCGGCGTGTGCTCGGTGCGCACCGACGTCACGGAGGACAACAACGAGTACCAGTTCAGGAACCTCGGGATA
CAATGCGTCAAGCGCAAGGACATCGTCGCCGAGCTGCGCCTCCGGGAGGAGCTCAGGGTGGACCCCTTCAGGACGGGGTTCGACCACCGCTCGCACACG
CAGAGCATCGACCTCAACGCGGTGCGGCTCGCCTTCCAAGTGTTCGTGCCGGACGGCGCCGGGCGCATGCGCCGGCCGCTGCCCACCGTCGTGTCCGAC
ATCATCTACGACAAGAAGGCCATGAGCGACCTCCTCATCATGCGCTCCTCGCACTGCTCCGGCACCATCCGCGGCGGCACGCAGGTCATCCTGCTCTGC
GAGAAGGTATCGAAAGAAGACACGGCGGTGATATTCTACCAGGAGGTGAACGAGCAGATAGTGTGGGAGGAGACCGTGACGCCCTCCTACGTGCACAAG
CAGGTCGCCGTGGTGTTCAACACGCCGCCCTACCGCGACCCCCAGCGCGACGAGCACGTCACCGTACACTTCCAGCTGAAGAGGCTGACGGACGACGCG
CGGAGCAACTCCTTCGCGTTCGAATACATCCCGGACTTCCGAGATTCAAATTACATATTGGGTAAAAAGCGGAAGACCAAAATGCCATTAATGGCCGCG
TATGAGCAAGAAAGGACCTTCCAGGAGCAGATGCCGATCAAGGCGGAGCCCAGAGAGAAGTCGCCGCACCACGCCACGTCGCCCTACGGCGCGTACCCG
CCCTACAGCGACCCGAATTGGATGGTTCCCCCGGAGCAGATGCGAACGATACCGGACCTCGCCGTACCGGCCGCTACCAACATGGACACCTTCGCCGAC
ATGGCGTGGAGCAACGTGCCGTATGGACAGGGCCTCCAGCCGCTGCAGCCCGGTGCCAACGTCATGCACTCCCCCCACTCCCTGTCCCCCATGCACAGG
GGGCAATCCGTGTCCCCCATGCAGGACTCCCAGTGCGGCTCCCCCATGCACGGCGGCCCCTGCGTATCCCCCATGCACGGCGGCGCCTGCGTGTCCCCC
ATGGTCGGCCCGATGATGCAAGTCGGGTCTACATCGCCCAACATGCAACACAACACTTATAGTCACCTGCCCCAGGGCGCGGAGGCGGACGGCGCCGGG
ACCACGTTCTCCGCCCAGTTCCTTGACCGCGAGCCCATACTCAACTCCAGCGAGTTCCGATCCCTCCTCGGGGGGGACGGGAACATGCTCTCTGCCGAC
CTCAACAAGCTCTCCACAAGCGACCTCCTGCTGTGA

>NM_001112755.1 [cytochrome P450 Cyp6b29] ID:NP_001106226.1

ATGGCAATTATCTATATTTTGTTAGCCTCCGTAGTTTTACCGCTTCTCCTCTATCTTTATTTCACAAGACATTTCAACTATTGGAAGAAACGAAATGTC
CCCGGGCCCAAACCCGTACCGCTTTTCGGGAACTTAATGGAATCGGCACTGCGGAAGAAGAACATTGGTATCGTGTTCAAAGAATTATATGAAAACTTT
CCAAATGAGAAGGTCGTTGGAATTTACCGCATGACAACACCCTGTTTGTTGATACGGACCAGGATGTCATCAAAAATATAATGATTAAAGATTTCGAT
GTGTTTGTCGATCGAGGAGTTGAACTAAGCAAAAGTGGTTTGGGCGCTAATCTTTTTCATGCAGATGGGGACACTTGGCGTGTACTTCGTAATCGATTC
ACGCCACTCTTCACTTCAGGAAAGTTAAAGAATATGCTTCATCTTATGATTGAGCGCGCCAACAAGTATATAGAACACGTCGAAATGTTATGCGACCAC
CAACCGGAGCAGGATATTCATACGCTTGTCCAGAAATACACGATGGCCACCATTGCGGCTTGTGCTTTTGGTTTAGATATCGACACGACGGATCCAAAC
AAAGATCAACTGAAAACGTCAGAAGAAATCGACAGGCTGTCACTAACTGCAAACTTTGCTTTTGAATTGGACATGATGTATCCGGGTGTTTTGAAAAAG
TTGAACAGTACATTGTTTCCGGGATTTGTGTCTAGGTTCTTCAAAGATGTAGTCAAAACTATTATCGAGCAAAGGAACGGTAAGCCCACAGACTGGAAT
GACTTTATGGATCTCATTTTGGCTCTGAGACAATTGGGTGATATCCAAGCAACAAAGAGAAACTCGGAAGACAAGGAATACAGTATTGAATTGACGGAT
GAATTAATAGAGGCTCAAGCCTTTGTGTTCTATATAGCAGGATATGAAACCAGTGCCACTACAATGACCTTCATGCTGTATCAGTTAGCTTTAAATCCT
GACATTCAAGACAAAGTTATAGCTGAAATTGATCAAGGTCTAAAAGAATCCAAAGGGGAAGTCACCTACGAGATGCTTCAGAATTTAACGTATTTCGAA
AAAGCATCTAATGAGACCTTGCGTATGTACTCGATAGTGGAACCTCTGCAAAGAAATGCAAAGATAGATTGTAAGATACCTGATACGGACATAGTTATT
GAGAAAGGAACAACAGTTTTATTCTCGCCCTTAGGGATACATCACGACGAGAAGTATTATCCCAATCCAAGTAAATTTGACCCTGAGAGATTTTCTCCA
GCTAATATCAGCGCGAGGCATCCTTGTGCTCACATACCGTTCGGCACTGGTCCACGTAATTGCATTGGGATGCGGTTTGCGAAAATTCAAAGCAGAGTG
TGTATGGTGAAAATGTTCTCAAAATTTCGTTTTGAATTAGCAAAGAATACGCCTAGAAATTTGGATATCGATCCTACAAGGCTACTTCTCGGCCCAAAG
GGTGGCATTCCTTTGAAAATTGTAAGGCGATGA

>NM_001184983.1 [flavin-dependent monooxygenase FMO2 precursor] ID:NP_001171912.1

ATGTATTCACGCGTATTCATTTTCGTTCTGTGTATTCAAATGTGTTTATTCTACAAAATACGGGAAAAGAAACGGGTTTGTGTTATCGGAGCCGGCATA
GCTGGTCTCTCATCGGCAAGATATTTAAAGGAAGAAGGAATCGACTTCGTTGTATTCGAAGCCACGAAGTATATTGGTGGTACGTGGCGTTACGATCCA
AGAGTTGGTACCGATGAAAATGGTCTACCATTGCACACCAGCATGTACAAGCATTTACACACGAATTTACCAAAACCAACGATGGAATTAAGAGGTTTC
CCATTACCAGATGGAATACCATCGTTTCCTAGTTGGAAAATTTATTATGATTACTTGAAAGATTACGCTAAGCATTTTGACATAGAAAAATATATACAG
TTCCGACACAATGTTACTTTGGTGAGACGAGAACAAAACGTTTGGAAAGTGACACACGAACACGTCATCACTGGGGAGGTATTTGAAGAAAATTATGAC
TACGTCATAGTCGGTAACGGGCATTTTAGTACACCGAACATGCCTAACATACGGGGAGAGAAACTGTTCAAAGGAACAATAATCCACAGTCACGACTAC
CGTGTACCGGACGTTTACAAAGATCGCAGGGTGCTTGTTGTCGGGGCGGGTCCATCAGGAATGGACATAGGCCTTGACGTGGCTGAATGCAGTAAGAGC
CTCCTACACAGCCACCATTCTAAAGTGAACTTCAGAACACCGTTCCCACCGCATTACGTGAGGAAGCCTGACGTAAAGGAATTCAATGAGACTGGAGTT
ATTTTTGTGGATGGAACTTATGAGGAAATCGATGACGTCATCTACTGCACTGGTTTTCAATACGACTATCCATTCCTAGACAAGACCTGTGGTCTAGAT
ATAGACCCGCACAGCGTGGTCCCCCTGTACAAATACATGGTGAACATCCGTCAGCCGAGCATGGTCATTTTGGGCTTAGTTGTTCGCGCTTGTCTCGTC
GTAGCTCTAGACGCCCAGGCACGGTATGCAACGGCCTTAATAAAAGGAAACTTCACTTTGCCGAGCGAAGCAGAAATGATGGACGAATGGCAGAGACGG
GCTGATGCGATCCGATCTAAAGGACTAAGAATGTCTCACATACACACCCTTGCAGAAAAAGAAGACGAATATTACGCTGAACTATCGGAGCAATCTGGA
ATAGAAAGAGTTCCACCGGTCATGTTCAAGATACGCGCCATGGACATTGAAGCTAAGCTTGAAAACCTGTACACGTACAGGCACTACGTGTACAAAGTC
ATTGACGACAACACGTTCGTTAGGACCCTGGAAAAAGAACAGATCAACGATACTTTGGTGATTTAG

>AB497079.1 [carboxyl/cholinesterase 5BL] ID:BAI66485.1

ATGATATCGGCCGTGAACGAATTCCTAGATGATCTTCGTGGTGGCAGGATGTCCGAGTCGCCTCTAGTCACGGTGGAGCAAGGGCAGCTCCAGGGTCGG
ATCGTAAACAGCCCGTCCGGGAAGGCCTTCTACAGTTTCCAAGGAATACCATATGCGAAACCCCCATTGGGGTCTTTGCGATTTAAGGCCCCACAACCT
CCAGAACCCTGGGACGGCATCAGGGATGCCACGGCGGAAGGGAACGTCTGTGCTCAAATCGATCCAGTCTTCGCGAAATCCTATGTCGGCGATGAAAAC
TGCTTGTTCCTAAACGTGTACACCCCTAGCACTGATGGAGCATTCCTCCCGGTCATGATTTGGATCCACGGTGGTGGGTTCAAGTGGGGATCCGGCAAT
ACCAACTTGTATGGACCAGATTTTCTAGTTGACAGAGATGTCGTGGTGGTGACAATCAATTATAGATGTGGTGCTTTAGGGTTCTTAAGTCTGAATACG
CCTGAAGTCCCAGGGAATGCTGGTATCAAAGATATAGTTCAAGCCATTAGATGGGTGAAAGATAATATCCATCACTTCGGTGGTAATGCCGGTAATTTG
ACAATATTTGGTGAAAGCGCGGGCGCGCGTGCGGTTTCGTTGCTGACAGCTAGTCCGTTAACCAAAAATTTAATCAGCAAAGCTATAATCCAATCTGGA
AACGCATTGTCTTCTAGAGCTTTTCAAAGAGACCCCTTACAAAGTGCTAAAGCTTTAGCCAGGAGTTTGGGCTGTGAAGCAGAAGACGTTGATGAAATT
CTAGAGTTTTTAATCGCGACTCCAGCCAAAGACTTGGTTGAGGCAGACGAGAAGTTGAATTCTTTGCAGAAAGTTCTAGAAACGAGTAATAATTTGTTT
GGGCTGGTAATCGAGAAGGAGTTTCCAGGAGTCGAAGCTGTTATCAGTGAGCCTTTCATTAACATACTAACATCAGGTAGAACAGCCAACATCCCAATA
CTAGTTGGAACTACGTCATTAGAGTACGCCTGCGAGAGAAAGTCTGATGATCTTCAAGAGTTGATACCAGCTGACCTGAACATAGACAGAAACTCAGAA
GAAGCCTTGGCAATCGTAGAAGAAATAAAGAAATTGTATTTCAAAGGGAACCACACAGGAGTGGAGAGTCTACCAGAATACTTCCATCTCTTATCAGAC
AAGTTGATCAATTTGGACACGCATCGATACATAAAATATTTGCTCCAAGTTACGAACCGTCCAATTTATTACTATAGATTTGATTATGTGGGCGAATTG
AATATCGCCCAGAAGATCTTCTTTAGTCTTGGACTGAAGTACGCTATGCACATGGACGAGTTGGGCTATCTGTTCAAGAATGACTTCCAGAAAGACGTG
GAGCCGACTCCTGAAGATATCAAGATGCGTGAGAGGATTGTCAGGCTCTGGACTAATTTCGCTAAATCTGGGAACCCGATACCGGACGAAAACCACTAT
TTGAACACCAACTGGCTACCGGTAACAAACGACAATTTATACTGTCTAAATCTAAATTCAGAATTAACTTTAATCTCAAACCCGGACAAAGAGAAAATG
GACTTTTGGGAGAAAACCTACGACAAACACTACAGGATCTGGGATGAAACGACCAACCAGTCTAAAAACGAACCACAAATCGATACATTCAAAAAAGAA
CCAGAAATCGATACATATCAAAATGAACCAGAAATCGATACATGCAAAAAAGAAACAGAAATCGATACATACAAAAATGAACCAGAAATCGATACGCCC
AAACATGAACCGGAAATAGTGTCCGTTATCGAGACAGTGGTCATAACCCAAGTCATGAACAAAGAGACCAGCGTTTCCGAAGAGAGGACAGAAGTCGTG
AGTAACTCTGACCAAGTCGACAGAGCCGAAGTTGAAACGCAAATCACAACTACCACACTCCTTGAAAACAACGATGGCTCCCAAACTTTGATAGTCACA
GAGAAAACTGAGACCCCCGAACAAGCACCGAGCGTCAAAGAAATGATACTGGAGTTCAGTGAACCGGCCCAACTAGAGTCGAATGAACACAAACCGGTC
AATGACCACATAAACACTAACGGAGTCGACAAGAAAGCCAGGACTTCGAACGAAATCAAAATGGTGCATAACAGCAACGGTGCTCCAAAGGACGTGATC
AGAGCTAACGATCCGCCCGAAGACGATCTACCCAAGAACATAGGAGTTAATAAATTCGTCAGTTTCTTCGAATCGCTAGGCGGAAAGAAGTAA

>NM_001043547.1 [hormone receptor 3] ID:NP_001037012.1

ATGTTGAACATGTTTGATATGTGGAACTCTGTGAGCAAGCTGGAGGCGCAGTCCAATGTGCAGCAAAGCCAACAGCCACACACTTCAGGTGGGAGCATT
AAAGCCCAAATCGAGATAATACCGTGCAAGGTATGCGGAGATAAATCGTCGGGGGTGCACTATGGCGTGATCACCTGCGAGGGATGCAAAGGATTCTTC
AGACGATCCCAGAGCACAGTGGTGAACTACCAGTGTCCTCGCAACAAGGCCTGCGTCGTGGACAGGGTCAACCGCAACCGATGCCAGTACTGCAGACTA
CAGAAGTGCCTCAAACTCGGCATGAGTCGTGATGCCGTGAAATTCGGTCGCATGTCGAAGAAGCAGCGAGAGAAGGTCGAGGACGAGGTCAGATACCAC
AAGGCGCAGATGCGGGTGCAGGCTGATGCGGCGCCGGACTCCGTGTACGACGCCCAGCAGCAGACGCCCAGCTCGAGCGACCAGTTCCACGGGCATTAT
AACAGCTACCCAGGATACGGGTCGCCGTTGTCTTCGTATGGCTACAACAACGCCGGGCCAGCGCTACCCTCGAACATGAGCGGGATGCAGCCGCAGCCC

CCAGCCCAGCCCCCGTACGAGGTCTCAGGCGACTACGTGGACTCCACAACGACATACGAGCCCAAACAGACAGGGTTCTTGGACGCAGACTTCATAAGT
CACGTGGAGGGTGACATTAGCAAGGTGCTAGTGAAAAGTTTGACAGAGGCGCACGCGAATACAAATCCGAAGCTGGATTACATACATGAGATGTTCGGC
AAGCCCCAGGATGTTTCTAAGCTCTTGTTCTATAACTCCATGACCTACGAGGAGATGTGGTTGGACTGCGCCGACAAGCTCACCGCGATGATCCAGAAC
ATCATTGAGTTCGCGAAACTCATACCTGGTTTCATGAAGCTCACCCAGGACGATCAAATACTGCTGCTTAAATCAGGTTCGTTCGAGTTGGCGATCGTC
CGCCTGTCGCGGCTAATCGACGTGAACCGCGACCAGGTGCTCTACGGAGACGTGGTGCTACCCGTGCGGGAGTGCGTGCACGCGCGCGATCCCAGAGAC
GTAGCTCTGGTGCAAGGAATCTTTGAGGCTGCCAAGAGCATCGCTCGACTGAAGCTGACCGAGACTGAACTGGCTCTATACCAGAGCCTTGTGCTCCTG
TGGCCAGAGCGTCACGGCGTGATGGGCAACTCGGAGATCAGATGTCTCTTCAACATGTCCATGTCGGCGATGCGGCATGAGATCGAGGTCAACCACGCG
CCGCTCAAGGGTGACGTCACCGTGCTGGATACACTCCTGGCCAAGATACCCACTTTCAGAGATCTCTCCCTGATGCACCTCGGAGCGCTGAGCCGTTTC
AAAGCGACGCATCCGCATCACGTTTTCCCAGCTTTATACAAAGAATTGTTCTCTTTAGACAGTGTTTTAGATTACACGCACGGATAA

>AB060275.1 [90-kDa heat shock protein] ID:BAB41209.1

ATGCCGGAAGAAATGGAGACACAGCCAGCGGAGGTTGAAACCTTCGCGTTCCAGGCTGAGATCGCTCAGCTTATGTCCCTGATCATCAACACCTTTTAC
TCCAACAAAGAAATTTTCCTTCGTGAGCTGATTTCCAATTCATCGGACGCTTTAGACAAAATCAGGTATGAATCTCTCACGGATCCGTCAAAACTCGAT
AGTGGCAAAGAGCTGTACATCAAGATCATTCCCAACAAGAACGAGGGCACTCTTACGATCATCGATACCGGTATTGGTATGACCAAGGCCGATTTGGTG
AACAATTTGGGAACCATCGCGAAATCTGGTACTAAAGCTTTCATGGAGGCTCTTCAAGCAGGTGCCGACATCAGCATGATTGGACAGTTCGGTGTTGGC
TTCTACTCCAGTTACTTGGTCGCTGACCGCGTGACTGTTCACTCTAAACACAATGACGACGAGCAATACGTGTGGGAATCTTCTGCAGGAGGCTCGTTC
ACAGTCCGCCCAGACAGCGGTGAGCCCCTTGGTCGAGGTACAAAGATCGTCCTTCACGTCAAAGAGGACTTGGCAGAATTCATGGAAGAACACAAAATC
AAAGAGATCGTAAAGAAACATTCCCAGTTCATTGGCTACCCAATCAAGCTGATGGTTGAAAAAGAACGCGAAAAAGAACTGTCTGATGATGAAGCCGAA
GAAGAAAAGAAGGAAGAGGAAGATGAGAAGCCTAAGATTGAAGATGTAGGTGAAGATGAAGACGAGGACAAAAAGGATACGAAGAAGAAGAAGAAAACA
ATTAAAGAAAAATACACAGAGGATGAAGAACTCAACAAGACAAAGCCTATCTGGACCAGAAACGCTGATGACATTACCCAAGATGAGTACGGAGACTTC
TACAAATCCCTTACCAATGATTGGGAGGACCATCTTGCAGTCAAACACTTCTCCGTTGAAGGTCAGTTAGAGTTCCGAGCACTACTTTTTGTGCCACGT
CGTGCACCTTTTGACTTGTTTGAAAACAAGAAGCGCAAGAACAATATCAAGCTGTATGTACGAAGAGTTTTCATCATGGACAACTGTGAGGATCTCATC
CCAGAGTACCTTAACTTCATTAGAGGTGTTGTCGACAGTGAGGACCTTCCTCTAAACATTTCACGTGAGATGCTTCAACAGAATAAAATTCTGAAAGTA
ATTAGGAAGAATTTAGTTAAAAAATGCCTAGAACTATTTGAAGAATTGGCAGAGGACAAAGAAAACTACAAGAAGTATTATGAACAATTCAGCAAGAAC
TTAAAATTGGGTATCCATGAAGACTCTCAAAACAGGGCTAAGCTCTCTGAGCTTCTGCGGTACCACACTTCAGCATCGGGTGATGAAGCCTGCTCTCTC
AAAGAGTATGTATCCAGGATGAAGGAGAACCAGAAACATATCTACTACATCACTGGAGAAAACAGAGACCAGGTGGCGAACTCCTCATTTGTGGAGAGA
GTGAAGAAACGTGGCTATGAAGTAGTCTACATGACTGAGCCCATTGATGAATATGTAGTACAACAGATGAGGGAGTATGATGGCAAGACCCTTGTATCG
GTCACAAAGGAAGGATTAGAACTTCCTGAAGATGAGGAAGAAAAGAAGAAACGTGAGGAAGATAAGGTGAAGTTCGAAGGCCTGTGCAAGGTGATGAAG
AACATCCTGGACAACAAAGTTGAGAAAGTTGTTGTCTCTAACAGACTTGTGGAGTCTCCTTGCTGTATTGTTACTGCTCAATATGGATGGTCTGCCAAC
ATGGAACGTATCATGAAGGCTCAGGCTCTACGTGACACATCCACAATGGGATACATGGCTGCGAAGAAGCATTTGGAAATCAATCCTGATCACTCAATT
GTGGAAACATTAAGGCAGAAAGCGGAGGCCGACAAGAATGACAAAGCTGTTAAGGATCTTGTTATCTTATTGTATGAAACTGCCCTGCTGTCTTCTGGC
TTCACGCTTGATGAGCCCCAAGTCCATGCTTCCCGTATCTATAGAATGATCAAACTGGGTCTTGGTATTGATGAAGATGAGCCGATTCAGGTTGAAGAG
CCTGCTTCTGGAGACGTGCCGCCATTGGAGGGTGATGCTGATGATGCATCTCGCATGGAGGAAGTTGATTAA

>XM_004931806.1 [acidic juvenile hormone- suppressible protein 1-like] ID:XP_004931863.1

ATGGAGAGGACTATGCTCGGAATTTTCCTGCGAGATCAAATCAGAAATGAGAAGATTCGTAGGAAAACCATGTTAACCGACATAGCCCAAAGGATTGCG
ATATTGAAGTGGCAGTGTGCAGGATACATTGATCAAAGTGCGGTTAACGAGTGGTCAAATTTACTAAAAAACAACGGGTTAATCTCTCGTCATCTACCA
TTTACGGTCCATGAAGAGAAACACAAACACGAAGCCATCATTTTGTTTAGGATACTTTACTTTACCAAAGACTACGACACATTCTACAAAGCAGCCCTT
TACGTGAGAGATCGTGTTAATGAAGATCTCTTCCTCTATGTGCTATCCACGACTGTTATTCACCGTCGAGATTTAGCTGGATTCATTATTCCTCCTATT
TACGAGATGTCGCCCGAGTACTTTAATAATGGACAAGTGATGACCTTAGCACAGAAAATTAATATCTTGGGAGATAGTATACTTGATGACTGTAAAGCT
GTTTTCAAATTTGACAATAGTATAGTTATAAGGGAAAATGATACTGTTTGGCCTTATTACATGTATGATCAGCCTTTACATTACTACAATCATGACGAG
GGCTTGAATTTATTCTATTATTACTATAATTTGCTGTATCCCTCGTGGCTCGGTGGAGACCTTGCTTCCCTTGAATTTATTTTCTGTTATCTTGAGAGA
TTGTCTAACGGCCTTTCTGAAATAGAAGACCTTGATCATCACATAGTGAAGGAGGGATATAAGTCTAGCATGATATACAACAACGGTATACCCTTCCCA
AGCCGTCCAGATAACTTCCATCTGGACCAGTTCGATTTTGCAGATGAACTTCAAAAGATCGTGGATTATGAGCGACGTGTACGTGATGCCATAGAAAAT
GGTTTCATTTTTAACGAATCTGGTGAAAAAATTAATTTAAGAATTCCCGAGGCTGTCGATATAATCGGACGTATCATTGAAGCTGGTGTTGATTCTCCA
AATGGTCGCTATTACAAAGACTTTATCACACTATGGAAGTCCATTCTTGGAAATTCATTGGCGCATAAGAACGATTATAGTGAAAGCTATGTACCTCTT
GTCATCCCTTCAGTCCTGGAGCATGATCAAACAGCACTCCGCGATCCTGCATTTTATATGATCTGGAAACGGGTCCTTAATTTGTTTAAGCTTTGGCAT
GAACAACTTCCTCCTTACACACGTGAAGAATTAGCCTTGTCTTCAGTGGTAATTGAAAATGTCGAAGTTGACAAATTTGTCACATATTTCGAAAATACC
TACACGAATATTAGTGCAGGTCTTTACAGATCAAATAAATGCAAAACAAGCATTGTCCCGGATGAAGTGAATATTCTTGTTCAGCATCCAAGGCTGAAT
CACAAGAAGTTTAATGTGCGAGTTCGTGCGAAGAATTCACTAGCCCAAAACGTTACAGTCAGATTCTTTTTGGGCCCAAAATATGATAGCAGAAAATTT
AATATACCACTCCACGAAAACGTTGAAAACTTTTTTTTGTTGGACGAATTTATCTACGACCTGCCTGAAGGCGAAAACGTCATCGTTCGTAAGTCGACT
GATAGTCCTCATTACACCTATAATTTGAGGGCTGCAAGCGATGTTTACAGAGAAGCTGTTGCGGCTTTAGACGGGACAGGAGATTACCATTTTGATCCT
ACGCAAATTATCAAATTTCCACACAATCTTGTGCTACCTAAAGGTCACTTAGCCGGTATGCCGTATGTGCTACTGGTTCACGTAACGGAGTACAAGCCA
CCCAGACTACCATTCGGAAGTACTTTCGATTCTGAGTTGTCCAGCGGACCAGGTTCCGGCTCTCGTCGTCTGGCCGGAGACCCACTGGGCTATCCTCTT
AATAGGCCACTGTATTCATGGCAGATTGAGAGCCTGCATAATTTTTATTTCCAAGACGTATTGATTTATCACAAGCCAACTCCGGAAATCAATGTTCCT
TACTCTTCTGAAGTTTAA

>NM_001109926.1 [c-Jun NH2-terminal kinase] ID:NP_001103396.1

ATGCCCCACGCGGCTCCCGTCTCCGCCATGTCGGCCCCGCCGCGCCACCCACACTTTTATACCGTTGAGGTCGGCGACACACGCTTCACCATCCTCAAG
CGCTACCAGAACCTCAAACCCATCGGCTCCGGCGCCCAGGGTATTGTATGTGCCGCATACGACACGGTGACGCAACAGAATGTTGCCATAAAGAAGTTA
TCGCGTCCGTTTCAGAACGTGACGCACGCGAAAAGAGCTTACAGGGAGTTCAAACTTATGAAACTTGTCAACCATAAAAATATAATCGGTCTGCTAAAC
GCGTTCACGCCGCAGAAAAGCCTCGAGGAGTTCCAAGACGTTTACCTGGTTATGGAGCTGATGGACGCTAATCTGTGCCAAGTGATTCAGATGGATCTG
GATCACGAACGGATGAGCTATCTGCTGTACCAGATGCTGTGCGGCATCAAACACTTGCACCTCGCCGGAATCATTCATCGGGATCTGAAGCCGTCCAAC
ATAGTGGTGAAGAGCGACTGCACGCTGAAGATCCTGGACTTCGGGCTGGCTCGCACCGCCGGGACCACCTTCATGATGACGCCCTACGTCGTCACGCGC
TACTACCGCGCGCCGGAGGTGATTTTGGGTATGGGCTACACCGAGAACGTGGACATCTGGTCCGTGGGCTGCATCATGGGGGAGATGATCCGCGGCGGC

GTGCTGTTCCCCGGCACCGACCACATCGACCAGTGGAACAAGATCATCGAGCAACTGGGCACGCCGTCGGCCGCGTTCATGTCCCGGCTGCAGCCCACG
GTCCGCAACTACGTCGAGAACAGACCGCGCTACTCCGGGTACAGCTTCGAGCGGCTCTTCCCGGACATACTGTTCCCCAGCGACAGCAACGAACACAAC
CGACTCAAGGCGTCGCAGGCCCGCGACCTGCTGTCGCGCATGCTGGTCATCGACCCCGAGCGCCGCATCTCAGTGGACGACGCCCTGCTGCACCCCTAC
ATCAACGTCTGGTACGACGAGGTCGAGGTCAACGCGCCCGCGCCGGCCTCATACGACCACTCGGTGGACGAACGCGAGCACACCGTGGAGCAGTGGAAG
CAGCTCATCTACCAGGAGGTGGTCGAGTACTCCGCGCCGCCCCACCCCCCGCCCCCGCACCCCCCGCCCGCCGACCACGCGCAGCCCGCATTCACCACG
TAG

>NM_001173153.1 [alpha-amylase precursor] ID:NP_001166624.1

ATGTTTCGGTACATCCTTCTACTTTCGGCCGTGACTCTGGCGTTGGCCTATAAAAACCCTCACTATGCATCGGGTCGCACCACCATGGTACATCTCTTC
GAATGGAAGTGGGATGACATCGCTGCTGAATGCGAAAGGTTCCTTGGACCCCGAGGATTCGGTGGTATTCAGGTTTCGCCACCAAACGAGAATTTGGTA
ATCTGGTCCCGCAACCGTCCTTGGTGGGAGCGCTATCAACCAATCTCCTACCGTCTAGTAACAAGATCTGGAAATGAAAATCAATTTTCGAATATGGTG
CGTCGCTGCAACAATGTTGGCGTCAGGATTTATGTGGACGCCATCATCAACCACATGACTGGAACTTGGAATGAGAATGTTGGTACAGGTGGAAGCACG
GCCAATTTCGAAAACTGGCACTATCCTGCTGTTCCTTACGGCAGGAATGACTTCAACTGGCCTCATTGTGTCATTACTGGCAGCGACTACAACTGCTGC
CCTGACAGAGTGCGCAACTGTGAATTGTCTGGTCTGAAAGACTTGAACCAAGGCTCTGATTATGTTAGACAGCAGATTCTAAATTATATGAACCGTCTT
ATTGACATGGGTGTTGCTGGTTTCAGAATTGATGCTGCAAAGCACATGTGGCCTCACGATTTGCGAGTGATCTACGATCGTCTCCGCAATTTAAATACC
GCCCACGGTTTCCCATCCGGTGCTCGTCCCTACATCTACCAAGAAGTCATCGACCTCGGTGGTGAAGCTATCAGTCGCAACGAATACACTCCACTTGCT
GCCGTTACTGAGTTCAGATTTGGACTGGAACTCAGTCAAGCTTTCCAAAGAAGAAACCAGCTTAGATGGTTGGTTAACTGGGGTCCACAGTGGGGCCTT
CTCGCTTCTGGAGACGCTTTGACTTTCATTGACAACCACGACAACCAGAGAGGTCATGGCGCTGGTGGAAACATCTTGACATACAAGCAATCCCGACAA
TACAAGGGCGCTATCGCTTTCATGTTGGCACATCCTTATGGCTACCCTCAATTGATGAGTAGTTTCGCCTTCACGGACACCGAAGCTGGACCTCCAATG
AACAGCAGGGGTGATATCACCTCTCCTACTATCAACGCTGATAATTCTTGCGGCAATGGTTGGATATGCGAGCACCGTTGGCGTCAGATCCACAACATG
GTTGTCTTCAGGAACACCGCTGGTAACGGTGCCCTTACCAACTGGTGGGACAACGGTAGCAACCAGATTGCTTTCTGCCGCGGCAACCAGGCCTTCATT
GCTTTCAACAACGATGCATGGGACATGGACCAGACTCTTCAGACTTGTCTCCCCGCCGGAACATACTGCGATATTATTTCCGGCGCCAGGTCTGGTAAC
CGCTGCACTGGAAAATCTATTGTAGTAGGCAGCGATGGTCGCGCTCTTATCATCCACCGCAGCAACGAGTATGACATGATGGTTGCTATCCATAGGGGT
GCTGACTCGAGATTGTAA

>gi|156044 (B.mori) storage protein 2 (SP2) gene(AAA27848)

ATGAAGTCTGTCTTGATTCTGGCTGGGCTTGTAGCCGTCGCGCTCAGCAGTGCAGTACCAAAACCGAGCACCATAAAGTCAAAAAATGTGGATGCCGTA
TTTGTTGAAAAGCAAAAGAAAATTCTGTCCTTCTTCCAAGATGTGAGCCAACTAAACACTGATGATGAATATTATAAAATTGGCAAAGACTATGATATC
GAAATGAATATGGACAACTACACTAACAAGAAAGCTGTTGAAGAATTTCTGAAGATGTACAGGACTGGTTTTATGCCTAAGAATTTAGAGTTCTCCGTT
TTTTATGACAAGATGAGGGATGAAGCTATTGCTCTATTGGATTTATTCTATTACGCTAAGGACTTTGAAACGTTCTACAAGAGTGCCTGTTTTGCGCGT
GTGCATCTCAATCAAGGTCAATTCTTGTATGCCTTCTACATCGCTGTTATCCAGCGCCCTGATTGCCACGGTTTCGTTGTTCCTGCTCCGTATGAAGTA

TACCCTAAAATGTTTATGAATATGGAAGTGCTGCAAAAAATTTACGTAACAAAGATGCAACATGGCCTCATTAATCCTGAAGCCGCAGCTAAGTATGGC
ATTCACAAGGAAAACGACTACTTCGTTTACAAAGCCAATTATTCTAACGCCGTTTTATACAATAATGAAGAACAAAGGCTGACATACTTCACTGAGGAT
ATTGGCATGAACGCTTACTACTACTACTTCCACTCTCATTTACCGTTCTGGTGGACATCAGAAAAATACGGAGCCCTTAAAGAGCGTCGTGGAGAGGTT
TACTTCTACTTCTACCAGCAATTATTGGCTCGTTACTACTTTGAGCGTCTTACCAATGGACTTGGTAAGATTCCCGAATTCTCATGGTACTCTCCGATA
AAGACTGGATACTATCCATTGATGCTAACTAAGTTTACACCCTTCGCACAAAGACCTGACTACTACAACTTGCACACCGAAGAAAACTATGAAAGAGTA
AGATTCCTTGACACTTATGAGAAGACATTCGTTCAGTTCCTCCAAAAGGACCACTTTGAAGCCTTCGGACAAAAAATTGATTTTCACGACCCGAAAGCC
ATTAACTTCGTCGGCAACTACTGGCAAGATAATGCAGATCTGTATGGAGAAGAAGTCACAAAAGATTACCAACGTTCTTACGAAGTATTTGCGCGCCGT
GTGCTAGGTGCTGCGCCTATGCCATTCGACAAGTACACTTTCATGCCTAGTGCAATGGACTTTTACCAGACTTCTCTTCGTGATCCTGCTTTCTATCAG
CTCTACAACAGAATTGTGGAATACATCGTTGAGTTCAAGCAATACTTGAAGCCTTACACTCAAGACAAACTTTACTTTGATGGTGTCAAGATAACTGAT
GTTAAAGTCGACAAATTGACAACATTCTTCGAGAACTTTGAATTCGACGCCAGCAACAGCGTGTACTTTAGTAAGGAGGAGATTAAGAACAATCACGTC
CATGAGTTAAGGTGCGCCACACGATTGAACCACAGCCCCTTCAACGTTAACATTGAGGTTGATTCTAATGTCGCCAGTGACGCTGTTGTCAAAATGTTG
CTGGCCCCCAAATACGATGACAACGGAATACCTCTCACATTAGAGGACAACTGGATGAAATTCTTCGAGTTGGACTGGTTCACAACTAAACTCACCGCT
GGTCAGAACAAGATTATCCGCAATTCGAATGAATTTGTCATATTTAAAGAAGACTCCGTGCCAATGACTGAAATTATGAAGATGCTCGACGAAGGAAAA
GTACCTTTTGATATGTCGGAAGAGTTCTGTTACATGCCTAAAAGACTCATGCTGCCTAGAGGTACTGAAGGTGGATTCCCATTCCAGCTCTTTGTTTTC
GTCTATCCATTCGACAACAAAGGCAAGGACTTGGCTCCTTTCGAATCTTTTGTTCTTGACAATAACCTCTTGGCTTCCCTCTGGATCGCCCCGTTGTTG
ATGCATTATTCAAGGTTCCTAACATGTATTTCAAGGATATTTTCATTTACCACGAGGGTGAACGGTTCCCTTACAAATTCAATCTTCCTTCGTATGACA
CACATGATAATGTTGTTCCAAAAAATTAAATTTTAA

>XM_004925443.1[liver carboxylesterase-like] ID:XP_004925500.1

ATGGTTGTGGTTTCCGTAAGCGAAGGTCTGTTAGAAGGAGAGCTTGTTAAGAATGAATACGGAGGAACATTCTACAGTTTCAAGGGAATACCTTACGCG
GAGCCGCCTGTGGGAAATCTTAGGTTCAAGGCCCCGTAcCCCGTTAAGCCATGGCCTGGAGTCCGCAATGCGAAGAAATTTGGACCAGTCTGTTTTCAA
AAGAACGTGCTTGTACCATCCAATGGAAGCGAAGACTGTCTTTATCTAAACGTTTACACCCCAAACATCAAACCTGACAGGCCAATTCCTGTCATGGTC
TGGATTCACGGAGGTGCATACAAATGCGGTAGTGGTAACGATGATTTCTATGGACCAGAATATCTTTTAAGACAAGGGGTCATTCTCGTTACGCTTAAC
TACAGACTGGAAGTACTCGGTTTCTTGTGCTTGGACACCGAAGATATACCAGGAAACGCTGGCATGAAAGACCAAGTCGCTGCTCTTCGTTGGGTCAAA
AAGAACATCGGAAACTTCGGCGGCGATCCTGAAAACATCACAGTTTTCGGTGAAAGTGCTGGAGGAAGTAGTATCAGCTATCACTTGATATCTCCGATG
TCAAAAGGACTCTTCAAAAAAGCAATAATACAAAGTGGCGCAATTACTTGTTGGTGGGCACTAACTTTCGAACCTCGTCAAAGAGCTCTAATTTTAGCC
AAGAAATTGGGTTTCGATTCGGAAGATGATAAGGAACTTTACGAATTCTTTAAGAATCAACCCTTGGAATCACTAGCGGACGTGGGATTGCCCGTGATA
CACTCCCAAAAAGAGAGTGAAATTTGTTTGTCTGTCACAGATGAAAAGCAATTTGGTGATAATGAAAGATTCTTTTATGGCGACATCATCGATGCCCTC
AACAACAGATTGGACAATGTCGATGTTATAATTGGATACAATCAAGACGAAGGCTTGATAACTTTGTTGGAAAACGATAATGATATCACAAAAGCCGTA
AATTTGGCGAAAATGTACCTCGAATATTTCGTACCAAAACCAATCGCATTGAACAGTCCGATCAAACAACAGCTCGAGATCGGTAGAAAATTTAAACAA
TTCTATTTTAAGAACAAGAATATTGGCGACGATTTAGAGAGTCTGGTCAAATATGCAGGCTTTGACATGTTCATTTACAACGTCTTGATTCAGGCAAAG
TTTCTCGCGAAATCCAAGAACAATATATATTTATATAAGTTCACGTGTAAATCTGAAAGGAATGTATTGAGTCGTTTGTTGCAAATTGACAACGTGCTC
GGAGGCAGGCCTGTGACGTGTCACGGCGACGATATTTTTTATATTTTCCCATCCGTGCCGGCTCCGAAAGTTAAAGTAGATTCCGCTGCTTTCAAATTA

ATCGACAATGTAACAAAGCTATGGACGAACTTCGCCAAATATGGAAATCCAACCCCTGGGGATGACTTGGGTGTCAAATGGGCTACATATTCCGTCGAA
GCACAAGATTACCTGGACATTGGCAATGAGCTGGTGGCAGCTAAAAAACCTGAAAGCGATCAAGTTAATTTTTGGGATGATATCTTCAGACAGTATTTA
CCTCATTGGATTTTCTGGTAG

>XM_004922045.1[ser./thr-protein phosphatase 2A 65 kDa] ID:XP_004922102.1

ATGGCAGCGAGTGACTCCGGGACGGATGAATCATTGTATCCGATTGCTGTTCTCATAGATGAGCTAAAGAATGAGGATGTACAGCTTCGTTTGAACTCT
ATAAAGAAATTGTCGACGATCGCGTTGGCTCTGGGCGTGGAGAGGACTAAATCGGAGTTGATCCCGTTCCTCACTGAGACAATCTATGATGAAGACGAG
GTTCTCTTAGCACTCGCTGAACAACTTGGTAACTTCATAAACTTGGTTGGAGGGGGAGAATTTGCACACTGTCTCCTTCCTCCACTAGAGACATTAGCA
GCAGTGGAAGAAACAGTAGTCAGAGACAAGGCAGTGGCCTCGCTCCGTGCTGTTGCCGAGCACCATAGCCCCCAGGCACTCGAGGAACACTTTGTGCCA
TTAGTACAGCGTCTTGCCGGAGGAGACTGGTTTACTTCCAGAACATCTGCCTGTGGACTCTTTAGTGTCTGCTACCCACGCGTTTCGGCCGTAGTGAAG
GCGGAACTTCGTCAGCATTTTTGCTCGCTCTGCCAAGATGACACTCCGATGGTGCGACGCGCTGCCGCCTACAAACTTGGGGAGTTCGCTAAAGTCGTA
GAAATCGAATACGTCAAGAGCGATCTCATTCCCATCTTTGTGTTTTTGGCAAAGGATGACCAAGACTCGGTACGTTTGCTAGCCGCAGAGGCATGTGCT
GTTGTGGCTTCTCTGCTGGCACCCGAGGATATGGAGCAACATGTGATGCCGACCGTGCGGGCTCGTGCCGGAGATACTTCGTGGAGGGTGCGGTACATG
GTCGCTGATAAGTTCGTAGAGCTGCAACAAGCTGTTGGTCCAGAGCTAGCTCGTACAGATCTAGCACAGATCTTCCAGGCACTGCTCAAGGACTCGGAG
GCTGAGGTTAGAGCCGCAGCTGCTGGGAAGGTCAAGGACTTCTGTATGAACTTGGATAAAGCTCATCAAGAACACATCATCATGACCATGATCTTGCCA
CAGATCAAGGACTTGGTGTGTGATGCCAACCAACACGTCAAGTCTGCACTAGCCTCCGTCATCATGGGCCTGAGCCCCATAGTGGGCAGACAGAACACT
ATTGAACATCTCCTGCCGCTGTTCCTCACACAACTGAAGGATGAATGTCCCGAAGTCCGACTCAACATAATCTCCAACCTCGAGTGTGTGAATGAAGTG
ATCGGCATCCAACAGCTGGTGCAGTCTCTCCTCCCCGCCATCGTGGAGCTGGCAGAGGACACCAAGTGGAGGGTCCGTCTGGCCATCATCGAGCACATG
CCGCTACTGGCGGGACAGCTGGGCCAGGAGTTCTTCGACGAGAAGCTGACCAGCTTGTGCATGTCGTGGCTGGTGGACCACGTGTACGCCATCCGCGAG
GCTGCGACCCTCAACCTGAAGAAGCTGGTGGAGCAGTACGGGCCGCAGTGGGCAGAGAACAACGTCATACCCAAAGTACTGAACATGTCGCACGAGCAG
AACTACTTGCACAGGATGACGTACTTGTTCTGCATTAACGTGCTGTCCGAAGTGTGCGGCAAGGACATCACGACCAGGGTGCTGCTGCCGACCGTGTTG
TCTATGGCCGACGACAATGTTGCCAACGTGCGGTTCAATGTTGCCAAGACGCTGCAGATAATGGCCAAATACTTGGACCCGGCCGTCATCCAGCCCCAA
GTCAAGCCGGTCCTGGAGAAGCTGAACGTGGACCCCGACGTCGACGTCAAGTACTTCGCATCGGAAGCGATCGCCGGAATAGCTGGATAA

>NM_001043385.1 [cathepsin B precursor] ID:NP_001036850.1]

ATGTTTATTTCGCGTGCGGCGTATGTTACGCTGGTGTGCGTTCTTGCAGCGGCCAAAGACTTACCGTATCCACTCTCCGATGAATTCATCAACACGATT
AACCTAAAGCAAAATAGTTGGAAGGCGGGACGTAATTTTCCGCGCGACACATCGTTCGCGCATCTTAAGAAAATAATGGGAGTTATCGAGGATGAACAT
TTTGCGACCCTGCCAATAAAGACTCATAAAATCGATTTAATCGCCGGTCTGCCGGAAAACTTCGATCCCAGAGACAAATGGCCTGACTGTCCAACGTTG
AATGAAGTCAGAGATCAAGGGTCTTGTGGCAGTTGTTGGGCTTTCGGGGCCGTCGAAGCTATGACAGACAGAGTATGTACCTATTCTAACGGAACTAAA
CATTTTCATTTTTCTGCCGAGGATTTGCTTAGTTGCTGCCCTATTTGTGGACTGGGATGCAGCGGAGGAATGCCGAGACTAGCTTGGGAATATTGGAAG
CACTTCGGTCTAGTATCAGGAGGTAGTTACAATTCCAGTCAAGGTTGCAGACCTTACGAGATCCCTCCGTGTGAACATCACGTACCCGGCAACAGGATG

CCCTGTAGCGGTGACACGAAGACTCCAAAATGCACAAAAAAATGCGAATCTGGATACGACGTTAATTACAAACAAGACAAACAATACGGAAAACATGTA
TATACTGTGTCCGGAGACGAAGACCACATCCGCGCCGAATTGTTCAAGAATGGTCCGGTCGAAGGTGCTTTCACAGTATATTCAGATTTGCTGTCGTAC
AAGAGTGGTGTATACAAACACACACAAGGGGACGCTCTCGGCGGGCACGCCGTCAAGATATTAGGCTGGGGTGTCGAAAATGATAACAAATATTGGCTT
ATTGCAAACTCGTGGAACTCAGATTGGGGAGACAATGGCTTCTTCAAAATTTTGCGCGGCGAGGATCACTGCGGTATTGAGAGCTCCATTGTTACGGGT
GAACCGTTCTTAGATGAACACTAG

>XM_004928291.1 [RAC serine/threonine-protein kinase] ID:XP_004928348.1

ATGGCGATGGCGGCGCCCGGGATCATCGTGAAGGAAGGATGGCTGCAGAAACGTGGTGAACACATCCGGAATTGGCGAGATCGGTACTTCATATTGTTC
GACACGGGGGACCTGGTGGGCTTCAAGACCCAGCCGGAGCGGAACAACTACAGGGACCCTCTCAACAAGTTCACCGTACGCGACTGCCAGATCATGGCT
GTGGACAAACCTCGCCCCTACACTTTTACCATACGTGGCCTGCAGTGGACCACTGTTATTGAAAGGAACTTCTCAGTTGACAACGAAAAAGAAAGAGAA
GAATGGGTGAAGGCCATCCGAGAAGTTGCCTCGCAACTGAGCACCGGAGGGCCCAGTTCAGCATCGATGTCCGATGCTGACGATCGCGACATGGCACAG
CTCGGCACCAGCTTCCGTGACCCCCGCCGCATCACCTTAGAAAAATTCGAATTCGTGAAGGTGCTAGGCAAGGGTACGTTTGGGAAGGTGGTTCTGAGC
CGTGAGAAGGGAACGGGAAAGCTGTACGCTATGAAGATATTGAAGAAACACCTCATTATACAGAAGGATGAGGTCGCGCACACCATCACCGAGAACCGA
GTGCTCAAGAAGACCAAGCATCCATTTTTGACGGCTCTCCGCTATTCCTTCCAAACGGCAGACCGTGTCTGCTTCGTAATGGAATACGCTAATGGCGGT
GAATTGTTCTTTCATTTGTCCCGCGAGCGTTCGTTCACCGAGGACCGCACCAGGTTCTACGGAGCCGAGATAGTATCTGCTCTCGGGTACCTCCACTCC
GAGGGCATTATATATCGGGATTTGAAACTCGAGAATTTGCTGCTAGACAAAGACGGACACATCAAGATTGCAGACTTCGGACTATGCAAGNNNNNNATA
ACGTATGGACGCACGTCCAAGACGTTCTGTGGCACCCCCGAGTACCTCGCCCCTGAGGTCATAGAGGACTCCGATTATGGTCCCGCCGTAGATTGGTGG
GGTACCGGTGTAGTGATGTACGAGATGGCGTGCGGCCGGCTGCCCTTCTACAACCGGGACCACGACGTGCTGTTCGGGCTCATCATCAACGAGGAGGTC
CGGTTCCCCCGCAGCGTGTCGGCCGCGTGTCGCTCGCTGCTGGACGGGTTGCTGACCAAGGACCCTCGCGCGCGGCTGGGCGCCGGGCCGGACGACGCG
CACGAGATCATGAACCACCCCTTCTTCGCGTCGATCAACTGGAACGACCTCGTGGCCAAGAAGATCCCGCCCCCGTTCAAGCCTCAAGTGGAATCCGAC
ATCGACACCCGGTACTTCGACTCTGAGTTCACCGGGGAGAGCGTCGAGCTCACGCCGCCAGAGAATGAGAACAGCCTCGCCTTTATACAGGAGGAGAGC
TTCCCTCAGTTCTCCTATCAGGATATATGTTCATCAGCGCATTCAGCCCTCTCTCACATGTCGCATCACTCCGCGCTGACCGACAAACGGCACTAG

>NM_001048238.1[receptor for activated protein kinase C RACK 1 isoform 1] ID:NP_001041703.1

ATGTCTGAAACATTAAAACTCCGAGGAACTCTTAGAGGCCACAATGGCTGGGTTACTCAAATTGCAACTAATCCGAAATACCCGGACATGATCTTATCT
TCCTCTCGAGACAAAACTCTCATCGTGTGGAAGCTGACCAGAGACGAAAACAACTACGGTATACCTCAAAAGCGTTTATACGGTCATTCGCACTTCATT
TCTGATGTTGTGCTGTCTAGTGACGGTAATTACGCCCTTTCCGGTTCTTGGGACAAGACTTTGCGTTTGTGGGATCTCGCTGCAGGCAAGACTACCAGA
CGATTCGAGGACCATACTAAGGATGTACTCTCGGTAGCCTTCTCAGTTGACAACCGTCAGATAGTGTCTGGTTCTCGCGACAAGACTATCAAACTCTGG
AACACCCTTGCGGAGTGCAAGTATACCATCCAAGATGATGGACACAGCGATTGGGTGTCATGTGTCAGATTCTCACCCAATCATGCCAACCCCATTATT
GTATCCTGTGGTTGGGACAGAACTGTCAAGGTCTGGCATCTCACTAACTGTAAGCTCAAGATTAACCATTTGGGTCACTCTGGCTATCTGAACACTGTT
ACTGTATCACCTGATGGCTCTTTGTGTGCATCTGGTGGTAAGGATATGAAGGCGATGTTGTGGGATCTGAACGATGGCAAACACCTCCACACCTTAGAC

CACAATGATATCATCACGGCCTTGTGCTTCTCACCCAACAGATACTGGCTATGTGCTGCTTTCGGACCTTCCATCAAGATCTGGGATCTGGAAAGCAAG
GAGATGGTTGAAGAGCTCAGGCCTGAAATCATTAACCAAACGCAAACTTCCAAGACAGACCCTCCTCAGTGTCTCTCCCTAGCATGGTCTACTGATGGA
CAAACCTTATTTGCTGGTTACTCAGATAATACTATAAGAGTGTGGCAGGTTTCCATCTCAGCTCGATAA

>NM_001043588.1[clip domain serine protease 11 precursor]

ATGTACAAGCTTCTACTCATCGGCTTTCTCGCGTCGGCGTGCGCGCAGAACATGGACACCGGGGATCTGGAGTCGATCATTAATCAGATCTTCACATCA
GCGAAGCCCCCGACGCAATTGCAGCCGGTCACGCAGCCTAGTGTCGCTGACAGGGCTCCATCGACCCTGGTGCCTGGCGTCTCTACGAACGATGACCTT
TCGTGCCAGACCTCCGACGGCCAAGAAGGGGAGTGCGTCAACTATTACTTGTGCAACGCGGCCAATAACACCATAATCACTGACGGAACAAACGTCATC
GATATAAGAGTCGGCAGTGGCCCGTGTTCATCGTACATCGACGTCTGCTGTCTGGCTCCCGACCAGAGACCGCCAACAGATCCCATCACGCCCAGGCCG
GAGACCCTGCCAATGAACCAGGGCTGCGGCTGGCGGAACCCTGACGGCGTTGCCTTCCGGACCACGGGCGACGTGGACGGCGAGACCAAGTTCGGAGAG
TTCCCCTGGATGGTCGCCATCCTCAAGGTTGAACCAGTCGATGACAACGAGCCCGAAGGACAGAAGCTGAACGTTTACGTCGGGGGAGGCTCGCTGATC
CATCCGAACGTGGTGCTCACGGCCGCTCACTACGTTGCCGCCGCTAAGGAACTGAAGATCAGAGCCGGCGAATGGGACACGCAGAACACGAAGGAGATA
TATCCGTATCAAGACAGGACAGTCAAGGAAATCGTGATACACAAGGACTTCAATAAGGGGAACCTGTTCTACGACATAGCTCTGCTGTTCCTCGAGACT
CCAGTGGATTCAGCCCCGAACGTGGGAGTGGCGTGTCTCCCACCGGCCAGGGAGCGCGCCCCCGCCGGCGTCAGGTGCTTCGCCACCGGCTGGGGGAAG
GACAAGTTCGGGAAGGAAGGTAGATACCAGGTCATCATGAAGAAGGTGGACGTGCCGGTCGTGGACCGGAACACCTGCCAGAGCCAGTTGAGGAGGACG
CGGCTGGGGCGGTTCTTCCAGTTGCACTCGACGTTCATGTGCGCGGGGGGGGAGCCCGACAAGGACACCTGCCGGGGGGACGGGGGGTCGCCCCTCGTG
TGCCCCATAGACTATGAGAAGAATCGCTATGTCCAATACGGCATCGTAGCCTGGGGCATCGGCTGCGGAGAGGACGGCACTCCAGGAGTTTACGTGGAC
GTTTCGAACCTGAGGACCTGGATCGACGACAAGGTCGCCGGCAAGGGATACGATACTAGATCCTACGAGCCCTAG

>NM_001111352.2 [alpha-esterase 45] ID:NP_001104822.1]
ATGGTCAAAGTAAAAGTAACGCAAGGCTGGTTGGAAGGCGAGGTGCTGGAGACCGTGACCGGGTATAGAAAGTACTATAGCTTCAAGGGTATTCCATAT
GCTGAACCTCCCTTAGGAAAACTCAGATTCAAGGCTCCGCGACCAGCGCTGCCCTGGGAGGGAGTCAAAAAAGCAACACAGCATGGACCTGTATGCCCT
CAAGTGGACATCTTCACAGAAATCCTGATTCCGGGAAGTGAAGATTGTCTTTATTTAAACGTGTACACGCCCGAAATCAATTCTGAAACGCTCTTACCA
GTCATGTTCTTCATCCATGGAGGTGGTTACAAGAGTGGCTCAGGAAATGTTGACAATTATGGACCCGACTTCCTTGTAGCCCACGGAATAATATTAGTC
ACGATTAATTACAGACTTGACGCATTAGGATTCCTTTGCTTGGACACTGAAGAAGTGCCAGGAAATGCTGGTATGAAAGACCAAGTAGCTGCTTTAAAG
TGGGTTAAAGAGAATATTTCAAGTTTTGGTGGAGATCCAGATAACATTACTGTGTTTGGTGAAAGTGCCGGTGGGGCGTCTACATCTTTCCACGTCATA
TCGCCGATGTCAAAAGGCTTGTTTAAAAGAGTGATTTCGATGAGCGGGGTCCTATTCTGTGATTGGTCCATAGCATTCGAACCAAGAAGAAGAGCGTTC
AAACTTGGACAGCTTTTGGGAATTGAAACCGAGAATCCAGATGAGTTACTCGAATATCTACAAAACGTTCCAGTAGAGAAATTAGTCAATACAGATCCT
AACATAATAACAATGGAAGAACTACACAAAAATATGCTCAAATTTTATCATTTCACGCCAGTTGTCGAAAAAGATTTTGGTCAAGAACACTTTTTGACA
CGTGAACCAATTGAAGTCCTTAAACATGGAAATATTAATGATGTAGATATGCTATTAGGTTTCACAAATGAAGAAGCAATAATAGGAATAACGCTTTTA
GAGAAACATCTAGTGCATTTGTACAATAAGTACATCGATTTGTTCGCACCTAGGTTGATAACAATTAAGGCGACACCAGATAAAGTCTTAGAAATATCC

GACAGAATACGTCATTTTTATTTCGGCAATAAAAAGGTTAATTTAGAATCTATGAGGGAATTTGTCACGTTTGTTAACGACTGTGGATTTATATATCCC
ATTCACAGATTTGCGGGTAAATTGTCGGATCTAATTAATACGAAAGTTTTCATGTACAAATTTTCGATGTTTTCAAATCGAAATGTGTATGGTGCGGCC
GGTGCTAATTACAAGATAACTGGGGCCTCGCATTTGGAAGATTTGATGTATCTGTTTGACGCCAAAAAATTTGACTTGAAGATTGAGACAACGAGCAGG
GAATATCGTTTAATTAATCTGGCTTGCACAATTTTTACCAATTTTGCGAAATTTGGTAATCCAACACCGGACACATCTCTGGGAGTGAAGTGGCCAGAA
TACAATAAAGAGACAAGAAAATACTGCGACATCGGAGATGAGCTCACCATTGGACTGGATAACTACAGACAGACAGTCGACTTCTGGAAAAAAGTATAT
GAAGATGCCGGTCTAGAATTCTAA

>NM_001128314.1 [alpha-esterase 3 precursor] ID:NP_001121786.1

ATGCGGCTTAAAGTTATATTACCTCTGCTTTTCTTCGCCAGTATCTCTGGTGAGGACTTTGAGTTGCCCATAGTAAATATAGGACAAGGCGCTGTGCTC
GGTTCTCATGCTCACGATGGGGAATATTTTACGTTTTACGGGATCCCTTATGCCGATTCTACGTCAGGACCTCATCGGTTTAAGGCACCATCATCACCT
CCAAAATTTGAAGGTGCCTTTGTAGCGAACAGAAGAGATGTAAAATGTGTAAGACCACTTGGAGTGGGCTATGAAGGGACAGAGGATTGTCTAGTAGTT
GACGTGTTAACGCCTAATCTGGATGCTGCAGCTCAACTACCAGTGATGGTCTGGGTGAAGGGAAAAGAGTTCGACAGTGAATATAATCAAGATTTTTCA
TTCCGTAATTTCGTCCAGAAAGATGTCGTTGTAGTTTCAATCAACTTCCGTGAATCTATACTAGGATTTTTATGTTTGGGCACTGAAGTGGCTCCGGGA
AATGCTGGATTGAAAGATATCATAGCCGGATTGAAATGGGTAAAAGAAAATATTGCTCATTTTGGAGGAAACCCAGACGATGTTACTTTGTTTGGTCAT
GGTAGTGGTGCTGCTGCTGTTGATTTGGTTACTTTGTCACCCTTAGCTAATGGATTAGTACACAAAGCTATAGCACAAAGCGGTAATGCATTTGCTCCA
TGGGCTGTAACCCGTGACAACTTAAACTATGCCATTCAAGTAGCAGAGGCTCTTGGACATATCGTGACGAACATTCAGGAACTCTCCGAAATATTTACT
AGAACCAGTGTAGCTGCACTTATGGCCGTAATTAATGAATTGGATCTTACAGATAACTCATTGGCGTTTGCTCCATGCTTAGAACGAGAAGCGTTGAGT
GTAGAATCATTTATCACTAAATCGCCCTACCAAACAATTTTAGATGGAGAATTTTTACAAATACCTTTCATGACTGGGTTTGTAGAAAACGAAGGTACA
ATTCGTGCCACAGAAGCAATAGAACACGACTGGTTGCAAAGAATGGAAACTTCATTTCCAGACTTTCTCCAACCGGACTTACAATTTGAAAACGCAGAG
GGAGAATCAGCAATTGCAGAGGAAATAAAAGATTTTTATTTCAAAAACGAACTTATAAGTATGTTCAATATTGAGGACTACCTATCGTATCATGGTGAT
ACAATGATACTAGTGTCAACAATTAGAGAAGCTCGTATTCGTGCTGCAAGTTCAAGTTCACCAGTTTATTTATATCAGTTTTCGTACAGTGGATCTTTA
GGAAAACCTTTCGTTGGACCTATTCAAGTCGACGCAGCTGCACATAGCGAAGAATTGGCCTATATGTTTTATGAAAAATCGGATGATAATATCCCTTTG
CTCGATCTTACAATTGGAGATATTTTAGTCGAAAGATGGACAAACTTCGCCAAAACTGGGGCACCGGTAAGTGAGACTTCTCAGGTAGTGTGGAGACCT
TTTACCACCGAAAACAACCATTATTTAAGAATCCTTGATGACAGCGAAGTCCGAGGTGATCAAGAAACTACACTGGAACTGGATTTACAAAACCCTTAT
CCAGAAACACTATTATTCTGGGAGGAAATTTACAATGAACATTTCTTGGATGCCAGAGGTAAATGGGAACTAAGTGAAAGGACTGAGGGAACCGAAGAC
GGTGATGAAAACGGAAGTGGAGAAGAACTTCCGGAAAATGGTGGAGAGGCCGACAACGGAAGTGGTGAAGAAGTAGACGAGGGAAATGAAGGCGAGCCT
GAAGAAGACGAGGGAAATGAAGGCGAGCCTGAAGAAGACGAGGGAAATGAAGGAGAGCCTGAAGAAGATAATACACCCGGATCAGCATCAACGGCTATA
GCTTATACGTTTCCCATACTAACCGTGTTCGCGCTCATAAACAAATTGCACTTATCTCAGATCATGTCTTAA

>NM_001043607.1 [ADP/ATP translocase] ID:NP_001037072.1]

ATGTCGAACCTCGCCGATCCGGTCGCGTTCGCTAAGGACTTCCTGGCTGGCGGTATCTCCGCCGCCGTCTCCAAGACCGCCGTAGCACCCATCGAGCGT
GTCAAGCTGCTGCTCCAAGTACAGCACGTCAGCAAGCAGATCGCCGCCGACCAGCGTTACAAGGGTATCGTCGATGCCTTCGTCCGTATCCCCAAGGAG
CAGGGTCTCCTTTCATTCTGGCGTGGTAACTTCGCCAACGTCATCAGGTACTTCCCGACCCAGGCGCTCAACTTCGCCTTCAAGGACAAGTACAAGCAG
GTGTTCCTCGGCGGTGTTGACAAGAAGACGCAGTTCTGGCGTTACTTCGCTGGTAATCTGGCCTCCGGTGGTGCCGCCGGAGCCACCTCTCTGTGCTTC
GTGTACCCCCTCGACTTCGCACGTACCCGTCTCGCCGCCGATGTCGGTAAGGGAGATGGCCAGCGTGAATTCTCCGGTCTCGGAAACTGCATCAGCAAG
ATCTTCAAGTCCGACGGTCTGATCGGTCTGTACAGAGGTTTCGGTGTGTCCGTGCAAGGTATCATCATCTACCGTGCCTCATACTTCGGTTTCTACGAC
ACGGCCCGCGGCATGCTGCCCGACCCTAAGAACACACCCATTGTAATCAGCTGGGCCATCGCTCAGACCGTAACCACAGTCGCCGGTATCATCTCCTAT
CCCTTCGACACAGTCCGTAGGCGCATGATGATGCAGTCTGGTCGTGCCAAGAGCGATATCCTGTACAAGAACACCATCCACTGCTGGGCTACCATTGCC
AAGACCGAGGGAACCTCGGCCTTCTTCAAGGGAGCCTTCTCCAACGTCCTCAGAGGTACTGGTGGTGCCTTCGTGCTCGTCTTGTATGATGAGATCAAG
AAGGTCCTGTAA

>NM_001110363.1 [cytochrome P450 monooxygenase Cyp4M5]

ATGTTCGTTTACTTGATATTTATCGCTTCGTTTTTCTTATTAATTCATTTAGCTTTCAATTATAATTCGAAAGCTGTGATGATGAATAAAGTTCCTGGT
CCAAAGTTGTCGTTCATTCTCGGGAATGCACCAGAGATAATGATGTTGTCATCAGTTGAGTTAATGAAACTGGCCAGGAAATTCGCAAGTCGCTGGGAT
GGAATATATAGGATTTGGGCGTTTCCTTTGTCCATTATAAATATATACAATCCGGATGACGTAGAGGTGATAGTTTCGACCACAAAACACAACGAAAAG
AGTTCTGTGTATAAATTTTTGAAGCCGTGGCTTGGGGATGGATTGCTAATAAGTAAAGGAGAAAAATGGCAGCAAAGAAGAAAAATCCTTACGCCCGCT
TTCCACTTCAGTATCTTGCGACAGTTCAGTGTCATAATTGAAGAAAATTCTCAACGTTTGGTGGAATCATTGGAGAAATGCATCGGAAAACCTATAGAC
ATAGTACCTGTAGTATCAGAGTATACACTGAATTCCATTTGTGAGACCTCAATGGGCACTCAACTAAGCGACAAAACAGAAGATGCGTGGAAGGCATAC
AAGGACGCCATATATGAGCTCGGACCGTACTTCTTTCAAAGATTCACTAGGGTTTATTTATATTTTGACATTATCTTCTATTTAACATCTTTATGGAGA
AAAATGAAAAAACCTTTAAAGTCACTCCACGGATTTACTTCTACCGTTATAAAAGAAAGGAAAATTTACGTTGAACAAAATGGTGTTAAATTCGGCGAG
GACGTCAACGATGATGACCTTTACATATACAAAAAGAGGAGAAAAACTGCCATGCTGGATTTACTGATAGCTGCGCAAAAAGACGGAGAGATCGACGAT
CACGGAATACAGGAAGAAGTGGACACTTTTATGTTCGAAGGTCACGATACCACTGCGTCTGGATTGACATTTTGTTTTATGCTACTCGCCAATCACAGA
GCAGTTCAGGATAAGATTGTGGAAGAAATAAATGATATTATGGGAGATTCAACTCGACGCGCGAACTTGGAGGACTTGTCCAAGATGAAGTACTTGGAA
TGTTGCATTAAGGAGTCTTTAAGACTATATCCACCTGTGCATTTTATTAGCCGTAACTTAAACGAGCCTGTTGTTTTAAGTAACTATGAAATACCCGCC
GGTTCGTTCTGCCACATTCACATCTTCGACCTTCATCGACGCGCCGACATATACGAAGATCCACTAGTCTACGATCCAGATCGGTTCTCTCCAGAAAAC
AGCAAAGGACGACATCCGTACGCATACATACCATTCAGTGCTGGACCGAGAAATTGCATTGGTCAAAAATTCGCCATGATAGAAATGAAATCTGCCGTT
GCTGAAGTACTGCGAAAATACGAACTTGTGCCGGTAACGCGTCCTTCAGAAATTGAACTTATTGCCGACATCATACTGAGGAACTCTGGCCCGGTTGAG
ATTACATTTAATAAAAGGACCAAATAA

>EF542811.1 [cytochrome P450 CYP9A19] ID:ABQ18318.1]

ATGATTATCATTATATGGACCTTAGCTATAGGTTTGGCTTTCTTACTGTACCTGAAGCAGATATATTGCTACTTCAGCAAGCATGAAATCAAGAGCATA
ACCCCGCTTCCGATACTGGGCAACATGGGCAAGATTGTCTTCAAGATTAACCATTTTGTTGATGATATTTCTCAATTGTACAACAAATTTCCAGAAGAA
AGATTCGTTGGTAGATATGAGTTTGTAAATCCGGTGATCTACATCAGAGACATTGAGATCGTAAAGAGAATCACAATTAAAGACTTTGAACATTTCCTG
GATCATCGCACGATCGTCAATGAGGAAACCGATCCCATTTTCGGAAGGAATCTGTTTTCTTTGAAAGGCCAGGAATGGAAAGATATGCGATCCACTTTA
TCGCCAGCCTTCACCAGCTCCAAGATGAAGCTTATGATGCCCTTAATAGTCGAAGTTGGAGAGCAGATGATCTACGCGTTAAAAAAGAACATTAAAAAC
TCTGGTGTCGGATACGTGGACATAGACACTAAAGACCTGACCACTAGATACGCCAACGATGTGATCGCCTCCTGCGCCTTCGGTCTGAAGGTCGACTCC
CTCACCGAAGAGAACAACCAATTCTACGCGATGGGGAAAGCCGCTTCGAATTTCAGTTTCAAACAGATCCTACTCTTGTTGGGATTTATCTCATTTCCT
AAGATGATGAAGATGACGAAATTCACGCTGTTTTCGGAAGAAACGTCCGGATTCTTTAAAGAGCTGATAATGGGTACCATGAAAGACAGAGAAATGCGG
AAAATCATCAGACCAGACATGATCCATCTACTCATGGAAGCTAAGAAAGGAAAATTAGTCCACGATGATAAATCCAGCAAAGACACAGATGCCGGATTC
GCCACGGTAGAAGAATCTGCTGTTGGCAAGAAACAAATCGATAGAGTCTGGACTGATGACGACATCATCGCTCAAGCAGTTCTTTTCTTCGTTGCTGGT
TTTGAAACCGTTTCATCGGCAATGACGTTCCTCCTTCACGAATTGGCTCTCAACCCTGAAGTACAAGACAAATTGGTTGAAGAAATCAAGGAGAACAAG
GAGAGGAACAATGGAAAATTTGACTACAACTCCATACAGAATATGGTGTATCTGGATATGGTTGTTTCAGAACTCCTAAGGCTGTGGCCGCCAGGTGTC
TCCATGGATAGAATCTGCGTTCAAGACTACAACTTGGGGAAACCAAATGACAAGGCCAAAAGAGACTTCATACTTCGCAAAGGTACGGGTGTAGCTATT
CCCGTATGGGCTTTCCACCGAAACCCGGAATTCTTCCCGGACCCCCTGAAATTCGATCCCGAAAGGTTCTCAGAGGAGAACAAACACAACATCAAGCCT
TTCGCGTATCTACCATTTGGAGTGGGACCTAGGAATTGTATAGGTTCTAGGTTCGCTTTATGCGAAGTCAAAGTGATGGCATACCAGCTACTCCAGCAT
ATGGAGATATCTCCGTGCGAGAAGACGTGTATCCCCTCGAAGCTCAGCAAGGAAACCTTCAACCTTCGTCTTGAAGGTGGTCATTGGGTCAGGCTAAAG
ATTAGAGATTGA

>XM_004924554.1 [esterase FE4-like] ID:XP_004924611.1

ATGTGGTGGAGCACGTGTTTAAGTTTATGTGCTATTTTGGCTAGCGTAAGGCCAGATAGCACCGATTCAAGAGAAGTCAAAATATCTCAAGGAACCGTG
AAGGGGTATAGGGATCCCGAAGGTGATCTGTTTGCTTTTTATAATATACCCTATGCTACGGCTCCAAGCGGAACGGCGCGATTCAAGGCGCCGCTTCCA
CCACCGGTGTGGCTGAACACTTTCGAAGCAGTAGATAAAGGCGTCATTTGCCCACAGACGCCCCTTGGACCAGTACATAGCAATTTAACGCAACAAGAA
AATTGCCTTATCGTAAATGTGTTCGTCCCGGACACGGAGGAAAAGAACTTGCCTGTGGTGGTCGCCGTTCACGGAGGAGCTTTTCAAATAGGATACGGC
AATTGGGTCGAACTTAAAGAGCTTATGAGGATTAATAAAGTAATAGTTGTTGACTTTAATTATCGTCTCGGCGTACACGGGTTTTTGTGCCTGGGCACT
GAAGACGCTCCAGGCAACGCTGGATTGAAAGATCAAGTGGCATTATTGCGCTGGGTCAACAGAAATATTGGCAGCTTCGGTGGAAACCCAGATGACGTT
ACGCTGATAGGATACAGCGCAGGATCTGCAGCAGTGGATTTGTTGTTGCTCTCAAAATCGGTACGAGGTCTGTTTCACAAGATCATTCTTGAGAGTGGA
TCGAATTTAGCACCGTTCGCGGTCCAATTAGATCCATTAAAAAATGCTAAAGATTACGCTAAACAACTGAAGTTTGAAAGGTTTGATGACATATACGCT
TTAGAAGAGTTTTACAAAACAGCATCGTACCAAAAATTAACTTCCGATGCATTTATAGATAGACCTGACTCAACGTTTATGTTTGTACCTTGCGTCGAA
CGTGATGTCGGAGAGGAAATAATACTTGATGATGCTCCAATTAACCTTCTACAAAATAAACAGTACCCAAAAGTACCTATGCTAATTGGCTTTGCCAAC
ATGGAAGGCCTCATGCGTATAGATCTCTTTGACGAGTGGAAGACAAAAATGAATAAACGATTTTCGGATTTCCTCCCTGCTGATTTGCACTTCAAAAAC
GAAAGCCAAAAGAAGAAAGTAGCGAAAATTATCAAAGAATTCTATTTTGGTGATCGTTCAGTGAACACTGATGGCGTTTTGAATTATGTGAACTATTTT
AGTGATGTTATATTCATGACACCAGCCCTTAGATCTATTAAATTACTAACAGAGGCTGGTCACGATCAAGTATATTTGTATGAATATTCTTTTGTTGAT
GAAAATGTTCCGGTGGTGCCGCATACGGATGTCCGCGGAGCGAATCACTGTGCACAAACAATGGCAGTAGTGGATGGACCTGCTATAGCGGCCAGAGAA

GATGTAGTGGATACTGAAGAATATAGAGAAATGAGAAAGACAATGAGAACGTTGTGGAATAATTTCATAACAACAGGCAAACCAGTTCCGGAAGGCTCA
TCTTTACCGAAATGGCCATCTATTAATAAGGATCTTTCACCGTATATGTCTCTAGGAAACAAAATAGAACTAAAGGATACCTTGGCAGAAGAGAGAGTG
AAGTTTTGGGATGGCATCTACGAGAAATATTACCGTATACCCCGCCCGCCATCCCCACTTCCACCAAAACACACAGAATTATAA

>NM_001135201.1 [cathepsin L like protein precursor] ID:NP_001128673.1

ATGAATCGGTTTTGGTTGTTAATATTCGGCCTGCCCTTCGTGGCGGCAGCGACAATAGACTTGAAAGACGAACCTCTAGATTGGCCTGGAACATATCAT
TTCGAAGCCGTCAGGATGTCAATTAGTGCCGGCAACGTACAAGACTACTCGGTATGGAAAACAAATCATTCGTCACGCGTCGACTACAACAAAGGAGCT
GTGAAAAGTATTATCGTAGATGAAAACGCAAAATACAGATACGGAGTCTCATATGAGATCCACCCAGAAACTGACGATGGTGAAGATGCGATTTTCAAG
TGTAATGTAATGCTTGGATCTGAAGATAATATATTCGACTTAAAGATTATCTTGCCAGAAACCGACAACTTTGAATACGTCGGACCAGATTCACAAACC
GTTGAAAACACTATCAAGTTCGTCGCTGAAGATAGCGATTTGGAGACTAAGACAATAAAGACTATTTGGGCTACCTATGACGAGAAAAACAATAATTGG
CATCCAGTCAGATATGAAGTCAAAAACTACAATGAACTATTAGGACTTTTAGAGAAACACGAAATTTGGGATTACTTTAATTTCAACACCGGATTTGAC
AAATCAGCTTTCGACGTCAGCCAGTATGACTGCGACGACGAAAATGTCGAGGAGCATACATTACAGAGTGAAACTGTAACTAAATTTCTCATGTTCATG
GATCCCGAAAATGATAAACATGTTGATCACGTATTTAACAGTTTCAAAAACAAATTCGTCAGAAACTACGCTGATAACAATGAACATGAAACTAGAAGA
AACATTTTCAGGAAGAACATGAGACTAATCACGGAAACAAACCGTCAAAAACTCGGTTACACACTAGGGGTCACTCAGTTTGCGGACCGCACTCCCGAA
GAAATGCAAAGACACAAGGGCCTGCGTACACGTCCCGAAGGAAAGGTCGGAAATATTCCCTTCCCATATCCTGAACACAAAATACGAGATATTGCCGAA
GACCTTCCCTCAGAATACGATTTGAGAATCTTGGGCTACGTTTCTAAAGTCAAAAATCAAGAAGATTGTGGATCCTGCTGGACATTCGGTACCACTGCA
GCTGCTGAAGGAGCCCTTGCTCGAATCAACGGAGGAAGACTGTTGAGTCTATCTAACCAAGCAATCCTGGACTGCGCCTGGCCTTACGGCGGCAGTGGG
TGCGAGGGTGGATCCGACAATGCAGCGTATGATTGGATGATGAAATTCGGTTTACCAACCGAAGAAGAATATGGATCTTACACAAATGCGGACGGTATC
TGCAATATTAAAAATATGAGCACCATTTACCCGATTCGCGGTTGGACTGACGTCACGCCACTTAACGTTGAGGCTTTGAAGGTAGCTGCCGTGAACCAT
GGTCCGCTTTCAGTATCGATTGATGCTACTGATAAATTTTCGCTCTACACCGGTGGCATATTCTATGACACCACCTGCACAACTAAACGCCTGAACCAT
GAGATGGCGTTAGTCGGCTACGGTGAACGCGACGGCGACACCTATTGGATCATCAAAAACTCCTGGGGCCCCGACTGGGGAGTCGAAGGATACTTGCTG
ATTTCATCCCGTAACAACAACTGCGGTATCGCAACAGAACCAACTTACGTAGTCTACTAA

>XM_004928816.1 [glucose dehydrogenase [acceptor]-like] ID:XP_004928873.1]

ATGATATTAGCTCCAGAGGAGTGCGGCTGTCCGCTTATTGAAGAAGGAGTCTCTATAGAAAACTCGCCGATTTGCAGTGGAACTCTGCTATTTATGGTA
TTACTCGAAGGGTACATCCGAGGTCGTTGCAAAATCGCAACTCCATGCGCTAATGTGAGATCCGCTGAACAACTGGATGCCCGTTACGATTTTATTGTG
GTGGGCTCAGGTCCGGCCGGAGCCATTGTTGCTGGAAGACTCAGCGAAGATAAGAATTTTAATGTACTATTACTGGAAGCCGGAGGTCAAGAGCCTACG
GGAGCACGCATTCCCTCTTTCTACAGAGCATTCTGGTCGAACAAAGAGGTGGATTGGGATTATAGAACTGAGCCCGACAACTATTGTCTCGACCAAGGC
GATAAAGGATGTCTTTGGCCACGAGGAAAGGTTCTTGGTGGATCAAGTCTCCTTAATGGTATGATGTACCATCGAGGCCATGCCGCTGATTACGATGAC
TGGGTGAAGCTCGGTGCTGAAGGTTGGTCATGGGAGGAAAATCTTCCATACTTTGACATGACCGAGGGGAACAAGGAAATTGGAACTATAGTGAGCGAG
AAACACCACTCTAGTTCAGGGCCTTTGCCTGTTCAAAGATTCCGTTACCAAGGTCCAGCCGTTTATAAACTATTGGATGCCCTCAACGAAACTGGGTTC

TCTATCATAGCTGACATGAACGATCCTGAAACTCCCGATGGATTCACCATAGCACAAGCGTTTAACGATAACGGCCAGAGGTACACAACAGCGCGCGCG
TACCTGAAACCGAAGTCAGAACGACCGAACCTGACAGTGAAACTGGGAGCGCACGTTACAAGAGTCATAGTGACTGACGACACAGCCACCGGCGTTGAG
TTCATCGACTCCGACGGAAATTCAAATATCGTTTATGCTTCTCGAGAGGTGATTTTAAGTGCTGGAGCGTTAAACACTCCTCACATTCTTTTGCATTCC
GGCATTGGACCCCGAGAGACGCTAGAGAAGTACAACATTCCAGTCAAAGCAGATCTCCCAGTGGGTTTGAATCTTCAGAATCACGTTGGTGTCACTGTA
TCGTTTATACTGCCTAAACTCAACGACACACGAGTCTTGGATTGGAGCACGCTTGCCACATACTTGTTGAATCAGGAGGGGCCTATGACGTCCACCTCA
ATTACTCAGGTAACAGGATTGTTGTATTCAAGTTTGGCGGACAAAAGAAAAAAGCAACCTGACCTTCAATTCTTCTTTAATGGCATGTATGCAGAATGC
TCCAAGACGGGCTTCGTGGGAGAGACTATTGACAGTGACTGCCAAGAAAGAGGAACAAATATTACAGCCAATGCAGTGGCGCTCCTGCCAAAAAGCAAG
GGATACTTAACGTTGCAGTCTTCAGACCCACTAGTTTCTCCATTATTTTATCCGAATTTCTTCTCACATCCTGATGATATGATCGTGGCCAAAGACGGA
CTCAGATATTTGAAAAAAATATCTGAAAGCAAGATCCTAGAATCAGAATATGGCATAGAACTAGATCCCGAAGCGACTGATGAGTGTAGTGAGACCTCA
GAAGACTGGTCAGATGACTGGATGGAGTGCATGATCCGATTGCACACTGATGCCCAGAATCATCAACTCGGCACAACCGCTATCGGCTTGGTCGTCGAT
CCTCAACTAAAAGTCTACGGAATTGAAGGTTTGAGAGTTATCGATGCATCGGTCATGCCGTCACAGCCGACGGGCAATCCTCAAGCAGCGATCATGATG
GTCGCCGAGCGAGGCGCGGCTTTCATCAAAGACACTCATTCCTAA

>XM_004932354.1 [protein disulfide-isomerase A6-like] ID:XP_004932411.1

ATGTTACACGGATATTTTATAGGTATCTTGCTCTGTGCAACGGGGTCCTTGGCCCTCTACGATTCGTCTTCAGACGTTATCGAGCTGACACCGAGTAAT
TTCGACAAATTAGTTACAAATTCCGACGAAATTTGGATCATTGAGTTCTTTGCACCGTGGTGTGGACATTGTAAAAGCCTTGTTCCCGAATATAAAAAA
GCTGCGAGAGCTCTAAAGGGCATTGTAAAAGTAGGTGCCTTAGATGCTGATGAACATAGAAGTGTATCACAGAAATATGGAGTCACTGGCTTCCCCACA
ATTAAGATATTCACAGGGAGCAAGCACACACCATATCAAGGTCAGAGGACAGCAGAAGGTTTTGTTGAAGCTGCTTTAAAGGCAGCCAAGGAGAAGGCA
TATGAAAATCTCGGCAAGAAATCGAGTGGATCCTCTTCTGATAAGTCTGATGTCATTACTCTAACAGACAGCAACTTCAAAGAACTGGTCTTAGACAGC
GATGACCTCTGGCTGGTTGAGTTCTATGCTCCTTGGTGTGGGCACTGCAAGAATCTTGAACCTCACTGGGCTAAAGCTGCCACCGAACTTAAAGGAAAG
GTCAAGCTGGGTGCCCTGGATGCAACAGTTCACACAACAATGGCGTCACGCTACCAAGTACAAGGATACCCGACCATCAAACTATTCCCATCAGGGAAG
AAGTCTAGCGATTCTGCAGAAGACTACAATGGAGGCAGGACGTCAAGCGACATCGTTACTTGGGCTCTTGAGAAGCTCGCTGAGAATGTTCCCGCACCA
GATATCATACAGGTTGTTGGTGAAGAAACTTTGAAGGCTTGCAGCGAGAAGCCTCTATGTGTTGTATCAATTCTGCCTCACATCTTAGACTGCAATGCC
GCATGTAGGAATGACTATATCTCCATCCTCAAACGTCTTGGTGACAAATACAAGAACAAGATGTGGGGGTGGATTTGGGCAGAAGCTGGTGCACAACCA
GCACTTGAAGATTCTCTGGAGCTTGGCGGATTTGGTTACCCTGCCATGGCAGTTGTCAATGCTAAAAAACTCAAGTTCTCTACATTGAGAGGTTCTTTC
TCTGAAACTGGCATCAATGAGTTCCTCAGAGATTTGTCGTTCGGTCGTGGCCAAACAGCACCAGTGAAAGGAGCAGAGATGCCCAAAGCCGTGACCACA
GAGCCGTGGGACGGCAAAGACGGCGAACTTCCATTAGAAGAGGACATTGACCTTTCAGACATTGATTTAGAGAAGGATGAATTATAA

>EF535809.1 [cytochrome P450 CYP6AE9] ID:ABQ08711.1

ATGTTCTTACTGATTAATATTTGTGTTATTTTATTTGTGATATACTATTTAGTGACCAAAAAGTATAGTTATTGGAGAAACAGAAATGTTTCTCACGAA
AAACCAGTGCTTCTTTTGGGAAATTACGGTGACTTAATCCTGCAAAAAAAGAATTTTGGTGAGATGGCCCGAGCGATTTGTCAGAAGTTCCCTGGGGAG

CCTGTTGTTGGAGCTTTTTTTGGAACAGAACCGGTTCTTATACCTCAGGACCCTGAAGTCATCAAAACCATATTAACCAAAGATTTCTACTATTTTAAT
GGAAGAGAAATATCAGAGCATGTTCACAAGGAACTCTTGTCGTACAACTTATTTGCCACGTATAGAGACGAATGGAAAATATTGAGACAAAACCTAACT
CCAATTTTTCTACTGCAAAACTGAAAAGTATGTTTACTCTAATTGAGAAATGTTCAAAATCTTTTCAAAATCTACTGGAAGATGAAACGAAAATATCG
AAAGAGCTAGAAGTGAGAACTTTGATGCAGAGGTTTACAATAGAATGTATCGGATCGTGTATTTTTGGAGTAGATACTGACACATTGGGAAATGATAAA
ATGAATCCATTTAAAGCAGCAGGCAGCCAATTGTCAGACTTTTCGAGGCTTGTTTTTGTTAAAGGGATAGTAAGAGCAATTTGGCCCACTTTATTTTAC
GCCCTTGGATTTAAAACGTTTACTACTGAATTAGATATTTTCAAAAAACTGGTGAATGCTGTTTTTGCTCAAAGAAAACACAAGCCAACAACAAGGAAT
GATTTTGTTGACTTAATTTTGACATGGAAGAATAACAATACCATTACCGGAGACAGCATAGGAAGTTTTAAAAATAGCGATAAAACAAAATTTTCAATT
GATGTTAATGACGATTTACTTTTAGCTCAGTGCTTAGTATTTTTTGCTGCGGGCTTTGAAACTTCTGCAATGACGTCGAGTTACACTTTACACGAGCTA
GCGAAAAATCAAAGAGCTTTAAAGAAAGCCTGCGATGAGGTCGACGCTTATTTATTGCGTCACGGTAACAAGATCAACTACGATTGTGTCACGGAATTG
CCTTATCTCGAGGCATGCATTGAAGAGACTCTTCGTCTATATCCAGTTCTTGGTATAATAACTAGAGAAGTGATGGAAGACTACGTTTTGCTTGATAAA
ATACATTTAAAGAAAGGAGATCGTATACATGTACCGGTATTTCATCTGCATCATAATCCTGAACATTTTCCGAACCCGGAAGAGTATCGACCGGAGCGA
TTTTATGGTGAAGAAAAACGTAAGGTGAAGCCGTATACCTATTTACCATTTGGTGAAGGCCCACGAATTTGCATTGGGATGAGATTTGCAAAAATGCAG
TCTATAGCAGGCTTGATAACGATTTTGAAGAAATTCAGGCTGGAACTACCCGAAGGTGCACCTACTAAGATTGAGTTCAAGCCTGAAGCCTTTGTGACT
ACACCTAAAGATCTAATCAAAATCAAATTTTTGGAACGTGAAGGATGGCAGCAAAGAGTTTTTGTTTAA

>XM_004929226.1 [uncharacterized protein LOC101740647] ID:XP_004929283.1

ATGGCGCGCTACGCCCGTGTCGCTACTCTGGCCGCGTGCCTCTTGTTCGCCTGCGCGCTTGCTGATGGTCACCGATGGCGGCGACAAGCTGACGAGACG
GCAAAGAAAGATGAAAGCTTGGAACAAGAACTATGCAAGGACAAGGACGCCGGCGAATGGTTCCGGCTGGTGGCCGGCGAGGGCGACAACTGTCGCGAC
GTCATCCAGTGTACTGCCTCGGGAATACAAGCTATACGTTGCCCGGCCGGTTTGTTCTTCGATATTGAGAAACAGACCTGCGATTGGAAAGATGCTGTG
AAAAATTGTAAGCTCAAGAACAAGGAGCGTAAAATAAAGCCTCTCTTGTACACTGAAGAACCACTCTGTCAAGACGGCTTCCTCGCTTGCGGCGACTCT
ACTTGCATTGAACGCGGTCTTTTCTGTAACGGCGAAAAGGACTGTGGCGATGGATCTGATGAAAATTCTTGTGATATTGACAACGACCCAAATAGAGCT
CCGCCATGCGATTCCTCGCAGTGTGTCCTTCCTGACTGCTTCTGCTCCGAGGACGGCACAGTGATCCCCGGCGACTTACCCGCCAGAGATGTCCCTCAA
ATGATCACTATCACCTTCGATGATGCGATTAACAATAACAACATTGAATTGTACAAAGAGATATTTAACGGCAAGCGCAAAAACCCAAACGGTTGCGAT
ATCAAAGCCACTTATTTTGTCTCGCACAAGTACACTAACTATTCTGCCGTCCAAGAAACTCACCGCAAGGGTCACGAAATCGCAGTTCATTCTATTACG
CACAATGACGATGAACGTTTTTGGAGCAACGCTACTGTCGATGATTGGGGCAAGGAAATGGCCGGTATGAGAGTTATTATTGAAAAATTCTCTAACATT
ACTGACAACAGCGTTGTAGGAGTTCGCGCACCTTATCTACGTGTAGGTGGCAATAATCAGTTCACCATGATGGAAGAACAGGCATTCTTATACGACAGC
ACTATTACAGCACCGTTATCAAACCCCCGTCTTTGCCCTTACACCATGTACTTTAGGATGCCCCACCGATGCCACGGAAACCTTCAGAGTTGCCCAACC
AGGAGCCACGCAGTATGGGAAATGGTAATGAACGAGCTTGACAGACGTGAGGACCCAAGCAACGACGAATATTTACCCGGATGTGCCATGGTAGATTCT
TGCTCTAACATTCTTACCGGTGATCAATTTTATAACTTCCTCAACCACAACTTTGACCGTCATTACGAACAGAACCGAGCACCTTTAGGACTTTATTTC
CATGCAGCTTGGTTGAAAAACAACCCTGAATTTTTGGAAGCTTTCTTGTATTGGATAGACGAGATCCTTCAAAGCCACAACGACGTTTACTTCGTTACC
ATGACACAAGTGATCCAATGGGTACAAAACCCACGTACCGTGACTGAAGCCAAGAACTTCGAGCCGTGGAGAGAAAAGTGCTCCGTCGAAGGAAACCCT
GCATGCTGGGTACCTCATTCCTGCAAGCTCACTTCAAAGGAAGTTCCCGGAGAAACCATCAATTTACAAACTTGTTTGAGATGCCCAGTCAACTACCCC
TGGTTAAACGACCCCACGGGCGATGGCCACTATTAA

>AB262581.1 [glyceraldehyde-3-phosphate dehydrogenase] ID:BAE96011.1]

ATGTCAAAAATTGGAATCAATGGATTTGGCCGCATTGGCCGTTTGGTGCTCCGTGCTTCTATTGAAAAGGGAGCTCAAGTGGTCGCTATAAATGACCCT
TTCATCGGTCTTGACTATATGGTTTATCTTTTCAAGTATGATTCCACCCATGGCCGTTTTAAGGGCAGTGTTGAGGTTCAGGATGGATTCCTTGTTGTT
AACGGTAACAAAATTGCCGTTTTCTCAGAAAGGGACCCTAAGGCCATTCCATGGGGAAAAGCTGGGGCTGAATATGTTGTAGAGTCTACTGGTGTCTTT
ACCACTACAGATAAAGCATCTGCTCACTTGGAGGGAGGTGCTAAAAAAGTTATTATATCAGCTCCCAGTGCTGATGCCCCCATGTTTGTTGTGGGTGTT
AACCTAGAAGCTTATGACCCCTCTTTTAAGGTCATCTCAAATGCTTCTTGCACCACAAACTGTCTTGCCCCACTTGCAAAGGTTATTCATGATAACTTT
GAAATTGTTGAGGGCTTGATGACTACTGTTCATGCCACAACTGCTACACAGAAAACTGTTGATGGACCTTCTGGAAAATTATGGCGTGATGGCCGTGGT
GCTCAACAAAACATCATTCCTGCCTCTACTGGTGCTGCCAAAGCTGTGGGTAAGGTTATCCCTGCTCTTAATGGCAAGCTGACTGGAATGGCATTCCGC
GTCCCTGTTGCTAATGTATCTGTTGTTGATCTAACTGTTCGTCTTGGAAAACCTGCAAGCTATGAAGCCATCAAGCAAAAGGTCAAGGAGGCAGCTGAA
GGTCCTTTGAAGGGCATTCTCGGGTATACTGAAGATCAAGTTGTGTCCTCAGACTTCATTGGTGATTCACACTCTTCAATCTTTGATGCTGCCGCTGGA
ATTTCTTTGAATGACAACTTTGTGAAGCTGATCAGTTGGTACGACAATGAATATGGTTATTCCAGCAGAGTCATTGATCTCATCAAGTACATTCAATCT
AAAGATTAA

>NM_001043999.2 [fibroinase precursor] ID:NP_001037464.2

ATGAAGTGTTTAGTATTGCTGCTATGCGCAGTGGCTGCTGTGAGTGCTGTTCAGTTCTTTGACCTGGTCAAGGAAGAGTGGAGTGCCTTCAAGCTGCAG
CACCGTCTCAACTACGAAAGCGAGGTCGAAGACAATTTCCGCATGAAGATATACGCTGAGCACAAGCACATCATCGCCAAACACAACCAGAAGTACGAA
ATGGGCCTCGTTTCTTACAAGCTGGGCATGAACAAGTACGGAGACATGCTCCACCACGAGTTCGTGAAGACTATGAACGGCTTCAACAAAACTGCCAAA
CACAACAAGAATCTGTACATGAAGGGTGGGAGCGTCCGCGGGGCTAAGTTCATATCGCCGGCCAACGTGAAGCTGCCGGAGCAGGTGGACTGGAGGAAG
CACGGCGCCGTCACCGACATCAAGGACCAAGGGAAGTGTGGCTCATGCTGGTCCTTCAGCACGACTGGAGCTTTGGAAGGACAGCACTTCCGTCAGTCC
GGCTACCTGGTGTCGCTCTCGGAGCAAAACCTCATCGACTGCTCGGAGCAGTACGGGAACAACGGCTGCAACGGGGGGCTCATGGACAACGCCTTCAAG
TACATCAAGGACAACGGGGGCATCGACACCGAGCAGACCTACCCCTACGAGGGAGTTGACGACAAGTGCAGGTACAATCCCAAGAACACCGGTGCTGAG
GACGTGGGCTTCGTGGACATCCCCGAGGGCGACGAACAGAAGCTGATGGAAGCCGTGGCCACCGTGGGGCCCGTCTCCGTGGCCATCGACGCCTCGCAC
ACCAGCTTCCAGCTCTACTCCAGCGGAGTCTACAACGAGGAGGAGTGCTCCTCCACTGACCTGGACCACGGGGTTCTGGTGGTGGGTTACGGCACCGAC
GAGCAGGGCGTGGACTACTGGCTCGTGAAGAACTCGTGGGGCCGCTCGTGGGGCGAGCTGGGCTACATCAAGATGATCCGGAACAAGAACAACCGCTGC
GGCATCGCCTCCTCCGCCTCCTACCCCCTCGTGTAA

>NM_001195699.1 [cryptochrome 1] ID:NP_001182628.1

ATGCTTGGCGGTAGCGTTCTGTGGTTCCGTCACGGGCTGCGTCTCCACGACAATCCGTCTCTTCATTCGGCGTTGGAAGAAACGAGCGGCCCTTTCTTT
CCCATTTTCATTTTTGACGGGGAAACTGCTGGAACGAAAGTGGTCGGGTACAACCGAATGCGGTACTTGCTAGAAGCGTTAGATGATTTGGACAAGCAA

TTCAAGAAGTACGGCGGAAGATTACTCCTTGTTAAAGGGAAACCCAGTGCTGTGTTCCGCAGACTCTGGGAAGAATTTGGTATTCGGAAGTTGTGCTTC
GAACAGGACTGCGAGCCGGTGTGGCGTCCCAGGGACGAGAGCGTGAAGACGGCGTGCCGCGAGATCGGGGTCACGTGTCGGGAGCACGTGTCGCACACC
CTGTGGGAGCCCGACACCGTCATCAAAGCGAACGGGGGAATACCGCCGCTCACGTACCAAATGTTTCTGCATACTGTGGCGACCATCGGTGACCCGCCG
CGGCCTGTCGACAACGCCAAACTCCGCGGTATTAAGTTCGGTACATTGCCGCTGTGTTTCTATGAAGAATTCACTGTTTACGACAAGGTGCCGAATCCC
GAAGACCTCGGAGTGTTTCTCGAAAACGAAGACATCCGGATGATACGTTGGGTCGGTGGCGAGACGGCCGCCTTGAAGCAGATGCAACATCGACTCGCC
GTCGAATACGAGACGTTCTGCAGAGGTTCCTATCTACCGACGCACGGCAGCCCCGACCTCCTCGGGCCTCCGATATCGCTGAGTCCCGCTCTACGGTTC
GGATGCCTCTCCGTTAGGAAATTTTACTGGTCGCTCCAGGATTTGTTCCAGCAAGTGCACCAGGGAAGCTTGTGTTCTACTCAATACATTACCGGTCAG
TTGATATGGCGCGAGTACTTCTACACGATGAGTGTGAACAATCCTCATTACGGACAGATGACTGACAACCCTATTTGCTTGGACATCCCCTGGAAAAGC
CCTGAGGGAGATGAACTGGAGAGATGGGCGTCGGGGCGCACGGGGTTCCCGTTCGTGGACGCGGCCATGCGGCAGCTCCGGCTGGAGGGCTGGCTGCAC
CACGCCGTGCGGAACACCGTCGCCTCCTTCCTCACGCGCGGCACTCTGTGGCTCTCCTGGGAGCACGGGCTCGCGCACTTCCTCAAGTACCTGCTCGAC
GCCGACTGGTCCGTGTGCGCCGGCAACTGGATGTGGGTGTCCAGTAGCGCCTTCGAAGCGTTGCTGGACTCGGGCGAGTGCGCGTGTCCAGTGCGACTG
GGCCAGCGGCTCGACCCCAGCGGCGAGTACGTGCGGCGCTACGTGCCCGAGCTGGCGCGGGTGCCGACCGAATACATCTACGAGCCCTGGAAAGCGCCT
CTGGACGTGCAAGAACGTGCCAACTGTATAATAGGCAAGGACTATCCCGCGCCTGTGGTCAATCATATCGTAGCTGCGCAGCGCAACAGAAACGCCATG
GAGGACACAAAGATGGAGAAAAATCATGCGAAACAAAGAGGACGACCCTTGGCATCGAGCAATTCCGGGCGTAGATGGAATAGCCCCTTAGACTACCAG
CAAACAGGTAGGGAAAAAATATATTGA

>NM_001047080.1 [beta-1,3-galactosyltransferase] ID:NP_001040545.1

ATGAGGAGAAACAGATATAAATTGATTGTATTCGCTTTGGTACTTGTATATGTTTACCATTTTTTCGGTGTCGGCGATTACGTGCAGTCCAAGAACTTC
GATAGCGATTTCAATTATCCTCTCAACGTAGACATTCGACCGATAGTCCAAGCAATACTAGACGGGCAAAAGCCGAATGTTAAACCTATAAATTACTAC
CCGTACAAGTTCTTGAGCAACTATCGTCAATGCAGTGTCGTCAACAAACCGGATTTAGTCATAATAGTCAAGTCGGCTATAGACCATTTCGGACACAGA
GATGCAATTAGAAAGACTTATGGAAAACCACATGTCCAGGGCTACAACGTGAAGACATTCTTCTTTTTGGGCGTGGACAATGCCAGTTCAGATGTACAG
AAGAATATTACGAAGGAAATGACAGAATTCAAAGATATCATACAGATGTCTTTCCGTGACAGTTATTTCAACAACACCATTAAAACGGTCATGTCCTTT
CGCTGGATCTTTCAGCACTGCGCTGAAGCTCAACACTACTTATTCACCGACGACGACATGTACATATCGGTACAGAATTTACTAAAATACGTTAGCGAC
GTGACGACCGCGAGCGAAAGAGACGGAATACTGTTCGCCGGTTACGTCTTCAAGTCGGCGCCGCAACGGTTCCGGTCGAGCAAGTGGCGGGTGTCCTTG
GAGGAGTATCCCTGGGACAAGTGGCCGCCTTACGTGACGGCCGGGGCGTACGTGGTGTCAAACAAAGCGATGAAGATGCTCTACGTCGGAAGCTTGTTC
GTGAAGCATTTCAGATTCGATGACATTTACCTCGGCATAGTCGCGAAGAAGATGGGCATAGTGCCGACTCATTGTCCGCATTTCCACTTCTACAAGAAG
CCGTACGAGCGAGAGGTTTATAGCGATGTGATCGCCTCGCACGGATATTCTAATCACGACGAATTGATTAGAGTGTGGAATGAACAAAACGCGTTGTGA

>NM_001098327.1 [eukaryotic initiation factor 5C]
ATGAGTCAGAAGGTAGAAAAACCAGTATTATCGGGTCAACGGATCAAGACCAGAAAAAGAGATGAGAAAGAGAAGTATGACCCGAACGGTTTCCGCGAC
GCTCTGGTACAGGGTCTGGAGCGGGCCGGTGGCGATCTCGACGCAGCCTACAAGTACCTAGACTCGGCCGGATCAAAACTCGACTACCGACGCTATGGC
GAAGTCATATTCGATGTACTCATTGCTGGCGGCCTGCTGCTGCCGGGCGGTTCGGTGTCGATGGACGGCGAATCGCCCAAGACCAACACCTGCATCTTC

TCCGCCAATGAGGATATGGACACCATGCGAAATTTCGAACAGGTATTCGTGAAACTGATGCGTCGGTACAAGTACTTGGAAAAAATGTTCGAAGAGGAA
ATGAAAAAGGTTCTGGTCTACTTGAAAGGCTTCGATCCCGAACAGCGCATCAAGCTGGCGCGCATGACTGCACTGTGGATCGGTAACGGATGCGTGCCT
CCATCCGTGCTGCTGGTCCTGGTGAACGAACATCTGCTGAAGGACAACCTGGCGCTGGACTTCGTGCTGGAAGTGTTCGCGATCATCAAGCAGGAGCGC
GGCGTCACCTCGCTCGTCACGGCGCTCAAGAAGGGACAGCTTGAAGGCAGACTGCTGGAGTTCCTCCCGCTTAATCGCCGCAGCGAGGAGGCTCTGGGC
GCTGCTTTCTCTTCTCGCGGACTTGCCGAGCTGCTGCGACTACATCGGGCACAAGCGTCGCAGGAGGCTCGCCGTGAACTGACGGCGTCCCTGCTGGAG
CAGCTCGCCGAGGAGAAGGTGATCCGTGACGTCATCACCGACCTGCGAGAGATGGCGCAGAGGCACCACATTCCCGATCACGACGTCGTCACCATCGTG
TGGCAGTGCGTGATGTCGCGCGGCGAGTGGAACAAGAAGGAGGAGCTCATCGCCGAGCAGGCGGCCAAGCACCTGCGCCACTACACGCCGCTGCTCGCC
GCCTTCGCGCAGTCCGCGCGCGCAGAGCTCGCCCTGCTCACTAAGGTCCAGGAGTACTGCTACGAGAACATGAGCTTCATGCGCGCGTTCAGTAAGCTG
GTGGTAATGCTGTACAAGACGAGCGTCCTCTCGGAGGAGGTGATCCTCAAGTGGTACAAAGACACTAGTTCTTCCAAGGGCAAGGTCATGTTCCTTGAC
CAGATGAAGAAGTTTGTAGACTGGCTCCAGAATGCTGAAGAGGAGTCTGAGAGCGGAGAAGAAGAAGATTAG

>XM_004927754.1[NADH-cytochrome b5 reductase 2-like isoform X2]ID:XP_004927811.1

ATGAGTGAAACGGGAAGTGCGGTACTACCAATAATATTTGGTGTTGGATCAGTGGTAGTCTTAGCAACTATAATAGCCAATTGTCTATGGGGAAAGAAA
TCTAAAGGATCTAGCAAAAAGTTAATAACATTAGTAGATTCTAACGTAAAATATGCATTACCGCTGATAGAAAGGGAAGAAATCAGTCATGACACCAGG
AGATTCAGGCTGGGACTGCCGTCTCCTCAACATGTATTGGGATTACCGATCGGTCAACATATTCATTTGTCTGCAAAAATTAACGACGACCTAGTCATA
AGAGCATACACGCCGGTGTCGAGCGATGAGGATAAAGGCTATGTCGATTTGGTTATCAAGGTATACTTTAAGAATGTACATCCGAAGTTTCCCGAGGGA
GGGAAGCTGTCTCAGTACTTGAACAATATGAAGATAAATGATACTATAGATGTCCGTGGACCGTCAGGGAGATTGCAGTACACAGGAAACGGAACATTC
CTCATTAAGAAATTAAGAAAAGATCCGCCTACAAAAGTTGTCGTCAAGAAATTGAACTTGATTGCAGGCGGTACTGGCATAGCTCCAATGCTTCAGCTG
GTCCGACATATATGCACTGACGTCAACGACCGCACCGAACTGAAGCTGCTATTCGCGAACCAAAGCGAAGATGACATTCTACTTAGAGATGAATTAGAA
AGATACCAAAGGGAACATCCTTCCCAATTCCAAGTTTGGTACACGATTGATCGGCCAACGGATGGTTGGAAATACAGCTCCGGTTTCATCAACGATGAA
ATGATCCGCGACCATTTGTTTCCGCCGTCCAACGATGTTCTAGTACTGATGTGCGGTCCACCGCCGATGATCAACTTCGCTTGCAACCCAGCTTTAGAT
AAATTAGGCTTTAAGCCAGACCAACGCTTTGCTTATTAA

>NM_001083610.1 [cytochrome P450 9a20] ID:NP_001077079.1

ATGATTCTCCTCATTTGGGCCGTCGTGCTCATTGCCGCCTTCGTGCTGTTTTACAAACAAGCCTATTCTTTGTTCAGCAAGCACGGAGTGAAGGGCTTC
ACTCCGCTGCCTTTCTTTGGCAACATGGGGCGTATTGTAATAAAAATGGATCATTTCTCGGACCATATACAAAGTTTATACGATTCATTCCCAGAGGAA
AGGTTCGTCGGAAGATACGAATTTCTAAACCCAATGGTTATAATTCGTGACATTGAGTTACTGAAGAAAATCACGGTTAAGGACTTCGAGCATTTCCTG
GACCATCGGACTATCATCAACAAGGACACCGACCCGTTCTTCGGAAGGAGCTTGTTCTTCTTGAGAGATCAAGACTGGAAAGACATGCGCTCAACGCTT
TCGCCGGCGTTCACCAGTTCCAAAATGAAGCTCATGATGCCCTTCATCGTTGAAGTGGGAGAACAGATGAATAAAGCGCTAAAGCAGAGGATACAAGAA
GCAGGGGTTGGCTATGTGGACATCGACTCCAAAGACCTGACCACTAGATACGCCAACGATGTGATCGCGTCCTGCGCCTTCGGTCTGAAGGTCGACTCC
ATCACCGAGGAGAACAACCAGTTCTACGCGATGGGGAAAGCCGCTTCCACTTTCAACTTCAGGCAGCTTCTAATTTTCTTTGGTCTCGCATCTGTCCCG

AAGCTTGTGAAGATTCTACGCATTACGCTGTTTCAAAAAGAAATCAAAACCTTCTTCAGGGAGCTGATCTTGGGTACCATGAAGAACAGGGAAGCACAA
AACATTATCAGACCTGACATGATCCATCTACTCATGGAAGCTAAGAAAGGCAAGTTGAGGCACGATGAGAAATCCACAAAAGACAGCGACGCTGGCTTT
GCTACCGTGGAAGAGTCCTCAGTCGGCAAAAAGGATATTAACCGAGTGTGGACAGACGATGACTTGGTCGCCCAAGCGGTTCTGTTTTTCGTCGCCGGC
TTCGAAACAGTATCGTCAGCGATGACATTCCTGCTTCACGAGTTGGCTTTGAACCCTGAAGTGCAGGAGAAGCTGGTGGAAGAAATCCGAGAAAACGAG
AAAAACAACAACGGAAAATTCGACTACAACTCCATTCAGAACATGGTGTATTTAGATATGGTGGTGTCAGAGGTCTTAAGATTGTGGCCACCTGTTATT
GCCTTAGATAGAATGTGCGTTAAAGACTACAACTTGGGTAAACCAAATGACAAAAGCAAGGAAGATTTTATTATACGCAAAGATGTGGCGGTGGGTATA
CCAGTATGGGGCTTACATCGAGATCCCGAATTCTTCCCGAACCCCCTGAAATTCGATCCCGAAAGGTTCTCAGAGGAGAACAAACACAACATCAAACCC
TTCAGTTACATGCCATTTGGTCTGGGACCTAGGAATTGTATAGGTTCAAGGTTCGCTTTGTGCGAAGTCAAGGTGATGACTTACCAACTCCTCCAGCAC
ATGGAGATATCTCCGTGCGAAAAGACCTGCATTCCCTCGAAACTCAGCAAGGAGACTTTCAACCTTCGCCTTGAAGGCGGTCATTGGATCAGGCTGAAG
ATTAGAAATTGA

>NM_001043468.1 [molting fluid carboxypeptidase A precursor] ID:NP_001036933.1

ATGGGATCCAAGAGGCAAACGGTCGTGTTGTTCTTGGCGGCCCTAACCGTCGTTCTCAGCTCACCTCTGATCAATGAACTACAGCCCGGAGAAGAATGG
CCACAACGCAATTCGGTAAGACAACCGGTAGCTGATGACCAAACAGAACAGACACTGACAGAGGACTTCTCAACTGACGATCCGGCCCCAGCCGCAGTT
GTTGAAGATGCAATAATAGTAGCGAAAACAATTGAACGCGATCATGAGCCGAAAAAAATTGATTACTCCGGTTCACAAGTGTGGCAAGTTTCCACTACA
AAATCTGGAGCTCGTCAAGTAATCGGAAGACTTCGACGAAGAAACCTGATTTCTACATGGGGCGGGAATCAGTCTTCTGTGGACATCCTTATCAAACCG
AATGTCGTAGAAAATATTACACGTGTTTTCAAAAGAGAGAGCATAGACTACAACGTCGTGATAGAAGATTTACAAAAGAGGATCAATGAGGAAAATCCG
CCGCTAGATAACGATGAAATTGAACTGCAAGACAGACGAGGTCACCGAATGACATGGAAACAGTATCACAGATTAGAAGACATTTACGGCTTTATGGAT
TACTTGGCAAAAACCTATCCTTCTATCATCAGTGTGAAATCAATAGGAAAATCATTTGAAGGTCGAGATCTTAAGATACTCCGCATATCAAACGGTAAA
TCAGACAACAAAGCCGTGTTCATCGACGGCGGTATCCACGCTCGTGAATGGATAAGTCCCGCAACCGTCACTTACTTTATCTACCAGTTTGCTGAGTAT
TTCGATGTGGAATCTGACGATATTAAGGGCATCGACTGGTATTTCATGCCGGTCGTGAATCCAGATGGATACGAGTATACACATACCGTGGATCGTCTT
TGGAGGAAGAACAGAAAGCCTGGTTACCGTGCTTGCACTGGTACTGATCTCAACAGAAACTTTGGACATCATTGGGGAGGCAAAGGCGCATCAAACAGT
CCGTGCAGTGAAATATATCGTGGAAGTAATGCCTTTTCAGAACCAGAAACGTTAGCTTTATCTGAATTTATAAAATCAAGCGCGGCTAACTTCTCCGCA
TATGTTACATACCATAGCTATGGTCAATACATGCTTTATCCATGGGGATATGATAATGCAGTGCCTCCAGACTACAAGGATTTAGATAATCTTGGAAAA
AAGATGGCAGAGGCAATCTCAAAGACCGGTGGTTCTCAATATCAAGTCGGTTCATCTAGTGGTCTCCTTTACCCTGCAGCAGGTGGCTCAGATGACTGG
GCGAAATCACAAGGAATCAAATACTCTTACACTATTGAACTTAGTGACACAGGACGTTATGGTTTTGTTCTACCTACATCGTTCATTGTACCCGTTGCT
AAGGAAAATTTAGCTGGCTTGAGGGTTCTTGCTAGCCAAGTTATCAAGGAATAA

>NM_001043375.1 [beta-1,3-glucan-binding protein precursor]ID:NP_001036840.1

ATGTACAAAACATGTGTGTGGGTCTTGTTATTTAAAATCGTGCTGTGCTACGAGGCACCACCGGCCACGCTCGAAGCAATACACCCTAAAGGACTTAGG
GTTTCTGTTCCTGATGAGGGCTTCTCGTTATTCGCGTTTCACGGTAAGCTCAACGAGGAAATGGAAGGCTTAGAGGCCGGCCATTGGTCCAGGGACATC

ACGAAGCCAAAAAACGGAAGATGGATATTCAGAGATCGAAATGCTGCGCTGAAAATCGGAGATAAGATTTACTTTTGGACTTTTGTCATAAAGGACGGC
TTAGGATACAGACAGGATAACGGGGAGTGGACAGTTGAAGGTTTCGTAGATGAAGCCGGTAATCCAGTAAACACAGAGGGCTCTGAAATAACACCAGGA
GTAGAATTCACCAGCACCAGTCTGAACCCAGAGTCCCCTCAGTCCATACCCAATCAGCCTCCAGACAACCTGCCCGCTAAACCACCATCTGAAGGCTAT
CCCTGTGAGCTATCGCTATCCACAGTCTCAGTACCTGGTTTTGTATGCAAAGGGCAGTTGCTTTTCGAAGATCAGTTCAATATACCAATACACAGAGGT
AAAATTTGGGTACCAGAAGTAAAGTTCCCTGGTGAACCGGACTTTCCCTTCAACGTATACCTAAGCGATAATGCAGAAGTCAACGACGGAAAGCTGATT
ATAAAACCAGCTACCTTGGAGTCAAAGTATGGCGAAGATTTTGTTCGACAATCTTTGGATCTGTCGGAAAGATGCACTGGAACCGTAGGAACAGCTCAA
TGCTTGCGAGAAGCTTCTGGTCCTCTTATTTTGCCACCCATCATAACTGCGAAAATCAGCACTCGGCATCAATTTGCCTTCAAATATGGACGTGTCGAG
ATCAGAGCTAAAATGCCAAAAGGAGATTGGCTTTATCCTGAAATATTGCTCGAACCTCGTGATAATATATATGGTGTCCGGAACTACGCTTCGGGGATA
TTAAAAATCGCTAGCGTCAAAGGAAACGCTGAATTTTCAAAGAAACTCTACGCAGGTCCTATAATGACTGGATCTGATCCTTATAGATCTTTTTATCTT
AAAGAAAACATTGGATATGAATCATGGAATAACGATTTCCACAACTACACCCTAGAGTGGAGACCAGATGGCATTACATTGTTAGTTGATGGAGAAAGT
TATGGCGAGATAAAACCAGGAGAAGGTTTTTACAACGTGGCCAACTCATATAAAGTTGAAGCAGCGCCGCAGTGGCTTAAAGGCACAATCATGGCGCCT
TTTGATGAATTGTTCTATGTGTCGATCGGTCTTAATGTGGCTGGAATCCGAGAGTTCTCCGAAGACATCTCCAATAAGCCTTGGAAAAACTCTGCCACT
AAAGCAATGCTCAAATTCTGGGACGCCCGTAGTCAGTGGTTCCCTACGTGGGATGAAGATAGCGCTCTTCAGGTGGACTATGTTAAAGTTTTTGCAATA
TGA

>XM_004922403.1 [serine--tRNA ligase, cytoplasmic-like]

ATGGTCCTCGACTTAGACTTATTTCGTGCCGATAAAGACGGTAATCCAGATAAAATTCGAGAAAATCAGAAGAAGAGATTTAAAGATGTTGCCCTTGTG
GATGCAGTCGTCGAGCAAGACACCTTGTGGAGGAAATTGCGCCACGAAGCTGACAATTTCAATAAACTGAAGAATGTATGTAGCAAAGAAATTGGTTTG
AAAATGAAGAACAAGGAACCCAGTGGCTCTGAAGACGAACCCGTCCCCTCGGAGATCGCCAATAACCTGGTAAACCTCACCGGCGATAACTTGAAGCCT
CTCACAGTAAATCAAATAAAAAAAGTAAGAGTATTAATAGATAATGCAATATCAAAGAATGATGAAGGGTTATTGGCTGCTGAGAAAGCGCGTTCCGCT
GCACTCAGGGAAGTTGGAAATCATCTGCATGAGTCTGTACCAGTTGATGATGATGAAGACCATAACTTGGTAGAAAGGACAGACGGAGACTGCGGCTTT
AGAAAAAAGTACTCTCATGTGGATCTGATCTGCATGATTGATGGAATGGATGGAGAGAGGGGATCAGCTGTAGCAGGTGGTCGTGGTTATTATCTGAAA
GGTCCTGCAGTATTCCTAGAACAAGCCCTCGTTCAACTTTCTCTAAGAATGTTACTTGAGAAAGGATATACGCCCTTGTACACACCATTCTTCATGCGA
AAAGAGGTTATGCAAGAGGTAGCACAACTAGCTCAATTTGACGAAGAACTGTACAAAGTCATAGGGAAAGGTTCTGAAAACAAGGGCGACACTGTTGTC
GAAGAGAAATACCTTATCGCTACATCAGAACAGCCCATAGCTGCTTTCCACAGGGATGAATGGCTTCCAGAATCATCTCTTCCCATCAAATATGCGGGA
CTCTCCACATGCTTCAGACAAGAGGTTGGATCTCATGGACGTGACACGCGTGGTATCTTTCGAGTACATCAATTTGAAAAGGTGGAACAATTTGTTCTA
ACATCTCCTCATGATAACGCATCTTGGCAAATGATGGACGAAATGATAAACAATGCTGAAGAGTTCTGCAAAGCACTAGGACTACCGTACCGTGTCGTG
AACATCGTGTCTGGAGCTCTAAACCATGCAGCTTCCAAGAAGCTAGACTTGGAGGCCTGGTTCCCAGGATCCGGTGCGTTCAGGGAACTTGTCTCTTGT
AGTAACTGTCTGGAGTACCAAGCCAGAAGATTGCTTGTAAGGTACGGTCAGACGAAAAAGATGAACGCTGCCACTGAATACGTGCACATGCTGAACGCG
ACCATGTGCGCCACCACCAGGGTCATTTGCGCCATACTCGAGGTACACCAGACAGAGGACGGCATCAAGGTACCGGAAATCCTCAAACCTTGGCTTCCC
AAGCAGTATCAAGAATTAATACCGTTCGTGAAACCCGCACCCATTGACGTGGAGGCGGCCGCTGCAGCCAAAAAAGGAAAGAAACAAGAAAAAAAATGA

>XM_004931655.1 [voltage-dependent anion-selective channel-like isoform X4]

ATGGCTCCCCCATATTATGCTGACCTTGGAAAGAAGGCCAATGATGTCTTCAGCAAGGGCTATCACTTTGGTGTTTTCAAACTCGACCTGAAGACCAAG
AGCGAGTCTGGTGTTGAATTCACCAGCGGAATCACCTCCAACCAGGAAAGCGGAAAGGTTTTTGGCAGCCTTTCCTCCAAATTTGCAGTGAAAGACTAT
GGCTTGACTTTCACAGAGAAGTGGAACACAGACAATACATTAGCTACTGACATCACAATCCAGGACAAGATTGCTGCTGGCCTTAAAGTCACCCTTGAA
GGCACTTTTGCCCCACAGACTGGAACTAAAACTGGAAAATTGAAGACCTCATTCACCAATGACACAGTAGCAGTGAACACTAACTTGGATCTGGACTTG
GCCGGTCCAGTTGTAGACGTTGCAGCAGTACTAAACTACCAGGGTTGGCTGGCTGGTGTACACACCCAGTTTGATACACAAAAAGCAAAGTTCTCCAAG
AACAACTTTGCTCTAGGTTACCAATCTGGTGACTTTGCTCTCCACACAAACGTAGACAATGGTAAAGATTTCGGCGGTTCAATCTACCAGAAGGTATCT
GACAAGCTGGACTGCGGCGTCAGCATGAAGTGGACGGCGGGTTCGGCCGACACATTATTCGGAGTTGGAGCGAAGTACGCGCTGGACCAAGACGCGTCT
CTGCACGCCAAGATCAACAACAAGTCCCTCATCGGTCTTGGTTACCAACAGAAACTACGCCCAGGCGTAACCCTTACATTGTCTGCTGCCATCGATGGA
CAGAACTTCAATGCAGGTGGCCACAAGGTTGGCGTTGCCCTCGAACTCGAGCCCTAG

>NM_001099622.1 [proteasome beta subunit] ID:NP_001093092.1]

ATGTTGAGCGTTAACGAGAACTTTCCTGAATATGCTGTGCCTGGTGCGAAACAGCATCGTTTCGAGCCCTACGCGGACAACGGAGGTAGTATTGTAGCC
ATCGCCGGCGATGATTATGCCGTTATCGGAGCAGACACTCGTCTCAGCACTGGATTTTCCATATATACCAGAGACCAAAAAAAGCTGTTCAAATTATCT
GAGAGTACAGTATTGGGAGCGACTGGCTGCTGGTGTGACACATTAACCTTGACGCGTCTTCTACAAGCGAGGATGCAGATGTACGAACACGAGCATAAC
AAATCTATGACCACTCCGGCAGTCGCACAGATGCTCTCAACTATGTTGTATTACAAACGATTTTTCCCATACTACGTATCCAACGTGTTGGCGGGTTTG
GACAGCGACGGTAAGGGATGTGTATATAGTTACGACCCTATCGGACACTGTGCGCGCCATAACTTCCGCGCTGGTGGTTCCGCTGCTGCACAGCTGCAA
CCGCTCCTCGACAATCAAATTGGCTTAAAGAATATGCAGAATGTAACAGAAGCTCCTTTGCCTAGGGAAAAGGCCCTTGCTCTTCTGAAGGATGTGTTC
ATCAGTGCTGCAGAACGTGACATTTACACTGGAGACTCCATCTACATTCTCATCATCACTGCAAGTGGTATTCAAGAAGAGAAATTTGAACTTCGCAAA
GATTAA

>NM_001048238.1 [receptor for activated protein kinase C RACK 1 isoform 1]

ATGTCTGAAACATTAAAACTCCGAGGAACTCTTAGAGGCCACAATGGCTGGGTTACTCAAATTGCAACTAATCCGAAATACCCGGACATGATCTTATCT
TCCTCTCGAGACAAAACTCTCATCGTGTGGAAGCTGACCAGAGACGAAAACAACTACGGTATACCTCAAAAGCGTTTATACGGTCATTCGCACTTCATT
TCTGATGTTGTGCTGTCTAGTGACGGTAATTACGCCCTTTCCGGTTCTTGGGACAAGACTTTGCGTTTGTGGGATCTCGCTGCAGGCAAGACTACCAGA
CGATTCGAGGACCATACTAAGGATGTACTCTCGGTAGCCTTCTCAGTTGACAACCGTCAGATAGTGTCTGGTTCTCGCGACAAGACTATCAAACTCTGG
AACACCCTTGCGGAGTGCAAGTATACCATCCAAGATGATGGACACAGCGATTGGGTGTCATGTGTCAGATTCTCACCCAATCATGCCAACCCCATTATT
GTATCCTGTGGTTGGGACAGAACTGTCAAGGTCTGGCATCTCACTAACTGTAAGCTCAAGATTAACCATTTGGGTCACTCTGGCTATCTGAACACTGTT
ACTGTATCACCTGATGGCTCTTTGTGTGCATCTGGTGGTAAGGATATGAAGGCGATGTTGTGGGATCTGAACGATGGCAAACACCTCCACACCTTAGAC
CACAATGATATCATCACGGCCTTGTGCTTCTCACCCAACAGATACTGGCTATGTGCTGCTTTCGGACCTTCCATCAAGATCTGGGATCTGGAAAGCAAG

GAGATGGTTGAAGAGCTCAGGCCTGAAATCATTAACCAAACGCAAACTTCCAAGACAGACCCTCCTCAGTGTCTCTCCCTAGCATGGTCTACTGATGGA
CAAACCTTATTTGCTGGTTACTCAGATAATACTATAAGAGTGTGGCAGGTTTCCATCTCAGCTCGATAA

>AB362775.1 [juvenile hormone epoxide hydrolase] ID:BAF81491.1]

ATGTCACGTCTTCTGCTTATCGTCTTGCCGCTGCTAGTGCTGGCGTCCATACCATTGTACCTCCTCGTTCTGAAGAGTCCACCGCCGATGCCTAAGCTA
GACCTGGAGGAGTGGTGGGGACCGCCCGAACTTAAACAGAAACAGGATACCAGCATCAAACCCTTCGAGATCACTTTCAGTGAGACGATGGTAAAAGAA
CTAAAAGAACGAATCAAGAAACGAAGACCTTTCGCCCCTCCATTGGAGGGTGTTGGCTTCAAATACGGGTTCAATTCGAAACAATTGGACAGTTGGCTC
AAATATTGGGCAGAAGAATACCCATTCGCTGAAAGGCAGAAGTTCCTTAACCAATATCCTCATTTCAAGACTAATATCCAGGGTTTGAATATTCACTTC
ATGAGGATTACTCCAAAGGTCCCTAAAGACGTTGAAATAGTACCACTTCTGTTACTCCACGGATGGCCGGGCTCCGTCAGGGAGTTCTACGAAGCCATT
CCTCATCTCACAGCTGTCAGCAAAGACAGGAACTTCGCTCTGGAAATTATCGCTCCAAGTTTACCTGGTTATGGCTTTTCCGACGCGGCGGTTCGTCCC
GGCCTCGCTGCTGCTGAAGTTGCAGTCATTTTCAAAAACCTGATGGCCAGACTTGGTTACAAGCAGTACTACGTCCAAGGAGGTGATTGGGGAGCTCTC
ATCGGTAGCGCTATGGCTACTTTCTTCCCTAAGGAAATAATCGGCTTCCACTCCAACATGGCGCTCACTCTTAGTCCAGCGGCGACGTTCCTTGAGTTT
GTCGGTGCCTTATTCCCATCTTTGATAGTCGAACCAGAACTAGCTAACAGACTTTATCCATTATCGGAGAAATACTCAACCCTTCTCGAGGAATTGGGC
TACATGCATATTCAAGCCACGAAGCCTGATACTGTTGGAATTGGGTTAACAGACTCCCCGGCCGGTCTCCTCGCCTATATCCTGGAGAAGTTTTCCACT
TGGACCAACCCCGACTTACGCAGTAAAGAAGACGGAGGTCTCTCCTACCGGTGGACAAAAGACCAGCTGATCGATAATCTCATGCTGTACTGGAGCACG
AAGTCAATAGTCACTTCAATGAGACTTTATGCTGAGTCTTTCAGTTCTAGGCACTTCGATTTAAAACTAGATGAGATTCAAGTACAAGTCCCAACATGG
GTGCTCCAGGCGAAACACGAGTTAGCCTACCAGCCGCCCTGCATCCTCAAACTGAAGTACCCGAAACTGGTCAATGCCAGTGTCATTGAAGACGGAGGC
CACTTCCTGGCGTTCGAGCTACCGGAGATCTTCGCCAAGGACGTCCTGAAGGCAATCGGCGAGTTCAGAAAACTCAAGAACGTGAAGACTGAGTTGTGA

>NM_001126249.1 [beta-fructofuranosidase precursor] ID:NP_001119721.1

ATGTTCGCCTGGAGCACACCGGTGGCGCTGGCCGCCGTGCTCGCCGTGACGCTGGGACAAGCTCTCCGCCAGCAAAATGAGACCACTAAGCGAGAACTT
GAAGAATACATCGCGGATAAAAAGGCAGAAATCAATCCACGGTACCGCCCGCACTATCACATCTCTCCGCCCGTCGGCTGGATGAATGACCCTAACGGC
TTTTCATACTACAAGGAAAAATTCCACCTCTTCTATCAGTTTTACCCTTACGACAGCGTTTGGGGTCCGATGCACTGGGGTCACGTTTCCAGTAGCAAC
CTGATCGACTGGGAACACCTCCCTACGGCTCTCATCCCTGAAACCGAAATGTGCTTCTCGGGCGGCGCCGTCGTCCACGGAGATGACCTGGTACTGTTA
TACACGGGCCGCGTCACTACTGACACGGACCCGTTTTACAACGAAACTCAATATCTAGCCTTCAGTAACGACGGAGTTAACTTCCGCAAGTATGAAGGA
AATCCAGTCCTCTCCTACGTGCCCGACAATTCAGCTGATTTCAGAGACCCCAAGATTTGGAAATTCAAAGATCATTGGTATGTTGTCATTGGCAGCTCA
AGCAATAAACGGGGAAGAGTGCTCCTTTACAGATCAGGGGATCTGTTTAATTGGGAGTTTTTGTCGGTCTTGGGAGAATCAGACGGCGACATGGGCTAC
ATGTGGGAATGTCCCGATCTGTTTGAACTCGGCGGAAAAACCATTTTCCTCTGGTCCCCTCAAGGATTGGAACCGAAAGGCGACAGATACAAGAACACG
TATCAGACCGGATACTATATTGGGGAATTAGACTATGAAACTTTTGAATTTAAAACTGATAAGTATTTCCAAGAATTAGATTACGGTCACGATTTTTAT
GCGACTCAAACCATACAGGGCGACGGCAAGACTTACTTGATTGGCTGGTTTAATATGTGGGAAGTGCCGCACCTTGAGAAAGAGGACGGCTGGGCGGGG
ACGACGACGCTCGTTAGAGAGCTGCAGCTGATCGGCACGCGGATCACCATGAACCCACTAGAAGGAATCCAAGATCTGAGAACCGATAGCGTCCATAAC
GGAGATCTAGAGCCCCAGCAAGCGATAGAATTCGGCCCAACTGCTGAAATTATTCTACAAGGATGTCTCGACCAGAAGATTGAGTTGCTGATTCAAGGT
AAAGAAGGAGGTCTTGTCACAACAGTGACGTGGGATCCTGAAGTCGGTAAAGTGATTGTTAACAGGAGCGGAGAAGTAAGGCAAGTGGAATGGGTTCCA

ATCGGAAAGACCTCATGGAGACTATTCTTGGATGCAAGTTCTTTGGAGTTGTTCTGTGGAGAAGGTGAGGTAGTATTCAGTTCGAGGATCTTCTCTGAT
GGAGATTGGGTCGTAAAGAATTCCAGCCCGCAAACCTTGTCCGTAGAAGCGTATCGATTGAGAAGAAGTGTACCCGCTTAA

>AB620073.1 [glycosyltransferase] ID:BAK82125.1

ATGTTAGGGAAAGATCCCAAAAAGCAGATTGTGAAATATCGCGAATCTCAGTGTACGTTAAAAAGACTGATAATGAGGAGAAACAGATATAAATTGATT
GTATTCGCTTTGGTACTTGTATATGTTTACCATTTTTTCGGTGTCGGCGATTACGTGCAGTCCAAGAACTTCGATAGCGATTTCAATTATCCTCTCAAC
GTAGACATTCGACCGATAGTCCAAGCAATACTAGACGGGCAAAAGCCGAATGTTAAACCTATAAATTACTACCCGTACAAGTTCTTGAGCAACTATCGT
CAATGCAGTGTCGTCAACAAACCGGATTTAGTCATAATAGTCAAGTCGGCTATAGACCATTTCGGACACAGAGATGCAATTAGAAAGACTTATGGAAAA
CCACATGTCCAGGGCTACAACGTGAAGACATTCTTCTTTTTGGGCGTGGACAATGCCAGTTCAGATGTACAGAAGAATATTACGAAGGAAATGACAGAA
TTCAAAGATATCATACAGATGTCTTTCCGTGACAGTTATTTCAACAACACCATTAAAACGGTCATGTCCTTTCGCTGGATCTTTCAGCACTGCGCTGAA
GCTCAACACTACTTATTCACCGACGACGACATGTACATATCGGTACAGAATTTACTAAAATACGTTAGCGACGTGACGACCGCGAGCGAAAGAGACGGA
ATACTGTTCGCCGGTTACGTCTTCAAGTCGGCGCCGCAACGGTTCCGGTCGAGCAAGTGGCGGGTGTCCTTGGAGGAGTATCCCTGGGACAAGTGGCCG
CCTTACGTGACGGCCGGGGCGTACGTGGTGTCAAACAAAGCGATGAAGATGCTCTACGTCGGAAGCTTGTTCGTGAAGCATTTCAGATTCGATGACATT
TACCTCGGCATAGTCGCGAAGAAGATGGGCATAGTGCCGACTCATTGTCCGCATTTCCACTTCTACAAGAAGCCGTACGAGCGAGAGGTTTATAGCGAT
GTGATCGCCTCGCACGGATATTCTAATCACGACGAATTGATTAGAGTGTGGAATGAACAAAACGCGTTGTGA

Helicoverpa armigera sequences matching 50 open reading frames selected from _Heliothis virescens_ (Back to top)

>JF417990.1[annexinIX]ID:AEB26321.1
ATGAGTGGACAACAGTACTACCCTTACAAGTGCACCCCCACGGTGTACCCAGCGGAGCCCTTCGAGCCTGCTGCGGACGCCGAGACTCTTCGCAAGGCG
ATGAAAGGAATCGGCACCGATGAGAAAGCCATCATCGATGTCCTTTGCAGACGTGGTATCGTCCAGCGTTTGGAAATCGCTGAGACTTTCAAGACCAAC
TACGGCAAGGACTTAATCTCTGAGCTGAAGAGCGAGCTGAGCGGAAACTTGGAGAACGTGATCGTGGCGCTCATGACTCCCCTCCCCCACTTCTACGCT
AAGGAACTGCATGACGCCGTCGCGGGGCTTGGTACTGATGAGGAAGCTATCATTGAAATCCTCTGCACACTCTCCAACTATGGCATCCGTACCATCTCT
GCTTTCTACGAGCAATTGTACAACAAGAGCTTGGAGTCTGACCTGAAGGGCGACACATCTGGCCACTTCAAGAGGCTCTGTGTGTCTCTCTGCATGGCC
AACCGTGATGAGAACCAGGGCATCGATGAGGGAGCGGCGAAGGCTGATGCTGAGGCTCTCGCGTCTGCCGGTGAGGGACAGTGGGGCACTGACGAGTCT
GTCTTCAACTCCATCCTTATCACACGCTCTTACCAGCAGCTTAGACAGATCTTCGCTGAATACGAGCAGATGACCGGCAAGGACATCGAGGAGACCATC
AAGAAAGAATTCTCTGGAAGCATCGAGAAAGGCATGCTTGCTATCGTCAAGTGCGTTAAGAGCAAGGTAGGCTTCTTTGCTGAGCGCCTTTACTACTCC
ATGAAGGGACTGGGAACTAACGACAAGACCCTCATCAGAATCATCGTCAGCCGTTCAGAAATCGACCTCGGTGACATCAAGCAAGCTTTCCTTGAAAAA
TACGGAAAGTCCTTGGAGAGCTGGATTGCCGACGATACGAAAGGCGACTACAAACGCGTGCTACTCACTCTAGTGTCGTAA-

>HM347785.1[NADPHcytochromeP450reductase]ID:ADK25060.1
ATGTCAGACAGCGCACAGGACGTTCTAAAGGATGCGGCGACCGGCGCTGCAGCTGCTGCTGCCGCTGGGGGATCGCTTTTCAGCACGTTTGATATCATT
GTGCTGGTCATCTTATTGGGCGGCACCATTTGGTGGCTGTATAACTCTAAAAAGGAAAATAAAAAAGATGAAATCCTTCTTAGTAAATATTCAATTCAG
GCTGCGGGATCCATCCAAGTTACAGAAAATTCTTTCATAAACAAATTGAAGTCGTCCGGAAGAAGTTTAGTTGTATTCTATGGATCCCAAACTGGTACT
GGTGAGGAGTTTGCCGGTCGTCTTGCTAAAGAAGGCATACGATACAAGATGAAAGGCATGGTTGCTGATCCTGAAGAATGTGATATGGAAGAACTCATG
AAACTCCAAGACATTCCGAATTCTTTAGCTGTGTTCTGTATGGCAACATATGGCGAGGGAGATCCCACAGATAACTCCATGGAGTTTTATGAATGGTTG
AAGAACGGAGAACCCGATTTGACTGGTTTGAATTATGCTGTGTTTGGCCTTGGCAACAAAACATATGAACATTACAATGCTGTTGCTATTTATCTAGAC
AAGCGTCTTGAAGAACTTGGCGCTACTCGAGTGTATGAACTTGGTCTTGGGGATGATGATGCAAACATTGAAGATGACTTCATTACATGGAAAGACAAG
TTTTGGCCAGCAGTTTGTGAGAAATTCAATATTGAGAGCACTGGAGAAGAAGAATTGATTCGTCAATTCAGACTTGTGACACATGCCCCAGGGGATATC
CAACCAAACAATGTGTTCACTGGTGAAATTGCCAGGCTGCACTCTCTACAAGTTCAAAGACCGCCCTATGATGCTAAGAATCCTTTCTTAGCCCAAATT
ACAGTAAACAGAGAGCTACACAAAGGCGGCGACAGGTCCTGCATTCATGTGGAACTTGATATTTCAGACTCAAAGATGAGATACGAAGCAGGTGACCAT
GTGGCTGTATACCCAATAAATGATACCAATCTTGTTGAACGACTGGGTCAGTTAACAGGAGCAAATCTTGACGAAATATTCTCGCTCATCAATACTGAT
CAAGAAAGCAGCAAGAAACATCCATTCCCTTGCCCCACATCCTATCGCACTGCTCTATCCCACTATGTGGAAATCACTGCATTACCCCGTACTCACATT
CTACGTGAGCTTGTTGAATATTGCTCTGATGAGGAGGATAAAAAGAAGTTGATGTTGATGGCAACTAACTCTCAAGAGGGCAAGGCTCTGTATCAGTCA
TTCATAGTGGATGCTTGTAGAAATATTGTACACATTTTAGAAGACATACCATCATGTAAGCCTCCCCTGGATCATCTTTGTGAACTGCTGCCGCGTCTG
CAACCAAGATATTATTCAATTTCATCCAGTCCTAAGATGTATCCAGAGACTGTGCATATTACTGCTGTGATTGTTAAGTATAAGACACCAACTGGTCGC
GTTAACAAGGGCGTAACTACCACATGGCTGGCCGAAAACAAACCAGAACCTGGAAAACCACTACCTCGTGTACCCGTGTACATCAGGAAATCGCAGTTC
AGGTTACCATTGCAGACCCAAACTCCGATAATAATGGTCGGCCCAGGTACTGGTTTGGCACCTTTCCGTGGTTTCTTACAAGAACGTGCATTCGCTCGT
GCTAATGGGAAGGAAGTTGGAGAAAGTGTTCTTTACTTTGGATGCAGGCATCGTGATCAGGACTACATTTACCAAGATGAACTTGAAAAGTATGAGCAA
AATGGTGATGTTAAATTAAACCTTGCATTCTCTCGTGACCAAAAAGAGAAAGTTTACGTGACACATTTGTTGGAAAAGGATATGGACCTTTTATGGGAC

GTCATTGGTAATCGCAACGGTCACTTCTACATCTGCGGTGATGCGAAGAATATGGCTGTTGACGTAAGGAACATAGTTCTAAAAGCTATCCAAGAAAAG
GGCGGCCGCACAGAGTCTGAGGCAGTGCAATTTATTAAGAAGCTGGAGTCTATGAAGAAATATTCAGCAGATGTATGGAGTTAA

>GU584103.1[3-hydroxy-3-methylglutarylcoenzymeAreductase]ID:ADM13643.1
ATGAAAGTCTGGGGAGCTCACGGGGAGTTCTGCGCTCGCCACCAATGGGAGGTCATCGTGGCCACATTAGCTCTGCTGGCTTGCGCAGCCAGCGTCGAG
AGGAATGGCCCTGGAAACAGGTCGGAACACTGCGCCGGCTGGGCCAGGGCCTGTCCCGGGCTAGAGGCAGAGTACCAAGCCGCTGATGCCGTAATAATG
ACCTTCGTACGCTGCGCAGCCCTCCTATACGCCTACTACCAAATCTCAAATCTCCATAAAATAGCCTCAAAGTATCTTCTCATCATTGCCGGGGTGTTT
TCCACATTTGCAAGCTTTATTTTCACTTCAGCTGTCGCCAGTTTGTTTTGGAGTGAACTGGCGAGTATTAAGGATGCCCCCTTCCTGTTTTTATTAGTC
GCTGATGTGGCTAGGGGGGCGAGAATGGCGAAGGCAGGATGGTCGGCTGGGGAAGACCAGGGTAAGAGGGTTGGAAGAGCACTGGCGCTGCTTGGACCA
ACAGCGACCCTCGATACTCTGCTAGCAGTTTTATTAGTGGGAGTTGGAACTTTATCTGGTGTTCCCAGGCTAGAACACATGTGTACCTTCGCCTGTCTA
GCTTTATTAGTAGACTATCTGGTCTTCGTAACGTTCTACCCAGCGTGCCTCTCCTTGGTCGCGGATTTCGCTCCAGGACGCAAAGAAATGTCCCCAGAC
AGTCCGTTTTCTGAAGCCGATCTTAAACCGAACCCAGTGGTTCAGAGAGTGAAGATGATAATGGCGGCAGGGCTGCTGTGTGTTCATCTCACCAGCAGA
TGGCCCTGGAGCAACGACAACGGGATCATCGAAGGTCCCATAGACACATTAACTCCTTCCACAAAAGATAATATTTTGTTACATTCGTATATCAAATGG
TTTTCCGTTTCTGCGGATTACATAGTCATAGCGACACTGCTCTGTGCCTTGATTATAAAGTTTGTATTCTTTGAGGAGCAACGAAACTGGGTTATTGAC
ATGAACGATATGACGGTGAAAGAAGTAGTACAAGAGCCAGCTCGGAATAAGCCTAAATTCTCAGTAGGAGATGACTCGAATTCTGAGGTTTCCACGCAA
ACGGAGGGAGTATTGGAAGATGAATGGCCTACGCTTTCACCGAGCTCGTCCGCTGCTAAATTAAACTCCAAAAAGCGTCCAATGGCGGAGTGCCTCGAA
ATATACAGATCTGAGGGAGCCTGCAACTCTTTGAGTGATGAAGAAGTCGTGATGTTGGTTGAACAATCCCATATTCCTTTACACAGACTCGAAGCGGTC
TTAGGAGATCCTTTACGCGGCGTAAGACTGCGTAGAAAAGTAGTCGGGTCTAGGTTCCAGACTGAATTAGCTATAAAACAACTGCCTTACTTAAATTAT
GATTATTCTAAAGTTTTGAACGCCTGCTGCGAAAATGTGATCGGTTATGTTGGTGTGCCAGTCGGGTATGCTGGGCCTCTGGTTGTTGATGGTAAGCCG
TATATGATCCCTATGGCTACAACTGAAGGGGCTCTAGTGGCTTCGACAAACAGAGGTGCCAAAGCCATCGGTTCCCGAGGAGTCACAAGCGTGGTTGAA
GATGTGGGTATGACAAGAGCACCGGCTATTAAGCTGCCTAACGTTGTGAGAGCTCACGAATGCAGACAATGGATAGACAATAAAGATAACTACGCACTC
ATCAAAGAAGCTTTCGACTCCACATCGCGTTTCGCTCGACTCCAAGAAATTCACATAGGAGTTGACGGCGCAACTCTATACTTAAGATTCAGGGCTACC
ACAGGCGATGCTATGGGTATGAACATGGTCTCAAAAGGCGCTGAAAATGCCCTCAAACTCCTCAAAAACTACTTCCCTGATATGGAAGTTATAAGCTTG
TCTGGTAACTACTGCTCGGACAAAAAAGCCGCAGCTATTAACTGGGTTAAGGGAAGAGGGAAACGAGTGGTTTGTGAAACGACGATATCCCACGATAGT
TTAAGAACGATCTTCAAAACCGATGCCAAAACTCTGGCTCGGTGCAACAAGATAAAGAATCTCTCTGGATCTGCGTTAGCTGGTTCCATTGGCGGGAAT
AACGCTCACGCAGCAAATATGGTGACAGCTATTTATATAGCTACAGGCCAGGACCCGGCTCAGAACGTTACCAGCTCGAATTGTTCGACAAACATGGAG
GTTTGTGGAGAAAATGGAGAAGATCTGTATGTGACCTGTACTATGCCTTCTTTGGAAGTTGGTACAGTAGGTGGAGGGACTATCCTAACAGGCCAGGGT
GCTTGTTTGGACATTCTAGGGGTCAAAGGCGCGGGGGCACGACCAGCAGAAAATTCTGCCCGATTAGCCTCCTTGATCTGCGCCACAGTTTTAGCGGGA
GAGCTCAGTCTAATGGCTGCCCTTGTGAATCCAGATCTTGTAAAATCACATATGAGGCATAACAGATCGACGGTGAACGTGCAAGCGAGTTGCAACTTA
CAAACTGACGGTATCACCTTGAAAGTGCCGACCTTATAA

>AY279535.2[midgutaminopeptidaseN2]ID:AAP37951.1
ATGCAATTCATCACCATCATACTCCTGGCGTCGGCCGCCATCATCTCGGCCGACTTCCCTCTTCCACCGGAATTTGATGAACCGGAGTTCTTCAGTACT
TCTGACCCAGATTCCTACCGGCTCCCCGAAGATCTGGACCCCATAAATTATGTTGTGGAGGTTACCCCTTACTTCACTGCTACAGATACCAAGGAGGCG
TTCACCTTTGATGGTCTTGTTACCATCACTCTTAGGACACTGAAAGCTGACCTGAACGCGCTCATCATTCAAGAGAACGTACGCACAATAAATTCCGTA
GCTCTAACTACCGAAGCCGGTACCTCTGTTCCTCTTCATGCCACTACTCCATTCGAACGCATAACTGCCTACCATTTCTTGAAAGTCAACCTACCTGCT

GGGGCTACTTTAGAAAATGGAGCAGTTTACAAGCTGACTGTCGATTATGTCGGTAACATTAATGAGACACCATTATCCCGAGGTGTATTTAGAGGCAGC
CATAAGGACGCAAACGGTAATACACGCTGGTACGCAGCTACTCATTTGCAACCAACAAACTCAAGACAAGCATTTCCCAGCTTCGATGAGCCTGGTTTC
AAGTCTACCTTCGACATCATCATCAACAGACCAGTAACCTTTGCTCCGTCCTTCTCCAATATGGAATCAAATCAAGCGATTTGGTCAATAACCGCATT
AGGGAGGTGTTCTATACTACCCCGCGGATGTCAGCCTACTTGGTAACCTTCCACATCAGTGAAGACTTCACCGTCATCGCCAACAACAATAACGACGCG
AGATCTTACAGAATCTTAGCCCGACCCACCGCTGCTGGCCAAGGACAATACGCTCTAGAAGTCGGACCTCCAGTGACTAATTGGCTCGGCGAGTATTTA
GGTATTGACTACTACAGCATGGATGAGAACACTAATATGAAGAACGATCAAATAGCTTCTCCTTATTGGGCTTCAGGAGCCACTGAAAACTGGGGATTG
GTAACATACAGGGAACTGCGTCTGTTATACCAAGAAGGTGAGACCAATGCTTTGGACAAGATGTACATCGGTACTATTACTGCTCACGAGCTCGCTCAC
AAGTGGTTCGGTAACCTCATCACCTGCAGATGGTGGGATAACGTCTGGATCAATGAAGGCTTCGCTAGCTACTTCGAATATTTCGCCATGGATGGAGTA
GACAAAACAATGGAATTAGAAGATCAGTTCAACATTATGTACGTTCAAAGCGCTCTCTCTGCTGATGCCACGCTTTCGACAAGGGCCCTTCAACACACA
GTGAACAGCCCAACCGAAGTCACAGGACACTTCAGTGGTATCAGCTACTCTAAGGGAGCTTCTTTGCTCCTCATGTTGAAGCATTTCCTTACGGAAAAT
ACTTTCAAAAAGGCTTTGAACATATTCTTGGAAGCCAGGAAATTCGAACACGCATTCCCAGCAGACCTCTTCAGTGCTTTTGCTACAGCAGTTCAGCAA
GATGGTGTACCATCAAACACCTTCGATATTGCATCATTCATGAAATACTGGGTAGAGGAACCAGGATATCCAGTCCTTGAAGTTTCTGTCAATTCAGCA
GCTGGTCGCATTGAGCTATCTCAGAAACGTTTCTTAGTCAGCGCCACTGCCACGCCCACCGACCAAGTTTGGCCGCTGCCCCTTACTTACACAACTGAG
AGCAACCCTGACTGGCAGAACTTGTTGCCTAGTAAAGTGATGACTGCAAAGACTGACTTCATTGAAAGGAATGTAGGAACAAATGAATGGGTTATCTTC
AACGTTCAACAGAAAGGTATCTACCGTGTGAACTATGACACTCGCAACTGGGAGTTGCTTGCAGCCGCCCTGAGCAGAGACCACACGGCTATCCATCAT
TTGAACAGAGCTCAGATCGTTGACGACGTTTTCGCTCTGATGAGATCTGGACAAATAACGTATCGTCTCGGATTCAAAGTTCTGGACTTCTTGAAGAAG
GACACCAGCTACTACTCGTGGTATCCGGCTATTACTGGTTTCAACTGGCTGAGGAACCGCTTCCTCCATCTCCCTACGACCTTGGCTGCTTTTGACGAA
ATTCTTTACGGCTTCTTAGATGCTGTGATAACTGATCTTGGATATGATGTGGTTGCAAATGAACCTCTTACGAGAACACTGAATAGATTCTTCACACTG
TCATTCGCTTGCAACATTGGACACAAAGGTTGTGTCGACAATGCTGTACAAAAGTTCGTGGCTTTGAAGGATAACAGTGTCGCTGTCAACCCTAACCTG
AGGCGCCACGTGTTCTGCGAAGGTCTCCGTGCCGGCGGTCTCGATGAGTGGCAGTACCTCTACAACCGTCGCCAAGCCTCCAACAACCAGGGAGACGAA
GTCGCTATGCTCAGATCTTTAGGATGCACCAGCAACACTGCGGCTGGACAGGCGTACTTGAAAATGATTTTGGATGACGATGTCGTCAAAGCCCAGGAC
CGAGTGAACGCATTCTCGTTCTTCTACATGGGACATCGTGACAACGCCAAGGCAGGTCTGCAATTCCTCAAGGACAACGTTGATGCTATCAGAAAAGCT
GTCGTCCTTCCAGCGTGGTTCAACAACGTTCTCACAACCACCGCCGGCTATTTGGACGAAGCTGGTCTGAGGGATATGGAAGAATGGCTGTTGGCTAAC
CAAAACGCTGTCCCCGAATTCGCTGTGGGCATCAGCGCCATAACCTCAGCTAGAAATAACATGCAGTGGGGTTCAGACAATGCTGCTACCATTATAGCT
GCAGCCAATGATGAAGATCCCCCAGAAGATGGTGGTTCAGGAGAAGAAGTAGACCCGACACCTGCGCCGACTACAACTACAACACCTGCGCCGACTACA
ACTACAACAGAAGCGCCAACTACGACTACAACAGAAGCGCCAACTACAACTACAACAGCTGAACCAACAACAGAAGGGTCTGGCGAAGAATCAACAACA
CCTGATGGATCATCAACTACAGCAGCGCCGGAGTCAGGCGAAAGTGAACAAGATGAGTCAAGTTCAGCAACAATCTTCCTACCAACCACCGTACTACTG
TCGTGGACAGTACTCGCAATGTTGTTATAA

>JN315690.1 [relish]ID:AEO51739.1
ATGTCTACAAGCAGTGACCACGATATGAGCGATGCCTTCAGCTCTCCCATGTCGTTTAACATATACTCTCCTCCTCATAGTTCTCCGTCGCAGCAGGTT
CCTCAGCTCGCGACTGACCTCACTGAGCTGACTTGCGCTGACAATAAGCGACAATTTCTTAGACACGCCATGAAACCATTTCTGCGTATCACGGAACAG
CCTCAGAATCACTTCAGATTTCGGTATGTCAGCGAGATGGTTGGCACACATGGATGTCTTCTGGGAAAATCTTATGGCTCCAACAAAACAAAGACTCAT
CCTACTGTTGAGCTACTTAATTACAGAGGTAAGGCTCTGATAAGGTGTCGTTTGGCTCAACACAACAACCCTGATGAGCACCCACACAAGTTGCTTGAA
GATGAACAGGATCGAGACATGAGTTACCTTGTTCCTGATCAGGGAAGCTATAAAGTCGGGTTCGGCGGGATCGGCATTATACACACTGCGAAAAAAGAT
GTGGCTGCGCTACTATTCAAGAAGTTTTCTGAACAATGCAAGAATACTAACGTGAACTTGAAAGACCTTCAAGTACAATGCGAGAATATGGCCAAAACT
ATAAATTTGAATATCGTTCGCTTGAAATTCAGTGCACATGATACTGTTACTGGTCAAGAGATATGTGAACCTGTGTTCTCAGAACCTATTCATAATATG

AAGAGTGCTGCTACTAACGATTTGAAGATATGCCGCATTAGTCGGTCCTCCGGCAGTGCATCTGGGGGAGAAGATGTCTTCATACTTGTCGAGAAAGTT
AACAAAAAGAACATAATGATTCGCTTCTTCGAGATGGACGAGAACGGTGAAAGAGGTTGGTCTAGCTACGGCCAGTTTATGCAGAGCGATGTTCATCAC
CAATACGCCATTGTTTTTAGGACTCCGCCATACAAAAATCCCGAGACTTCAGTCGATGTGCACGTGTTCATAGAACTAGTGCGTCCCACTGACGGCCGT
ACCAGCGAACCTAAAGAGTTCAGGTACAAAGCGAACCAAGCTTACAAACAGATCAAGAAAAGGAAAACCGGATCGTCTTACTGCTCTATCGGCAGTTCT
TCAAGTGGATCTCTGAAAAGTGGCTGCGACATTCCCATATCTGTTGTCAATCATCAAGCTGAAGAAATTATGGAGCGCGATCCCGATCCGACTTCGATG
TATATCTTGCCTCAGGTTCACAACCCGACTTCGCCGAACCAATGCGATCTTGGCAGCGCTCTATACTCAGCCCCCGGGTCTGAGTCCAGCATGTCTCCG
ATGTCCAGCCCCATGTGGAGCGAGCCCCACAGCGTCATGCTCCCGGAGCCACCCATCGCAGACTTGAAGCTTAACTCGGCAGACTTCGAACAGATCACA
GCAACAAACAACGATCCCGCCAGCTATCAGATACCAGAGGAAGTTAAAAGCTTTTTGAATGAATACATGAAAAGTTACGGAGAAACTGTTCCCGAAGAA
AAAAACAGTATGGAGTTTATTCGCTCATTGCTAGTCGCTGACTCGGGACGGCCAAAGTTACAGCAAGACGCGAAGGCACATTTCCTGACACCACGAGCG
CGTGAAGGCTTTCCCAAAGATGAGGGCGAACTCAAAAAGTCGAAGACGGAGTACCCGGCTTTCTACAAAACGGAGGATGGCATCGAAGTTAAGAAGATT
GTAAAAGACTTGTGCGAAATGATCAGAAATAAAAAGGGATTCAAAAAGGCTGAAGTTAGAAGCCGGTTGGAGCGGTTATTTGAAATAAGACTGTCGAAT
GGAGATACATTCCTCCACATGGCATTGTGCAGTAACCAGCCAAGTCTTGAGTACATCGTGAAAATAATCCATAGTGTTAAGGCTACGCACCTCCTTGAT
TGCGCCAATGATAGACAACAGACCACACTACACATCGCCGTTGTCAATGATATGCCGAAAATGGTGTCTTTGTTTGTTGCGAAAGGATCGAACCCAATG
TTAAAGGATGACGAGGACCTAAATGTGATCCACTACGCCGTGAAGTACAAGTCGTGTTTAGAAGTCCTTCTGGACTCCATCAAGAAGAACGATGTGCCC
TGTGATTTGAACGATTACAATGGTGAGAAGCAGTCAGCCCTACACATGGCGGTGGTGTCGGGCTGGGCGGACGGGGCTCGGCTGCTGCTCTCGCACGGC
GCCAGCTACAGCGTGCGCGACGCGTCCGGCCGCACGCCGCTGCACCTCGCCGCCTACGACGACCAGCTGCCCGTGTTAAGGACCCTGCTTGAATTTATA
CCCCCGAGCGAAATTGATGTGATGGATGACGCTGGAAATACGGCACTACAAATCGTCTGTGGCGGCACGACCGTCAGGGAAAATTCTGTAGAAATAGCA
AGACTGCTGCTTGAAAAGAAGGCATACCCTCTGAAACACGAGGATTGCAACGAGTCCGCTTGGAGACTGGTCAGGAAGAAGCCGGAACTCAGAGAGTTA
ATGAAAGCTTACGTCACGTCCGCCGAGTGCATCGACGAAGACGACATTAAGTCTGAACCGGAGGATGATTTCGAGTCCGCTGATGAAGGCGAGTACTTG
GAGACGGGCCTGGCGGAGCTGAGCGCGTACAGCCGCGAGGTGGCGGCGCTGGTGGACGGCGCGGCGGCGTGGCGCGAGCTGGCGCGGCGCCTGCGCCTG
GACGCGCTGCTGGACTGGTGCGCCGCGCAGCCCAGCCCCACGCTCACGCTGCTCAAACACCTCAAGGAGTCTCGAGATGACATATCTTCAAAGTCTTTG
GCCTTGATTTTGGAAGACATGGGACAAATTGAAGCGGCACGTGTCATAAGGAGGTATATCGATTGA

>EU325551.1 [aminopeptidaseN5]ID:ACB54941.1
ATGGCTATGGAAGTAGACGAAAATACCATTCGAGTTTACAGTGACAGGGTTCCAAATGTCAATATATACGCTTCCGCCACATTGGCCACTGATGACACT
CATTTGCTAAGGATTAATTTGAATCAAACTATGACAATCCTCCAACCGCACACTATAGAGATTGAATACGTTGGCCATTACGCTGAAAATATGTTCGGT
ATTTACTTATCGAAATATGAAAACAACGGTCAAGAGCAGAGACTGATCACATCACAACTGCAGCCTACGTTCGCTCGTCGCGCGTTCCCCTGCTATGAT
GAACCGGCTCTCAAGGCCGTCTTCAGGACCACCATCTATGCACCAGCCTCTTACACCGTCGTCAGGAGTAACATGCCTTTGAGGACTGATCTGCTCAAA
GAAGACGTAGCCGGCTACGTGAAGCACGAATTCCAAGACACCCTCATCATGTCCACATATCTCATCGCGTACCTGGTCTCCAACTTCGTCGCCATCGAA
AACAACGTGAACCCTCTCTACCGCGTCCCCTTCAGGGTCTACTCCAGACCTGGTACTCAGAACACTGCTGAGTTCGCCCTGACCTTTGGACAGCAGAAC
ATGGCAGCTTTGGAGAGATACACTGAGTTCAACTATGAGTTCCCGAAGATGGATAAGGTGGCCGTACCTGATTTTGCTGCTGGTGCTATGGAGAATTGG
GGTCTGGTTATCTACAGAGAAGTGGCACTTCTAGTAACAGACGGAGTGACAACAACAGCAGTCAGACAGAATGTAGGCAGGATCATCTGTCACGAGAAC
GTTCACCAGTGGTTCGGTAACGAAGTGGGGCCTCTGTCCTGGACCTACACTTGGCTCAACGAAGGTGTTGCCAACTTCTTCGAGAATTTTGCTACTGAT
CTGGTGAGACCAGAATGGCGTATGATGGACCAGTTCGTGCTGGCTCTGCAGAACGTGTTCCAATCTGATGCTGTCGCCAGCGTCAACCCCATGACGCAT
GAGGTGTACACGCCTTCTCAGATCCTCGGCACTTTCAACGCTGTTGCTTATCAGAAATCTGGTTCCGTGATTCGCATGATGCAGCACTTCCTCACTCCT
GAAGTATTCAGACAAGGACTTGTCATCTACATACGCAACAACACACGTGACGCAGCCTCACCCCTGAACCTCTACGCTGCTCTCCAGCAGGCTCTAGAC
CAGTCTTCCCATAGCATCGGCTTCCCAGTCAACACCATCATGCAGCGCTGGGTCAACCAGGGCGGCTTCCCAGTCTTGACGGTCACTAGGAGCGCTCCC

ACTGCCCAGTCTATTGTTGTTGAACAGGAACGTTTCCTGACAGACAGAAGCCAGAGGCTCACAGACAGATGGCACGTGCCTATCAACTGGGTGCTATCT
ACCAACCCTGACTTCAGCGACACCAGTCCTCAAGATTGGGTGCCTCCTACCTTCCCCGCGAGGTCTTTCGATATTCCCGGATTGTCGAACGCTGAATGG
TTCATCATTAATAAGCAGCAGACAGGTTACTACAGAGTAAACTACGAGCCTTCGAACTGGGCGGCATTAGCCAGAGTCCTTAACAGTTCTCACGAGACC
ATCCACGTGCTTAACCGTGCTCAGATCTTAGACGACTCCTTCAATCTGGCCAGGAATGGTCGTTTGAACTACGAGTACCCCTTCACCATTTCAAGCTAC
CTGGTGAAGGAGAGTGACTACATACCGTGGGGAGCCGCCAACCCTGCCTTTACTTACCTCGACACCGTGCTCAGTTCTTCTCCTGCTTACGGCCTTTTC
CAGAGATATCTCCTGGACTTATCAGCTCCTCTTTACCAACAACTTGGTTTCGAGGCATCACAAGACGAAGAATTCGTGACTCCTTACCACAGGAATATT
ATTTTGGATCTGAACTGCCGTCATGGCAATCCTGCGTGCATAAGAACTGCCCAGACGCTGCTTGAAAGATTCAGAACTGACGAAAGCCAACCCTTGAAT
GCTGACATCCAAACCCTGGTCTTCTGCTCCGGTCTTCGTGGAGGCAGTGTGGAGAACTTCAACTTCTTATGGGACAGATACTTGGCGACCTCAGACTCT
AGTGAGCAGTCTATCCTGCTGAACGCTCTTGGATGCACTTCTAATGAAGAAAGGCGTGCTTTCTACATGAACCAGGTGATCAGTGACGACTCTGCAGTG
AGGGATCAAGACAGACACACCATCTTGGTGTCAGTCATCAACGCCAGCCCCAACAGTACTCAGGCAGCTTTCGAGTTCATTATTGAGAACTTCGCTGCT
ATTCAGCCAAGAGTACAAGGCTTGACTGGAACAACCAACATCTTGAACGCTCTCTCCAGAAGACTGACGAGCCAGGCTGATTATGATCAGGTCCTGGCA
TTTGAACAGCGTTACCAGAACATCTTCACAGCCGGTGAGCTGGCTTCCATTGCCGGTATCAAGGAGAATATTGCCGCCTCCATAACTTGGAGCACCCAA
AACGCACCGATCGTTGAAGCTTGGCTTAGAACGAACTATGGTGAAAGTGGAGCTACCGCTCTGGTTTCTGGCTTCCTAGTACTTATTTCTATCGTTGTG
ACGATATACAATCATTAA

>JF417984.1[heatshockprotein]ID:AEB26315.1
ATGCTCAAAATGCGGTGGAGTATTCTAGCGTTGGCCTGCGTGGTCGTGGCGGTTTGCGCCGACGACAAGAAGGATAAGGAGAAGGACATTGGCACCGTC
ATCGGTATCGACTTGGGTACCACATACTCCTGTGTGGGTGTGTACAAGAACGGACGCGTGGAGATCATTGCTAACGACCAGGGTAACCGTATCACCCCC
TCATATGTAGCCTTCACAGCCGACGGCGAGCGTCTCATCGGTGACGCCGCCAAGAATCAGCTCACCACCAACCCTGAAAACACAGTGTTCGATGCCAAG
CGTCTCATTGGTCGTGAATGGGGAGATGCTACAGTCCAACATGACGTCAAATTCTTCCCCTTCAAGGTTGTTGAGAAGAACAGCAAACCCCATGTTTCC
GTCATGACCTCCCAAGGCGACAAGATCTTCGCCCCTGAAGAAATCTCCGCTATGGTCCTCATCAAGATGAAGGAAACCGCTGAGGCTTACCTCGGTAAG
AAGGTGACCCACGCCGTCGTCACAGTCCCGGCTTACTTCAACGACGCTCAGCGTCAAGCCACCAAGGACGCCGGTGCCATTGCTGGTCTCCAGGTCATG
AGAATCATCAACGAGCCCACCGCCGCCGCCATCGCTTACGGTCTTGACAAGAAGGAAGGCGAGAAGAACGTGTTGGTATTCGATTTGGGTGGTGGTACC
TTTGACGTGTCTCTGCTCACCATCGACAACGGAGTATTCGAAGTAGTAGCTACCAATGGTGACACTCACTTGGGTGGTGAGGACTTCGATCAGAGAGTC
ATGGAGCACTTCATCAAGCTGTACAAGAAGAAGAAGGGCAAGGACATCAGGAAAGACAACCGCGCCGTGCAGAAACTGCGTCGTGAAGTCGAGAAGGCT
AAGAGGGCTCTGTCTTCCAGCCACCAGGTCAAGATCGAAATTGAATCGTTCTTCGAAGGTGAAGATTTCTCCGAAACTCTTACCAGGGCTAAGTTCGAA
GAATTGAACATGGATCTGTTCAGATCTACCCTCAAGCCTGTACAGAAGGTGTTGGAAGACGCCGACATGAACAAGAAGGACGTCGACGAGATCGTACTT
GTCGGAGGTTCTACCCGTATCCCCAAGGTTCAGCAGCTCGTCAAGGAATTCTTCAACGGCAAGGAGCCTTCCCGAGGTATCAACCCCGACGAAGCCGTC
GCTTACGGTGCTGCCGTCCAGGCTGGAGTGCTCAGCGGTGAACAAGACACTGACGCTATTGTGCTGTTGGACGTCAACCCTCTGACCATGGGTATCGAG
ACCGTCGGTGGAGTCATGACCAAGCTTATCCCCCGTAACACCGTTATCCCCACTAAGAAGTCCCAGATCTTCTCTACCGCCAGCGACAACCAGCACACC
GTCACCATCCAGGTGTACGAGGGTGAGCGTCCAATGACCAAGGACAACCACTTGCTCGGAAAGTTCGATCTGACTGGCATTCCTCCTGCACCTCGTGGT
ATTCCTCAGATTGAAGTAACCTTCGAAATCGACGCTAACGGTATCTTACAAGTATCTGCCGAGGACAAGGGAACCGGAAACCGTGAAAAGATCGTGATC
ACCAACGACCAGAACAGACTGACACCTGAGGACATTGAGAGGATGATCAAGGATGCCGAGAGGTTCGCCGATGAGGACAAGAGGCTCAAGGAGCGCGTT
GAGGCCAGAAACGAGCTGGAAAGCTATGCTTACTCCATCAAGAACCAGCTCCAGGATAAGGAGAAACTTGGTGCTAAGTTGAGTGATGACGAGAAGACT
AAGATGGAGGAGGCGATCGATGCCGCCATCAAGTGGCTCGAAGACAACCAAGACGTCGACTCAGAAGAATACAAGAAACAGAAGAAGTCCCTTGAGGAC
GTAGTCCAGCCCATCATCGCCAAGTTGTACCAAGGACAAGGTGGCGTGCCCCCGCCCGGCGCGGGAGGTGACGAGGAAGACTTCAAAGACGAGTTGTAA

>HQ011924.1[inhibitorofapoptosisprotein]ID:ADM32901.1
ATGTGGTCGTGTTTCTTATCTTGTGTACATACAAAAAATACTGGATTACAAATGGATATTACCAAAGTGGCATCCAATGGCCCCGCCTCACCATTAACG
CTATTCAAGAGCGCTCCGCGCGAGGCTAAGATTCGACCTTTGGTTGGGCCGCTAGTGCTGCCGACGCCGAGCTACGACTCAAACGCCGGCTCTCCGGCC
TCGTCACCCTCTACACCTTCCTGTTCCTCTTCATTCAGCATCGACAAGACCGACAACCATGACACGTTTGGCTTCAGCACCGATACAATTGATATGCGA
CGAGAAGATGAAAGAATAAAAACGTTCGAAAAATGGCCAGTCACATTTCTACCCGGAGAACAGCTCGCTCGGAATGGATTTTATTATCTTGGCCGCGGA
GATGAAGTACGCTGTGCATTCTGCAAAGTTGAAATTATGAGATGGGTCGAAGGCGATGATCCCGCCAAGGACCATCAGCGTTGGGCGCCACAATGCCCA
TTCGTTCGCAAGCTGGGCGGCGGCGCCAGCACGGACTCGAGCAGCTCGGGCCGAGACGAATGCGGCGCCCGCGCGGCCGCCACCAGCACCACGCCCCCA
CGCATGGCCGGCCCCGTACACCCACGCTTCGCTTCCGAAGCTGCGCGTTTGCGCAGTTTTAAAGACTGGCCGCGATGCATGCGGCAAAAACCAGAGGAA
CTTGCCGAAGCTGGTTTCTTCTACACCGGTCAGGGGGATAAGACCAAGTGTTTCTATTGCGATGGCGGTTTGAAAGACTGGGAAAATGATGACGTGCCC
TGGGAACAGCACGCGCGCTGGTTCGACCGCTGCGCCTACGTGCAGCTGGTGAAGGGCCGCGAGTACGTACAGAAGGTGATGTCCGAGGCCTGCGTGGTG
ACGGCGGCCGAGGCGGAGAAGGACGTGGCGCCGGCCCGGCCGCCCAGCCAGCCCACCACACCCAGCACGCAACCTGAAACGCCGGAAAACTCCGTAGAC
GATTCCAAATTGTGTAAAATCTGTTATGCAGAGGAACGCAACGTGTGCTTCGTACCGTGCGGACACGTGGTGGCCTGCGCTAAGTGCGCGTTGGCGGCA
GACAGGTGCCCGATGTGCCGCAGGACGTTTCAGAATGCAGTGCGATTGTATTTCTCATGA

>JN315687.1[dorsal]ID:AEO51736.1
ATGGCGCGCCGCGACCAGCCCTACGTAGAGATCGTCGAGCAGCCGGCCAGCAAGGCGCTCAGATTTCGCTACGAGTGCGAAGGCCGGTCAGCAGGTTCG
ATCCCCGGCGTGAACAGCACGTCAGACAACAAGACTTACCCTACTATTAGGATATGCGACTTCACGGGACAGGTCGTCATCGTGGTGTCCTGTGTCACC
AAAGATGAGCCTTACAGGGCACACCCCCACAACTTAGTGGGTCGTGAACGCTGCGAGCGAGGAGTCTGCACGATCCCGACCCTCATCACGAAGGAAACA
TGCGAGTACCAGTTCAAGAACCTCGGCATTCAGTGCGTGAAACGGCGTGACATTGCCGAGGCCCTCACAGTCAGGGAGAAGCTCAGAGCCGACCCCTTT
AGAAAAAACTTCGATCACAAGAACCACCCACAAAGCATCGACTTGAACGCAGTTCGGCTATGCTTCCAAGTGTTTTTGCCGGACGACTCGGGGCGGCTG
CGGCGGCCGCTGGCGCCGGTCGTCTCCGACGTCATCTACGACAAGAAGGCCATGAGCGACCTGCTCATCATGAGGTCCAGCCACTGTTCTGGTACCGCT
AGGGGTGGCACGCAGGTTATTCTGCTTTGTGAGAAAGTGACCCGAGAAGACATAGAAGTAGTGTTCTACCAGGAAGAGAACGGCATCGTGGTATGGGAG
GAGATGGCCATCCGCATCCTCGTGCACAAGCAGGTCGCCATCGCCTTCGAGACGCCCGCCTACAAGTACCCCAACAC
CACGGACCATGTTCATGTCCACTTCCAGCTGAAGCGTCTGTCAGACAACGCCCGCAGCAACTCCCTGCCGTTCGAGTACATCCCCGAGTTCACAGATGT
GAGTAATAAGAGGCGGAAGGTGGTAAGCGACATGTTACGAAACTACGAAATTGACAGAATCTATTCACCGGAGCAGATCAAGTCGGAACCTAGAGACCG
GACTCCGCCACACCATAACATTAGCAGTCCGCCACTCCACGGCTACGCGCCGCCCTACGAACAGAACTGGAACATAGACAACCTGCAGGGCGGCATGGC
AGTCCCGGGCCCGAGCCACGTCAGCCCGGCGGCCGCCCACGGCAACCTGTTCGTGCAGGAGTCGGCCATGTGGGCCAACCCGCAGTACGGCCAGCTGTC
CCCTGGCCATATGCAGCCTGGCATGCCCCAGAACTTACAGGTGTTACAGCCCAATATGCAGGCCATGTCTCCCAGTATGCAGCCTATGTCGCCCAACAT
GCAGCCGCTGTCACCCAACATGGCGTACGGAGAACGCATGTCGCCCAACGTGGCTGTCATGTCGCCCCACGGCCAGGTCATGTCTCCCCACGGCCAGGC
CCTGCCGCCCCACGGCCCCGGCATGTCCCCGCACGGCCCCGGCATGCAGGCCATGTCACCCCTCGGCCGCGTGTCGCCCAACGTGCCGCAGAACATGGG
CCACATCTCGCCTAACCTGCAGCAACAGCCTGCTTATGTACAGCAGGCTCCTCAGCACCAAGCGGCGGCTGGGTCTCTGATGGAGACGGAGACGCACAG
CACCACGTCCCTCAGCAACCTGCTGCTGGACCGCGGCGAGCACTCGCTGCTGCTGAACTCGGGCGAGCTGGCCGGACTGTCCGCGCTGCTCGGCGACCG
CGCGCAGGACTCGCAGCCCGCACTCGACTACTGA

>DQ458470.1[cytochromep4506B2]ID:ABE60885.1

ATGTGGGTGTTTTATCTGCCGGTAGTGATATCAGTGTTAGCCGTAGCGCTTTATTATTATTTTACACGGACATTTAATTACTGGAAAAATCGAAATGTT
CGCGGACCGGAACCTACCGTATTTTTCGGAAACTTGAAGGACTCAGTCTTAAGAAGAAAAAATATTGGCGTAGTCATGGAAGAAATGTACAACATGTTT
CCTGATGAAAAAGTTATTGGAATCTATAGGATGACCACACCATGTGTCCTTGTACGAGACTTAGATATTGTTAAACATATCATGATAAAAGACTTCGAA
GTATTCAGTGACCGCGGTTTAGAATTCAGCAAAGAGGGATTGGGACAAAACCTATTCCACGCTGATGGAGACACTTGGAGAACTTTGAGAAATCGATTT
ACACCAATTTTCACATCTGGAAAACTGAAAAACATGTTTTATCTTATGAATGAAGGTGCTGATAACTTTATTGACCACGTGAGCAAAGAGTGTGAAAAG
CATCAAGAATTTGAAATTCACACACTTCTTCAGACTTATACCATGTCTACGATCTCCTCCTGTGCTTTTGGAGTAAGCTACGACACCATCAGTGATAAG
CTAGATACTTTAGCAATTGTAGATAAAATTATTTCGGAACCAAGTTATGCTATAGAATTAGATATGATGTATCCGGGTCTCCTTCCAAAACTCAATCTT
TCAATTTTTCCTTCTGTTGTACATAAATTCTTCAAAAATCTTGTGAACACAATTGTCACTCAAAGAAATGGCAAACCTTCAGGGCGTAACGACTTCATG
GATCTCATACTGGAACTCCGTCAAATGGGAGAGATCACCAGCAACAAATATGGTAACAATATGTCAACTCTTGAAATAACAGAATCAGTTATGTGTGCC
CAAGCTTTTGTGTTTTACATCGCTGGTTATGAAACTAGTGCTACTACTATGGCCTACTTGACATATCAGTTGGCACTTAATCCTGACATACAAAATAAG
TTAATAGCAGAAATAGATGAAGCAATAAAAGCTAATGGTGGAAAAGTAACATACGACACCGTGAAGGATATGAAATACTTGAACAAAGTTTTTGACGAA
ACCCTTCGTATGTACTCTATAGTAGAACCACTGCAAAGAAAAGCCATAAGAGATTACAAGTTACCAGGAACAGACGTGGTAATTGAGAAAGACACCGTA
GTGCTAATATCTCCAAGAGGCATTCACTACGACCCGAAATATTACGACAACCCTAAACAATTCAACCCTGATAGATTTGATGCGGAGGAAGTGGGCAAG
CGTCACCCGTGCGCGTACTTACCATTCGGACTTGGACAAAGGAATTGCATAGGTATGCGGTTCGGTAGACTCCAGTCTTTATTATGCATCACCAAGCTG
CTGTCCAAGTTTAGATTGGAGCCATCGAAAAACACTGACAGGAATTTACAAGTAGAACCGTACCGATTCATTATTGGACCAAAAGGTGGCATCCGTTTA
AACATTGTTCCGAGAAAAGATGTATCTTAA

>GU564660.1[gene=FMO2][FMO2A]ID:ADH16752.1
ATGCTTCGGAAATACTCCATTGTTTTTATTGTGTTGTTACAAATATGTATAAGTTTTGGTGCTCATGTCAAGTCAAAACGCGTATGTGTGATCGGTGCA
GGGATAGCTGGTATATCCTCGGCCAGATATTTAAAAGAGGAAGGGATTGACTTCACCGTTTTTGAATCCACGCGATACATAGGAGGCACTTGGAGATAT
GATACTCACGTGGGTACAGATGAAAATGGTCAACCACTTCATACAAGCATGTACAAATACTTAAGAACAAACCTGCCAAAAGCCCCTATGGAAATGAGA
GGCTTCCCCCTGCCTGATTACCTGCCCTCGTACCCTACCGGGAGAGACTTCTACCATTATCTAGAAGAGTGTGTGGATCGTTTAGACATTAAAAAATAT
ATTAAGTTTTTACACGCCGTAGTTTCAGTAAGAAGGATAAACGAGGTGTGGAAAGTCAAATACGAACACGTTGTAACTAAGGAGACATTTGAGGAAGAC
TTTGATTACATCATTGTTGGGAACGGACACTTTAGCAAACCTAGCTATCCTAATATCCCTAGTGAAGATCTTTTTACAGGTAGAATAATCCACAGCCAC
GACTACAAAGCGCCTGAACCTTTCACAAATCGTCGCGTACTAGTCGTGGGCGCCGGTCCATCAGGCATGGACATCGGGCTTGAAGTAGCTGACGTGGCC
AGTGCTCTCATCCACAGCCATCACTCTAAGATCAATTGGACCACGCCTTTCCCTCCTCATTACCACAAGAAACCTGATATCAAGGAGTTCAATGAAACT
GGAGTCATATTCGAGGATGGTAGTTTTGAAGAGATTGATGATGTTATTTATTGTACAGGTTTCTACTACGACTTTCCGTTCTTGGATGAGTCAAGTGGC
CTGACCATGGAGCCCAAGAGTGTGGTGCCTCTGTACAGGTACACGGTGAACATCAACCAGCCCTCCATGTTCATCATGGGAGCCTTCATCAGAGCCTGC
TTAGTGGTCGCTTTGGATGCTCAGGCACGCTACGCAACAGCATATATTAAAGGGAACTTCAGTCTACCGACCCGAGACGAGATGATGCTGGAGTGGCAG
AAGAGAATGGACACCATCCGCTCCAAAGGACTGCCCACGTCTTACATACACATTCTCGGGGAAAAAGAGGACGAATTCTACGCGGAACTTACAAGAGAG
TCGGGTATCGAGAGAGTTCCTCCAGTGATGTTCAAGATCCGCACAATGGACACTGAAGCCAAGATAGAGAACTTATATACCTACAGAAACTACGCTTAT
ACCGTCATTGATGACCATACTTTTGTCAGGAAGGTAGAAGACCCAGCTATTAGGAAAGGCATGCATGAATATGGGTTAGCGTATATGAATTAA

>FJ997310.1[carboxyl/cholineesteraseCCE014a]ID:ADF43475.1
ATGGCAATGATATCAGCCGTCAACGACTTTTTAGACGATCTGCGAGGGGGCAGGATGACAGACGCACCGGTAGTGACCGTGGAACAAGGTCAGCTGCAA
GGCAAAGTGGTGAACAGCCCCACTGGAAGGCGTACTACAGCTTCCAAGGGATCCCTTATGCTAAACCTCCTCTAGGATCCTTGAGATTCAAGGCACCA
CAACCCCCAGAACCATGGGACGGCATCCGCGAAGCGACTTCCGAAGGGAGCGTAAGTGCACAAGTAGACTTCTTGGGATCCAAACAGTTCACTGGTGAC
GAGAACTGCCTCTTCTTAAACATTTCCACCCCCAACCTAGACGGGGAGTTCCTCCCAGTAATGGTATTCATCCACGGAGGAGGTTTCACGTTTGGATCA
GGCAATTCCGACTTCTACGGAGCTGATTATTTAGTAGAAAAAGATGTAGTTGTAGTTACAATAAACTACAGGTGTGGTGTCCTTGGATTCCTGAGTCTG
AACACTCCTGAAGTGCCTGGTAATGCCGGCATTAAAGACATGGTCCAAGCTATCAGGTGGATTAAGAATAATATTCACAATTTTGGAGGTAACAGCGGT
AATGTGACCATTTTTGGAGAAAGCGCTGGTGGAGCGGCCGTTTCTCTGTTAACAGCTAGTCCTTTAACTAAAAACTTGATCAGCAAGGCTATCATACAG
TCTGGAACTGCTCTATCAAACTGGTCCATACAGAGGAATCCTCTAGAATCGGCTAGAAACATCGCAAAGATCTTAGGATGCGAGTCGACTGATCCCGAA
GAGATTTTGGACTTCTTAAACGCTACATCAACGAAAGACCTGGTCGAGGCCCATCAGAAACTAAGTCCTGAGGAAGAATTCCCGGACACAATAAACGCT
TTTGGTTACGTTGTCGAAAAAGAATTCCCGGGAGTCGAGGCTGTCATAACAGAATCATTCTTAGACCTGTTAACATCTGGAAGGGTCGCAGACATCCCA
ATAATGATCGGTTCAACGTCCCTAGAGTTTACTTTAGAGAGGAAAACAGATGATTTGCAAGTTTTCATCCCAGACGAGTTGAATATCGAGAGGAACTCA
GAGGAATCGCTGGCGATTGCTGACCAAATTAAGACTTTATACTTTAAGGGTAGTCATACTGGGGTAGAAAGTCTACCTGAGTATTTCGAGCTCCTCTCA
GATGCAATCATTAACATAGACACTCACAGACATGTCCAATACTTGATTAATGTATCAAAGAAACCCATTTACTACTACAAGTTTGATTACGTAGGGGAA
TTGAATATTTCTAACAAGATTTTGAATAGTTTTGGTCTGAAAAGGGCAATGCATATGGATGAGTTAGGGTATCTGTTCAAGAATGACTTGCAGAAGGGT
GTGGAGCCTACTACCTTGGATGTGAAGATGAGGGAAAGAATGCTCAGGCTTTGGACCAACTTCGCTAAGAGTGGAAACCCAACACCCGACGAGAACCAC
TACTTAACCGTCACCTGGTTGCCAGTAACCAAGGACAACCTTTATTACCTGAACTTGGGTCAAGAACTATCTTTGGCAACCAACCCGGACAAAGAAAAG
ATGGAATTTTGGGATGAACTTTACAGCAAATACTACCGCAGCTGGGAGCAACCTAGATCAAGTGCTGTAGAAACGGCTGTCGTATATTCTGAACCTAAA
ATCGTTGAGTCGATCTCTGAAAGTGGGACGGTATACACTTATTCTGAAACGACGGAATCCCATCACACCTTCATATCTGAAAGCCACGAGATTATAACT
GAGAACAACCAAATCATTTCCGAAACACACGAAGTCATTACGGGTAATGACGACTCCGTTCCCCAAATACAGGAAGTCGTTACGGAAAATAATGAAGTC
GTTACGGAAAATAATGAAGTCGTAACGGAAAATAATGAAGTCGTTGCAGAAAATAATGAAGTCGTTCCGGAAAGAAATGAAGTCGACAACGTAGTTCAT
ATTAAGCCTGCTACACCCGAACCTGTTAAAATTGAAGATGTGGAACCGGTTGTAACTGGGGTTGTTCAGAACGGAGATCAGAAGCCTAAACCAAGGCTT
TCGAATGAAATCAAGATGGTGACTCGTTCGAATGGGGCTCCTAAAGATGTGATAAGGGCCAATGACCCCCCAGAAGATGATTTACCTAAAAATATTGGC
GTTAATAAGTTTGTCAACTTCTTCGAATCGCTTGGAGCTAAGAAGTAA

>FJ009448.1[molt-regulatingtranscriptionfactorHaHR3]ID:ACH86113.1
ATGAACAACAACCAGTTCCACGATTTGTTTGGGTCCCAGTGGCCTCCGGACCAGCACGGAGGTCACTCGTCGGCATCGACGATGCTGCACCAGTCCCAG
GGCCTGCCCCAGGGTATGCAGCTGAAGAGAGAACCTCATACGGATGTGCAGCCGGTGATGCACAACCAGATGGGCATGGACATCACGTCAGGGTCCGTG
GCGGACAGTACATCACCGCCGCCTGGCAGCAGTGATGGGATGTTCGGCTCGTCTATATCTGGCATGTTTATGGATAAGAAAGCCGCTAATTCTATAAGA
GCTCAAATCGAAATAATACCATGCAAGGTGTGTGGGGACAAGTCATCTGGAGTCCACTATGGCGTCATCACCTGCGAGGGGTGCAAGGGTTTCTTCAGG
CGATCACAGAGTACAGTGGTGAACTACCAGTGTCCACGGAATAAAGCCTGCGTGGTCGACCGGGTCAACAGAAATCGTTGTCAGTATTGCAGATTGCAG
AAATGCCTCAAACTTGGAATGAGCCGTGATGCGGTGAAGTTTGGACGGATGTCGAAAAAGCAACGTGAGAAGGTGGAAGATGAGGTGAGATTCCATCGC
GCACAGATGAGAGCTCAGACTGACACGGCGCCCGACTCAGTATACGACGCTCAACAGCAGACGCCGAGCTCAAGCGACCAGTTCCATGGTCACTACAAT
GGCTACCCGGGCTACGGGTCGCCGCTGTCCTCATATGGGTACAACAACGCTGGACCGGCGCTACAGTCCAACATGGGAGGCATACAGCCTCAAGCACCT
CAACAGCAGCCCTATGACGTGTCGGCTGATTACGTGGACTCCACCACGGCTTACGAGCCGAAACAGACCGGGGGATTCTTGGATCCTGATTTTATTAGT
CATGCGGAGGGTGACATCAGCAAAGTTCTGGTGAAGAGTTTGGCAGAAGCACACGCAAATACCAACCCCAAACTGGAGTTCATTCATGAGATGTTCAGA

AAGCCACAGGATGTCTCCAAGCTTCTATACTATAGTTCAATGACGTACGAGGAAATGTGGCTGGATTGTGCAGACAAATTAACTGGGATGATTCAGAAT
ATTATCGAGTTCGCTAAACTTATACCCGGGTTCATGAAACTGACTCAGGATGATCAGATTTTGCTCTTGAAATCTGGCTCGTTCGAGCTAGCCATAGTC
CGGCTGTCGCGACTGATAGACGTCAATCGTGACCAAGTGCTATACGGTGATGCGGTGCTGTCCATCAGAGAATGTGTGCATGCACGTGACCCCCGCGAC
GTGGCGCTAGTGGTTGGTATCTTCGACGCAGCGAAGACGATCGCTCGACTCAAACTCACTGAGACTGAACTGGCGCTATACCAGAGCCTTGTGCTATTG
TGGCCAGAGCGGCACGGCGTCCGCGGCAACCCTGAGATCCAGTGCCTGTTCAACATGTCGATGGCGGCGATGCGGCACGAGATCGAGACCAACCACGCC
CCCCTCAAGGGAGACGTCACTGTACTGGACACGCTGCTCGCCAAGATACCCACCTTCAGAGAACTATCGCTGATGCACCTGGAAGCGTTGTGCCGCTTC
AAGGCCGCCCACCCACACCACGTGTTCCCGGCCCTCTACAAGGAGCTGTTCTCACTCGACAGCGTCCTGGACTACACCCACGGTTAA

>GU230740.1[gene=HSP90][heatshockprotein90]ID:ADM26743.1
ATGCCCGAAGAGATGCAGACCGATGTCGCGGAGGTGGAGACCTTCGCCTTCCAGGCGGAGATCGCCCAGCTCATGTCCCTGATCATCAACACATTCTAC
TCCAACAAAGAAATCTTCCTCCGTGAGCTGATTTCCAACTCTTCTGACGCCTTGGACAAGATCCGGTATGAGTCACTCACCGACCCATCGAAGCTGGAC
AGCGGCAAGGAGCTGTACATCAAGATCATCCCCAACAAGAGCGAAGGTACACTTACAATTATTGATACTGGTATTGGTATGACCAAGGCTGACCTCGTC
AACAACCTTGGTACCATCGCCAAGTCGGGAACCAAGGCGTTCATGGAGGCTCTTCAGGCAGGCGCCGACATCAGCATGATTGGTCAGTTCGGTGTGGGT
TTCTACTCATGCTACTTGGTGGCTGACCGCGTGACCGTTCACTCGAAGCACAATGATGACGAGCAGTACATGTGGGAGTCATCAGCGGGAGGCTCATTC
ACAGTCCGCCCCGACCACGGCGAGCCCCTCGGTCGCGGTACCATGATCGTACTACACATCAAGGAAGACCTCACTGAGTATCTTGAAGAGCACAAGATC
AAGGAAATCGTTAAGAAGCACTCACAGTTCATCGGCTACCCCATCAAACTGATGGTGGAGAAGGAACGCGAGAAGGAATTGTCTGATGACGAGGCTGAG
GAGGAGAAGAAAGAAGATGAGAAGGAGGACGACAAGCCTAAGATTGAGGATGTCGGCGAGGATGAGGAGGAGGACAAGAAGGATAAGAAGAAGAAGAAG
ACCATCAAGGAGAAATACACTGAGGATGAGGAGCTTAACAAGACCAAGCCCATCTGGACCCGCAACGCTGATGACATCACTCAGGAGGAGTACGGTGAC
TTCTACAAATCCCTCACCAACGACTGGGAAGACCACCTGGCCGTCAAGCACTTCTCTGTTGAAGGTCAGCTGGAGTTCAGAGCACTTCTGTTTGTGCCC
CGCCGTGCTCCCTTCGACTTGTTTGAGAACAAGAAGCGCAAGAACAACATCAAGCTGTACGTGCGCAGAGTGTTCATCATGGACAACTGCGAGGACCTC
ATCCCTGAGTACCTGAACTTCATCAAGGGTGTGGTCGACAGTGAGGACCTGCCCCTCAACATCTCTCGTGAGATGCTCCAACAAAACAAAATCCTCAAA
GTAATTAGGAAGAGCTTAGTTAAGAAATGCTTAGAACTCTTCGAGGAGTTGGCTGAGGACAAGGAGAACTACAAGAAGTATTACGAACAGTTCAGCAAG
AACCTGAAGCTTGGCATCCACGAGGACTCTCAGAACAGGTCCAAACTGGCTGACTTGCTCCGCTACCACACATCTGCCTCCGGTGATGAGGCTTGCTCC
CTCAAGGAGTATGTATCCCGCATGAAGGAGAACCAGAAACACATCTACTACATCACTGGTGAGAACCGTGACCAGGTAGCCAACTCTTCATTTGTTGAG
CGAGTCAAGAAGCGTGGTTATGAAGTTGTCTACATGACCGAGCCCATTGATGAGTATGTAGTACAACAGATGAGGGAGTACGATGGCAAGACCCTTGTC
TCAGTCACAAAGGAGGGTCTGGAACTCCCTGAGGATGAGGAGGAAAAGAAGAAGCGTGAGGAAGACAAGGTCAAGTTCGAAGGCCTCTGCAAGGTGATG
AAGAACATCCTTGACAACAAAGTTGAGAAGGTTGTTGTGTCCAACCGTCTGGTTGAGTCACCATGCTGCATCGTCACTGCTCAGTATGGTTGGTCTGCC
AACATGGAGCGTATCATGAAGGCCCAGGCTCTCCGTGACACATCCACCATGGGTTACATGGCAGCTAAGAAGCACCTTGAGATCAACCCCGACCATTCC
ATTGTCGAGACTCTGAGGCAGAAGGCGGAGGCTGACAAGAACGACAAGGCTGTGAAGGACTTGGTCATCCTGTTGTACGAGACTGCGCTGCTGTCGTCT
GGCTTCACCCTGGACGAGCCCCAAGTGCACGCGTCCCGCATCTACCGCATGATCAAGCTGGGTCTTGGCATCGACGAGGACGAGCCTATCCAGGTGGAG
GAGTCGAGCGCCGGCGACGTGCCCCCGCTAGAGGGTGACGCTGATGACGCGTCGCGCATGGAGGAAGTAGATTAA

>AY661710.1[hexamerine]ID:AAT76805.1
ATGGGTCGTTTGGGGTTGTGTGTTCTGGCGCTTCTAGTGGCCGGGGGCCACTCGGACCCCGTGAGGAGCAACCTCTCGCAGAAAGCTGCGGACCCAGTG
TTCCTGCAACGCCAATTGGATTTGATGGTCTTGTACTTCCACCTTCTTGAACCGAATCATCTTGACTCTTGCCAAGCTATCGCTAAGTCCTGGAGTCTC
GAGAGGAACATCGAACATTACAGCAATGTGACTGCTGTAACTACTTACATCGAGATGTTGGAGCACCAGTGGATACTCCCACGTGCGGTACCCTTCTCG

CTTCTGCAAGCCGAGCACAGATTCGGAGCCGTCACCTTATACAATGTGCTTATCTCTGCTAAGGACTACGACACATTCTACAAGACCGCTGTTTACGTT
AGGGATCTCGTCAACGAGAACCTCTTCGCTTACGTGCTCAGTATAGCCATTCTGAACCGCCCTGACACCCAAGGCATTTACATCCCTCGCCTTCCTGAA
GTCTTCCCGTCATACTTCTACAACGGTGAAATTATGACAACCGCTCAGAGGATTAACACCCATGGCCAAAACATGGTTGAGCACTACCCTTCAACCTAC
AAATGGGACAACAATGTGGTGATCAGGTGGAATGCTACGATCTGGCCTTACTTAAGAAGCGAAACCATGCCTCTTGCTTACTTCACTCATGACTTCAGC
CTAAACACCTTCTACTACAACCTCCATCTTGCCCAACCCAGCTGGCTGCACAGTGAAGTTCTTCCAGTGAACAAACACAGGCGTGGAGAATGGTTCTGG
TTCTTGCACAAGCAGATCCTCACTCGTTACTACATGGAGAGACTGTCTAACGGACTCGGTGAAATCCCCGAGCTTGGCCACGAGATTGTTCAGTACGGT
TACGCTTCGGGTCTTTTGTACCACAATGGCGTGCCCTTCCCTGCAAGGCCCAACTACTTTAACTTGGATCAGCCCCAGCTCGTTAATGAAATCCAAGAA
ATCCTCGACTACGAGCGCCGTATTCGTGACGCCATCGACCAGGGTTACGTTGTTAACCACCTTGGTGAACACATTGACATCTGCGCTCCTGAGGCCATC
GAAATCTTGGGTAGCATTATTGAGGCTAACGTTGACTCTCCTAATGCCAAATACTACAAGGACTTCATCAGCATCTGGAAGAAGGTTTTGGGCGACTCT
ATTGTTCAAGACAACCAGTACCACAACAACTACGTCCCTCTGGTTGTTCCCTCGGTTTTGGAGCACTACCAGACTGCTTTGCGTGACCCCGCGTTCTAC
ATGATCTGGAAGCGTGTCTTGGGACTGTTCCAAATGTGGCAGGAGAAACTTCCTCTGTACAAGAAAGAAGAACTGGCTCTTCCCCAGGTGGCCATCCAG
AAGGTCGATGTAGACAAGCTGGTGACATACTTCGAATACACTTACTTGAACGTCTCTTCTCACCTGCACATGAACCAGGATGAAGTTAAGGGTTACTAC
GACCAAGTCAGCGTGTTGGTACAGCAACCCGTCTTGAACCACAAGAGGTTCCAAGTTCGCGTGAACGTTAAGAGCGAGGTCGCTAAGACCGTTCTCGTC
AAGTTCTTCTTGGCGCCCAAATATGACAGCCAAGGCTATGAGATCCCTCTTCACATCAACACCCAGAACTTTATGCAGCTGGATGAGTTCACGTATGAC
CTTCCTGCGGGCGAATGCACAATTACTCGTGATTCCATTGACACCTCTGGCAAGAAGTGGGTGTCCGGTATCGAGATATATGAGGCGGTTGAGAAGGCT
GTGCAAGGCAAGGGACAGTACACCATCGACCCGAACATGGAGAAACTCGCCGAACATCTTATGCTGCCTAAGGGTCGCGTCGGAGGCATGCCGTTCGTC
CTGATGGTCTACATCTCAGAGTACCACGCCCCGAAGGTTGCTCCCGAACAAGTCTCGTACCCAGCTTTGTCTCTTGGCCTGTCTCCCGTTATTCGCCAA
CTGACCGACGAGCCATTCGGTTTCCCAGTCAACAGGCCTCTTCACCCATGGCAGGTGGAGGGAGTCAAGAACTTGTACCTCCAAGATGTCTTGATCTAC
CACAAGCATACCCCCGAAATCGAGGTTCCCCACATGGAATAA

>JF727877.1[c-JunNH2-terminalkinase]ID:AEE81067.1]
ATGCCCCACGCGGCGCTCCCAGCCTCCGCCATGTCGGCCCCGCCGCGCCACCCTCACTTCTACACCGTCGAGGTCGGCGACACGCGCTTCACCATACTC
AAGCGCTACCAGAATCTGAAGCCCATCGGATCCGGAGCACAGGGAATAGTATGCGCCGCGTACGACACGGTGACGCAACAGAATGTCGCCATCAAGAAG
TTGTCGCGGCCCTTCCAAAACGTCACGCACGCCAAGCGCGCGTATCGCGAGTTCAAACTTATGAAACTTGTCAATCATAAAAATACAATCGGTCTTCTA
AACGCGTTCACGCCACAGAAGAGCCTGGAGGAGTTCCAGGACGTGTACCTGGTGATGGAGCTGATGGACGCGAACCTGTGCCAGGTGATACAGATGGAC
CTCGACCACGAGCGCATGAGTTACCTGCTGTACCAGATGCTGTGCGGCATCAAGCATCTGCATCTTGCTGGGATTATACATCGGGACCTGAAGCCGTCC
AACATAGTGGTGAAGAGCGACTGCACGCTGAAGATCCTGGACTTCGGGCTGGCGCGCACGGCCGGCACCACCCTCATGATGACGCCCTACGTCGTCACG
CGCTACTACCGGGCGCCCGAGGTGATCCTGGGCATGGGATACACGGAGAACGTGGACATCTGGTCGGTGGGCTGCATCATGGGCGAGATGATCCGCGGC
GGCGTGCTCTTCCCCGGGACAGACCACATCGACCAGTGGAACAAGATTATTGAGCAACTAGGCACTCCGTCGGCCGCGTTCATGGCGCGGCTGCAGCCC
ACCGTGCGCAACTACGTGGAGAACCGGCCGCGCTACACCGGCTACAGCTTCGAGCGCCTGTTCCCCGACATCCTGTTCCCGTCCGATAGCTCCGAGCAC
AACCGCCTCAAGGCGTCGCAGGCGCGGGACCTGCTGTCGCGCATGCTGGTCATCGACCCCGAGCGCCGCATCTCCGTCGACGACGCGCTGCTGGACCCC
TACATCAACGTGTGGTACGACGAGGGCGAGGTCAACGCGCCTGCGCCGGCGTCGTACGACCACTCGGTGGACGAGCGCGAGCACACGGTGGAGCAGTGG
AAGCAGCTCATCTACCAGGAGGTGGTGGAGTACGCAGCACCACCACCACCGCCGCCGCACCACCCGCCGCCCGACCACGCGCAGCCCGCCCTCACCACA
TAG

>EF600049.1[alpha-amylase]ID:ABU98614.1

ATGTTCCGGCTCATCCTTCTGCTTGCTGCCGTGTCTTTGGCACTGGCTTACAAGAACCCACACTATGCGTCAGGCCGTACAACTATGGTCCACTTGTTT
GAATGGAAGTGGGATGACATCGCAGCCGAGTGCGAGAGGTTCTTAGGCCCCAGAGGATATGGTGGTATCCAGATCTCCCCTCCCAATGAGAACTTGGCT
ATCTGGTCCGCTAACCGCCCCTGGTGGGAACGCTACCAGCCGATCTCCTACCGTCTCGTCACCCGTTCTGGTAACGAACAGCAGTTCGCTAGCATGGTG
CGAAGATGTAACGATGCTGGTGTCAGGATCTACGTGGACGCCATCATCAACCACATGACAGGCACTTGGAACGAGAACACCGGTACTGGTGGTAGCACC
GCCAACTTCGGCGACTGGCACTACCCCGCCGTGCCTTATGGCAGGAACGACTTCAACTGGCCTCACTGTGTCATCTCTGGCAGTGATTATGGCTGCTGT
CCTGATAGAGTACGTAACTGCGAGCTCTCCGGTCTTAAGGACTTGAACCAGGGTACTGAATACGTTCGTCAAATGATCGTCAACTACATGAACCATCTC
ATCAGCTTGGGTGTTGCTGGATTCAGAATTGACGCCGCCAAACACATGTGGCCCGGAGACATGCGCGTCATCTTCGACCGTCTACACAACCTCAACACC
GCCCACGGTTTCCCCTCCGGCGCTCGTCCCTACATCTACCAGGAAGTCATTGACCTCGGTGGCGAAGCCATCACTCGCGACGAATACACTCCCCTTGCC
GCAGTCACTGAATTCAAATTTGGAATGGAACTTAGCCGTGCTTTCAACCGCGGAAACCAACTCAGATGGCTGGTCAACTGGGGACCTGCATGGGGCCTC
CTTGCTTCAAACGATGCTCTAACCTTCATTGACAACCACGACAACCAAAGAGGTCACGGTGCTGGTGGCAACATCCTCACATACAAACAGGCTAAGCAG
TACAAGGGTGCCATCGCTTTCATGTTGGCCCATCCTTATGGCTGGCCTCAGCTTATGAGCAGTTTCGACTTCCACAACACTGAAGCTGGTCCCCCAATG
GACAGCAGTGGCAACATCATCTCTCCTTCTATCAACTCTGACAACTCTTGCGGTAACGGCTGGATTTGCGAGCACCGTTGGCGTCAAATCTACAGCATG
GTAGCCTTCAGAAACCGCGCCGGTAACTCCGCCATCAGCAACTGGTGGGACAATGGCAGCAACCAGATCGCTTTCTGCAGAGGCAACCAGGGCTTCGTC
GCTTTCAACAACGACTACTGGGACTTGAACCAGACTCTTCAGACCTGCCTCCCCGCCGGTACCTACTGCGACGTGATCTCCGGCGAGAAGAGCGGTAAC
AACTGCACCGGCAAGCGCATCACTGTCGGCAGCGACGGCCGCGCTAGCATCTCTCTAGGAGCCAATGACTACGACATGGTGTTGGCCATCCACACTGGT
GATGAATCGAGGCTGTGA

>JN315688.1[arylphorin]ID:AEO51737.1]
ATGAAGACTGTCTTGTTCCTAGCAGGGCTCGTCGCTCTGGCGACGGGCGGCGCCGTCCCCCACCATGAGCTCAAAACGAAGCCTGTTGATGCTCAGTTC
GTTGGCTACCAGAAGAAGATTCTGTCGCTCTTGGAACACTCTCAACAACTGGACCTGCACTCTGAATACTACAAAGTTGGCAAGGATTACAATGTTGAG
GCTAACATTGACAACTACACAAACAAGCAAGCTGTCCAACAGTTCCTCGAGTTGTACAGAACTGGATTCCTGCCCAAGTTCCTCAAGTTTTCAATCTTC
TATGAGAGGATGAGGGATGAAGCTATTGCCCTATTCCATCTTATGTACTACGCTAAGGACTTCGAAACCTTCTGGAAAACCGCCGCCTGGGCCAAGGTT
TGGCTGAACGAGGAGCAGTTCTTATATGCGTACTACATTGCTGTGGTCCAGAGGTCTGACTTGGATGGTATCGTTCTCCCAGCGCCTTACGAAGTTTAC
CCACAGTTATTCTTGAACAAGAACATTCTGACTAAGATGTCCGTCATCAAAATGCAGAATGGACTGTTCCAATCTGATTTTGCTGCTCAATACGGCATT
GTCAAGGAAAGCGACTACTACGTGTACTATGCCAACTACTCCAACGCGTTGGCTTACCCCAACCAGGAACAAAAACTGTCATACTTCACTGAAGATGTA
GGCTTGAACGCTTACTACTTCTACTTCCACTCACAAATGCCCTTCTGGTGGAAGTCCGAGAAATACAACCTATTGAAAGATCGTATGGGTGAAATTTTC
TTCTACTACTACCAGCAACTTCTGGCCCGTTACTCCCTGGAGCGTCTTCCTCATGGATTAGGCGACATTCCTGAATTCTCCTGGTACTCTGAATTCAAG
ACTGGTTACTACCCTCAATTGTCTGCAAACTTCTTGCCCTTCGCTCAAAGGAGTAACAACTACAATATCCACTCTGAGAAGAACTACGAATACATCCGT
TTCCTGGACACCTATGAAAAGACGTTCTTCCAATTCTTGCAGAAGGGAGAATTCAAGACCCCTGAGAAGGAAATGAACTACGTCGGCAACTACCGGCAC
ATGAACTCAGACCTGTACTCCGAGAAGAGCAACAAGGACCTGCACCAGTACTCTTATGAGATCATCGCCCGTCACGTGCTCGGCGGTAGCCCCAAGCCT
TTTGACAAGTACTCATTCATGCCCACCGCCCTTGACTTCTACCAGACTTCTCTCCGTGACCCCGCCTTCTACCAGCTCTACCAGAGAATCGTCGACTAC
CTCGTCGCCTACAAAGAATACGTCAAACCTTACTCTCACAACGACCTTCACTTCGTCGGTGTTAAGATTAATGACGTGAAAGTCAGTGAATTGGTTACT
TACTTCGATTACTTCGACTTTAACGCCACAAACAGCGTGTTCTACAGCCAGGAAGAGCTCAAAGCTTACCCAACAGAATTCATTGTTCGTCAACCTCGC
CTGAACCACAAACCATTCACTGTATCTGTTGACTTGAAGTCTGATGTAGCCTCTGATGCTGTTGTCAAGATCTTCATTGGACCCAAATACCACCCCAAC
GGATACCCAATCAACATTGAAGAGGACTGGATGAAATTCTACGAACTGGACTGGTTCGTACAGAAGCTTGCCCCAGGAGAGAACAAGATTGAGCGCAAG

TCCAGCGAATTTGCGTTCTTCAAGGATGACTCTATCCCGATCAACGAGATCTACCAGTGGTTAGACCAAGGCAAGGTTCCCTACGACATGTCTGTTGTG
CCCGACAACATGCCTAGGAGGCTGATGCTGCCGAAGGGTACCCCTGGTGGTTATCCATTCCAGCTGTTCGTGTTTGTGTATCCATTCAACGGAGTTAAG
AAGGGAGAAGACGTGTTCCAGAACTACCTTGCTGACAACAAGCCCTTCGGATATCCGTTCGACCGCCCCGTGCGGGAAGCTTACTACAGGCAACCCAAC
ATGTACTTCGAAGATGTACAGATCTACCACAAGGACGCGTATTTACCTTACGAGATGAACGTTCCCTCTTATTTCAGCCAGAAGAAGCAGTAA

>FJ997313.1[carboxyl/cholineesteraseCCE016a]ID:ADF43478.1
ATGACGTCAGTGATACATATCGTGTCACTTCTAGCAGTGTTGTCGGCGCCGGCGCTCGCCGTGCAAGTGAGAGTGAGCGATGGCTTGTTGGAGGGAGAG
CGACTGATCAACGAGTATGGCGGTGCTGAGTACTTCAGCTTTCGAGGAATCCCGTATGCGCAGCCACCTCTCGGAGATCTCAGATTCAAGGCTCCACAA
CCTCCGACCCCGTGGAACAATGTCCGCAGCGCCAAGGAGTTTGGTAACAATTGTCTGCAGTACGACTTGTTCATAGACAAAGGAAAGCGAAGTGGCGAT
GAAGATTGTCTGTATCTCAACGTGTACACTCCTGAGATCACTCCCAGCGAGCCCCTGCCAGTCATGGTCTGGATCCACGGAGGAGGCTTCGTCTCCGGC
AGTGGGGATGACAATGTATACGGACCTAAGTTCCTTGTGAGACACGGAGTTATTTTAGTCACGATTAACTACCGACTCGAAGTTCTTGGTTTCCTTTCC
TTAGACACAGAAGAAGTCCCTGGAAACGCAGGAATGAAAGATCAAGTGGCCGCGCTCAGGTGGGTCAATAAGAACATCGCCAACTTCGGTGGAGACCCT
AACAACGTTACCATATTCGGTGAAAGCGCTGGAGGGGTCAGTGTCTCTTATCAAGTAATCTCACCAATGTCCAAAGGATTATTCAAGAGAGCCATAGCT
CAGAGTGGTGTCAGTGTCGGTTATTGGGCGCAGTCATATAGGCCAAGAGAAAGAGGCTTCGCTTTGGCCAGGAGTTTAGGATTACATACTGACAATGTC
ACTGAAGTTTATGAGTTCCTGAAAGTTCAGCCAGCTGAATCACTCGTACAAATCAAGGCGGCAGTAACTTACTCCGAACACGAAAGACCTAACGTTGAA
GTTTACTTTAGCGTGACTGATGAAAAACAATTCGGAAACAATGAGAGATTCTTCTACGGAGATATGGTGAATGCAGTGTCAAAAAACGTCCACGAAGGC
GTAGATATTATGACAGGATACACGGCTGACGAAGGACTCATGGGTGTTGCTATCTTCGGTCAAATCAATTCAAGTTTAGAACAAGCTAGAAGTTTCCCA
CAATTCTTTGTATCCTACCCAATGTCGCTCAATTTGCCAGTTGATGATCAATTGGAACTTGGAATGAAAATCAGGGACTATTATTTTAAAAATCAGATT
TCAATACCTGATGATTGGGAAGGTCTATCAAGATACTACGGCATGGATATATTCTCATTCCCCACTGTGTGGTGGATTAGGCTAATCGCTCGCACGAAT
AAGAACAAAGCATATCTGTACAAGTTCACCGTCAAGTCCGAGCTGAACAAGGCTGCGCATATGATGGGATTAGCCGACATACTCGGAGACAGGCAGGTG
GTCGCTCACAGTGACGATTTGTCATACTTGTTCAGTTCGCGACACATGCCTACTTTTGACATGACCACGACCCCATTCTTATACATTGACCGTGTTTGT
AGGCTGTGGGTCAATTTCGCGAAATATGGGGACCCTACTCCTGACGCATCGTTGGGTGTGGAGTGGAAGCCGTACTCGCTGGAGAAGCAAGACTATCTG
GACCTCGGCAACCAGCTGGTGGCCGGGAACGAGCCAGATGCCGAAGAAATAAAGTTCTGGGAAGATACTCTGACTGAATTCGGACAGAAACTGTACTTG
GATGATGATGAGGATTCGGTCAACTCATCCCGGTCAGCACTTATCCCGCAGGATAAGTTGTCCGCATTAATATTATTGTTATTTTTCTTTGGCCAACTC
ATCCGGCTTTAA

>JF460008.1[Proteinphosphatase2reg-subunit-isoform ?]ID:AEB77782.1
ATGGCAGCAAGCGACTCTGGGACGGATGAGTCACTGTATCCTATTGCGGTGCTCATCGACGAGTTGAAAAACGAGGATGTGCAGCTTCGTTTGAACTCT
ATCAAGAAGTTATCCACCATCGCGTTGGCCCTCGGAGTGGAGAGGACCCGGTCGGAGCTCATCCCGTTCCTGACAGAGACTATTTACGACGAAGATGAG
GTTCTACTCGCCCTCGCCGAACAACTTGGAAACTTCATCAACCTGGTTGGTGGTGGTGAGTTTGCTCACTGTCTCCTCCCCCCATTGGAGTCTCTGGCC
ACCGTAGAAGAGACGGTAGTCCGTGACAAGGCCGTAGCCTCACTCCGTGCTGTGGCTGCTCATCACACTCCTCAAGCTTTGGAGCAGCACTTTGTACCG
CTGGTGCAGCGTCTCGCTGGCGGTGACTGGTTTACTTCCCGTGCGTCTGCTTGTGGATTGTTCAGTGTCTGCTACCCCCGTGTATCAGCGCCTGTCAAA
GCGGAACTACGCCAACATTTCCGCTCCCTCTGCCAGGATGACACCCCCATGGTCCGTCGCGCGGCCGCCTACAAACTGGGCGAGTTCGCCCGCGTCGTG
GAGGTGGAATACGTCAAGAGTGACCTGATACCTATGTTTGTTACGCTGGCTCAGGATGGGCAAGACTCAGTGCGTCTACTCGCAGCAGAGGCCTGCGCC
GCCGTAGCCGCCCTCCTGCCTCCCGAGGACATGGAGCAGCTGGTGATGCCCACAGTACGCGCGAGAGCTGGAGACACCTCCTGGAGGGTGCGGTACATG
GTCGCTGATAAGTTCGTGGAGCTGCAACAGGCAGTAGGACCGGAGCTGGCGCGGTCGGACCTGGCGCAGATCTTCCAGGCTCTGCTGAAAGACACCGAG

GCTGAAGTACGCGCCGCCGCCGCTGGCAAGGTGAAGGACTTCTGCATGAACTTAGACAAGGCGCATCAAGAGCACATCATCATGACAATGATTCTCCCG
CAAATCAAGGATTTAGTCTGCGACGCGAACCAACACGTCAAATCCGCGCTCGTTTCCGTTATTATGGGTTTGAGCCCCATTGTCGGTCGCCAGAACACC
ATCGAACATCTCCTGCCTTTGTTCCTCACTCAACTGAAGGATGAGTGCCCTGAAGTCAGGCTGAACATCATCTCCAACCTGGAGTGCGTCAACGAAGTC
ATTGGAATCCAGCAATTAGTGCAATCTCTCCTCCCCGCCATCGTAGAACTCGCTGAAGACACAAAGTGGCGCGTCCGTCTAGCGATCATCGAACACATG
CCTTTACTCGCTGGCCAGCTCGGCCAAGAGTTCTTTGATGAAAAACTCACCGGCTTATGCATGTCTTGGCTCATCGACCACGTTTATGCTATCCGTGAA
GCCGCCACCCTGAATCTTAAGAAATTAGTAGAACAATACGGATCACAGTGGGCTGAAACTAATGTAATCCCCAAAGTACTGGCCATGTCCCGCGAACAG
AATTACCTGCACAGAATGACTTACTTATTCTGCATCAATGTGCTGTCTGAAGTTTGCGGCAAGGATATCACTACTAGAGTACTCTTGCCCACTGTTCTG
TCCATGGCAGATGACAACGTTGCCAACGTGAGATTCAACGTCGCCAAGACTCTGCAGAAGATGGCGCCGTTCCTAGACCCCGCCGTCATCCAGCCGCAA
GTGAAGCCAGTTCTCGAGAAACTGAACGTAGATCCCGACGTGGATGTCAAGTACTTTGCCTCCGAAGCCATCGCCGGCATCGCTGGATAA

>AF222788.2[cathepsinB-likecysteineproteinase]ID:AAF35867.2]
ATGGCCGCTTCGCGTGCAACGTTTGTTGCGCTCGTGTGCGCTCTCGCGCTCGCCGCCGCCGATGTGCAGAATCCGCTCAGTGACGATTTCATCAATCTC
ATCAACACAAAGCAAAACTCTTGGAAAGCTGGTAGGAACTTCCCAGAGCACACGCCCTTCGCACACATCAAGAGATTAGCGGGTGTCCTGCCAGATTAC
CATTTGTCTAAATTAAGCAAAGTTGAACATGAAGATGAATTGATTGCGAGTTTACCGGAGAACTTTGACCCGAGGGACAAATGGCCGAACTGCCCTACT
TTGAACGAAGTCAGGGACCAGGGATCTTGTGGTAGCTGCTGGGCGTTCGGTGCTGTGGAGGCCATGACCGACCGGTACTGTACATACTCCAATGGAACT
CAACACTTCCATTTCTCCGCTGAAGATCTCTTAAGCTGCTGCCCAATCTGTGGTCTGGGATGTAACGGAGGTATGCCAACGTTAGCTTGGGAGTACTGG
AAGCATTTCGGGCTTGTGTCTGGTGGTAGCTACAACTCAAGCCAGGGCTGCAGACCCTACGAGATTCCTCCGTGTGAACATCACGTACCCGGTAATAGA
ATGCCTTGCAATGGTGATTCTAAGACCCCAAAATGCGAAAAAACCTGCGAATCAAACTACAATGTTGACTACCGCAAAGATAAGCGGTACGGCAAACAT
GTTTTCTCAGTGTCAAGTAAGGAAGACCATATCAGGGCTGAGTTATTTAAGAACGGCCCAGTTGAAGGTGCGTTTACAGTGTACTCGGACTTGCTGAAT
TACAAGACCGGTGTTTACAAGCACACTATTGGCGACGCTCTCGGTGGCCACGCTGTTAAGATCCTGGGCTGGGGCGTGGAGAATGGAAACAAGTACTGG
CTCATCGCTAACTCATGGAACAGTGACTGGGGTGACAATGGATTCTTTAAAATCCTTCGTGGTGAGGATCACTGCGGTATCGAAAGCTCTATAGTAGCC
GGTGAGCCAATGTTCGTTGAATATTAG

>JN399217.1[serine/threonineproteinkinaseAkt]ID:AEM63703.1
ATGGCGGAGGCGGCGCCCGGGAACATTGTGAAGGAGGGCTGGCTGCAGAAGCGCGGCGAGCACATCCGCAACTGGCGCGACCGCTACTTCATCCTGTTC
GACAACGGCGACCTGGTGGGCTTCAAGACGCAGCCGGAGCGGAACAACTACCGTGACCCGCTCAACAAGTTCACGGTGCGGGACTGCCAGATCATGGCC
GTGGACAAGCCGCGCCCCTACACCTTCACCATCCGCGGCCTGCAGTGGACCACCGTCATCGAACGCAACTTCTCCGTGGACAACGAGAAGGAACGCGAG
GAGTGGGTGGCGGCCATCCGCTACGTGTCGTCGCAGCTGAGCGCGGGCGGGCCCAGCGCCGCCGCGCTGCCCGAGAGCGACGACCGCGACATGGCGCAG
CTCGGCACCAGCTTCCGCGACCCGCGCCGCATCACGCTGGAGAAGTTCGAGTTCGTGAAGGTGCTGGGCAAGGGCACGTTCGGCAAGGTGGTGCTGAGC
GCCGAGAAGGGCACGGGCAAGCTGTACGCCATGAAGATCCTCAAGAAGAACCTCATCATACAGAAGGACGAGGTGGCGCACACCATCACCGAGAACCGC
GTGCTCAAGAAGACCAAGCACCCCTTCCTCACGGCGCTGCGCTACTCGTTCCAGACGGCGGACCGCGGGTGCTTCGTGATGGAGTACGCCAACGGCGGC
GAGCTGTTCTTCCACCTGTCGCGCGAGCGTTCCTTCTCCGAGGAGCGGACGCGCTTCTGCGGCGCCGAGATCGTGTCCGCGCTCGGCTACCTGCACGCC
GAGGGCATCATCTACCGCGACCTCAAGCTCGAGAACCTGCTGCTCGACAAGGACGGCCACATCAAGGTCGCCGACTTCGGCCTGTGCAAGGTCAACATC
ACGTACGGGCGCACCACCAAGACGTTCTGCGGCACGCCGGAGTACCTGGCGCCCGAGGTGCTGGAGGACACGGACTACGGGCCGGCCGTGGACTGGTGG
GGCACGGGCGTCGTCATGTACGAGATGGCGTGCGGCCGCCTGCCGTTCTACAACCGCGACCACGACGTGCTGTTCTCGCTGATCCTGAGCGAGGAGGTG
CGGTTCCCGCGGGCGCTGTCGGCGGCCTGCCGGGCGCTGCTGGCGGGGCTGCTGACCAAGGAGCCGGCGTCGCGGCTGGGCGCGGGCCCCGACGACGCG

CACGAGATCATGAACCACCCGTTCTTCGCGTCCGTCAACTGGGCCGACCTGGTCGCCAAGAAGATCCCGCCGCCCTTCAAGCCGCAGGTGGAGTCGGAG
ACGGACACGCGCTACTTCGACTCGGAGTTCACGGGCGAGTCCGTGGAGCTGACGCCGCCCGAGAGCGACTCCAGCCTCGCCAGGATACAGGAGGAGCAG
TTCCCGCAGTTCTCTTACCAGGACATCTGCTCGTCGGCACACTCGGCGCTGTCGCACCACTCGGCGCTGGCTGACAAGCGCCAGTAG

>JF417987.1[receptorforactivatedproteinkinaseC]ID:AEB26318.1
ATGACTGAAACTCTAAAGCTTAGAGGAACCCTCTGTGGCCACAATGGCTGGGTTACCCAAATTGCGACCAACCCTAAATACCCTGACATGATTTTGTCT
TCTTCCCGAGACAAAACCCTCATCGTATGGAAGCTGACCAGAGACGAGACTAACTACGGTGTCCCGCAGAAGCGTCTGTACGGTCACTCTCACTTCATC
TCGGACGTTGTGCTCTCCAGTGACGGAAACTACGCTCTGTCTGGCTCCTGGGACAAGACCCTGCGTCTGTGGGATCTTGCTGCCGGCAAGACCACCAGG
CGTTTCGAAGACCATACTAAGGATGTCCTCTCCGTGGCATTCTCAGTTGACAACCGTCAGATCGTGTCTGGCTCCCGAGACAAGACCATCAAGCTGTGG
AACACACTGGCTGAGTGCAAGTACACCATCCAGGATGATGGCCACAGTGACTGGGTGTCCTGCGTCCGCTTCTCCCCCAACCATGCCAACCCCATCATT
GTGTCCGCTGGTTGGGACCGCACCGTTAAGGTCTGGCACCTTACCAACTGCAAGCTGAAGATCAACCACCTTGGTCACTCTGGCTACCTGAACACAGTC
ACCGTCTCCCCTGACGGTTCCCTCTGCGCCTCCGGTGGCAAGGACATGAAGGCCATGCTCTGGGACTTGAACGATGGCAAGCATCTGCACACCCTGGAC
CACAATGACATCATCACATCATTGTGCTTCTCGCCCAACAGATACTGGCTGTGTGCTGCCTTCGGACCTTCCATCAAGATCTGGGATCTGGAAAGCAAG
GAGATGGTTGAAGAGCTCAGGCCTGAGATCATCAACCAGACCCAGACCTCCAAGTCAGACCCACCCCAGTGCTTGTCTCTGGCGTGGTCCACAGACGGT
CAGACCCTCTTCGCTGGCTACTCCGACAACATCATCAGAGTCTGGCAGGTGTCAGTCTCAGCGCGATAA

>EU770391.1[serineproteinase-likeprotein1]ID:ACI32835.1
ATGATAAAAGCCGTTCAAATCCTGCTGCTGGTAGCCGCAGCTCACTGCCAGAAGAACAATGGGGACCTGGACTCAGTTCTCAGCGAGATCTTCGGCCCC
CCTCCCGGCAGTTCCACTAGGAACCCGAGCGTACTGTCGACAGTAAGTCCCATTGGGGACAAGACTCAAGCGTCTACTATCGTACCACCGATAAATAAT
AATGCAGGTGAAGTGGACCTGTCGTGTACCATGCCTGATGGCCAGGAGGGCGAGTGTGTGAGCTATTATCTCTGCAACGTGAACAACAATACCGTCATC
GTTGATGGAGCGGGCGTCATCGACATTCGTGTCCGTGACGGCCCCTGTGAGTCCTACATTGACACCTGCTGTCTGGTGCCCGACAAGAGGCCCAAAGAC
CAGCCTATTAAACCTAAGCCTAAACCTGATGAGCCACAGCGTAAAGGATGCGGTTGGGCCAACCCAGATGGTGCTGATCTCCGCACAACCGGTGAAGTA
AATGGAGAGACCAAATTCGGAGAGTTTCCATGGATGGTTGCCATCCTGAAGATCGAACCAGTCAACGAGAATGACCCTGACGGTCAGAAACTGAACGTG
TACGTCGGCGGAGGCTCTCTCATCCATCCCAGCGTGGTGTTGACAGCTGCTCACTATGTGGCCAGTGCTAAAGCCTTGAGGATCAGAGCTGGCGAGTGG
GACACGCAGAACACCAAGGAGATCTACCCCTTCCAAGATAGGGATGTTGCCTCTGTGATTACGCATCAAGATTTTAACAAAGGAAACCTGTTCTACGAC
GTAGCAGTACTCTTCCTGGCACAGCCAGTAGACCTGGCTCCCAACGTAGGAGTGGCGTGTCTCCCACCACCCAGGGTCCGCCAGGAGGACGGCACCAGG
TGCTTCGCTACTGGTTGGGGCAAAGACAAGTTCGGCAAGGAAGGACGGTACCAGGTCATTTTGAAGAAGATCGAGCTCCCAGTAGTGAAACACGACAAA
TGTCAGACGGCCCTCCGTAAGACCAGGCTGGGCAGGTTCTTCGAACTACACTCCACCTTCATGTGCGCTGGCGGCGAGCCCGGCAAGGACACCTGCAAG
GGAGACGGCGGATCACCCCTGTCTTGTCCTATTGAGTACGAAAAGGACCGTTACGTTCAAAGCGGCATTGTCGCGTGGGGCATCGGCTGCGGAGAAGAC
GGTACTCCTGGAGTCTACGTCGACGTCTCAGTCGTGAGAGACTGGATCGACGACAAAGTGGCTGGCAAGGGCTACGACCCTAAGACTTACACGTATTAA

>FJ997318.1[carboxyl/cholineesteraseCCE017a]ID:ADF43483.1
ATGTTTGTTATAAGAACTATTGAAAAGTTAGTTGTTCGTACGAAGCCGGGTGCTATGCGGTTTTCGTCAGTTGGCAACTCACATGACAATCCAGTGGTG
ACCGTGAAACAGGGCAAATTGAAGGGAGCGGTTAAGAAAGGTATAGATGGGAGCCCTTACTACAGCTTTAAAGGAATCCGCTATGGGGAACCTCCCGTT
GGAGAGCTAAGGTTTAGGGCACCAGTACCAATAAAGCCATGGGATGGTATACGGATGCTACAGAACACGGGCCAGTGTGCCCTCAGTTCGATATGTCT
GTAGTAGACTTTGTGGAAGGGAGCGAGGACTGTCTCAGTTTAAACGTCTACACAAAGTCATTGCAACCTAACTCAAAACTACCCGTAATGGTCTACATA

CACGGTGGAGCATTTTTATCTGGATCTGGAAATGCTGAGACATATGGACCTGAATTTTTATTCCTACATGATGTAATTGTAGTCACTATTAACTACAGG
CTCGAAGCACTAGGTTTCCTCTGCCTAGACACTCCTGAAGTACCAGGTAACGCTGGTATGAAAGACCAAGTTTTAGCCATGCGTTGGGTTAAAGAAAAT
ATCAGCACTTTTGGTGGGGACCCTGACAATATCACGCTGTTTGGAGAGAGTGCTGGTGGAGCCTGCGTGAACTTCCACATGCTCTCACCGATGTCAAAA
GGACTGTTTAACAAAGCGATAGCCCAAAGTGGAGTTTGTTTAGAATATTGGGCCATAGCTCACCAACCTGTAGAGAGAGCGTTTAGATCTGGCAAAGTT
TTAGGCAAAGACACAAAGGATAAGAAGGAATTATTGGATCATTTAAGAGGATTGCCTGCCCTTGATTTAGCGAGGCTTACGGTCAAAACAAGAACTCCA
GACGAGAAATTTAGAGGACTACCTATTTATTACACTCCAACAGTGGAGAAGAAATTTGAAGGACAAGAACAGTTTTTGACTGAAGACCCTCTACAAATG
CTTTTGTCAGGCAAAGTGAACAAAGTACCATTCATGTGCGGGTACAATACTGCCGAAGGGATTTTATTAATCCCTGAACATAGGAAAAAGTTGGAAGTT
CTGAATAAGGAACCATCCTACCTTGTTCCAAGAGACATAGCAATGATATCTTCTAAGGAGAAACTGGAAGAGTTCGGCCAACGGGTCAAAGAGTTTTAT
TTCGGAGGTAAAGATGCCACTGAAGATGATGTTCAGCCTATAGTAGATGTCCATACAGATATTAATTTCACGTACCAAGCTCACAGACTGATGCATTTC
TACCATAAGATGGCGCCAACTTACATGTACCGATTTGACTACTGCACTAAAATGAACATGATGAGGAATATCTTTGGTGATGATGCAGTAAAAGGCGCA
TGTCATGTAGATGATCTATTTTATCTGTTCCACACACCAATGACTGAAAGTATTTATGAAGAGAATGAGGAGATAAGAAAGGCAGTGTATGATGTTACT
AAATTATGGACTGATTTTGCAAAGACTGGAAATCCTACACCAACAAAGGACGGCGTGGAATGGCGTCCCTACAACAGTTCAACCAAAGAGTATTTACTC
ATAGACAAAGAAACAAAAATGTCCAGTAACCTGGAGAAGGAAAGAGTTGAATTCTGGAACAAAATGTACGCTGACGCTGGTTTGCCTAGTATTACTAAG
TCGAACTTATAG

>FJ997303.1[carboxyl/cholineesteraseCCE002a]ID:ADF43468.1]
ATGTTTGCGTACGCTTTGTTTGTGTTGCTAGTTGCGTACGCGCATGGTTTGCAAGTGAGCACTATTGAAGGTACGGTGGAAGGCGGGAAGGCGTCAGAT
GGAGATTATTATGCTTTCTACGGTATACCTTACGCTGGGTGGACGACTGGTCGAAGCCGGTTTAAGGCGCCCGTTCTACCTTCATGGTATCCAGGAGTC
TTCCCCGCCACTGAGAACACCGTGATCTGCGCACAGCCAACAACCAGAGGTCTCGTCGGGTCAGAAAACTGCCTCACAGTTAACGTTTTCACGAAAAAT
GTTACAAATCCCAAACCAGTCATCGTTTGGATCAACGCCGAAGAGTACACCAACACGAACACAGAAGTCTTCTCATATCGAAGACTAGTCGAAAATAAT
GTTGTTTTCGTGTCTATGAACTTTAGACTATCGATTTTCGGATTCCTGTGCTTGAACGTCCAAGAAGCACCGGGTAACGCCGGTTTGAAAGACATCATT
CAAGGACTGAAATGGGTCAAGGACAATATTGGAAACTTTGGAGGGGACCCTAAGAACGTTATTCTAATGGGTCACGCATCTGGAGCAGCGATGGTAGAC
TTGATAACGCTTTCACCTCAAAGTGAAGGCCTCGTGCATAAAGCTATTATTCTAAGTGGATCAAGTCTGGCCCCATGGGCAGTATCTTATGACCCTATA
AAGTCTGCCCAGTTAGTGGGAGATAAACTTGGTTACGGAGGCAAGGCAAGAGATGACCTAGCAAAACATCTAGTCAAAACCGATATCAACCTTTTAAAT
ACGGCCTTGGACGATTTCAAGTATTTCAACAACACACCACAATTTGCACCATGTATCGAGGATCCCAAAATAAATCCTAACTTCACAGTCCTTTCTGAC
GCTCCTATCAACATTCTGAGATCTGGAAAATACCCTCAAATTCCTGTTATATACGGATATACAAACAGGGAAGGAACAATGCGTGCAGCGCAGGCAGAC
TATGGCAACTGGTTGAACTACATGCAGACAAACTTCACCAATTTCCTTCAAGTCGACATGGACTTCGGTAAAAATAGAACAGCTGTAGCAGCGGCTATT
CGTGATCACTACTTCGCTAGCAAGGCCATCAGCATGGAAACTATCGAAGACTTCCTAGATTATGAAGGAGACACTCTAGTTCTCGTGTCTGTAATCAAG
GCAGCTAAGGAAAGGGCTTTAACCTCTAAGAGTGAGGTCAGATTGTTGGAGTTCAGTTATTTGGGCACCATGAACTCTCACTGGGTGCACAATCAGATT
CCTTTGAGTGGAGCCAAACATGGAGCCTTCCTCAACTTTTTGTTTGGATATGATCTACGTCCTGTTGATGAGGCTGTTAGCAATTCGATCGTAAAGCGC
TTCGTTGATTTCGCTTATAACTGGAACAACACGACTGCCAGTGCGGGTAACATCGTCAACGTCAATTTAGTCAACTCAACAGCGACTGTCAATTCCACC
AGCACCAATGCCACAGCCAACATTGCTGGGACCAATGTGACTTCTTCTGCAGCAATGAGTAATGGAACTTCTATTGTCAATGGAACTGTCGTTGTCAAC
AATACAAATAATGCCATCCCTGTAACCAAAGGCAATGATTGGCCACCAGTTAGGCCGAACCTTACCAACTACCTCTTCTATGGAGGTAATGGGGCTCCT
ACTATTAACAATGGTTTCATCTACGTTGAAGAGGCGAGATTTAACCCTCATGTGCAAAGAATGTCTTTCTGGGACAATCTTTATGCTAAGTATTATGTT
GCTCCTACTTTAGCTAGTTCTTCTAGTGTTACAATTAGTTGTTTTATATCTGTGGTACTTTGTCAGTTAGTTGTTCTTTTGTTTTAA

>AY253868.2[ADP/ATPtranslocase]ID:AAP20934.2

ATGTCGAACCTCGCTGATCCGGTCGCGTTCGCGAAAGATTTCCTCGCTGGCGGTATCTCCGCCGCGGTCTCCAAGACTGCCGTGGCACCCATCGAGCGT
GTCAAGTTGCTGCTCCAGGTCCAGCACGTCAGCAAGCAGATCTCCGCCGACCAGCGCTACAAGGGTATCATCGATGCCTTCGTCCGCATTCCCAAGGAG
CAGGGTCCCCTGTCCTTCTGGCGTGGTAACCTCGCCAACGTCATCAGGTACTTCCCCACCCAGGCGCTGAACTTCGCCTTCAAGGACAAGTACAAGCAG
GTGTTCCTCGGAGGCGTCGACAAGAACACCCAGTTCTGGCGCTACTTCGCGGGTAACCTCGCCTCCGGTGGTGCCGCCGGAGCGACCTCCCTGTGCTTC
GTGTACCCGCTCGACTTCGCGCGTACCCGTCTCGCCGCCGACGTCGGCAAAGGCGACGGCCAGCGTGAATTCAAGGGTCTCGGCGACTGTCTGGGCAAG
ATCTTCAAGTCCGACGGTCTGACCGGTCTGTACAGAGGCTTCGGCGTGTCCGTGCAGGGCATCATCATCTACCGTGCCTCCTACTTCGGATTCTACGAC
ACCGCCCGTGGCATGCTGCCCGACCCCAAGAACACACCGCTCGTCATCAGCTGGGCCATCGCGCAGACTGTCACCACCGTCGCCGGTATCATCTCGTAT
CCCTTCGACACGGTCCGTAGGCGCATGATGATGCAGTCCGGCCGCGCCAAGGGCGACATCCTCTACAGAAGCACCATCCACTGCTGGGCCACCATCGCC
AAGACCGAGGGAACCGGCGCCTTCTTCAAGGGAGCCTTCTCCAACGTCCTCAGAGGAACTGGAGGCGCCTTCGTGCTTGTGTTATACGATGAGATCAAG
AAGCTTCTCTAA

>EF591060.1[gene=Cyp4s1][cytochromep450CYP4S1]ID:ABU88427.1
ATGCTGCTGTCACTAGCTCTGGTGGGCTTTATCACCTTCGTACTATGGAGACTCCTGGCCGAGGAGGAGAATCCTCTGGACAAGCTGCCAGGACCGAAG
AAACTGCCTATTGTAGGAAGTGCCTTCGAGTTTATGAGGATGAATCCACGAGACCTGTTCATAAAAGTCCGCCTCTTTGCTGAAATATACGGTGACAGA
TACCTTATCAAGATCCTGGGAAGACGGATACTGCATGTTCATAATGTCAAGGATGTTGAGGTCGTGTTAGCTCACTCSCGTAATATAAAGAAGAGCAAG
CCTTACACATTTTTGGAATCTTGGCTTGGTACAGGATTATTGCTGAGTAACGGTGCAAAATGGCACAAGCGCCGTAAGATCCTGACTCCGACCTTCCAC
TTCAACATCCTGAAGAGTTTCTCTATCGTCATGAAGGAGAGAAGCAAGGGCCTGGTGGAGAAGATCAAGACCTTGGAGAATACAGATGTCAACCTACTG
CCTTTGATCAGTGATTATACACTGTTTATTATTTGTGAAACTGCAATGGGCACTCAATTAGACTCCGACAAATCAGCAAAAACTCAAGAATACAAGAGT
GCAATCTTATCGATCGGAAACCTCCTCTTTGCCCGTCTAACAAGAGTGTGGCTTCACAACGAATACTTCTTCAAAATGCACTCCCTCGGTAGAAGATTC
CAGAAATGTCTAGAAAAGGTCCATTCCTTCGCTGACGATGTCATCATGGAAAGAAAGAAGAATTGGAAGCCAGGACAGAGTGAGTTTACAGAAGAAGAT
TCTGTGGGAGGCAAGAAGAGATTAGCTATGCTGGATTTGCTGTTGGAAGCGGAGAGTAAAGGAGAAATTGATTTAGAAGGAATTAGGGAAGAAGTTAAT
ACTTTTATGTTTGAGGGACACGACACGACAGCAATGGCGATAGTTCTTGGCCTGATGCTGATAGCAGACCATGAGGAAGTTCAGGACCGTATATTCGAA
GAATGCCAGAAGATCTTCCCTGATGCTGAGAGCACGCCATCAATGTCTGATCTGGCCGAAATGAAGTATCTGGAGGCTGTGATCAAGGAAACCCTGAGG
CTGTACCCTAGCGTGCCTTTTATTGCGAGAGCGATTACTGAGGATTTTATGTTGGATGATCTCCTAGTAAAGAAAGGTTCAGAAGTCTCTATCCACATC
TATGACCTGCACCGTCGAGCAGACCTGTTCCCCGAGCCTGAGGCCTTCAAGCCTGAGCGGTTCCTCAGCGGAGAAGCTATGCATCCGTACGCCTTTGTG
CCGTTCAGCGCTGGTCCTAGGAATTGCATAGGTCAAAGGTTTGCCATGCTAGAAATGAAATGTGTGCTGAGTGGAATTTGTCGTAACTTCAAGTTGCAG
CCGCGCGTGAAGGGTGCTCGGCCGGCGTTGTTAGCAGACATGCTGATTCGGCCCGCTGAGCCCATCTATGTGAAATTTATTCGTAGATAG

>DQ003275.1[gene=CYP9A17][cytochromeP450]ID:AAY21809.1
ATGATACTAGTCCTGGTCTGGGTGGCGGTGCTGATCGCCGTGGCGGTGCTGTACCTCCGCCAGGTCTATTCCCGGTTCTCTCGATATGGCGTGAAGCAA
TTCAGGCCGGTGCCCATCTTGGGCAACATGACCAGGATCCTCCTCAAACAAGATCACTTCGTAGACGATACCATGAGATATTATAACAGCTTTCCTGAA
GAGAGGTTTGTGGGCAAGTTTGAATTCATCAAGGAGTTGGTGGTGATCCGCGACATTGAGCTGGTGAAGAAGATCGCCGTCAAAGACTTTGAACACTTT
CTCGATCACCGCTCTATGTTCAGTTCCAGTGACTCTTTCTTTTCTAGAAACTTATTCTCTTTGAAAGGTCAAGAATGGAAAGACATGCGTTCGACTTTA
AGCCCAGCTTTCACAAGTTCCAAGATGCGTATGATGGTGCCCTTCATGGTGGAAGTTGGTGACCAAATGATGGCTGCGATTAAAAATAAGATCAAGGAA
TCTGGAAATGGCTATATAGACATCGAGTGCAAGGACCTGACGACTCGTTATGCCAACGACGTGATAGCCTCCTGCGCCTTCGGTCTGAAGGTGGACTCT
CACAACGAAACTGACAACGAGTTCTACACCATGGGCAAGCTGTCTTCCACCTTCAACTTCCGTCAGATGCTAGTGTTCTTCTTTATTGCAAATGCACCT
ACAGTTGCTAAGATCCTTAAACTGGATTTCCTTTCGGAAGCGGCGAAGAAGTTTTTCAGGAACTTAGTCCTTGATACCATGAAGAACCGTGAGCTGAAC

CATATCATTAGACCTGATATGATTCATTTGCTTATGGAAGCTAAAAAAGGTAAACTGACTCATGAGGAAATTAAGTCTAATGATGTAACTGCTGGCTTT
GCGACAGTTGAAGAATCCGCCGTGGGACAGAAGGAAATTACTAGAGTGTGGACTGATGAAGACCTCATCGCTCAGGCAGTATTATTCTTCATCGCTGGT
TTCGAGACCGTGTCTTCTGGCATGTCGTTCCTGCTGTACGAGCTGGCTGTGAACCCTGACGTCCAGGAGCGCCTGGCGCAGGAGATCAAGGAGAACGAT
GCCAAGAACGGCGGCAAGTTCGACTTCAACTCCATCCAGAATTTGCAGTACATGGATATGGTTGTGTCTGAACTGCTGCGCCTGTGGCCTCCTGGGGCA
GCACTGGACAGGATCTGCACTAAGGACTACAATTTGGGAAAACCTAACGACAAGGCCAAACATGATTTTATTGTCCGCAAAGGCACAGGTATCAGCATA
CCAGCTTTTGCCTTCCACCGAGACCCCCAGTTCTTCCCGAACCCAGAAAAGTTTGACCCTGAACGGTTCTCCGAGGAAAACAAGCACAACATCCAGAGC
TTTGCCTACATGCCCTTCGGTATTGGACCTAGGAATTGTATAGGATCAAGATTTGCTCTCTGCGAGATGAAGGTGATGGCATACCAGATTCTGCAGCAT
ATGGAGGTATCTCCCTGTGAGAGGACTTGCATACCAGCCAAGCTTGATACCGAGACCTTCAACATGCGACTGAAAGGAGGCCACTGGCTTAGATTCAGA
CCTAGGCAGTAG

>FJ997299.1[carboxyl/cholineesteraseCCE001h]ID:ADF43464.1
ATGAGGCGACTGTTGATAGTGTTATGTCTAGTAGTGTTAAGTAAAGGTGATGATAATGAGTGGCGGCAAGTGCGAACGGCGCAAGGACACGTGCGTGGG
CGCAGGGACCCAGAAGGTGGACTGTACGCTTTCTATAATATACCCTACGCTATCGCTCCACGGGGAAGAGAAAGGTTTAAGGCACCGTACCCTCCACCA
GTGTGGCTACAACCTCTGGACGCTGTAGATAAAGGCATCATTTGTATGCAAAGTCCTTCACCATTCATAGACACCAAGTCTAAGACTATGCAGGAAGAC
TGTCTTATTGCGAATGTCTATGTCCCCGATACTGAGGATAAAAATCTACCAGTCATAGTATACGTCCATGGAGGGGCGTTTGAATTAGGATATGGAGAC
ATGATAAAACCGACAAAATTAGTGAAGACTAAAAAAGTTATAGTTGTCAACTTCAATTATAGACTTGGAGTACATGGGTTCCTGTGCCTGGGCTCCAAC
ACTGCTCCTGGCAACGCTGGTCTTAAGGACCAGGTGGCTTTACTTCGCTGGGTAAAAAAGAACATCGCTAATTTCGGCGGTAACCCAAATGACGTCACA
ATAGCAGGCTATAGTGCTGGTTCAGTGTCTGCAGATCTGTTGATGCTCTCCAAATCTGCTGAGGGTCTGTTTAACAAAGTGATTCTGGAAAGTGGGGCA
AGCGTAGGCTCAATTGCAATTCAAATGGATCCTCTGGAAACCGCTAAAGCCTACGCAAAATTGTTGAACTTCAGTGACTTTGAAGACTTTCATGCCTTA
GAAGAGTTTTATGCAACAACTCCTTTAGATTCAATACTATTGAGTACATTTTCACAAAGAAAAGATAGTACCGTTTTCTTCTCGCCGTGCATTGAACGC
AAGGGAAAAGGGGCATTTCTGGATGATTCTCCAGTCAACATTATAAAGAACCGTAAATATAAAAAGGTTCCCATTTTGATAGGCACAAGCAACATGGAA
GGATCATTGCATATACCTATTTTTGCAAAATGGAAAGACGGTTTAAATCAGAAATTTTCAGATTTCCTCCCAGCTGACTTGCAATTCGAAAAAGATGAA
GAAAGAGATGAGATTGGTAAAAAAGTAAAAGAATTTTATTTTGATGACAAGCCAATAGCTGAAGAAACTATTCTAAATTCTCTTGAGTACTTCGGCGAT
CTTACTTTTGGCTATCCTACCTTAAAACACGTTAAACTACATGTAGAAGCCGGTCATGACCAAATATATTTGTATGAATATTCCTTTGTGGATGACAGT
ACTCCTTATGTGCCGTTCACGGAGGTTCGTGGAGCTGGCCACTGTGCTCAGACTACGGCAGTACTTGATGGGGGCGGCCCTATGGTGCCCGATGAAAGT
AATATATCTAAACAATACAAGCAAATTAAAGCCTCAATAAGAGAAATGTGGTTAAACTTCGCTACCAAGGGAAAACCTGTACCAGAGGAATCAAATCTA
CCTGCGTGGCCTGTAGCGAATGAAAATGGAACCCCATACATGACCATAGGAGATGTTATTGAACTGGGTGATGGAGTATACCTTGGGAAACGTGGACAA
TTCTGGGAAGACATTTACCAGAATTATTATAAAGCGCCGGTGCCTCCACCGCAGCCGCCACCGGACAAACACACAGAACTATGA

>EU528473.1[cathepsinL-likeprotease]ID:ACD40324.1
ATGTTTGGGCTGGTGGCTGTCACACAGGCTTTAAGTGTGAAAAGAGAAATTAAAGATAAGAAGCTCGAATCTAGCAAGAAATTGAAATGGCCTAGTCAA
TACCAGATAAAAGCGACTCGTTTGTCGCTTACTGACGATGTCATTGAAAACTATGCCGTATGGAGAACTTCAAAGCAATCCAGGATTGATTACAACGAT
GGGAACGTGAAATCTTTTGCGACATCTAAGAACAAACAGTTCCGTTATGGTGTGCAGTATGAGATCCATCCTGAAGCATCAAGGGACCAAGAAGTGGAA
GTGGTGTGCAATGCTATGGAAGGAGGGAGATACGACAGGGTAGAACCCTCAACTATTCTGCCTGATACTGATGAGTTTGAGTATGATGATGAAGAGGTG
ATGAATGACAAAGACTGCGTCAAATTCGTTAATAACGTGAAGGATGAACAGACTGACTCGGAGCAGACCCTCTGGGCCACTTACAACGAAGATGACAAA
GCTTGGGTTCCTGTCAGATACGAAGTTTTGGAGTTCAACTCCTGGTTGGGATCGAAGGACAAGCA

CGAAATCTGGGACTTCTATGATTACGAGAAAGCTTTCTCCTTCCGCAGCGTATTCGACGTTGAAGACTACGGTTGCAGTGACGCTGTTACTCACACCAA
AGACAGTGAAAATGTGACTAAACATTTGTTGTTCATGGATGCTGAGAACGACAAACATGTCGACCATGTCTTTAAATCATTCCAAAAGACCCACAACAG
GAAATACTCTGACGCCAACGAGCGCGCCATGAGGAGGAGTCTTGTCAAGAGGTCCATGAGGCAAGTCCTTGAGAACAACCGTCAAAAGAAAGGCTATAA
ACTTGCCATCAACCAATTCGCTGACAAGACCCCAGAAGAAATGAAAAAGTACATGGGTCTGCTCAGACGCCCTGAAGGCAAAGTCGGAACAATCGCTTT
CCCTTACGATGAAGCTAAGCTGAATGAAATCTCCAGTGGTATTCCTGATGAATATGACGCTAGGCTCGAAGGTCTTGTGTCGAACATCAAAAATCAAGA
AGAGTGCGGTTCTTGCTGGACCTTCGGAACCACAGCTGCAGTAGAAGGCGCCCTTGCGCGTATCAATGGCGGAAAACTGATGGCTTTGAGCAACCAGGC
TCTTGTCGACTGCGCTTGGGCTTACGGCGCCTATGGTTGCGAAGGTGGTACCGACAACGCTGCCTATGAGTGGATGATGGAATACGGACTGCCGACTGT
AGCGGAATACGGACAATACACTAACAAGGATGGCGAGTGCAACATCCTGAATATGACCGAGACTTTCCCCATCCGTGGCTTCGTGGATGTCACCCCACT
GAGCGTTGGAGCTTTGAAGGTTGCCCTAGTGAACCATGGACCTCTGTCGGTATCAGTCCACGTCACCGAAGCTTTCCTTCAATACTCTTCTGGAGTTTT
CTACGACACTGAATGCTCGCAACTTCCTAACGATCTGAACCATGAAGTGACCCCGGTAGGTTACGGCCAACGTGATGGAGACACCTTCTGGATCCTCAA
GAACTCCTGGGGACCCCACTGGGGTATCGGCGGCTACATGTACATCTCTTCGCGGGACAACATCTGTGGTGTTTCTACGGAACCCACCTACGTTGTCTT
CTAA

>EU629216.1[glucoseoxidase-likeenzyme]ID:ACC94296.1
ATGATTCTGGCGCAGCAAGATTGCGGCTGCGCTACAGTAGTAGAAGGTGCCAGTATCCTGAACTCCACAGCATGTAGCGGCACCTATCTGTTCATGGTA
CTCCTACAAGGGTACTTATGGGGCCGCTGCGAAATCGCCACGCCTTGCAAGAGAATCGAGTCCATAGATGAAACAGAGTCAGAATATGACTTCATAATA
GTGGGAGCTGGGTCTGCAGGATCCATTGTAGCTGGGAGACTGAGCGAAAATACTTCGTATAACGTCCTTCTATTAGAAGCTGGTGGGCCCGAGCCTCTG
GGTGCCCGTGTCCCATCATTTTACAAAACATTCTGGGGCCACGATGAGGTAGACTGGCAAGGGCGAGCTGTACCTGATCCCAACTTCTGCCGCGACCAA
GGAGAACTCGGGTGCCAATGGCCGCTAGGAAAAAGCTTAGGAGGATCTAGTCTCCTCAACGGTATGATGTACCACAAAGGCCACGCCGCAGACTACGAG
ACCTGGGTGGAAGAAGGTGCGGAAGGTTGGTCCTGGGATGAGGTCAAACCATTCATGGACTTGGCGGAAGGTAACAGACAAGTGGGAAGCCTCGTGGAT
GGGAAGTACCACTCCGAAACTGGACGCATGCCAATACAAACATTTAACTACCAGCCCCCGCAGCTTAGGGATCTGATAGAAGCAATCAACCAGACGGGA
CTGCCGATCATCACGGACATGAACGACCCGAACACACCCGACGGCTTTGTAGTAGCACAAACCTTTAATGACAATGGCCAGCGCTACACCACAGCCCGC
GCATACCTGGCTCCGAAATCCGAGCGACCCAACCTGAGCGTCAAACTCTATGCCCACGTGACTAAAGTCCTCTTCGACGGAAGAAGGCTGTGGGAGTG
GAATACGTCGACAAGAACGGGAATACGAAAACTGTCAAGACTACTAAAGAGGTAATCGTGTCAGCAGGACCATTGACCAGTCCTAAAATCCTGATGCAT
TCTGGTGTGGGACCTAAAGAGGTCTTAGAGCCATTGGGCATTCCCGTAGTAGCTGACGTGCCCGTCGGCAAGAGGCTGAGGAACCACTGCGGAGCCACG
CTGAACTTCCTCCTGAAGAAGTCCAACAACACCCAGTCTTTGGACTGGAGTGCAATGACTGACTACTTGCTGGAACTTGACGGGCCTATGAGTTCTACT
GGGCTTACTCAGCTCACTGGTCTCCTATACTCAAGCTACGCAGACAAGAGTCGCAAGCAGCCAGACCTACAGTTCTTCTTCAACGGCCTGTATGCTGAC
TGCTCCAAGACTGGAGTCATCGGAGAACCGGCTGAGGACTGCAGCGATGGTTACAAAATTTCAGCGAATGCCGTAGCCCTGCTCCCGCGCAGCGTGGGC
CACGTGACTATCAACTCGACCGACCCCTTCAAGTCAGCACTGTTCTACCCCAACTTCTTCTCTCACCCCGATGATATGAACATCGTGATGGAAGGCGTT
GATTACTTGCGCCAGATCTTCGAGAGTGAGGTGCTACAAGAGAAATACAAAGTAGAACTAGACCCGGAATACACAAAAGAGTGTGACGACTACGAAGCC
TGGTCAAGAGACTGGAAGGAGTGTATGATTCGCCTACACACTGACCCACAGAACCATCAGCTCGCTACCAACGCTATTGGCAAGGTCGTTGACCCGCAG
CTTAGGGTTTATGACGTCAAAAACTTGCGAGTATGTGACGCGGGATCCATGCCGTCACCGCCCACCGGCAACCCTCAAGGCGCCATCATGGTGGTCGCA
GAACGATGCGCACACTTCATCAAACAGACCTGGCAATAG

>JF417986.1[proteindisulfideisomerase]ID:AEB26317.1

ATGTTGCACACATATTTCTTGGGTATTTTACTATGTGTGGGGTCGGGTTTCGCCTTGTACGACTCGAGTTCCAGTGTCGTGGATCTGACGCCAAGTAAT
TTCGACAAACTAGTCACGGATAGTAATGAAGTGTGGTTGGTCGAGTTCTACGCTCCGTGGTGCGGCCACTGTAAGAACCTAGTTCCCGAATACAAGAAA
ACTGCCGATGCATTGAAGGGCATGGTCAAAGTAGGAGCACTGGATGCCGACCAGTACAAAAGCTTTGCCAAGAAGTATGGAGTCACCGGGTTCCCTACC
ATTAAGATCTTCACCGGCAGCCAGCACACTCCTTACAAAGGTTCAAGGACCGCGTCAGCTATGGTAGATGCCTGTCTCGAAGCCCTGAAGAACAAGGCA
TATGGCCGTCTTGGTACCAGACCTGAACGCTCATCAGAAAAGTCGGACTCTGGCGTGATCACCCCGACGGACGAGAACTTCCAAAAGCTGGTGCTGAAC
AGCGAAGACCTCTGGCTGGTGGAGTTCTACGCGCCCTGGTGCGGACACTGCAAGAACTTGGAGCCCCACTGGGCTAAGGCTGCTACCGAACTTAAGGGC
AAGGTGAAACTCGGAGCCCTAGATGCCACAGTACACCAAGCAATGGCTTCCCGCTTCCAAGTGCAGGGCTACCCGACCATCAAGCTGTTCCCATCAGGC
AAGAAGACCGCGGATTCCGCTGAAGACTACAACGGAGGCAGGACTGCTAGCGACATTGTAACTTATGCCCTTGAAAAGCTCGCTGAAAATGTGCCCGCT
CCTGAGATTGTTCAGGTCATAGACGAGGCGACAATGCAGGCCTGCAGCGAGAAGCCGCTGTGCGTGGTGTCCATCCTGCCGCACATCCTGGACTGCAAC
GCCGCCTGCCGCAACGAGTACCTCGCCATACTCGCCAGGCTCGGCGACAAGTACAAGAGCAAGATGTGGGGATGGGTGTGGGCGGAGGCTGGCGCACAA
CCAGCTCTGGAAGAAGCTCTGGAGCTGGGCGGCTTCGGCTACCCCGCATGGCTGTCGTCAACGCTAAGAGGATCCTTCTCCAGGGATCTATCATTCGGT
CGCGGCCAGACTGCCCCAGTTAAAGGCGCAGAGATGCCTAAGATCGTGACCACCGAACCCTGGGACGGCAAGGACGGTGAACTGCCACAAGAAGAGGAC
ATTGACCTCTCAGACGTAGATCTTGAGAAGGACGAGTTATAA

>GU937509.1[gossypol-inducedcytochromeP450]ID:ADW23116.1
ATGATCACTTCATTGCTACTAACGGCAGTTTTTGTGATAATCTTCACAATCTACCTCGTGTCCAAGAAAAAGTTCCAATACTGGGAGAAAAGGAAAGTA
CCACATTTACCTCCGGTTCCTCTCCTGGGAAATTTTGGGAACTTTATCCTGCAGAGGCAATTTCTTGGCTACACATTACAACAAATATGTGGAAAGTTT
CCCAACGTACCATACGTGGGTGCCTATTTTGGCACAGAACCTGCCCTGATCGTCCAAGATCCTGAACACATCAAGCTCGTCATGACTAAGGACTTCTAC
TTCTTCAGTTCCCGTGAGATATCTGAATATGCCGACAGGGAAAGGTTTACTCAGAACCTCTTCTCCACTTCCGGAAACAAATGGAAGGTGTTACGTCAG
AACCTGACTCCAGTGTTTACCTCCGCGAAGATGAAGAACATGTTCCATTTGATCGAAAAGTGTTCTCACGTGTTCGAAGATTTTCTCGATAAGGAAGCC
AAAAGCAACGAGGTCGAAATGAGGGCTCTTGTAGCGAGATACACTATGGACTGCATAGGAACCTGTGCATTTGGCGTTGAAACAAAAACCATGAATGTG
ACGGAAAATAATCCGTTTACAGCAGTAGGTAACAGCATTTTCATGTTAAGCCGGGTCCAAGGATTTAAATTTGTTTTGAGAGGTATCTACCCTTCACTT
TTCTACTTGTTGGGATTCAGAACTCTTCCACCAGAAGTTAATGCATTCTTCTCCAATTTAATGACTGGAGTTTTTAAGGGACGCAACTATACGCCCACA
TCTCGGAATGACTTTGTCGATTTCGTATTGAAGTGGAAACAAAATAAAACTATGACAGGGGACAGTCTGACTAACATGAAATATGATTCACAGAAAAAA
GTGACTTTAGAAGTCGACGATGATCTCTTAGTGGCACAGTGCTTTATATTTTTTGCTGCTGGATATGAAACTTCGGCCACCACTTTGAGTTTTACTTTG
TATGAGTTGGCGAAACACCCAGAAGCTCAGAAGAGAGCTATAGCCGAGGTGGACGATTATCTGCGGCGGCACAACAATGAGCTGAAGTACGAGTGTCTT
TCGGAGATGCCATTTGTAGAAGCATGCTTTGATGAGACTCTTCGTAAATATCCAGTTTTAAGTTTGTTAACTCGCGAAGTGGTAGAGGATTACACTTTC
CCTTCGGGATTGAAGGTAGAGAAAGGTCTCCGTATATTCCTGCCTCTGTATCACTTGCACCATAACCCGGAGTTCTTCCCGGATCCGGAGGAGTATAGG
CCTGAGCGGTTCCTGCCTGAGAACAAGGATAAAATAAAGCCGTACACGTACATGCCCTTCGGTGAAGGCCCGAGACTTTGTATTGGAATGAGATTCGCG
AAAATGCAAATGACCGCTGGAATAATAACTTTGCTGAAAAAATACCGTTTGGAACTGGCTCCAGGGATGCCCCAGAATATTGAATTTGAACCTAATTCT
TTTGTCTCGCAAGTTGCGGGAGGAATCAATCTGAAGATGATAAAAAGAGAAAGTTGGGAGGGAAGACTACTGAAGAACCTCGAAAAGGCATATTAA

>GQ411189.1[gene=cda1][chitindeacetylase1]ID:ADB43610.1
ATGGCGCGCTACGCCCGTGTCGCTACTCTGGCCGCGTGTCTCTTGTTCGCCTGCGCGGTTGCCGATGGGCACCGATGGCGGCGGCAGGCTGACGACCGC
GAGGCGCCGAAGAAAGACGACAGCTTAGAGGTAGAACTATGCAAGGACAAGGACGCCGGCGAATGGTTCCGCCTGGTGGCCGGCGAGGGCGACAACTGT

CGCGACGTCATCCAGTGCACCGCCTCGGGCATTCAAGCTATTCGTTGTCCAGCTGGTTTATACTTCGATATCGAGAAGCAGACCTGTGATTGGAAAGAC
GCTGTCAAGAACTGTAAATTGAAGAATAAAGAGCGTAAAGTAAAGCCTTTGTTGTACACGGAAGAGCCTTTGTGCCAAGATGGATTCCTCGCGTGCGGT
GACTCCAACTGTATCGAGCGCGGACTCTTCTGTAACGGCGAGAAGGACTGCGCCGACGGCTCCGACGAAAACTCTTGCGATATCGACAACGACCCTAAC
AGGGCTCCGCCTTGCGATGCGTCGCAGTGTGTGCTGCCTGACTGCTTCTGCTCTGAAGACGGCACGGTGATCCCCGGCGACCTGCCCGCCAAGGACGTG
CCCCAGATGATCACCATCACCTTCGATGACGCCATCAACAACAACAACATCGAACTTTACAAAGAGATCTTCAACGGAAAACGTAAAAACCCCAACGGT
TGCGACATTAAGGCCACATACTTTATTTCGCACAAGTACACCAACTACTCGGCTGTTCAGGAAACTCACAGAAAGGGACATGAAATCGCTGTACACTCA
ATCACGCACAACGATGACGAACGCTTCTGGAGCAACGCTACCGTTGACGACTGGGGCAAGGAGATGGCCGGTATGAGAGTCATCATCGAGAAGTTCTCG
AACATCACTGACAACAGTGTCGTGGGTGTGCGAGCGCCGTACCTCCGTGTCGGTGGTAACAACCAGTTCACCATGATGGAAGAGCAAGCCTTCTTGTAT
GACAGCACCATCACTGCTCCCCTGTCCAACCCGCCGCTATGGCCATACACTATGTACTTCAGAATGCCTCACCGTTGTCACGGAAACCTGCAGAGCTGC
CCCACCAGGAGCCACGCCGTGTGGGAGATGGTGATGAACGAACTTGACCGTCGTGAGGACCCCACCAACGATGAGTACTTGCCTGGATGCGCCATGGTT
GACTCTTGTTCTAACATTTTGACAGGAGATCAGTTCTACAACTTCCTCAACCACAACTTCGACAGACATTATGAACAAAACCGTGCCCCATTGGGTCTC
TACTTCCACGCTGCTTGGCTTAAGAACAACCCCGAGTTCTTAGAGGCTTTCTTGTACTGGATTGACGAAATTCTCCAAAGCCACAATGACGTATACTTC
GTAACAATGACTCAAGTAATCCAATGGATCCAAAACCCTCGCACTATCACCGAAGCTAAGAACTTCGAGCCCTGGAGGGAGAAGTGCTCCGTTGAAGGA
TACCAGGCCTGCTGGGTGCCCCACTCTTGCAAACTTACCTCCAAGGAGGTTCCCGGTGAAACCATCAACCTGCAGACGTGCGTGAGATGCCCTGTCAAC
TACCCCTGGCTGAACGACCCTACGGGTGACGGCCATTACTAG

>JF417983.1[gene=GAPDH][G3P-dehydrogenase]ID:AEB26314.1

ATGTCCAAAATCGGTATCAACGGTTTCGGCCGCATTGGCCGTCTGGTCCTCCGTGCCGCCGTCGAGAAGGGCGCCCAGGTCGTTGCCATCAACGACCCC
TTCATCGGCTTGGACTACATGGTCTACCTCTTCAAGTACGACTCCACCCGCGGTCGCTTCAAGGGCTCCGTTGACATCCAAGATGGCCACCTTGTGGTC
AACGGCAACAAAATCGCCGTCTTCTCCGAGAGGGACCCCAAGGCCATCCCATGGGGCAAGGCTGGTGCTGAATACGTCGTTGAATCCACTGGTGTTTTC
ACAACCACGGAGAAGGCCTCTGCTCATTTAGAGGGTGGTGCTAAGAAGGTCATCATCTCCGCTCCCAGCGCTGACGCACCCATGTTCGTCGTTGGTGTC
AACCTTGAAGCATATGACCCCTCTTACAAGGTCATCTCCAACGCTTCCTGCACAACCAACTGCCTCGCTCCTCTGGCTAAGGTCATCCATGACAACTTC
GAGATCATTGAAGGTCTGATGACCACTGTACACGCCACCATTGCCACCCAGAAGACAGTGGATGGACCCTCTGGCAAACTGTGGCGTGATGGCCGTGGT
GCCCAGCAGAACATCATTCCCGCCTCCACCGGTGCTGCCAAGGCCGTCGGCAAGGTCATTCCCGCTCTGAACGGAAAGCTGACTGGTATGGCTTTCCGT
GTCCCCGTCCCGAACGTGTCTGTCGTTGACTTGACTGTTCGTCTTGGCAAGCCCGCCACCTACGATGCCATCAAACAGAAGGTCAAGGAGGCCGCTCAG
GGTCCTCTGAAGGGCATCCTCGACTACACCGAGGAGCAGGTTGTGTCATCCGACTTCATCGGTGACTCTCACTCGTCCATCTTCGATGCCGCTGCCGGT
ATCTCTCTGAACGACAACTTCGTCAAGCTCATCAGCTGGTACGACAACGAGTTCGGCTACTCCAACCGTGTCATCGATCTCATCAAGTACATCCAGACC
AAGGATTAA

>AY373973.1[cathepsinL-likeprotease]ID:AAQ75437.1
ATGAAGAGTATAGCGGTGCTGCTGTGCGTGGTGGGCGCGGCGTGCGCGGTGTCGCTGCTGGACCTGGTGCGCGAGGAGTGGAGCGCCTTCAAGCTGGAG
CACAGCAAGCGCTACGACAGCGAGGTGGAGGACAAGTTCCGCATGAAGATCTACCTGGAGAACAAGCACCGCATCGCCAAGCACAACCAGCGCTTCGAG
CAGGGCGCCGTCAGCTACAAGCTGCGCCCCAACAAGTACGCCGACATGCTCAGCCACGAGTTCGTGCACGTCATGAACGGCTTCAACAAGACCCTCAAG
CACCCGAAGGCCGTGCACGGCAAGGGTCGCGAGTCCCGGCCCGCCACGTTCATCGCGCCGGCGCACGTCACCTACCCCGACCACGTGGACTGGCGCAAG

AAGGGCGCCGTCACTGAGGTCAAGGACCAGGGCAAGTGCGGTTCTTGCTGGGCCTTCAGTACGACTGGCGCGCTCGAGGGCCAGCACTTCCGCAAGACC
GGGTACCTGGTGTCGCTGTCGGAGCAGAACCTGATCGACTGCTCGGCCGCGTACGGCAACAACGGCTGCAACGGCGGCCTCATGGACAACGCCTTCAAG
TACATCAAGGACAACGGCGGCATCGACACCGAGAAGGCCTACCCCTATGAGGGCGTCGATGATAAGTGCAGGTACAACGCGAAGAACTCCGGCGCGGAC
GACGTCGGCTTCGTGGACATCCCTCAGGGTGACGAGGAGAAGCTGATGCAGGCCGTGGCCACCGTGGACCCGTGTCCGTCGCCATCGACGCCTCGCAG
GAGAGCTTCCAGTTCTACTCCGACGGCGTCTACTACGACGAGAACTGCTCCTCCACAGACCTCGACCACGGAGTGATGGTGGTGGGGTACGGCACGGAC
GAGCAGGGCGGCGACTACTGGCTGGTGAAGAACTCGTGGGGCCGCACGTGGGGCGACCTCGGCTACATCAAGATGGCGCGCAACAAGAACAACCACTGC
GGCATCGCCTCCAGCGCCTCCTACCCGCTCGTGTAG

>GQ896502.1[cryptochrome]ID:ADN94464.1
ATGCTTGGTGGAAGTGTCCTCTGGTTCCGTCACGGGCTGCGTCTCCATGACAACCCGTCTCTTCACTCTGCGCTGGAAGAGAAGGGATTTCCTTTCTTC
CCTATCTTTATCTTCGATGGAGAAACTGCTGGAACGAAACTGGTTGGCTACAACCGTATGCGGTATCTCCTGGAAGCCCTCGATGACCTGGACAGCCAG
TTCAAGAAGTTTGGCGGCAGGCTTATTATGCTGAAGGGCAAGCCTAATGTCGTGTTCCGAAGACTTTGGGAAGAATTCGGCATCCGTAAACTATGCTTT
GAGCAAGACTGCGAGCCAGTATGGCGGGCTCGCGATGACAGCGTGAAGAGCGCCTGCAAGGAGATCGGCGTGGTGTGCCGGGAACATGTCTCCCATACT
CTGTGGGAGCCCGAGACTGTCATCAAGGCTAATGGCGGGATTCCGCCGCTTACTTACCAGATGTTTTTGCATACTGTAGCAACTATTGGTGATCCCCCA
CGCCCAGTGCCCAATATCGACTTCACGGGAGTCAAGTTCGGCAGCTTGCCGGAGTGTTTCTACCAAGAGTTTACGGTCTACGATAAGACTCCTAAACCT
GAAGACTTAGGCGTGTTCCTTGAGAACGAGGACATCAGGATGATCCGTTGGGTGGGTGGCGAGACCACCGCGCTTAAGCAGATGCAGCAACGTCTGTCT
GTGGAATATGAAACATTCCTGAGGGGTTCGTATTTGCCGACTCATGGCAACCCTGACTTATTGGGACCACCGATTTCTTTGAGCCCTGCGCTGCGGTTT
GGGTGTCTTTCAGTGAGAAGCTTCTATTGGGCCGTACAAGATCTTTTCCGTCAAGTGCACCAGGGACGTCTCACCACTAATTCCGCTTCGCATTTCATT
ACGGGTCAGCTGATTTGGCGCGAATATTTCTACACGATGAGCGTGAACAACCCTAACTACGGCCAGATGGCTGGCAACCCCATCTGCTTGGATATCCCC
TGGAAGAATCCAGAAGGAGATGAGTTGCAGAGATGGGTAGAAGGCCGTACAGGTTTTCCGTTCGTGGACGCAGCTATGCGCCAACTGCGCACGGAAGGC
TGGTTACACCACGCGGCGCGTAACACTGTGGCCTCGTTCCTCACGAGAGGCACGCTGTGGCTGTCGTGGGAACACGGCCTTAATCATTTCTTGAAGTAT
CTGCTGGATGCTGATTGGTCAGTCTGCGCCGGCAACTGGATGTGGGTATCATCATCAGCGTTCGAGGCTCTGCTGGACTCGGGCGAGTGCGCGTGCCCC
GTGCGGCTGGGGCAGCGCCTCGACCCCAGCGGCGAGTACGTGCGCAGATACGTGCCCGAGCTAGCGCGCATGCCGGTGGAGTATATTTACGAGCCATGG
AAGGCGCCTATCGACGTACAAGAGCGCGCTACGTGCGTCATCGGCAAGGACTACCCGGCACCCGTCGTCAACCACTTGGTGGCTGCTCAGAGGAACAAG
AACGCTATGAAGTGGTTGAGCCGCACTGTCGCCGACCGACTGCAGAAGGACAGTTGGCTCGACATAGTCGGGGAACTCCGTCATATGTTACAAAAGGCG
CCTCCTCACTGCTGCCCGTCCTCAGAAGACGAGATCCGACAATTCATGTGGCTCAACGAGTAA

>GQ904195.1[beta-1,3-galactosyltransferase]ID:ADK56127.1
ATGAGACGAGGAAACTTTAAATATTTATTGTGTGCGTGTGTGATGGTGTGCATATACTATTTTTTTGGTGTTAGTGACTATATACATTCTAAAAGCTTC
GACGATAATTTCGATTACCCTCTTAATATAAATATTAAAAGCATCGTGGACGATGTGTTATCAGGCAAAAAGGTAACAGTGACTCCTATCAATTATTAC
CCGTACAGGTTTCTAACAAACTCGGGAAAATGTTCTACCACACACAAATTAGACCTGTTTATCGTGGTAAAATCAGCGATGGACCATTTCGGTCACAGA
GACGCCATAAGGCAAACATACGGCCAGGAGAATGTGCCAGGACGCACTGTAAAGACCTTATTCTTCTTAGGAATAGATGGCAAACAGAAGTCATCGCTA
CAGAAACAAATAGACAAAGAAATGGCCGACTTCAAAGATATAATACAGATGGACTTTATCGACAACTACTACAACAACACTATAAAGACTATGATGTCG
TTCCGCTGGGTGTACGAGCACTGTCCCACTGCTGACTACTACTTGTTCACCGACGACGACATGTACATCTCTGTGCACAACCTGCTGGTGTACATACAC
GACCGCGAGGCGACACGCAAGCCGGAGCCAGCGGGCTTTACTACGAACTACAAGTCCATCACTGACACTGCGGAGACGGACAGCGACTTCCTCTACACT
GGTTACGTCTTCAATTCAGCTCCACAGAGGTTCAGGTCGAGCAAGTGGAGAGTATCCCTTGTAGAATACCAGTGGGACAGGTGGCCCGCCTACGTCACA
GCCGGCGCTTACGTCCTGTCTAACAAAACTTTAAAGGTAATGTACATAGCTAGCCTTTTTGTCAAACATTTCCGATTCGATGATATATATTTAGGTATA

GTGGCTAAAAAGGTCGGTATTGTACCGAAGCATTGTCCTGATTTCTATTTCTACAAGAAGAAATTTAGTCCGGACGGTTACAAGGACGTTATAGCGTCT
CATGGTTACGAAGACCATGCGGAACTGATCAGGGTGTGGTGCGAGATGAATAGTCTTTAA

>EU526835.1[eukaryoticinitiationfactor5C]ID:ACD74811.1
ATGAGTCAGAAGGTAGAAAAACCAGTATTATCGGGTCAACGGATCAAGACCAGAAAAAGAGATGAGAAAGAGAAGTACGATCCGAACGGTTTCCGCGAC
GCGCTCGTGCAAGGTCTGGAGCGCGCCGGCGGCGACCTGGACGCGGCCTACAAGTTCCTAGACGCGGCCGGCTCCAAGCTCGACTACCGGCGATATGGC
GAGGTCATATTCGACGTGCTTATCGCTGGGGGGCTGTTGCTCCCCGGCGGGTCGGTGTCGATGGACGGCGAGTCCCCCAAGACGAACACCTGTATTTTC
ACCGCGAACGAAGATATGGAGACCATGCGAAACTTTGAGCAGGTATTTGTGAAGCTCATGAGACGTTACAAATACTTGGAAAAGATGTTTGAGGAAGAA
ATGAAAAAGGTGCTTGTGTACCTAAAAGGTTTCGAACCTCTACAGCGCATAAAGCTTGCTCGTATGACTGCACTATGGATCGGCAACGGCTGCGTGCCC
CCGTCGGTGCTGCTGGTGCTGGTGAACGAGCACTTGCTGAAGGACAACCTGGCGCTGGACTTCGTGCTCGAGGTGTTCGCCACCGTCAAGGCGGAGCGT
GGAGTTACCTCGCTCGTCACGGCCCTCAAGCGTGGACAGCTTGAAGGCAGGCTATTGGAGTTCCTGCCGCTGAACCGTCGCAGTGAGGACGTGCTGGCG
TCTTCGTTCGCTTCCAGAGGGCTCGGCGAGTTGCTGCGCCTGCATCGTGCACAGGCCTCGCAGGAGGCACGTCGCGAGCTGACGCAGGCGCTGCTGGAC
GAGCTGGCGGAGGAGAAGCCCATCCGCGACCTCATCCAGGAGCTGCGCGACATGGCCGCCAAGCACGCCATCCCCGACCATGAGGTCGTCGCCATTATA
TGGCAATGTGTGATGTCCCGTGGTGAGTGGAATAAGAAAGAGGAACTGTTAGCAGAACAAGCGGCTAAACATTTGCGCCATTACACGCCGCTGTTGGCT
GCCTTTGCGCAGTCGGCAAAGGCTGAGATTGCGTTATTAACTAAGGTGCAAGAATATTGTTACGAGAACATGAACTTCATGCGCGCGTTCAGCAAGCTT
GTCGTGATGCTGTACAAGACCAACGTGCTGTCGGAGGAGGTGATCCTCAAGTGGTACCGCGAGCCCAACTCCAGCAAGGGCAAGATGATGTTCCTCGAC
CAGATGAAGAGGTTCGTCGAGTGGCTACAGAGCGCCGAAGAAGAGTCCGAGAGTGGCGAAGAAGAAGATTAG

>HQ190046.1[NADPHcytochromeb5reductase]ID:ADO08221.1
ATGAGTAACGTAGAAGTGGCTGTGGATGACGCGTTTGGAATCCTTACAGTTTTACCCATAGTGGTTGGCGTTAGTGCCGCAGTGGTACTGGTGTCCGTC
ATTGCCAACTGGCTCTGGGGCAAGAAAGACAAGAAAGCAGCTCCTAAGAAGAGTTCCCAACTAATAACATTAGTAGACCCTAATGTTAAGTATGCTCTT
CCACTCATAGAGCGAGAGGAGATCAGTCATGACACAAGGAGGTTCAGATTTGGACTGCCTTCATCGGAGCATGTTTTGGGCTTGCCCATCGGCCAGCAC
ATTCACTTGTCGGCGAAGATTGATGACGACCTCGTCATCAGATCTTACACCCCTGTCTCAAGCGACGAAGAGAAGGGATATGTCGAACTTGTTATTAAG
GTATACTTCAAAAATGTCCACCCTAAATTCCCCGATGGTGGTAAAATGTCTCAACACTTGAACAGCCTGAAAATAAATGACACAATTGATGTCCGAGGA
CCCTCAGGCAGGTTGCAGTATGCAGGCAACGGCTTGTTCCTCATCAAGAAAATGAGGAAAGACCCACCCGTAGAATTGCGGGCGAAAAAACTCAACATG
ATCGCAGGTGGTACTGGCATCGCGCCAATGCTACAGCTCATCAGACATATATGCAAAGATGCGAGTGACCCCACAGAAATGCGACTGTTGTTTGCCAAC
CAGACTGAAGAAGACATCTTGTTGAGAAACGAACTGGAAAAGTACCAAGCTGAGCATCCCGAACAGTTCAAACTGTGGTACACTCTTGACAGGCCCAAT
GAAGGATGGAAATACAGCGTCGGTTTCATCAACGATGAAATGATCAAGGAGCACTTGTTCGCACCAGGGGATGATGTTTTAGTGCTAATGTGTGGACCT
CCCCCAATGATCAACTTTGCTTGCAACCCCGCCCTTGAGAAACTCGGCTATCCTGAATCACAACGTTTCGCATACTAA

>EU327674.1[microsomalcytochromeP450]ID:ABY47596.1
ATGATAGCCCTACTATGGCTGGCGGTGCTCGTCGCAGCTCTGACGCTGTACCTGCGCCAAGTCTACTCCAGGTTCAGCCGCTTCGGAGTCAAGCACTTC
GAGCCAGTCCCGCTGGTGGGCAACCTGAGCACCGTGCTGATGCGCAAGGCACATGCTTCTGAGGACTTCAACAATTTGTACCAGGCTTTCCCTGGAGAG
AGGTTCGTGGGCCGTTATGAGTTCTTGAGGAACATCGTGATGATTCGTGACCTGGAGCTGGTGAAGAGCATCACCGTGAAAGACTTTGAACACTTCATC
GATCACCGCATGTTAGCTGATGCTGACGTGGAGCCTCTGTTCGGCAGGAACTTGTTCTCCTTAAGAGGGCATGAATGGAAGGAGATGCGTTCCACGCTG

AGTCCAGCGTTCACCAGCTCCAAGATGAAGGCGATGGTGCCTTTCATGATGGAAGTCAGCGAGCAGATGATCAACTTCTTGAAAATGCAGATCAAGGAA
TCTGGAGGAAAGCATGCAGATATTGAATGCAAAGACCTGGTGACTCGCTACGCCAATGATGTGATAGCCTCATGTGCCTTTGGTCTGAAGGTAGACTCA
CACAATGATAGAGAAAACGAGTTCTACTCCATCGGCACTGAAACTGCTAACTTTGACTTCAAGAAGATGCTGGTGATCTTTGGGTATGCTTGCTTTCCT
GCTGTAATGAAGAAATTTAACGTGAAAATGTTTTCGGAACTCATAGTGAATTTCTTCAAAAACATAGTAATTGGTACTATGAGGAACCGTCAAAAGAAC
AATATTTTAAGACCTGACATGATACACCTCCTCATGGAAGCCAAAAAAGGAAAACTAACGCATGAAGAAAAAGCTGCTGAAGCCAACACTGGGTTTGCA
ACAGTAGAAGAATCGGACATTGGAAAAGTAACTGTTAAAAAAGAATGGTCAGAAGACGACTTGACAGCTCAAGCGGTACTGTTCTTCGTTGCTGGCTAC
GAAACTATCTCATCAGCTATGGCATTCCTCATCTATGAGCTGGCAGTCCACCCTGAGGTACAGGAGAAACTAGCGAAGGAGATCAGGGAACATGACGCT
AAGAACGGCGGCAAGTTTGACTTCAACTCCATACAGAACATGCCTTACTTGGATATGGTTATTTCAGAGGTGCTGAGATTATGGCCCCCAGCAGTAGGT
TTGGACAGAGAATGCTCAAAAGATTACAATTTGGGAAAACCTAATGACAAAGCAGAGAAGGATTACATCCTCCGCAAAGGTGAGGCTTTGGTGATCCCG
GTGTGGTCTATCCACCACGACCCTGAATACTTCCCGGACCCCTACAAGTTTGACCCTGAGAGATTCTCGGAGGAGAACAAGCACAAAATCAAGCCATTC
AGTTATATGCCTTTTGGACTTGGCCCCAGAAATTGTATTGGGTCAAGATTCGCTCTGTGCGAAGTCAAGGTCATGGCATACCAGCTGATCCAGCAGATG
GAGCTGTCTCCCTGCGAGAAGACTTCCATACCAGCTGTACTGGCTAAGGATACCTTCAACCTGAAGGTGGAAGGGGGACATTATATTAGGGTCAAGCTG
CGCCAGTAA

>FJ226477.1[moltingcarboxypeptidaseA]ID:ACL27225.1
ATGGGGTCCTTGATGAAGACGGTCGTGCTCCTGTGCGTCATATCCTCGGTGTTATGTACGCCGTTCATCAATAAACTACAGCCAGGACAAGAATGGCCT
ACTCGTAGCTCAGTTAAACAACCGTATCATCAATTCGATGAGATTGAAAATGGGACAATATCAGACACAGTTGCTACTGAAGTCGCAGCTCAGGAAGAA
AAGGCGCCTGAAAACAATAAACCTAAGGTAAAAGAAGATGACGTAGAGAGAATAGACTACAGCGGAGCCCAAGTTTGGAAAGTATCAACAAAGAAATCA
AACGCACACCGTGTTATTAATCAACTCGCGTCCGAGAAACTCATAGCATCATGGGGAGGCAACCACACATCAATAGACATTCTTGTGAAACCGAAAGCC
GTAGAAAACGTAACTGCAACCCTAAAAAATAAGGATATTGCGTATTACATCATGATTGAAGACCTGCAACAAAGAATCGACGAGGAAAATCCACCCTTA
GATGAAAATGAACTGGAGTTGCAAGACAGACGAGGTCATCGCATGACATGGAAGCAGTATCATAGACTGGAAGATATTCACGGATTTTTGGACTACTTG
GCAAAAAACGTATCCTTCAATCGTTAGCGTCAATAGTATAGGAAAATCTCACGAAGGCCGCGACCTCAAGGTATTACGCATCTCAGACGGCAAAAAGACT
AACAAAGCAGTGTTTATTGACGGCGGCATCCATGCGCGTGAGTGGATCAGTCCTGCTGTCGTTACATACTTCATTAACCAATTTGCGGAAAACTTTGAT
GTCGAATCTGACGACATTAGGAACATCGACTGGTACTTCTTGCCAGTAGTAAATCCTGACGGTTACGAATATACGCACAAGACGGACCGTCTCTGGCGT
AAGAACAGGAGAGGATTTGGTGGCTGTGCCGGCGCGGATCTCAACAGAAACTTTGGATATCAATGGGGAGGCAAAGGAGCTTCAAGATATCCATGCAGT
GAAATCTACCGCGGCAGCGGGCCTTTCTCTGAACCAGAATCTAAAGCAATTGCTCAATTCTTTAAAAGTACTGGAGCAGACTTCTCAGCGTACTTGACA
TACCATAGTTATGGACAGTACCTGCTCTACCCATGGGGCTATGACAATGCATTGCCACCAGACCATAAGAACCTTGAAACTGTGGGCAAGATGATGGCT
CAGGCCATACAAAAGACCGGAGGATCAGAATACAAAGTAGGATCATCAAGCGGGCTCCTCTACCCAGCTGCTGGCGGCTCCGATGACTGGGCCAAATCA
TTAAACATCAAGTACACTTACACAATTGAATTGAGTGACACAGGCCGTTATGGCTTTGTGTTGCCAGCATCATACATTGAGCCTGTTGCTAGGGAGAAC
TTAGCTGGCTTAAGGGTTCTAGCAAGCCAAGTGAACAAAGAGTAA

>EU770381.1[gene=BGRP1][beta-1,3-glucanrecognitionprotein1]ID:ACI32825.1
ATGGCGAGCGTGTGTGTTTTGTTTTTAATTTTTGTGTTAAGTTTTAATAATGGGTTGTGTTATGAAGTGCCAGCGGCTACTTTGGAAGCTATTTACCCT
AAAGGCTTAAGGGTTTCTATTCCTGACGATGGCTTCTCCCTATTCGCCTTCCACGGCAACCTGAACGAAGAAATGAACGGCCTCGAAGCTGGCCGCTGG
TCCAGAGACATTACCAAGCAGAAGAATGGACGCTACACCTTCAGGGACAGGAGTGTTGAGCTGAAGGTTGGAGACACCATCTATTTCTGGACGTATGTC
ATCAAAGATGGACTCGGATATCGACAGGATAATGGGGAGTGGACTGTCACAGGATACGTCGACGAAGCAGGAAACTCTGTCAACCCAACCGATGGTTCC

CCTCTCTCTTCATCTTCTCCTGTCGCCTCCCAACCTTTATCAGGCTCCCAACCATCATCAGGCTCCAGCCCATCCCTTGGAACTGCTCCTGCAGCAGCT
CCAGCCTCATTCAGTGCAGAGTACCCATGCGAGCTGTCAATTTCCAAGGTCAATGTACCAGGATTTGTGTGCAAGGGTCAGCTGCTTTTCGAAGATAAT
TTCAACTCCGGAATAGAAAAGGGGAAGATTTGGACTCCTGAGATCAAGTTCCCTGGAGAGCCGGACTTCCCATTCAACGTATACCTCAATGACCGCAAT
CTTCATGTAAGAGATGGACGGCTGAGTATCAAACCGATCACCCTGGAGTCTAAATACGGAGAGGACTTCGTGAGGCAGACACTGGATATCACGGCTAGG
TGCACTGGAACCATCGGGACTATGGAATGTTCGAGAGAGGCGTCTGGTCCTCAGATCTTGCCACCCATCATCACTTCTAAGATCACCACAAAGAACAAA
TTCGCTTTCAAGTACGGCAGGATTGAGATCAGCGCGAAATTGCCCCTTGGGGACTGGATTTATCCAGAAATCCAGCTGGAACCTCGTGACCACGTCTAC
GGCATCAGAAATTACGCGTCAGGACTCATGCGCATAGCTACTATCAAAGGAAACGCGGAATCTGCCAAGAAACTGTACGCGGGTCCTATCATGTGCGAT
ACTGAACCTTACAGATCTGCTTATCTAAAGGAGAAAGCGGGCTCTGACCTCTGGAGCAGAGATTTCCATAATTACTCATTGGAATGGAGACCAGACGGC
ATATCCCTATTCGTCGACGGCGAGAAATATGGCGAAGTAACTCCCCCAACAGAAGGGTTCTACAAGACAGCCACCGACAACCAAGTGGCTGCAGCTGCG
CAGTGGCTGAAAGGAACCACTATAGCCCCCTTTGATGATATGTTCTACATATCTCTCGGCCTAAACGTGGGAGGAGTTCACGAGTTCCCCGACACGGAC
AACAAGCCGTGGAAGAACCGTGCGACCAAGGCTATGCTGAACTTCTGGAACGCTCGGGAACAATGGTACTCCACCTGGTACGACGATACCAGCGCGCTG
CAGGTGGATTACGTCAGGGTATATGCGCTTTAG

>JF417988.1[seryl-tRNAsynthetase]ID:AEB26319.1
ATGGTGCTCGACTTAGACTTGTTCCGTGCCGACAAAGACGGGAACCCCGACAAAATCCGTGAAAACCAAAAAAAAAGGTTCAAAGATGTGGCCCTTGTT
GACACCGTGGTCGAACAGGACACTCTCTGGAGAAGATTACGCCACGAAGCTGACAATTACAACAAGCTGAAGAATGTGTGCAGCAAGGAGATTGGTCTT
AAGATGAAGAGCAAGGAGCCCGTTGGCCCAGAAGACCAGCCGGTACCAGCTGAAATAGCAGAGAAGCTAGTTGACCTGACTGGAGATGACTTGAAGCCT
TTAACTGTTAACCAAATTAAAAAGGTCAGAGTATTAATAGATGAAGCCATAGCAAAGAATGAACAGGGTCTCATAGCGGCAGAGAAGGCTCGTTCCGCA
GCTCTTCGTGAAGTAGGCAACCACCTCCACGAGTCTGTCCCTGTGGATGATGATGAGGACCACAACAAAGTGGAGAGGACTCACGGAGACTGCACGTTC
AGGACCAAGTACTCTCATGTGGATCTCATTTGTATGATTGATGGTATGGACGGCGACCGAGGCTCAGCCGTGGCCGGTGGGCGCGGGTACTACCTCAAG
GGCGCGGCGGTGTTCTTGGAGCAGGCGCTCATACAGCTGTCGCTCAGGATACTGCTGAAGAAGGGTTACACGCCACTGTATACTCCATTCTTCATGAGG
AAAGAGGTGATGCAAGAAGTAGCTCAACTAGCCCACTTCGACGAGGAACTGTATAAGGTGGTCGGCAAGGGATCTGAGAACAAGGGCGATGTGGCCATC
GAGGAGAAATATCTCATCGCGACGTCTGAGCAGCCGATAGCCGCCTACCACAGGGATGAGTGGCTTCCTGAGGCTTCATTACCCATAAGGTATGCAGGT
CTCTCAACGTGTTTCCGTCAAGAAGTAGGCTCCCACGGACGTGATACTCGCGGTATCTTCAGGGTTCACCAATTTGAGAAGGTAGAACAATTCGTCCTG
ACCTCCCCCCATGACAACGCGTCCTGGCAAATGATGGACGAGATGATCACCAACGCAGAGGAGTTCTACCAACTGTTGGACATCCCCTACCGCGTCGTC
AACATCGTGTCAGGGGCGCTCAATCATGCGGCCGCTAAGAAACTGGACTTGGAGGCCTGGTTCCCCGGCTCTGGGGCTTTCCGTGAACTGGTGTCGTGC
AGCAACTGCTTGGAGTACCAGGCTAGGAGGTTGCTTGTCAGATACGGTCAAACGAAGAAGATGAACGCAGCCACGGAGTACGTGCACATGCTGAACGCG
ACGATGTGCGCCACCACGCGCGTCATCTGCGCCGTGCTCGAGGTCAACCAGACTGAGGAGGGCATCAAGGTACCTGAAGCCCTGAAACAATGGATGCCT
GAGCAATACCAGGAACTGATTCCATTCGTGAAGCCAGCACCCATCGACGTGGAAGCTGCCGCCGCCGCCAAGAAAAAAGGAAAGAAATAA

>JF417989.1[voltage-dependentanion-selectivechannel]ID:AEB26320.1
ATGGCTCCCCCATACTATGCTGATCTTGGAAAGAAGGCCAATGATGTCTTCAGCAAAGGCTACCACTTTGGTGTATTTAAATTTGACCTGAAGACCAAG
AGTGAAACAGGTGTTGAATTCGCCAGTGGAATCACCTCCAACCAAGAAAGCGGAAAGGTTTTCGGTAGCCTTTCATCAAAATATGCAGTGAAGGACTAT
GGCCTTACGTTCACTGAGAAGTGGAACACAGACAACACACTTGCCACTGATATTACGATCCAGGACAAGATTGCTGCCGGCCTTAAAGTAACCCTTGAA
GGAACCTTTGCACCACAGACCGGAACCAAGACTGGTAAATTGAAGACCTCTTTTGCTAATGATACCGTTGCCGTAAACACCAACTTGGATTTGGATCTG
GCTGGACCCATTGTGGATGTGGCTGCTGTCCTCTCCTTCCAGGGCTGGCTTGCCGGTGTGCACGGCCAGTTTGACTCACAGAAGACCAAGTTCTCCAAG
AACAACTTCTCTCTTGGTTATGAGTCTAAGGACTTCAACCTACACACCAATGTTGACAACGGAAAGGACTTCGGTGGTTCTATCTACCAGAAGGTGTCA

GAAAAGATCGACTGCGGTATTAGCATGAAGTGGACCGCGGGCTCGGCCGACACACTGTTTGGCGTCGGAGCCAAGTTCGCGCTAGACGCCGACGCGTCT
CTGCATGCCAAGGTCAACAACAAATCTCTCATTGGTCTTGGATACCAACAGAAGTTGCGCCCAGGCGTGACTTTGACAATTTCTGCTGCTATCGATGGC
CAGAACTTCAACGCTGGTGGACACAAAGTGGGTGTCGCCCTGGAGCTCGAGCCCTAA

>FJ378903.1[proteasomesubunitbeta1]ID:ACJ13433.1
ATGCTGAGTGTTGGCGGAAATTTTCCTGAATATGCTGTTCCTGGCGCTAAACAAGTTCGCTTCGAGCCGTACGCTGACAACGGAGGAAGCATCGTTGCT
ATTGCCGGTGATGATTTTGCAGTAATTGGTGCTGATACACGTCTTAGTACGGGATTCTCGATCTACACAAGGGAACAAAAGAAATTGTTTAAGCTGTCC
GACAAAACTGTATTGGGCGCGACCGGCTGCTGGTGCGACACGCTGACTCTCACCAGACTGCTCTCTGCCAGAATGCAGATGTATGAACACGAGCATAAC
AAGTCTATGTCGACTCCTGCCGTGGCTCAGATGCTTTCCACGATGCTCTACTATAAGCGGTTCTTCCCATATTATGTGTCTAATGTGTTGGCTGGTCTG
GATGCTGACGGAAAAGGCTGCGTGTACAGCTACGACCCTATCGGCCATTGTGAACGCTCCAACTACCGCGCTGGTGGCTCAGCGGGTGCTCAGCTGCAG
CCCCTGCTTGACAACCAGATAGGGCTTAAGAACATGCAGAACGTTACAGAAGCCCCCATACCTAAAGAGAAGGCTTTAGCCTTACTGAAGGATGTGTTC
ATCAGCGCTGCTGAGCGTGACATTTATACTGGAGACTGCATTTACATATTGATTATTACTGCAAGTGGCATCCAAGAGGAGAAATTTGAATTGCGTAAA
GATTAA

>JF417987.1[receptorforactivatedproteinkinaseC]ID:AEB26318.1
ATGACTGAAACTCTAAAGCTTAGAGGAACCCTCTGTGGCCACAATGGCTGGGTTACCCAAATTGCGACCAACCCTAAATACCCTGACATGATTTTGTCT
TCTTCCCGAGACAAAACCCTCATCGTATGGAAGCTGACCAGAGACGAGACTAACTACGGTGTCCCGCAGAAGCGTCTGTACGGTCACTCTCACTTCATC
TCGGACGTTGTGCTCTCCAGTGACGGAAACTACGCTCTGTCTGGCTCCTGGGACAAGACCCTGCGTCTGTGGGATCTTGCTGCCGGCAAGACCACCAGG
CGTTTCGAAGACCATACTAAGGATGTCCTCTCCGTGGCATTCTCAGTTGACAACCGTCAGATCGTGTCTGGCTCCCGAGACAAGACCATCAAGCTGTGG
AACACACTGGCTGAGTGCAAGTACACCATCCAGGATGATGGCCACAGTGACTGGGTGTCCTGCGTCCGCTTCTCCCCCAACCATGCCAACCCCATCATT
GTGTCCGCTGGTTGGGACCGCACCGTTAAGGTCTGGCACCTTACCAACTGCAAGCTGAAGATCAACCACCTTGGTCACTCTGGCTACCTGAACACAGTC
ACCGTCTCCCCTGACGGTTCCCTCTGCGCCTCCGGTGGCAAGGACATGAAGGCCATGCTCTGGGACTTGAACGATGGCAAGCATCTGCACACCCTGGAC
CACAATGACATCATCACATCATTGTGCTTCTCGCCCAACAGATACTGGCTGTGTGCTGCCTTCGGACCTTCCATCAAGATCTGGGATCTGGAAAGCAAG
GAGATGGTTGAAGAGCTCAGGCCTGAGATCATCAACCAGACCCAGACCTCCAAGTCAGACCCACCCCAGTGCTTGTCTCTGGCGTGGTCCACAGACGGT
CAGACCCTCTTCGCTGGCTACTCCGACAACATCATCAGAGTCTGGCAGGTGTCAGTCTCAGCGCGATAA

>FJ602793.2[juvenilehormoneepoxidehydrolase]ID:ACM78602.2
ATGGTGCGACTCCTGTTCATAGCGCCCATTTTGGCGGTGATCCTAGTTCCCATATACTTCGTATTCCTCCAGGGACCCCCTCCCTTACCCGACATTGAC
CTCAACGAGTGGTGGGGACCGGAGAGCTTGAAAGCCAAACAAGACACCAGCATCAGACCCTTCAAAGTTGCTTTCGATGACGCTGCCATCAGAGACTTG
AAAGACCGTCTCAAAAGATCGCGGTCTTTCACCCCACCACTAGAGGGCGTGGCCTTCGAGTACGGTTTCAACAGTGGCCAGTTGGACTCCTGGCTGAAG
TATTGGGCCAATGAGCACCAGTTCAAGGAACGGGAGAAGTTCTTCAACCAGTTCCCGCAGTTCAAGACTAACATTCAAGGGCTTGATATACATTTCATT
AGGGTTACACCTAAGGTGCCAGCAGGAGTGGAAGTGGTTCCTCTCCTTCTTCTCCACGGATGGCCAGGTTCCGTCAGGGAGTTCTACGAAGCCATCCCC
CTCATCACTGCGGTCAGCAAGGATAGAGACTTCGCCATCGAAGTCATCGTCCCAAGCCTGCCTGGCTACGGATTCTCAGATGGAGCTGTCCGTCCAGGA
CTCAGTGCCCCCCACATTGCTGTCGTGATGAGGAACCTGATGCATCGCCTCGGCTTCAAGCAGTTCTACGTGCAAGGCGGAGACTGGGGCAGTCTGATC
GGCACCACCTTGGCTACCTTCTTCCCTAAGGAAGTCCTGGGCTACCATACCAACATGGGTGCTGTTCTGTCTACTAAGGCCACATTGATAGAGATCATC
GGATCTTTCTACCCTTCACTAATTGTGGAACCTCATCTAGCCGACCGCATGTATCCAATGGGTCAGAGATACGCCACTCTGGTTGAAGAAATGGGCTAC
ATGCATATTCAAGCCTCCAAGCCTGACACAGTTGGTGTTGCACTCACGGATTCCCCAGCTGGTCTCCTGGCATACATTTTGGAGAAGTTCTCCACTTGG

ACGAGGAACGAGCACCGCTTGAAGGCCGATGGAGCCTTAACATTCCGCTTCACAAAGGATCAGCTTATAGATAACTTGATGATGTACTGGGCTCCAAGT
TCCATCACCACCTCGATGAGGCTGTACGCTGAGAGTTTTAACTCTAAAATTTTTGGGCTGAAGCTTGACGAGATTCCAACACCAGTACCAGTTTGGGTG
ATCCAAGCGAAGTACGAGTTGGCATACCAGCCTCCTTGCATCCTCAAGCTCAAGTTCCCCAACCTTCAAGGAGTAACAGTCCTGGAGGATGGAGGGCAT
TTCCTGGCGTTTGAACTGCCCAAGGAATTTTCCGAGGATGTCCTGAAGGCCATGGCGGTCTTCAGAAAGCTGTCCAAAAATAATGTGAAGACTGATTTG
TAA

>EF600050.1[gene=GH32FruA-1][fructosidase]ID:ABU98615.1
ATGTTGTCAATCGCAACAAAGTTTATACTAACAGCTCTGTTCTTGAACAGTTGCTGGGCCATTGATGTCAATCCTCGCTACGTACCGCATTACCACGTC
TATCCGCCTTCTGGATGGATGAACGATCCCAACGGCTTCTGTATCTTCGAAGATGAATACCACTTGTTCTACCAATACAACCCTTACTCCAGCCAAGAA
CCTGGAGTAGCTCATTGGGGACATGTGAAGAGTCCTGATCTAATCAATTGGGAACATTTACCTACAGCAATGACCCCAGATCAACCGTACGACATCAAT
GGAGTATTCTCAGGAAGTGCTATTGTCGAAAATGGAACAATGTATCTCCTCTATACTGGTAACGTTAATAACCCAATCAACAAGCAAGTGCAAGCTTTA
GCTGCAAGCCAGGATGGAATATCAGTGGTTAAATATCCTGGCAATCCTGTCATCGAAGGGGCAGATTTTCAGCCAAACATAAGAGACCCGAAAGTCTGG
AAGCACGGAGATCTTTTCTACATGGTGCTGGGCAACTCTTTCGATGATAACACCCGCGGTCGAGTACTGCTATATTCATCGCCGGATTTAATATCTTGG
ACACTTGAGTCAGTTTTAGATGAGTCTGACGGCTCTGTGGGTCATGTTTGGGAGTGCCCTGATTTCTTCGAGTTGGACGGAAAATACGTACTGCTATTT
TCTCCACAAGGTATGCAAGCCATTGGTGATAAATACAAGAACTTTTTCCAGACTGGATACGTAGTGGGCAACTTTGATTATGAAAACGAAATTGTTTAC
ACCTATAACCGAATTTTAGAGAATTTAGACCACGGACATGATTTTACGCTACTCAAACTATTTTTGGACGGCTCAGGACGTAGGGTTTTTGTGGCTTGG
TTTAGCATGTGGGAGTCGGTACACCCTGAGCGCAACGACGGTTGGAGTGGTCAAATTACTATTCCGAGAGAGCTTGAATTGACAGAAGGTTTAAGGCTC
TTACAAAAACCTGTTAAGGAGCTTGATGCTGCACGAGGTCCAAAATTACGCTCTGGAAAGGCTAAAGCCGGTTACACCCTGGGATTAGAAGCCGCGGCT
GCTGACATTCGAGTGACGGCACCAAGACTACAAGATTTTGAGTTACTTTTGGAATCTGATGATTCTACTCTTTCAATCAAATATGACTACAGGAGAGGT
ACAGTCACTCTGGATCGGGGAGGTGACGATGGTGTTCGCAGAACAAAGTGGAGGCCTGAAGGTAATCTTATCTGGCAGGTCTTGGTAGACTCGAGCTCC
GTTGAACTGTTCTGTGGAGAAGGTGAAGTCACTTTCTCCAGTAGACTCTTCCCTAACGGTGCATTGAAAATCAGGTTAGGTGATAGTTCTAATGCTGAT
GATCTAACAGTTTACAAAATGCTTCGTACTATTGCAGCACCCGGTGACAATTGA

(Back to top)

Table S2B. Codon usage data for *Heliothis virescens*, *Helicoverpa armigera*, and *Bombyx mori*.

Annotated *H. virescens* transcriptome was used to select 50 full length open reading frames that also had full length homologous sequences for *H. armigera* and *B. mori* in public databases. All selected sequences had E-values below -135. Codon usage was calculated for each ORF and the sum of codon numbers within each insect species was used to calculate average codon usage per 1000 codons, the proportional codon usage of degenerate codons, and the relative adaptiveness of each degenerate codon. Relative adaptiveness was calculated by setting the codon with highest usage fraction within each degenerate codon set to 100% and proportionately scaling the fractions of remaining codons. Non-degenerate codons AUG (Met) and UGG (Trp) were not used in these calculations.

			Hv							Ha				Bm		
AmAcid	Codon	Number	/1000	Fraction		Rel. Adap.		Number	/1000	Fraction	Rel. Adap.		Number	/1000	Fraction	Rel. Adap.
Ala	GCG	399	15.9	0.211		63.7		371	14.7	0.198	57.7		316	12.5	0.183	57.5
Ala	GCA	303	11.1	0.160		48.4		285	10.2	0.152	44.3		327	12.5	0.189	59.5
Ala	GCT	626	23.4	0.331		100.0		643	23.8	0.343	100.0		550	21.1	0.319	100.0
Ala	GCC	561	22.3	0.297		89.6		578	23.0	0.308	89.9		533	21.0	0.309	96.9
Cys	TGT	148	5.8	0.345		52.7		138	5.5	0.337	50.7		171	6.9	0.417	71.5

Cys	TGC	281	11.4	0.655		100.0		272	11.1	0.663	100.0		239	9.8	0.583	100.0
Asp	GAT	584	22.0	0.374		59.7		596	22.2	0.378	60.8		739	28.4	0.470	88.5
Asp	GAC	978	38.0	0.626		100.0		981	38.9	0.622	100.0		835	34.0	0.530	100.0
Glu	GAG	829	31.4	0.491		96.4		844	32.2	0.498	99.3		683	26.1	0.404	67.8
Glu	GAA	860	31.0	0.509		100.0		850	30.5	0.502	100.0		1008	37.1	0.596	100.0
Phe	TTT	308	11.9	0.259		35.0		307	11.7	0.254	34.1		423	15.7	0.342	51.9
Phe	TTC	880	33.5	0.741		100.0		900	34.4	0.746	100.0		815	32.3	0.658	100.0
Gly	GGG	161	6.0	0.096		27.9		162	5.8	0.095	27.3		186	7.2	0.115	35.3
Gly	GGA	468	18.2	0.278		81.1		479	18.4	0.279	80.8		527	21.0	0.325	100.0
Gly	GGT	479	18.9	0.284		83.0		480	18.9	0.280	80.9		465	19.4	0.287	88.2
Gly	GGC	577	23.8	0.342		100.0		593	25.0	0.346	100.0		443	18.4	0.273	84.1
His	CAT	210	8.0	0.353		54.5		209	8.0	0.345	52.6		253	9.7	0.415	70.9
His	CAC	385	14.6	0.647		100.0		397	15.3	0.655	100.0		357	14.2	0.585	100.0
Ile	ATA	277	10.2	0.201		39.1		259	9.5	0.186	35.3		423	15.9	0.282	71.3
Ile	ATT	390	14.0	0.283		55.0		396	14.6	0.285	54.0		485	18.5	0.323	81.8
Ile	ATC	709	27.7	0.515		100.0		734	29.2	0.528	100.0		593	24.0	0.395	100.0
Lys	AAG	959	38.1	0.614		100.0		991	39.9	0.623	100.0		774	32.9	0.490	96.3
Lys	AAA	604	23.1	0.386		63.0		600	22.9	0.377	60.5		804	31.0	0.510	100.0

Leu	TTG	409	14.9	0.177		59.5		410	15.0	0.179	59.5		503	19.0	0.220	100.0
Leu	TTA	250	9.3	0.108		36.4		251	9.1	0.110	36.4		322	12.2	0.141	64.0
Leu	CTG	687	26.7	0.298		100.0		689	27.0	0.301	100.0		476	19.0	0.208	94.6
Leu	CTA	210	7.6	0.091		30.6		187	6.8	0.082	27.1		247	9.0	0.108	49.1
Leu	CTT	304	11.1	0.132		44.3		308	11.7	0.135	44.7		322	12.2	0.141	64.0
Leu	CTC	447	17.4	0.194		65.1		444	17.4	0.194	64.4		419	16.8	0.183	83.3
Met	ATG	661	24.9	1.000		100.0		653	24.7	1.000	100.0		598	23.1	1.000	100.0
Asn	AAT	429	15.5	0.326		48.4		373	13.2	0.294	41.7		536	19.7	0.412	70.0
Asn	AAC	887	33.4	0.674		100.0		895	33.8	0.706	100.0		766	29.1	0.588	100.0
Pro	CCG	230	9.0	0.175		55.4		248	9.4	0.181	59.4		326	13.0	0.257	100.0
Pro	CCA	313	11.5	0.239		75.4		294	10.9	0.211	69.3		320	11.7	0.253	98.2
Pro	CCT	415	15.4	0.316		100.0		412	15.1	0.304	100.0		313	12.0	0.247	96.0
Pro	CCC	354	13.8	0.270		85.3		368	14.4	0.304	100.0		308	11.9	0.243	94.5
Gln	CAG	551	21.2	0.602		100.0		545	21.2	0.602	100.0		399	16.1	0.480	92.1
Gln	CAA	365	13.3	0.398		66.2		361	12.6	0.398	66.2		433	16.4	0.520	100.0
Arg	AGG	259	9.5	0.208		87.5		244	8.9	0.198	84.1		206	7.5	0.169	58.0
Arg	AGA	293	10.8	0.235		99.0		290	10.6	0.235	100.0		355	13.3	0.291	100.0
Arg	CGG	81	3.2	0.065		27.4		92	3.5	0.075	31.7		100	4.1	0.082	28.2
Arg	CGA	97	3.7	0.078		32.8		84	3.2	0.068	29.0		136	5.2	0.111	38.3

Arg	CGT	220	8.4	0.177		74.3		241	9.3	0.195	83.1		216	8.6	0.177	60.8
Arg	CGC	296	11.9	0.238		100.0		282	11.4	0.229	97.2		208	8.6	0.170	58.6
Ser	AGT	225	8.3	0.134		65.8		218	8.1	0.131	64.9		228	8.4	0.142	69.7
Ser	AGC	318	12.1	0.189		93.0		301	11.5	0.182	89.6		280	11.1	0.174	85.6
Ser	TCG	227	8.4	0.135		66.4		205	7.9	0.124	61.0		249	9.7	0.155	76.1
Ser	TCA	245	9.4	0.145		71.6		263	9.8	0.159	78.3		241	9.5	0.150	73.7
Ser	TCT	328	12.2	0.195		95.9		335	12.5	0.202	99.7		327	12.7	0.203	100.0
Ser	TCC	342	13.5	0.203		100.0		336	13.9	0.203	100.0		285	11.8	0.177	87.2
Thr	ACG	231	8.8	0.164		50.2		217	8.3	0.155	48.4		290	11.2	0.214	77.5
Thr	ACA	307	11.7	0.218		66.7		305	11.0	0.218	68.1		334	12.5	0.247	89.3
Thr	ACT	408	15.1	0.290		88.7		429	15.5	0.307	95.8		374	14.8	0.277	100.0
Thr	ACC	460	17.7	0.327		100.0		448	18.0	0.320	100.0		354	14.2	0.262	94.7
Val	GTG	613	23.6	0.328		100.0		641	25.2	0.346	100.0		518	20.4	0.300	100.0
Val	GTA	319	12.0	0.171		52.0		311	11.4	0.168	48.5		304	12.2	0.176	58.7
Val	GTT	395	14.9	0.212		64.4		380	13.6	0.205	59.3		429	16.5	0.249	82.8
Val	GTC	540	20.6	0.289		88.1		523	20.1	0.282	81.6		474	18.6	0.275	91.5
Trp	TGG	394	15.3	1.000		100.0		391	15.4	1.000	100.0		391	15.7	1.000	100.0
Tyr	TAT	303	11.4	0.281		39.0		300	11.2	0.282	39.2		410	15.8	0.393	64.8
Tyr	TAC	776	30.1	0.719		100.0		765	29.5	0.718	100.0		633	25.2	0.607	100.0
		26165						26104					25579			
End	TGA	9	0.3	0.180		29.0		6	0.2	0.120	18.2		11	0.5	0.220	36.7

End	TAG	10	0.5	0.200		32.3		11	0.5	0.220	33.3		9	0.4	0.180	30.0
End	TAA	31	1.3	0.620		100.0		33	1.4	0.660	100.0		30	1.3	0.600	100.0
