# Supplementary material for: Correction: Generation of a Transcriptome in a Model Lepidopteran Pest, Heliothis virescens, Using Multiple Sequencing Strategies for Profiling Midgut Gene Expression
Source: PLoS One. 2015 Jul 24;10(7):e0133948. doi: 10.1371/journal.pone.0133948 (PMC4514890; doi:10.1371/journal.pone.0133948)
Supplement: S1 File — (PDF) [file pone.0133948.s001.pdf]

PLOS ONE

RESEARCH ARTICLE

Generation of a Transcriptome in a Model Lepidopteran Pest, *Heliothis virescens*, Using Multiple Sequencing Strategies for Profiling Midgut Gene Expression

Omaththage P. Perera[1☯*], Kent S. Shelby[2☯], Holly J. R. Popham[2¤], Fred Gould[3], Michael J. Adang[4], Juan Luis Jurat-Fuentes[5]

1 Southern Insect Management Research Unit, USDA, Agricultural Research Service, Stoneville, MS, 38776, United States of America, **2** Biological Control of Insects Research Laboratory, USDA, Agricultural Research Service, Columbia, Missouri, 65203, United States of America, **3** Dept. Entomology, North Carolina State University, Raleigh, NC, 27607, United States of America, **4** Dept. Entomology, University of Georgia, Athens, GA, 30602, United States of America, **5** Dept. Entomology and Plant Pathology, University of Tennessee, Knoxville, TN, 37996, United States of America

☯ These authors contributed equally to this work.
¤ Current address: Agbitech, 1601 S. Providence Rd., Columbia, MO, 65211, United States of America
* OP.Perera@ars.usda.gov

OPEN ACCESS

Citation: Perera OP, Shelby KS, Popham HJR, Gould F, Adang MJ, Jurat-Fuentes JL (2015) Generation of a Transcriptome in a Model Lepidopteran Pest, *Heliothis virescens*, Using Multiple Sequencing Strategies for Profiling Midgut Gene Expression. PLoS ONE 10(6): e0128563. doi:10.1371/journal.pone.0128563

Academic Editor: Peng Xu, Chinese Academy of Fishery Sciences, CHINA

Received: September 9, 2014

Accepted: April 29, 2015

Published: June 5, 2015

Copyright: This is an open access article, free of all copyright, and may be freely reproduced, distributed, transmitted, modified, built upon, or otherwise used by anyone for any lawful purpose. The work is made available under the Creative Commons CC0 public domain dedication.

Data Availability Statement: All relevant data are within the paper and its Supporting Information files.

Funding: This work was partially supported by the U. S. Department of Agriculture under National Research Initiative Award No. 2004-35607-19935 to M.J.A. and J.L.J.-F., and Biotechnology Risk Assessment Grant Award No. 2008-03046 to J.L.J.-F. and O.P.P. The funders had no role in study design, data collection and analysis, decision to publish, or preparation of the manuscript.

Abstract

Heliothine pests such as the tobacco budworm, *Heliothis virescens* (F.), pose a significant threat to production of a variety of crops and ornamental plants and are models for developmental and physiological studies. The efforts to develop new control measures for *H. virescens*, as well as its use as a relevant biological model, are hampered by a lack of molecular resources. The present work demonstrates the utility of next-generation sequencing technologies for rapid molecular resource generation from this species for which lacks a sequenced genome. In order to amass a *de novo* transcriptome for this moth, transcript sequences generated from Illumina, Roche 454, and Sanger sequencing platforms were merged into a single *de novo* transcriptome assembly. This pooling strategy allowed a thorough sampling of transcripts produced under diverse environmental conditions, developmental stages, tissues, and infections with entomopathogens used for biological control, to provide the most complete transcriptome to date for this species. Over 138 million reads from the three platforms were assembled into the final set of 63,648 contigs. Of these, 29,978 had significant BLAST scores indicating orthologous relationships to transcripts of other insect species, with the top-hit species being the monarch butterfly (*Danaus plexippus*) and silkworm (*Bombyx mori*). Among identified *H. virescens* orthologs were immune effectors, signal transduction pathways, olfactory receptors, hormone biosynthetic pathways, peptide hormones and their receptors, digestive enzymes, and insecticide resistance enzymes. As an example, we demonstrate the utility of this transcriptomic resource to study gene expression profiling of larval midguts and detect transcripts of putative *Bacillus thuringiensis* (Bt) Cry toxin receptors. The substantial molecular resources described in this study will facilitate development of *H. virescens* as a relevant biological model for functional

Competing Interests: The authors declare that no competing interests exist.

genomics and for new biological experimentation needed to develop efficient control efforts for this and related Noctuid pest moths.

Introduction

Heliothine moths are major polyphagous pests of commodity crops such as maize, cotton, soybeans and vegetables throughout the world [1]. In the Western Hemisphere, larvae of the tobacco budworm, *Heliothis virescens* (F.), are major pests of agricultural production through their feeding on cotton, soybean, tomato and other crops [2,3]. Populations of *H*. *virescens* are notorious for rapidly evolving resistance to insecticides [4], and are now primarily controlled in cotton by cultivars genetically modified to express insecticidal proteins from the bacterium *Bacillus thuringiensis* (Bt).

Control of *H*. *virescens* and other closely related heliothines would be greatly advanced by better understanding mechanisms underlying susceptibility and resistance to insecticides and biological control agents. For instance, availability of molecular resources would aid in the identification of gene silencing targets for field applications of novel insecticidal technologies [5–7]. Moreover, *H*. *virescens* is one of the most important lepidopteran models for characterization of development [8,9], pathogenesis [10,11], resistance to insecticides and pathogens [12–14], and nutritional physiology [15,16]. However, as with other economically important agricultural pests, the development of molecular resources for *H*. *virescens* has not received the efforts spent on model organisms such as *Drosophila*, which in turn is also hindering the development of *H*. *virescens* as a model for functional studies [17–19].

Massively parallel "next generation" sequencing technologies have revolutionized the acquisition and analysis of transcriptomes and genomes from non-target [20–23], beneficial [24–28] and pest insects [29–42]. Platforms such as the Illumina HiSeq2000 can produce in excess of one terabase of sequence data per run, which allows a stunning acceleration in acquisition of molecular resources from any invasive pest or beneficial insect. These technologies have been applied to selected lepidopteran species as model systems for the study of pathogenesis [41,43]. Moreover, available transcriptomic resources have guided the development of molecular markers associated with functional transcripts pertaining to life history and physiology. For instance, molecular markers from next generation sequencing projects, such as single nucleotide polymorphisms (SNPs), microsatellite loci, barcoding, etc. can provide extremely useful experimental tools for the study of taxonomy, development, adaptation, and ecological genomics of insect pests [17–19,44,45]. These markers have also advanced investigations of ecological and nutritional immunology, and have identified targets for gene silencing with RNA interference (RNAi) [6,7,39,46–49]. Methods associated with next generation sequencing, such as transcriptome profiling by RNA-Seq, have been applied to resolve agricultural issues [50]. However, despite current technological advancements, some of the developed non-model insect transcriptomic resources are limited by the sequencing technology, or by the specific life stage or tissue source, limiting the amount of useful information and the potential detection of relevant transcripts.

To help resolve the current critical lack of molecular resources for *H*. *virescens*, we present here the most comprehensive budworm transcriptomic resource developed to date. This transcriptome was obtained by combining transcript data from diverse sequencing platforms for all life stages, tissues and numerous treatments of *H*. *virescens* into a single *de novo* transcriptome. This approach increases the probability of obtaining a comprehensive collection of common

and rare environmental transcripts. Furthermore, we demonstrate the utility of this resource for gene expression profiling of larval midguts, and report contigs encoding putative receptors for Bt Cry toxins.

Results and Discussion

Tissues and treatments represented in transcriptome

Our strategy was to pool transcript sequence data generated for *H*. *virescens* through diverse platforms to generate a comprehensive transcriptome, a resource which has been released to the research community (ncbi.nlm.nih.gov/genomeprj/49697). Initial efforts focused on transcripts obtained using both conventional cDNA library techniques and massively parallel Illumina sequencing from *H*. *virescens* hemocytes after baculoviral, bacterial and fungal infection. Hemocytes were selected as a good tissue to facilitate identification of putative resistance genes to pathogens because they are the primary immune responders, and can be isolated from insects in high number and relatively uncontaminated by other tissues or microbial flora. To activate the immune response, while minimizing the harvest of pathogen sequences, purified pathogen cell wall components devoid of nucleic acids were injected into the hemocoel, as previously described [51]. In the case of baculoviral infection, collected viral (HzSNPV) sequences were identified using the fully sequenced viral genome (NCBI accession # NC_003349) and these sequences were not included in this analysis.

A second group of transcripts was obtained from the midgut of *H*. *virescens* larvae through Illumina HiSeq 2000. This tissue was expected to yield transcripts related to digestive and defensive functions, including genes responsible for detoxification of toxicants ingested while feeding. Additional transcript collections were from Sanger sequencing efforts from all *H*. *virescens* life stages, and from Roche 454 pyrosequencing of female moth pheromone glands. However, time points were not optimized to sample all transcripts expressed during embryonic or larval development. Environmental treatments such as heat, cold, diapause, insecticide intoxication, and heavy metal intoxication were not included, and thus transcripts associated with these responses may not have been sufficiently sampled by our effort. Also ovaries, testes, fat bodies, Malpighian tubules and other major organs were not explicitly included in this sampling effort, although some of these tissues were represented among the Sanger reads. Consequently, while representing the most comprehensive collection of *H*. *virescens* transcripts currently available, the present transcriptome cannot be considered exhaustive or complete.

Assembly and analysis of contigs

An iterative *de novo* budworm transcriptome assembly was constructed by combining Illumina, Roche 454, and Sanger reads. Five cycles of addition and reassembly were performed using SeqMan NGen v2.1 in which over 138 million high quality input sequences (about 65% of >212 million reads) contributed to an initial budworm transcriptome of 69,643 contigs ≥80 bp with an average length of 383 bases (average coverage of 21), and 2,976 contigs greater than 2kbp in length. Contigs and singletons of <100 bases in length were excluded from subsequent analyses. Clustering of remaining contigs and singletons of > 100 bases in length displaying 95% or higher sequence identity [52] resulted in 63,648 sequence contigs with cumulative and average contig lengths of 42,683,498 and 670 nucleotides, respectively. The number of contigs of ≥2 kbp in length increased to 3,151 after clustering. The N_{50} and N_{90} of the final transcriptome were 1031 and 316 bp, respectively (Table 1).

In order to identify orthologous transcripts in the final *H*. *virescens* assembly, annotation of the clustered sequences was performed using BLAST2GO (blast2go.org) [53]. This procedure resulted in 29,978 candidate protein coding genes with at least one significant BLAST hit

Table 1. *De novo* Assembly and Annotation Summary of *Heliothis virescens* Transcriptome.

Parameter	Assembly Data
**Input Sequences (Cumulative Assembly)**	**Input Count**
Illumina Reads (36 nt)[1]	16,391,117
Illumina reads (42 nt)[1]	196,186,649
Roche-454 Reads[1]	371,302
Sanger Reads[2]	37,960
All Sequence Reads	212,987,028
Total Base Pairs	7,877,720,878 (7,877 Mbp)
**Assembly Totals[3,4]**	**Output Count**
Contigs ≥ 100 bp	63,648
Contigs ≥ 2,000 bp	3,151
Contig N_{90}	316 bp
Contig N_{50}	1031 bp
Contigs ≥500 bp	31,280
Contigs ≥200 bp	47,562
Cumulative Length of Contigs	42,683,498bp
Avg Coverage	21
Assembled Sequences	138,441,568
Unassembled Sequences	74,545,459
Avg Length of Contigs	670
**Annotated Contigs[5]**	**Count**
Unitigs	29,978
> 1000 bp	9679
800–999 bp	3008
600–799 bp	4274
400–699 bp	4341
200–399 bp	4422
100–199 bp	4254
**N_{50}**	1374 bp
**Cumulative Length of Annotated Contigs**	27,893,942 (Avg 930 bp)

[1]This report.
[2]NCBI dbEST.
[3]SeqMan NGen Assembly Report. SeqMan NGen 2.1.0 build 13 Assembly Parameters [Match Size: 15, Match Spacing: 10, Min Match %: 85, Match Score: 10, Mismatch Penalty: 20, Gap Penalty: 30, Max Gap: 15, Expected Coverage 22].
[4] Based on contigs ≥100 bp after clustering contigs with ≥90% sequence identity
[5]Significant BLAST score (eValue≤0.00001 using BLAST2GO).

doi:10.1371/journal.pone.0128563.t001

(Table 1). The majority of transcripts were most similar to those of other insects with well annotated genomes, while insects with reduced genomic representation in databases were less represented (Fig 1, and S1 Table). Thus, most contigs had significant matches to orthologs in *Danaus plexippus* (49.4% of annotated contigs) and *Bombyx mori* (30.4% of annotated contigs). Database entries for *Helicoverpa spp*. matched 4.5% of the contigs while only 2.3% (696) contigs matched *Heliothis spp*, reflective of the lack of relevant genomic resources for heliothines compared to other Lepidoptera models. Contigs that had no significant BLAST score (E-value cutoff 10E-3) were excluded from subsequent analyses.

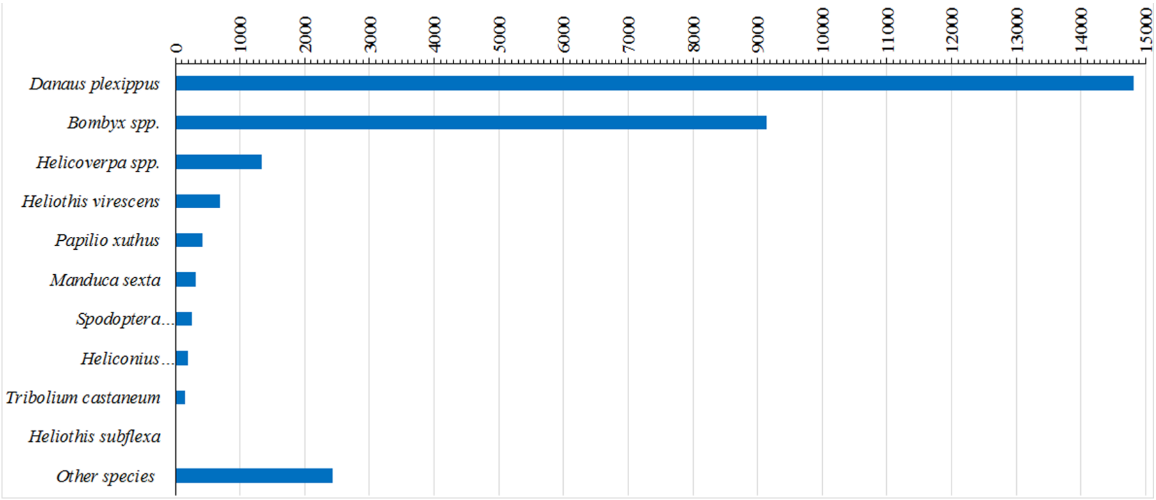

Fig 1. Top hit species distribution of assembled *Heliothis virescens* unigene set. Sequence length statistics of input unigenes (>100 nt) with significant BLASTx score (e≤0.00001).

doi:10.1371/journal.pone.0128563.g001

Within the contigs with significant BLAST matches, 13,298 (44.4% of total) were fully annotated and 2,239 had only mapped GO codes. Examination of resulting GO and KEGG maps demonstrated that transcripts corresponding to all major metabolic pathways expected for an insect were present (Fig 2A, 2B and 2C). Despite extensive pre-filtering of raw sequence output, detailed examination of the annotated contigs revealed several sequences of possible bacterial and viral origin. Apart from sequences probably corresponding to midgut microflora, which have received no detailed study in this species, known testicular endosymbionts of *H. virescens* (L22481.1) [54] were also detected within the assembly.

Codon usage comparison

Open reading frames of the *H. virescens*, *H. armigera*, and *B. mori* genes used in calculating codon preference and the comparative codon usage data are given in the S2A Table and S2B Table. Graphical representation of relative adaptiveness (RA) of codons is given in Fig 3. The total number of amino acid coding sequences in the 50 ORFs of *H. virescens*, *H. armigera* and *B. mori* were 26,165, 26,104, and 25,579, respectively. Relative adaptiveness was similar for most codons in *H. virescens* and *H. armigera*, but differed in *B. mori*. For example, RA for AGR and CGY codons (Arg) in *H. virescens* and *H. armigera* ranged from 74–100%, while AGA (100%) and CGU (61%) codons were predominantly used in *B. mori*. In *H. virescens* and *H. armigera* Leu was predominantly coded by CUG (100%) followed by CUC (65%) and UUG (60%). In *B. mori*, UUG (100%), CUG (95%), and CUC (83%) codons were used predominantly for Leu, followed by CUU and UUA (each at 64%). Both codons for Lys (AAA and AAG) were equally represented in *B. mori* ORFs, but AAG was predominantly used in *H. virescens* and *H. armigera*. Ochre codon (UAA) was predominantly used in the 50 ORFs from all three species to terminate translation. The other two stop codons, UGA (18 to 37%) and UAG (30 to 33%) were much less frequently used in all three species. In summary, comparison of *H. virescens* protein coding sequences with homologous (*i.e.* orthologous or paralogous) gene transcripts from *H. armigera* and *B. mori* indicated only minor differences in codon preference between three species.

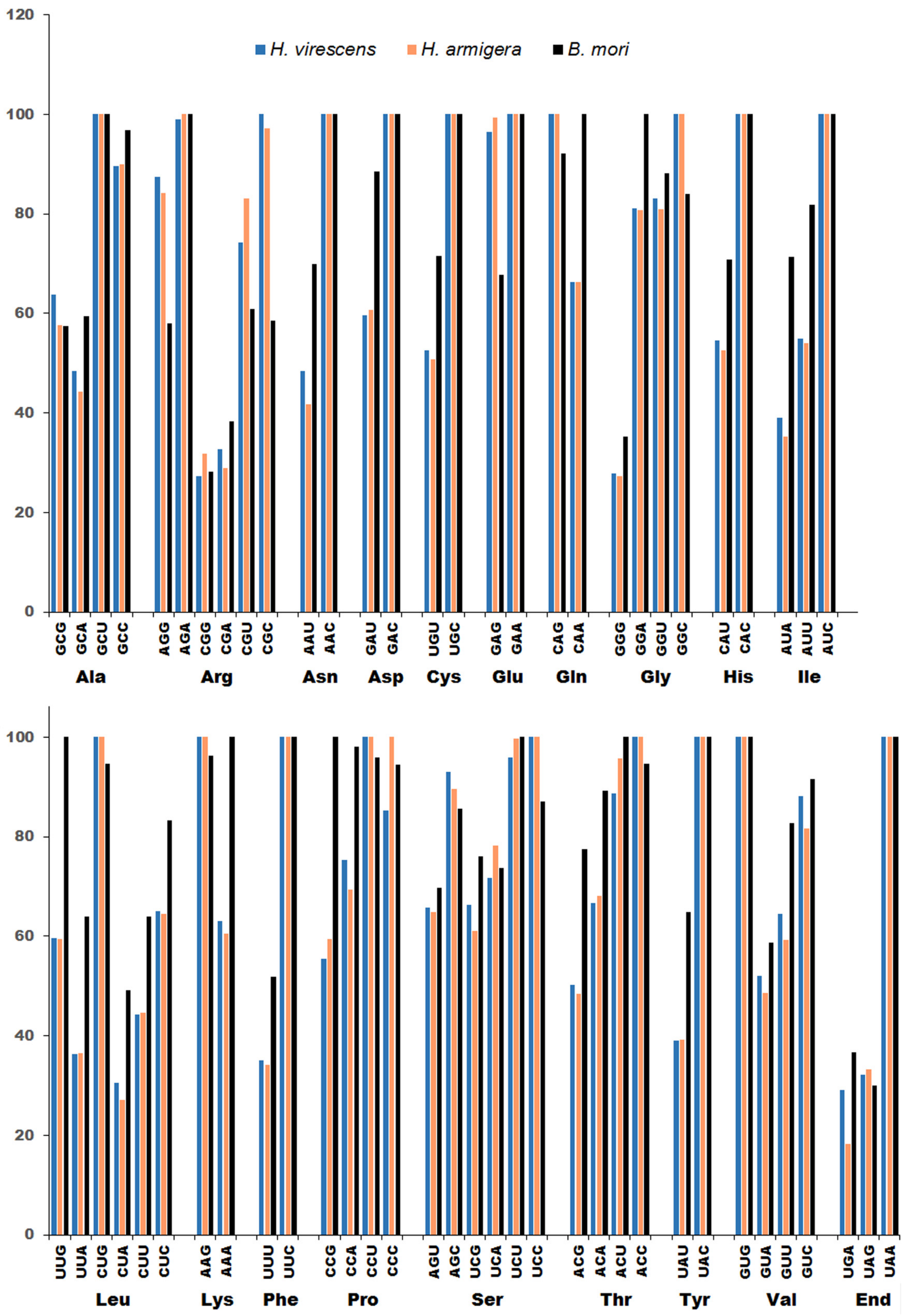

Fig 2. BLAST2GO annotation of assembled *Heliothis virescens* unigenes. A. Molecular function. B. Biological process. C. Cellular component. Computed with BLAST2GO.

doi:10.1371/journal.pone.0128563.g002

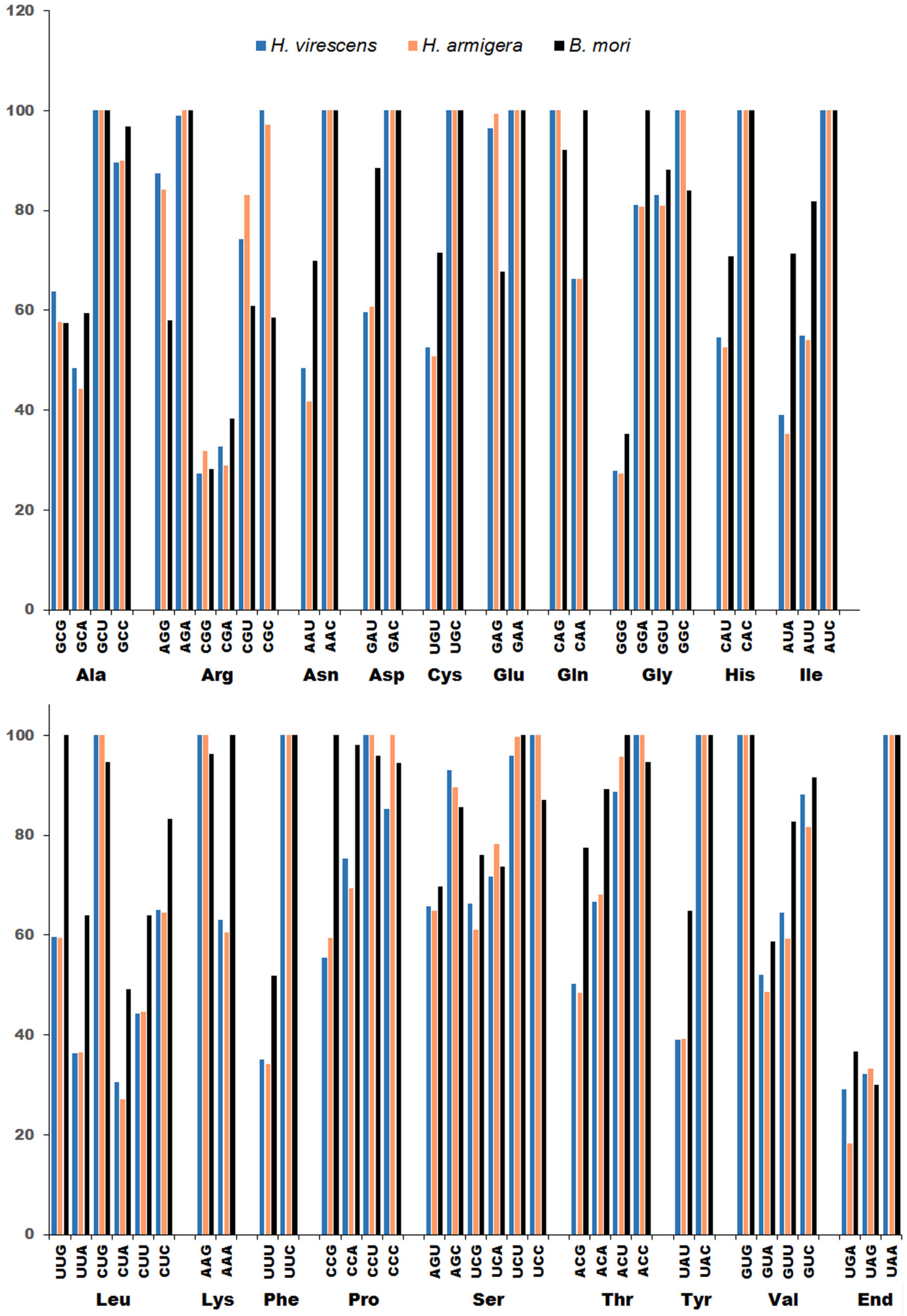

Fig 3. Comparison of codon usage in 50 full-length homologous coding sequences in *Heliothis virescens, Helicoverpa armigera*, and *Bombyx mori*. The annotated *H*. *virescens* transcriptome was used to select 50 full length open reading frames that also had full length homologous sequences for *H*.

armigera and *B*. *mori* in public databases. All selected sequences had E-values below -135. Relative adaptiveness of each degenerate codon was calculated for each open reading frame and the proportional codon usage of degenerate codons was calculated by averaging codon usage across all 50 sequences. The relative adaptiveness for each codon was calculated by setting the codon with highest usage fraction within each degenerate codon set to 100% and proportionately scaling the fractions of remaining codons. Non-degenerate codons AUG (Met) and UGG (Trp) were not used in these calculations.

doi:10.1371/journal.pone.0128563.g003

Hemocyte expressed genes

Molecular function (MF) and biological process (BP) GO terms significantly enriched in hemocytes ($p<0.01$, with Benjamini-Hochberg FDR) were identified, and the sequence contigs annotated with significant terms were selected using ArrayStar 5.0 software (DNAStar, Madison, WI). There were 4270 and 2951 transcripts with significant MF and BP GO terms, respectively, in the hemocytes (S3A Table, S3A Table, S3B Table, S3C Table and S3D Table). There were 554 and 533 sequence contigs annotated with significant molecular function terms for protein binding and binding, respectively. Significant BP GO terms for, translation, metabolic process, proteolysis, and oxidation/reduction were represented by 222, 209, 205, and 153 contigs, respectively. Previous smaller scale attempts to harvest immune response transcripts from an *H*. *virescens* hemocyte cDNA library identified many ESTs orthologous to known insect unigenes [17]. The present, much deeper sampling of the activated immune system resampled hemocytes, but also sampled other immune responsive tissues such as the fat body, cuticle and midgut. Within the biological processes, the immune system process GO term was significantly enriched in 85 hemocyte contigs, which were selected for further analysis (S3E Table and S3F Table). Orthologs of known pathogen-associated molecular pattern recognition molecules involved in signaling the presence of infectious microbes were identified in the *H*. *virescens* transcriptome (S3G Table). Interaction of these pathogen-associated molecular pattern receptors with cellular receptors results in the activation of signal transduction cascades leading to mobilization of the insect immune response [55,56]. Orthologs of receptors involved in the mobilization and coordination of immunity functions were identified within the *H*. *virescens* transcriptome assembly, such as cytokines, antimicrobial peptides, protease inhibitors, attacin, and lysozyme (S3H Table). *H*. *virescens* orthologs of several other immune system components participating in the encapsulation, melanization and clotting [17,50,51,57–61], including the amyloid-like precursor protein p102 [62] were identified in this assembly (S3I Table).

Antiviral immune response and siRNA

Gene silencing through RNA interference (RNAi) mechanisms has been demonstrated as an effective and highly specific insecticidal technology [5,63]. Orthologs identified in the *H*. *virescens* transcriptome included proteins participating in the micro RNA (miRNA) pathway, such as the nuclear microprocessor subunits *Drosha* and DGCR8, the nuclear membrane miRNA exporting protein exportin-5, and the cytoplasmic miRNA processing subunits loquacious, dicer-1 and argonaute-1 (S3J Table). Contigs encoding orthologs of components in the small interfering RNA (siRNA) pathway and Piwi-interacting RNA (piRNA) pathway were also identified. These included orthologs of dicer-2, the RISC subunit argonaute-2, argonaute-3, and aubergine protein (S3K Table).

Systemic RNAi may be possible through uptake of double stranded RNA (dsRNA) by an ortholog of the SID-1 protein identified in the *H*. *virescens* assembly. However, several insect species have been demonstrated to possess this ortholog but nonetheless lack a systemic RNAi response. Conversely proteins proposed to facilitate uptake of dsRNA into insect cells, such as scavenger receptors or lipophorins [64–66], were also present in the assembly (S3K Table). Future experimentation will be required to determine mechanisms and activity of these *H*. *virescens* orthologs in order to accomplish successful environmental RNAi in this pest species.

Midgut expressed genes

In order to identify transcripts unique to the midgut tissue we compared gene expression levels using RNA-Seq in whole insect, hemocytes and midgut tissues. Of the 7,765 transcripts with significant differential expression ($\geq$2-fold, p$\leq$0.01) between whole insect and midgut tissue, 1,895 showed $\geq$2-fold higher expression in the midgut. A larger number of differentially expressed genes (11,455) were detected when comparing hemocytes and midgut tissue, of which 5,334 were expressed $\geq$2-fold higher in the midgut. When considering the intersection of the three comparisons between pairs of samples (whole body *vs*. midgut, whole body *vs*. hemocyte, and midgut *vs*. hemocytes), 1,464 transcripts were identified as common (Fig 4). There were 1104 sequence contigs highly expressed in the midgut ($\geq$2-fold, P<0.01) compared to the other two samples (S4A Table), although only 296 transcripts had annotations. Among these transcripts with significantly higher expression (>2-fold, p<0.01) in the midgut, we detected 13 aminopeptidases, four cadherin–like proteins, 107 proteases (chymotrypsins, trypsins, and serine proteases), 35 carboxyl esterases, 37 cytochrome P450 monooxygenases, three amino acid transporters, and four each ABC class B and C transporters (S4 Table).

In an alternative strategy to identify midgut transcripts with significant GO terms, filtering with log base 2 expression value $\geq$0 selected a total of 32,418 contigs, of which 15,177 had homology to at least one gene in the database. Gene ontology (GO) analysis of GO terms with a nominal P-value $\leq$0.01 and Benjamini-Hochberg FDR correction for multiple testing was performed to identify significant molecular function and biological process GO terms in the selected set of gene transcripts (S4B Table and S4C Table). Within the subset of resulting 4,573 selected gene transcripts, there were 91 statistically significant (p$\leq$ 0.01 with FDR) biological process GO terms associated with 3,304 sequence contigs (S4C Table). There were 131 significant molecular function GO terms in the 4,751 gene transcripts at p$\leq$0.01 out of 6,392 transcripts annotated with one or more molecular function GO term (S4D Table). The number of sequence contigs annotated with different level 3 molecular function GO terms is given in Fig 2A. As would be expected from the function of the midgut tissue, the majority of transcripts (38.2%) had molecular function GO terms associated with digestive functions. Most of the transcripts (511 and 500 sequences, respectively) were annotated with the molecular function GO terms protein binding and binding. Other common molecular function GO terms denoting digestive function were hydrolase, peptidase/endopeptidase, and catalytic activities (277, 272, and 255 transcripts, respectively). There were also 237 sequence contigs annotated with the molecular function GO term "structural constituent of ribosome" representing ribosomal proteins (S4E Table).

The majority of digestive enzymes in the midgut of lepidopteran larvae are serine proteases [67]. Most of the sequence contigs matching to chymotrypsin-like, trypsin-like, and other serine protease enzymes in the databases were selected in significant molecular function GO terms (p$\leq$ 0.01 with FDR) in the midgut. Thus, out of the 60 chymotrypsin-like, 68 trypsin-like, and 99 serine protease sequence contigs found in the transcriptome, 51, 54, and 56, respectively, were selected in the midgut with significant molecular function GO terms (S4D Table) and 111 of these proteases had two-fold or higher expression in the midgut compared to whole body and hemocyte samples (S4A Table). Among the 73 sequence contigs annotated with GO terms for various protease inhibitor activities in the transcriptome, none showed elevated expression in the midgut compared to the other two tissues used in the study.

An additional function associated with the midgut tissue is the detoxification of xenobiotics. As expected, several enzyme classes involved in detoxification and xenobiotic processing mechanisms were also identified in the transcriptome assembly of *H. virescens* and many of them were highly expressed in the midgut. Members of four main enzyme superfamilies, carboxyl/

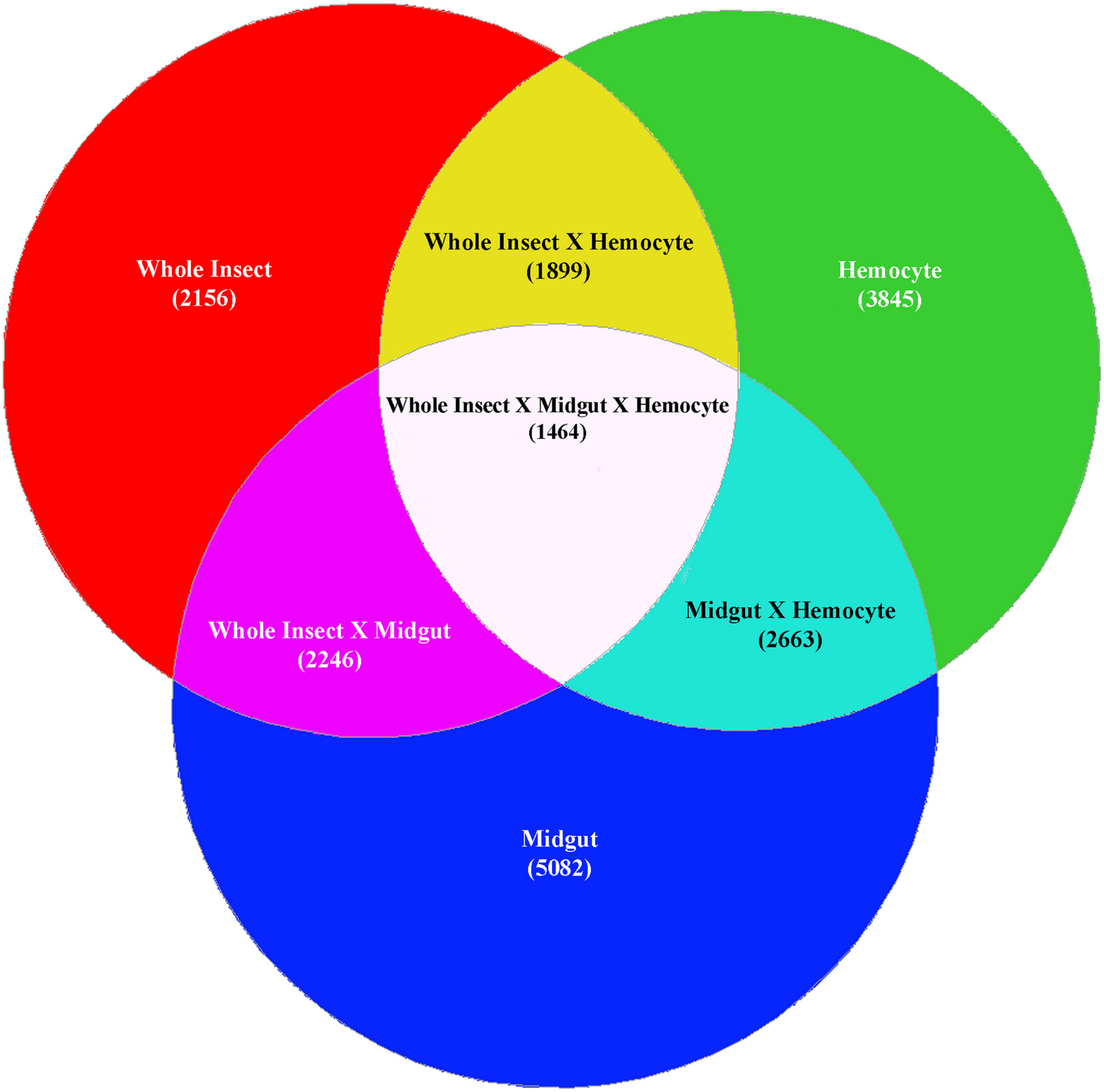

Fig 4. RNA-Seq determination of transcript expression level differences between tissues. Venn diagram demonstrating expression differences between RNA-Seq reads from whole insect, midgut, and hemocyte of *H*. *virescens*. The number of transcripts with significantly different expression levels ($\geq$2-fold, p$\leq$0.01) are shown within parenthesis.

doi:10.1371/journal.pone.0128563.g004

cholinesterase (CCE), carboxyl esterases, glutathione-S-transferases (GSTs), and cytochrome p450 monooxygenase (CYP450), were represented in the transcriptome and some of the contigs had high expression in the midgut. We identified 136 sequence contigs matching the carboxyl/cholinesterase (CCE) superfamily, which include functionally diverse groups of enzymes involved in xenobiotic degradation, neuronal development, and degradation of hormones and pheromones [68,69]. It should be noted that there were 20 sequence contigs matching database

entries classified as variants of "antennal esterase" among these sequence contigs. Out of the total 136 sequence contigs, 65 midgut expressed sequence contigs were annotated with significant molecular function GO terms. Eleven of these 65 esterases represented the antennal esterases with expression values ranging from 0.93 (stdev±0.21) to 5.49 (stdev±0.29) (S4D Table). Forty of the esterase contigs had two-fold or higher expression in the midgut compared to both whole body and hemocyte (S4A Table). In the case of GSTs, we detected 64 sequence contigs in the transcriptome, of which 32 were annotated with significant molecular function GO terms in the midgut with expression values from 0.12 (stdev±0.55) to 7.81 (stdev±0.42) [68,69]. Among the 112 esterase contigs, there were 23 sequence contigs matching database entries classified as variants of "antennal esterase". Out of the total 112 sequence contigs, 66 were significantly enriched in the midgut, with 11 of them representing the antennal esterases with expression values ranging from 0.92 to 5.49. There were 83 sequence contigs matching carboxylesterase, carboxyl esterase or carboxyl/choline esterase entries in the databases, of which 53 were enriched in the midgut with expression levels ranging from 0.51 to 9.54. In the case of GSTs, we detected 51 sequence contigs expressed in the midgut, of which 48 were enriched in that tissue with expression values from 0.12 to 7.85. The GST classes identified in the larval midgut of *H. virescens* included *delta*, *epsilon*, *omega*, *sigma*, *tau*, and *zeta* as well as a few unclassified entry matches. For CYP450s, we detected 197 sequence contigs in the transcriptome representing a wide range of CYP450 classes, including 4, 6, 9, 304, 306, 321, 324, 332, 333, 340, and 354. There were 56 CYP450 contigs enriched under molecular function GO terms in the midgut with expression levels ranging from 0.44 (±0.08) to 7.10986 (±0.51). Sequences matching CYP450 classes 4, 6, and 9, were most prevalent (76.8% or 43 out of 56) among the sequences enriched in the midgut (S4D Table). There were 11 CYP450 contigs with greater than two-fold expression in the midgut compared to both whole body and hemocyte (S4A Table).

Cry toxin midgut receptors

Larvae of *H. virescens* are targeted by biopesticides and transgenic crops containing Cry insecticidal proteins from the bacterium *Bacillus thuringiensis* (Bt). These Cry proteins bind to specific midgut proteins, and much effort has been devoted to the identification of these Cry toxin receptors and to characterize the Cry intoxication process [70]. Since high levels of resistance to Cry toxins are documented to involve alterations in receptor genes, efforts to identify Cry receptors are crucial in designing improved insecticidal proteins and effective resistance management strategies.

A number of laboratory strains of *H. virescens* have been documented to display high levels of tolerance to Cry toxins due to mutations or down-regulation of diverse receptor genes, such as cadherins [71], ATP binding cassette (ABCC) transporters [72], or membrane-bound alkaline phosphatases [73]. In addition, aminopeptidase proteins have also been reported as putative Cry toxin receptors in *H. virescens* [74,75]. Availability of the *H. virescens* transcriptome allows for the identification of putative Cry toxin receptor isoforms, and for determining their variability. Contigs representing proteins previously shown to bind Cry1Ac toxin (the most active Cry protein against *H. virescens*), such as membrane-bound alkaline phosphatase (mALP) [76,77], aminopeptidases [75,78,79], ABC transporters [72], and cadherins [77], were detected in the *H. virescens* transcriptome assembly.

Alkaline phosphatases

Among the 19 contigs from the assembly that matched to ALP sequences in databases, seven had greater than two-fold ($P<0.01$) expression in the midgut compared to whole body and

hemocyte (S4A Table). Eight ALP contigs were >200 amino acids in length and three of these represented full-length ALP sequences (Hv_Contig_1328, Hv_Contig_1343 and Hv_Contig_3366). Full length contigs Hv_Contig_1328 and Hv_Contig_3366 were enriched in the midgut compared to the whole body (S4A Table). All contigs >200 amino acids were used in sequence alignments and to construct phylogenetic trees with ALP proteins previously shown to interact with Cry1Ac or have altered expression in Cry1Ac-resistant insects (Fig 5). All the full-length ALP sequences and an almost complete transcript (Hv_Contig_5430) displayed 87–93% sequence identity and clustered in phylogenetic trees with *H*. *virescens* (ACP39712.1) and *H*. *armigera* mALP proteins previously reported as putative Cry1Ac binding sites involved in resistance to this toxin [73,76,77,80]. However, this contig had a very low level expression in the midgut and was not statistically significant. An adjacent cluster included two contigs possibly representing allelic ALP variants (97% identity) that displayed slightly lower identity (89%) to ACP39712.1. Based on the high sequence identity among all these contigs (89–98%), it is highly plausible that at least some of them represent allelic variants of the same gene, or isoforms from duplicated ALP genes. A second major cluster in the phylogenetic tree included partial contigs Hv_Contig_15532 and Hv_Contig_15507, which were grouped with soluble (68% identity) and membrane-bound (76% identity) forms, respectively, of ALP from *B*. *mori* (Fig 5). These observations support the variability in ALP isoforms in the *H*. *virescens* midgut and suggest that not all isoforms may contain Cry1Ac-binding sites, although further functional analyses would be needed to test this hypothesis.

ABCC transporters

Out of the 17 contigs in the assembly matching to ABCC transporters from databases, only five were >200 amino acids in length and were used for alignments and to construct phylogenetic trees with ABCC proteins proposed to be involved in Cry intoxication (Fig 6). In these trees, three main clusters were observed, one of them including proteins with reported relation to the Cry intoxication process. Proteins in this main cluster included ABCC2 transporters from *H*. *virescens*, *Trichoplusia ni* and *Spodoptera exigua* previously reported either as Cry1Ac binding proteins or as being associated with resistance against Cry toxins [72,81,82]. This cluster also included sequence contig Hv_Contig_327, representing the *H*. *virescens* ABCC2 protein ADH16740.1 (99.8% identity) and being most abundant in gut tissue with 2.89 (stdev±0.25) and 1023.81 (stdev±0.56) fold higher expression compared to whole body and hemocyte, respectively, as would be expected from a putative Cry1Ac receptor (S4A Table). Also in the same cluster was Hv_Contig_8943 with >93% identity to the *T*. *ni* and *S*. *exigua* sequences and showed significantly elevated expression in the midgut compared to whole body and hemocyte (S4A Table). As noted for ALP isoforms, it is highly plausible that this high sequence identity detected for ABCC2 contigs signifies allelic variants or products from duplicated genes. Lower identity (<74%) was detected between these ABCC2 contigs and ABCC2 transporters from *B*. *mori* and *Plutella xylostella* within the same group cluster. A second cluster included an ABC family transcript (Hv_contig_265) and an ABCC3 transporter from *S*. *exigua*, with 85% sequence identity observed between them. Sequence contig Hv_Contig_265 was also highly expressed in the midgut and showed greater than two-fold higher expression than the whole body and hemocyte (S4A Table). The third cluster included two contigs that were very different (<25% identity) from all the other sequences considered, and that did not have enriched expression in midgut compared to the whole body. The low homology and lack of localized expression suggest diverse physiological roles for ABC transporters in this cluster compared to the rest of analyzed ABC transporter contigs.

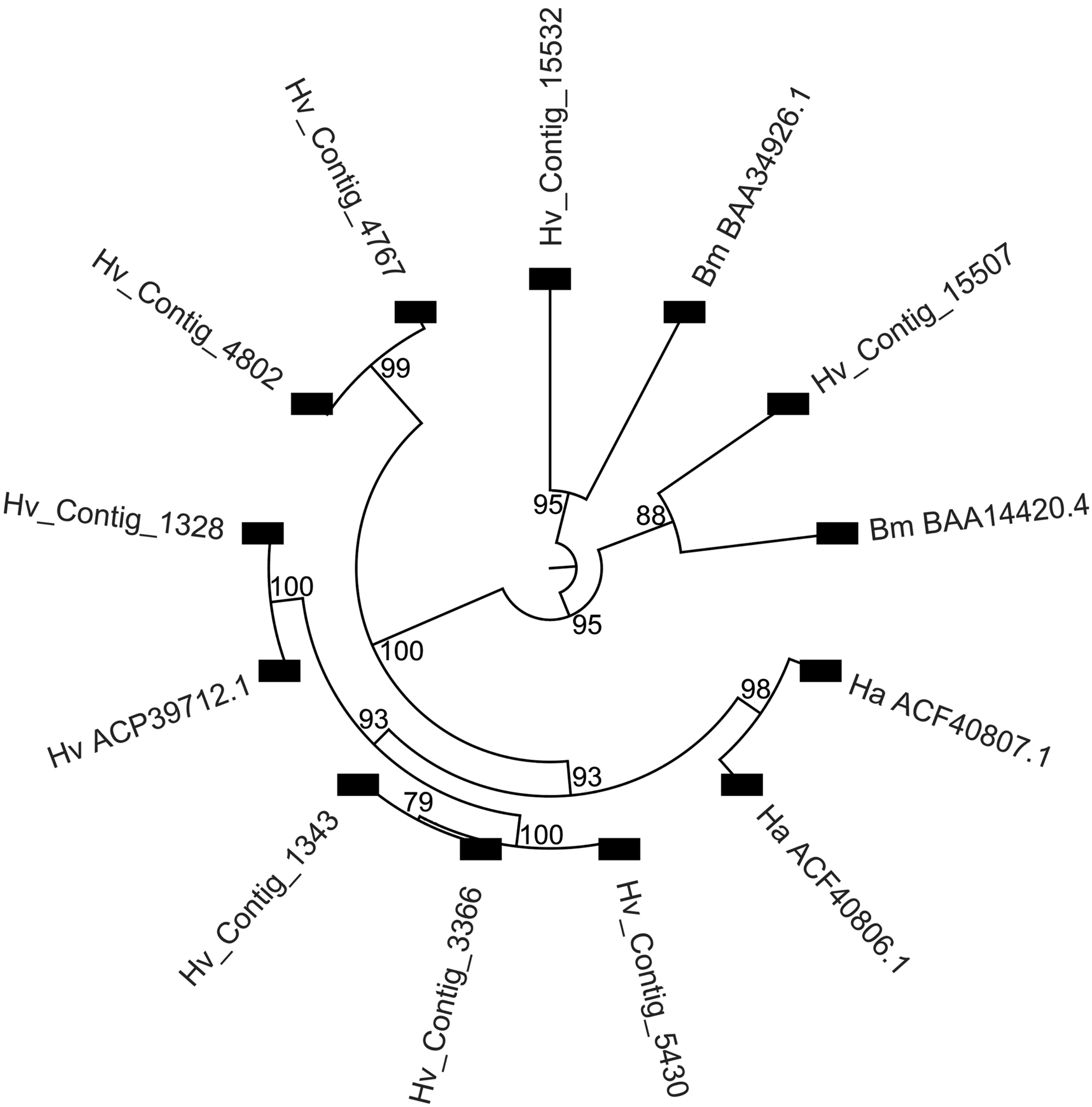

Fig 5. Phylogenetic tree derived from alignment of alkaline phosphatase (ALP) contigs in the *H. virescens* assembly and ALP sequences from databases. Sequences from databases were selected based on evidence of interactions with Cry toxins [76,80] or because membrane-bound and soluble forms have been described [102,103]. The phylogram only displays nodes above a 70% bootstrap threshold (1,000 replicates), numbers in nodes represent bootstrap values. Lepidopteran ALPs used include proteins from *Heliothis virescens* (ACP39712.1), *Helicoverpa armigera* (ACF40806.1 and ACF40807.1), and *Bombyx mori* (BAA34926.1 and BAA14420.4).

doi:10.1371/journal.pone.0128563.g005

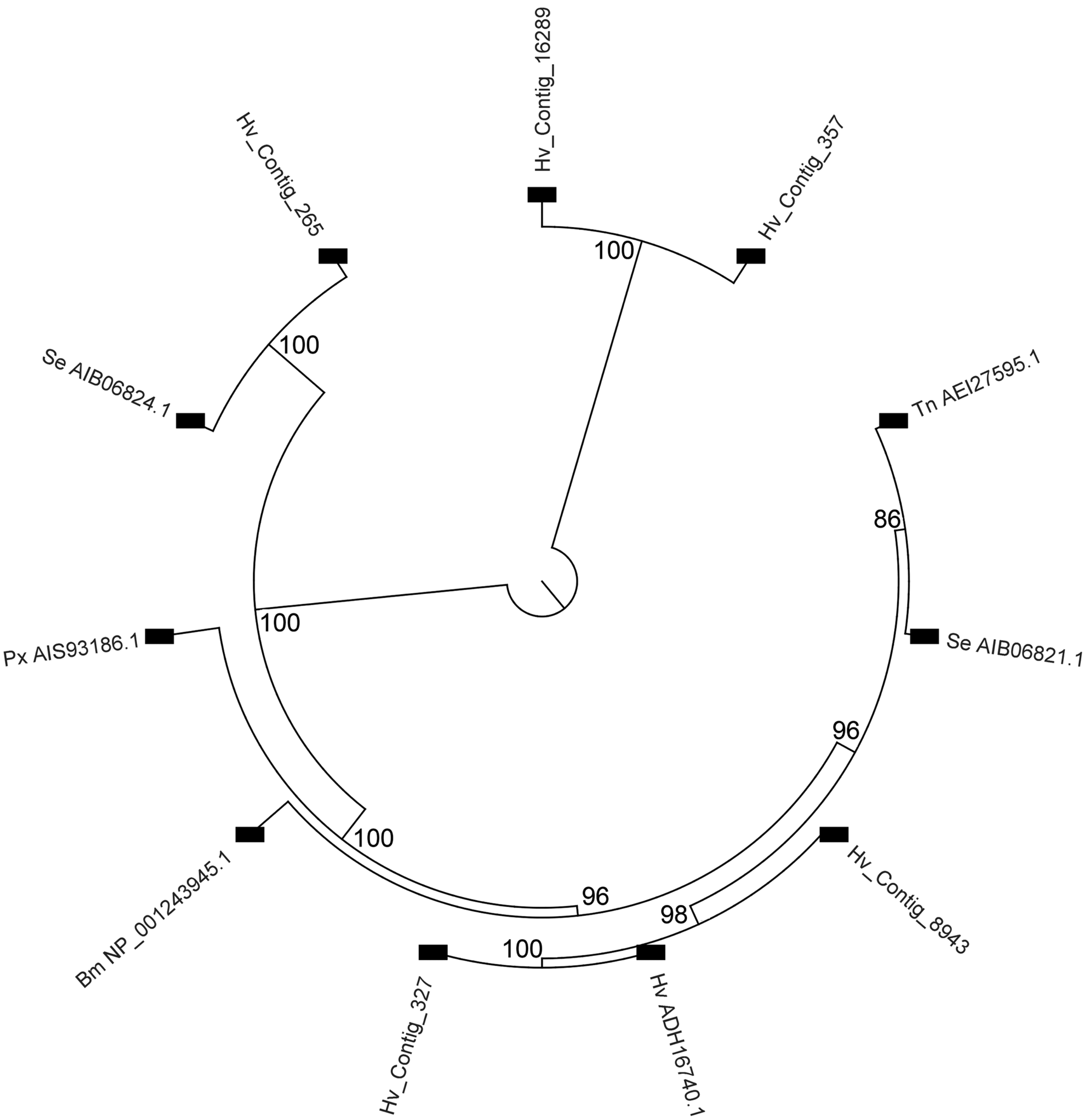

Fig 6. Phylogenetic tree derived from multiple sequence alignments of ABC transporter contigs from the *H. virescens* transcriptome assembly and reported lepidopteran ABC transporter proteins with Cry toxin receptor function [72,81,104,105]. The cladogram only displays nodes above a 70% bootstrap threshold (1,000 replicates), numbers on nodes indicate bootstrap values. Lepidopteran Cry toxin ABCC2 receptors used include proteins from *Heliothis virescens* (ADH16740.1), *Plutella xylostella* (AIS93186.1), *Trichoplusia ni* (AEI27595.1), *S. exigua* (AIB06824.1 and AIB06821.1) and *B. mori* (NP_0012439451.1).

doi:10.1371/journal.pone.0128563.g006

Aminopeptidases

A total of 67 predicted aminopeptidase sequences were detected in the *H. virescens* assembly (S4A Table), which included alanyl (most abundant with 36 contigs), aspartyl (1), glutamyl (2), leucyl (3), methionyl (8), and prolyl (8) aminopeptidases plus nine unclassified aminopeptidases (S1 Table). Expression levels (log base 2) of aminopeptidases in the midgut ranged from no expression to 11.49 (Stdev±0.42), with sequence contig Hv_Contig_818 displaying the highest relative level of expression. Among the sequence contigs that were highly expressed in the midgut, 19 showed significantly higher expression ($\geq$2-fold, P<0.01) in the midgut compared to whole body and hemocyte. The APN coded by Hv_Contig_786 with 2.22 (±0.16)-fold increase in the midgut compared to whole body was at the lowest end while the APN coded by Hv_Contig_818 (with 99% identity to *H. virescens* APN AF173552_1) with 23.38 (±0.37)-fold increase over whole body represented the highest relative midgut expression. All 19 aminopeptidase contigs with significantly higher expression in the midgut were either minimally expressed or not detected at all in the hemocytes (S3E Table and S4A Table).

Out of the 67 aminopeptidase contigs identified, 39 were identified as enriched in the midgut with significant biological process GO terms (S4C Table and S4E Table). Of the total number of aminopeptidase contigs, 30 were >200 amino acids in length and were used for alignment and tree construction with previously reported APN proteins proposed as Cry toxin receptors [83]. In the resulting tree (Fig 7), a number of clusters were observed mostly representing APN classes previously reported [83]. Thus, a cluster including APN Class 2 proteins [83] included two contigs (Hv_Contig_2713 and Hv_Contig_920) that were >98% identical between them and displayed up to 69% identity to APNs reported as Cry1Ab binding proteins from *M. sexta* (CAA66466.2) and *P. xylostella* (CAA66467.1) [84]. Hv_Contig_920 had 10.5 (stdev ±0.29)-fold higher expression level in the gut compared to the whole body and the expression level in the hemocytes was negligible (S4A Table). However, the expression level of Hv_Contig_2173 was not different from that of whole body. An *H. virescens* APN in Class 3 (Q11000.1) previously reported as a Cry1Ac binding protein [78] clustered, and was identical (>99%), to contigs Hv_Contig_666 and Hv_Contig_667. An alternative *H. virescens* APN in Class 1 (AF173552_1) that was previously reported as a receptor shared by all Cry1A toxins [79] was identical (>97%) and clustered with two contigs (Hv_Contig_818 and Hv_Contig_997). Three alternative contigs were identified as representing (>97% identity) the Class 4 *H. virescens* 110 kDa APN (AAK58066.1) previously reported to act as a binding site for Cry1Ac and Cry1Fa toxins [75]. Interestingly, this binding was not conducive to toxicity [85], which supports that even though some of the Class 4 APN contigs present have relatively high levels of expression in the midgut, their interactions with Cry1Ac toxin are probably irrelevant to toxicity. Two contigs (Hv_Contig_786 and Hv_Contig_18327) clustered with low identity (<65%) with a Class 5 Cry1Ac-binding APN from *P. xylostella* (CAA10950.1). Similar identity (~60%) was observed between a Class 8 APN (ACV04931.1) previously reported as Cry1Fa binding protein in *O. nubilalis* [86] and Hv_Contig_1103 (Fig 7). These observations demonstrate the existence of genes from all the APN Classes previously reported to contain Cry toxin receptor APNs in the current *H. virescens* transcriptome. As expected from Cry toxins targeting midgut cells, all APN contigs clustering with previously reported Cry toxin receptor APNs had enriched relative expression in the midgut tissue, in contrast to APN contigs grouped in alternative clusters which did not have high expression in the gut.

Cadherins

Resistance to Cry1Ac toxin in *H. virescens* has been linked to mutant cadherin alleles [71,77]. We identified 61 sequence contigs in the transcriptome that matched database

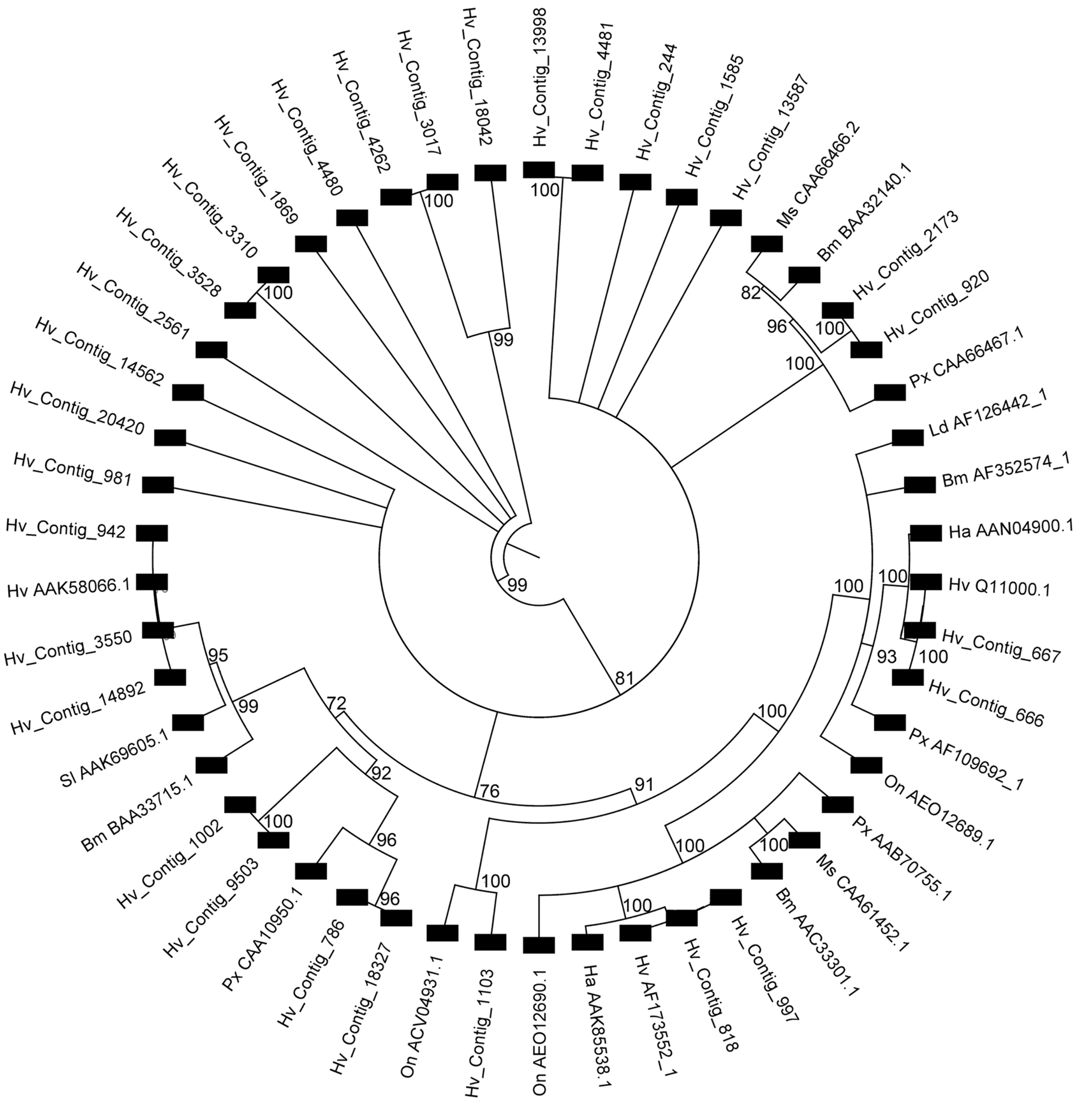

Fig 7. Phylogenetic tree derived from multiple sequence alignments of aminopeptidase (APN) contigs from the *H. virescens* transcriptome assembly and reported lepidopteran APN proteins with Cry toxin receptor function [83]. Alignments included proteins from *Heliothis virescens* (Q11000.1, AF173552_1, and AAK58066.1), *Helicoverpa armigera* (AAN04900.1, and AAK85538.1), *Plutella xylostella* (AF109692_1, AAB70755.1, CAA10950.1, and CAA66467.1), *Lymantria dispar* (AF126442_1), *Bombyx mori* (AF352574_1, AAC33301.1, BAA33715.1, and BAA32140.1), *Ostrinia nubilalis* (AEO12689.1, AEO12690.1, and ACV04931.1), *S. litura* (AAK69605.1), and *Manduca sexta* (CAA61452.1, and CAA66466.2). The cladogram only displays nodes above a 70% bootstrap threshold (1,000 replicates). Numbers on nodes are bootstrap values.

doi:10.1371/journal.pone.0128563.g007

entries for cadherin-like or mutant cadherin, and 17 of them were enriched in the midgut under molecular function GO terms (S4D Table). However, only 16 of these contigs encoded fragments that were longer than 200 amino acids and were used with previously reported Cry1A-receptor cadherins in phylogenetic analyses. In the derived tree four major clusters were observed (Fig 8). Only one of these clusters included previously reported Cry1A-receptor cadherin proteins, while contigs in the other three clusters displayed no homology (<21% identity) to any of the Cry1A receptors. Moreover, contigs in clusters not including Cry1A-receptor cadherins did not have increased expression in midgut compared to whole body (S4A Table). Six assembly contigs clustered among the reported Cry1A receptor cadherins, more specifically in proximity to the *H. virescens* cadherin (AAV80768.1) reported as Cry1A binding protein [87,88] with altered expression in Cry1A-resistant larvae [71,89]. While one of these contigs (Hv_Contig_117) only displayed 47% identity to AAV80768.1, the other five contigs displayed >91% sequence identity to that protein and may represent polymorphisms in agreement with previously reported highly variable cadherin transcript production in Lepidoptera [90,91]. Interestingly, the most contig most like AAV80768.1 (Hv_Contig_115, >98% identity), had lower relative expression in midgut compared to the whole body. This observation is in contrast to Hv_Contig_15 in a very close phylogenetic cluster (94% identity to AAV80768.1), which had 11.4 (±0.65)-fold higher relative expression in midgut tissue compared to whole body (S4A Table). Taken together, these observations support that almost identical cadherin transcripts have localized expression, suggesting that they may have distinct physiological roles. Despite different levels of identity at the whole sequence level and expression levels, high identity was observed when comparing the Cry1Ac toxin binding region in AAV80768.1 [88] in other Cry1A-receptor cadherins and the five *H. virescens* contigs in the same tree cluster (Fig 9). Interestingly, this short region was not detected in any of the predicted cadherin contigs that did not cluster with Cry1A-receptor cadherins or that were not included in the phylogenetic analysis (total 23 contigs), suggesting that a limited subset of cadherin genes in *H. virescens* encode Cry1A toxin receptors.

Conclusions

By combining new transcriptomic resources generated using Illumina and Roche 454 platforms, with existing published and unpublished Sanger-generated ESTs, we have assembled, annotated, and made public a *de novo* transcriptome of budworm. We expect that availability of this resource will enable detailed, and comprehensive studies of budworm immunobiology, pathogenomics, pathophysiology, digestion, reproduction, endocrinology, olfaction, diapause and biochemistry. Arguably, and based on the assembly output, the assembly in this work represents the most complete transcriptome currently available for any lepidopteran pest. This newly constructed transcriptome will also serve as a reference assembly for all future functional genomic studies of this pest species. The availability of extensive transcript data also enables high-throughput proteomic studies, which have been previously hindered by the lack of relevant genomic resources [13,92–94]. The availability of other tools such as inbred strains, cell lines, viral isolates, RNA-seq and microarray data, RNAi tools, and quantitative genetic markers [95,96], promotes *H. virescens* to the status of a "model" organism. We believe that these resources will support and accelerate biologically-based pest management of this and closely-related highly destructive moths in the

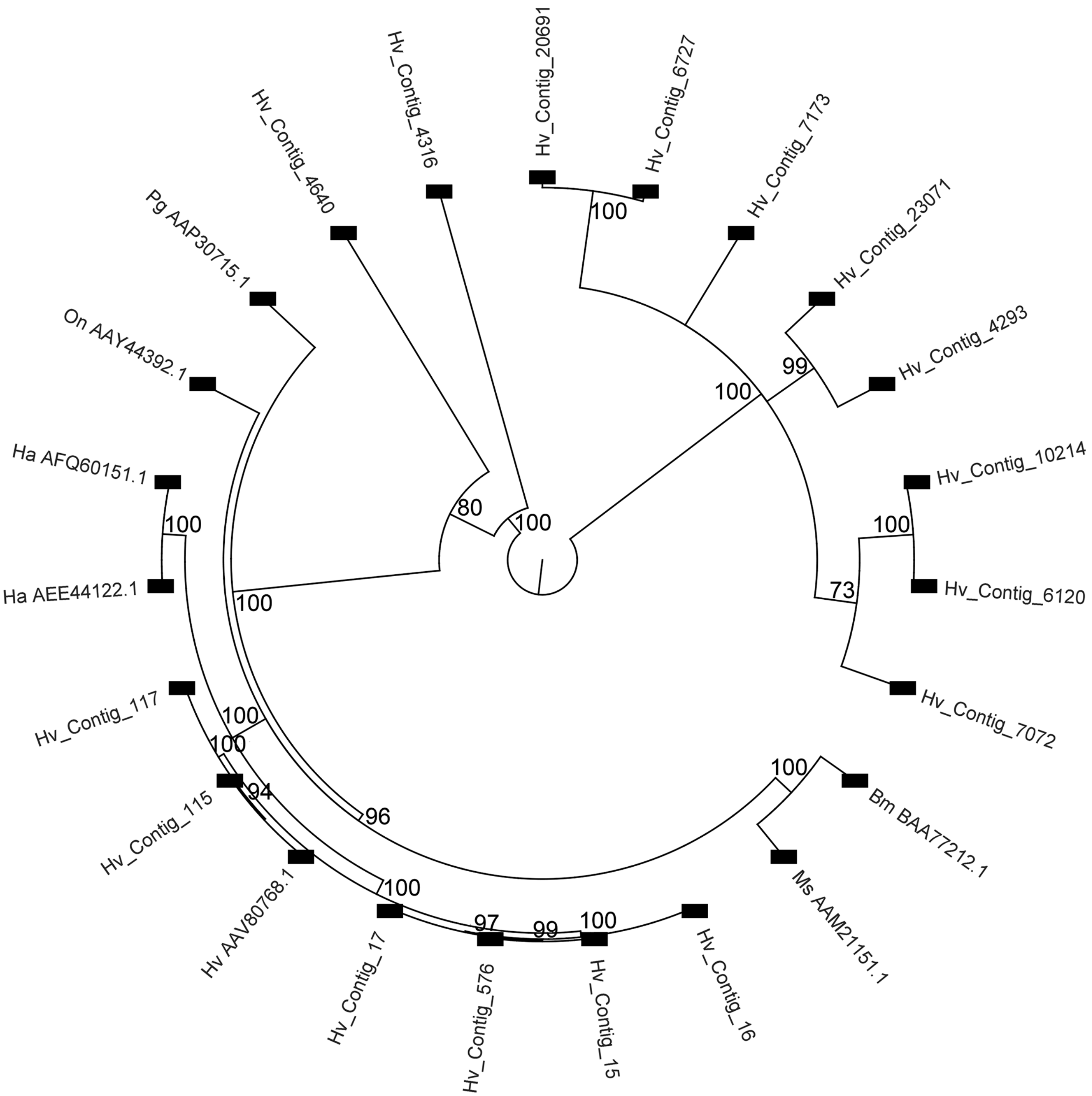

Fig 8. Phylogenetic tree derived from multiple sequence alignments of cadherin contigs from the *H. virescens* transcriptome assembly and lepidopteran cadherins with reported Cry receptor evidence [83]. Cry receptors used included *Bombyx mori* BtR175 (BAA77212.1), *Manduca sexta* BT-R1 (AAM21151.1), *Heliothis virescens* Cry1Ac receptor (AAV80768.1), *Helicoverpa armigera* isoforms (AFQ60151.1 and AEE44122.1), *Pectinophora gossypiella* cadherin (AAP30715.1), and *Ostrinia nubilalis* OnBt-R(1) (AAY44392.1). The cladogram only displays nodes above a 70% bootstrap threshold (1,000 replicates). Numbers on nodes are bootstrap values.

doi:10.1371/journal.pone.0128563.g008

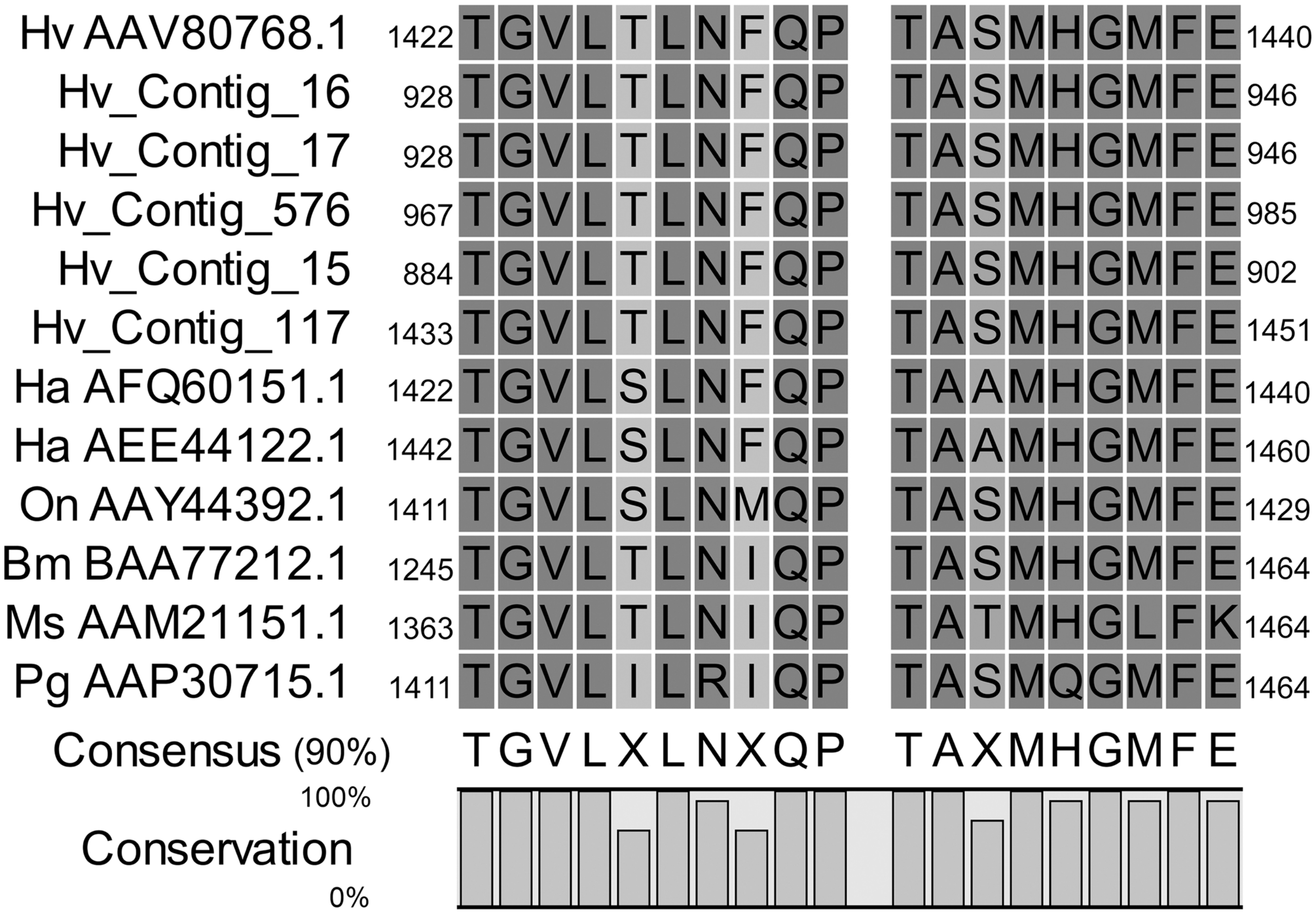

Fig 9. Alignment of the Cry1Ac-binding region of *H. virescens* cadherin receptor in selected assembly contigs and lepidopteran cadherins. The Cry1Ac-binding region in *H. virescens* cadherin [88] was compared with cadherins with reported Cry toxin binding evidence that were selected for phylogenetic analyses. Numbers at the both ends of sequences indicate amino acid position in the full length sequence.

doi:10.1371/journal.pone.0128563.g009

heliothine group, as well as allow testing of fundamental biological questions using *H. virescens* as a model.

Materials and Methods

H. virescens tissue RNA pools

All insects used in this study were laboratory colonies of *H. virescens* without any tolerance to chemical insecticides or *Bacillus thuringiensis* crystalline toxins. Hemocytes were prepared from early 5th instar larvae as previously described [60]. Midguts of late 4th instar *H. virescens* larvae fed control diet or diet surface-contaminated with 1 μg/ml of purified Cry1Ac activated toxin were dissected and extraneous tissue attached to midguts was carefully removed and pooled with tissues from the remainder of the body. Dissected midguts were placed in RNA*later* (ambion.com) overnight at 4°C or snap-frozen in liquid nitrogen. Total RNA was extracted from hemocytes or midgut tissue using RNeasy kits (qiagen.com), following manufacturer's instructions. Isolated total RNA was subjected to DNase treatment to remove any residual DNA. Total RNA was also extracted from different life stages from egg to adult using TriZol reagent

(invitrogen.com). Total RNA yields ranged from approximately 500 μg (eggs) to over 2 mg (midgut). The mRNA was isolated from 500 μg of pooled total RNA samples using the PolyA (+)-Track mRNA purification kit (www.promega.com). Pheromone glands were dissected from two to seven day-old PBAN treated *H. virescens* females (YDK strain). TransPlex Whole Transcriptome Amplification (WTA1) and the Complete Whole Transcriptome Amplification Kits (WTA2) (sigmaaldrich.com/life-science/molecular-biology/whole-genome-amplification/whole-transcriptome.html#sthash.BQ5bg7ni.dpuf) were used to prepare extracted pheromone gland RNA. Samples were submitted for sequencing to the North Carolina State University Genomic Sciences Laboratory.

Library construction and sequence generation

For Sanger sequencing, degenerate forward primers amplifying trypsin and chymotrypsin transcripts were used with oligo dT as reverse primer to generate PCR amplicon fragments of 500~700bp in size. Amplicons were gel-purified using a QIAquick gel extraction kit (Qiagen, Valencia, CA), cloned into pGEM-TEasy vector (Promega, Madison, WI), and transformed into competent DH5α *Escherichia coli* (Invitrogen, Carlsbad, CA) that were plated onto LB agar plates containing 100μg/ml ampicillin. A total of 960 clones were randomly picked and sequenced from T7 and SP6 promoter ends using BigDye terminator chemistry (Applied Biosystems, Foster City, CA) on an ABI3700 capillary sequencer (Applied Biosystems). Sequencing in both directions resulted in sufficient sequence coverage to overcome failure or truncation of sequence due to the presence of polyA tails [97]. Additional *H. virescens* expressed sequence tags (ESTs) were kindly provided by Dr. Bruce Webb (Department of Entomology, University of Kentucky, Lexington, KY).

RNA-seq of hemocyte (42 nt) and midgut (50 nt) samples was performed by the DNA Core Laboratory of the University of Missouri DNA Core Facility (biotech.rnet.missouri.edu/dnacore/) according to the standard Illumina RNA-seq protocol (Part # 1004898 Rev. A, rev Sept 08) using an Illumina Genome Analyzer IIx. RNA quality was examined using the Experion Automated Electrophoresis system (www.bio-rad.com). The library prep was performed as described elsewhere [60,98,99] according to the manufacturer's instructions from the pooled PCR products. Short read (36 nt) Illumina Genome Analyzer II sequencing of mRNA pools was carried out by the National Center for Genomic Resources, (Santa Fe, NM) following standard protocols. Roche 454 sequence reads from fourth instar larval midgut and female pheromone gland tissues used in the transcriptome assembly were generated at the North Carolina State University Core Facility.

Sequence assembly and annotation

Short reads (36 and 42 nt) generated by Illumina Genome Analyzer IIx platforms, Sanger reads from various cDNA libraries, and Roche 454-sequence contigs assembled from midgut and pheromone gland mRNA sequences were used to assemble the transcriptome using SeqMan NGen v2.1 software (www.dnastar.com). Over 212 million reads obtained from 16 Illumina Genetic Analyzer II flow cell lanes were iteratively assembled in five cycles due to computer memory and software limitations. Sequences from four Illumina GAII lanes and 78,000 Sanger sequences were included in the initial assembly followed by four iterative assemblies adding data from 3 additional lanes and all unassembled reads from the previous assembly to each successive assembly. Assembly match percentage was set to 93% with a match window size, match spacing, mismatch penalty, and gap penalty set to 15, 10, 20, and 30, respectively. The 5th iterative assembly of Illumina sequences contained 28,859,147 reads assembled into 69,643 contigs. Cumulatively, 212,987,028 reads were used in five stages of assembly. A separate assembly was

created from Roche-454 sequence reads obtained from midgut and pheromone gland (216,420 and 155,282 reads respectively) with all parameters set as for Illumina iterative assemblies except the match percentage set to 85%. The SeqMan assembly file containing all assembled Illumina and Sanger reads was re-assembled with 9,273 Roche-454 contigs obtained from midgut and pheromone gland cDNA libraries using an 85% match rate.

Redundant sequence contigs in the transcriptome assembly were clustered using the CD-HIT_EST tool (weizhong-lab.ucsd.edu/cdhit_suite/cgi-bin/index.cgi?cmd = cd-hit-est) in the CD-HIT Suite. The transcriptome sequence file in FASTA format was used with a sequence identity cut-off value of 0.90. The longest sequence of each cluster was retained in the assembly by selecting and deleting all shorter sequences. The transcriptome assembly was further refined by removing sequence contigs shorter than 100 nucleotides [52].

Contigs and singletons resulting from this assembly were annotated using BLAST2GO v3.0 (www.blast2go.org) [53,100]. NCBI non-redundant database (nr) was initially interrogated using BLAST2GO v3.0. Sequences without blast hits were checked against local databases built with peptide sequences of annotated genomes of *Bombyx mori*, *Danaus plexippus*, and *Heliconius melapomne* (ncbi.nlm.nih.gov/genome/browse/). Annotation of KEGG orthologies (KOs) and metabolic pathway mapping was accomplished using the utilities provided by the Kyoto Encyclopedia of Genes and Genomes (www.genome.jp/kegg). All sequences have been deposited in the NCBI database (ncbi.nlm.nih.gov/genomeprj/49697) with accession number SRP005629.

Codon usage comparison

Annotated *H*. *virescens* transcriptome was used to select 50 full length open reading frames that also had full length homologous sequences for *H*. *armigera* and *B*. *mori* in public databases. All selected sequences had E-values below -135. Codon usage was calculated for each ORF using the Codon Usage utility of the Sequence Manipulation Suite (bioinformatics.org/sms2/codon_usage.html). The sum of codon numbers within each insect species was used to calculate average codon usage per 1000 codons, the proportional codon usage of degenerate codons, and the relative adaptiveness (RA) of each degenerate codon. Relative adaptiveness was calculated by setting the codon with highest usage fraction within each degenerate codon set to 100% and proportionately scaling the fractions of remaining codons. Non-degenerate codons AUG (Met) and UGG (Trp) were not used in these calculations.

Phylogenetic analyses

All sequence analyses, alignments and phylogenetic tree construction were performed in the CLC Genomics Workbench 7.5.1 software package (clcbio.com). Contigs annotated as alkaline phosphatase, ABC transporter, cadherin, or Aminopeptidase were selected from the *H*. *virescens* assembly and translated in the respective frame. Only sequences that were longer than 200 amino acids were selected for further analyses. Protein sequences in each functional subgroup were aligned with respective proteins sequences from the NCBInr database that have been previously reported as relevant to Cry intoxication in Lepidoptera. Sequences were aligned and the alignment used to construct an UPGMA tree using the Kimura protein distance measure correction and performing bootstrap analysis with 1,000 replicates. Trees were represented as circular phylograms with a bootstrap threshold for nodes of 70%.

RNA-Seq profiling

The transcriptome unigene set and available annotations for *H*. *virescens* were imported to ArrayStar 5.0 gene expression profiling software (DNAStar, Madison, WI). Illumina

HiSeq2000 and Genome Analyzer II sequence read files (36 to 50 nt) obtained from two replicates of whole insect and three replicates of larval midgut and hemocytes, containing 8–28 million reads per replicate were imported into the RNA-Seq experiment. Read density for each transcript was normalized for transcript read length using the RPKM (Reads per Kilobase of exon model per Million mapped reads) method [101]. Replicate groups containing sequence reads from each replicate experiment were created and the expression levels were normalized to an internal calibrator gene (Hv_Contig_26369, β-actin). This gene was selected based on uniform raw expression levels across the tissues used in the study as detected in initial analyses. Normalized expression values and standard deviations were calculated for each transcript. Transcripts with a linear expression value greater than or equal to an arbitrary cutoff value of 10^{-1} were considered as genes expressed in the gut and hemocyte tissue, and those with expression value below cutoff were not considered in the analysis. Unless otherwise noted, all expression level comparisons were filtered using Student's t-test at 95% confidence limit and a Benjamini-Hochberg false discovery rate (FDR) for multiple testing correction was applied where appropriate. Gene ontology (GO) terms and ID's of BLAST2GO annotated genes were imported into ArrayStar 5.0 to perform GO term enrichment analyses. Sequence contigs with linear expression values ≥ 0.1 and a significant correlation with GO terms of biological process and molecular function were filtered ($P \leq 0.05$ with Benjamini-Hochberg FDR correction for multiple testing) to identify groups of genes enriched in each tissue type.

Supporting Information

S1 Table. List of contigs and annotations of *Heliothis virescens* transcriptome assembly.
(XLSX)

S2 Table. Full-length open reading frames of *Bombyx mori*, *Helicoverpa armigera*, and *Heliothis virescens* used in comparative codon usage analysis and summary of codon usage data.
(PDF)

S3 Table. *Heliothis virescens* sequence contigs with immunity-related functions.
(XLSX)

S4 Table. RNASeq analysis of differential tissue expression.
(XLSX)

Acknowledgments

We thank Bruce Webb and Sarjeet Gill for sharing sequences, and Nathan Bivens and Sean Blake of the University of Missouri DNA Core Facility and Bill Spollen of the University of Missouri Informatics Research Core Facility for assistance with sequence generation and analysis. Larry Brown and Clavin A. Pierce provided technical assistance. Mention of trade names or commercial products in this article is solely for the purpose of providing specific information and does not imply recommendation or endorsement by the US Department of Agriculture. USDA is an equal opportunity provider and employer.

Author Contributions

Conceived and designed the experiments: OPP KSS HJRP. Performed the experiments: OPP KSS HJRP. Analyzed the data: OPP KSS. Contributed reagents/materials/analysis tools: OPP KSS HJRP FG MJA JLJF. Wrote the paper: KSS OPP.

References

1. Cho S, Mitchell A, Mitter C, Regier J, Matthews M, Robertson R (2008) Molecular phylogenetics of heliothine moths (Lepidoptera: Noctuidae: Heliothinae), with comments on the evolution of host range and pest status. Systematic Entomology 33: 581–594.
2. Albernaz KC, Silva-Brandao KL, Fresia P, Consoli FL, Omoto C (2012) Genetic variability and demographic history of *Heliothis virescens* (Lepidoptera: Noctuidae) populations from Brazil inferred by mtDNA sequences. Bulletin of Entomological Research 102: 333–343. doi: 10.1017/S0007485311000678 PMID: 22126989
3. Fitt GP (1989) The ecology of *Heliothis* species in relation to agroecosystems. Annual Review of Entomology 34: 17–52.
4. Heckel DG, Gahan LJ, Daly JC, Trowell S (1998) A genomic approach to understanding *Heliothis* and *Helicoverpa* resistance to chemical and biological insecticides. Philosophical Transactions of the Royal Society B 353: 1713–1722.
5. Baum JA, Bogaert T, Clinton W, Heck GR, Feldmann P, Ilagan O, et al. (2007) Control of coleopteran insect pests through RNA interference. Nature Biotechnology 25: 1322–1326. PMID: 17982443
6. Mao YB, Cai WJ, Wang JW, Hong GJ, Tao XY, Wang LJ, et al. (2007) Silencing a cotton bollworm P450 monooxygenase gene by plant-mediated RNAi impairs larval tolerance of gossypol. Nature Biotechnology 25: 1307–1313. PMID: 17982444
7. Hunter WB, VanEngelsdorp D., Hayes J, Westervelt, Glick E, Williams M, et al. (2010) Large-scale field application of RNAi technology reducing Israeli Acute Paralysis Virus disease in Honey Bees (*Apis mellifera*, Hymenoptera: Apidae). PLOS Pathogens 6: e1001160. doi: 10.1371/journal.ppat.1001160 PMID: 21203478
8. Parthasarathy R, Palli SR (2007) Developmental and hormonal regulation of midgut remodeling in a lepidopteran insect, *Heliothis virescens*. Mechanisms of Development 124: 23–34. PMID: 17107775
9. Loeb MJ, De Loof A, Gelman DB, Hakim RS, Jaffe H, Kochansky JP, et al. (2001) Testis ecdysiotropin, an insect gonadotropin that induces synthesis of ecdysteroid. Archives of Insect Biochemistry and Physiology 47: 181–188. PMID: 11462222
10. Jurat-Fuentes JL, Adang MJ (2006) The *Heliothis virescens* cadherin protein expressed in *Drosophila* S2 cells functions as a receptor for *Bacillus thuringiensis* Cry1A but not Cry1Fa toxins. Biochemistry 45: 9688–9695. PMID: 16893170
11. Kirkpatrick BA, Washburn JO, Volkman LE (1998) AcMNPV pathogenesis and developmental resistance in fifth instar *Heliothis virescens*. Journal of Invertebrate Pathology 72: 63–72. PMID: 9647703
12. Hoover K, Washburn JO, Volkman LE (2000) Midgut-based resistance of *Heliothis virescens* to baculovirus infection mediated by phytochemicals in cotton. Journal of Insect Physiology 46: 999–1007. PMID: 10802113
13. Jurat-Fuentes JL, Adang MJ (2007) A proteomic approach to study Cry1Ac binding proteins and their alterations in resistant *Heliothis virescens* larvae. Journal of Invertebrate Pathology 95: 187–191. PMID: 17467006
14. Brito LO, Lopes AR, Parra JRP, Terra WR, Silva-Filhoa MC (2001) Adaptation of tobacco budworm *Heliothis virescens* to proteinase inhibitors may be mediated by the synthesis of new proteinases. Comparative Biochemistry and Physiology 128B: 365–375.
15. Johnston KA, Lee MJ, Brough C, Hilder VA, Gatehouse AMR, Gatehouse JA (1995) Protease activities in the larval midgut of *Heliothis virescens*: Evidence for trypsin and chymotrypsin-like enzymes. Insect Biochemistry and Molecular Biology 26: 375–383.
16. Klocke JA, Chan BG (1982) Effects of cotton condensed tannin on feeding and digestion in the cotton pest, *Heliothis zea*. Journal of Insect Physiology 28: 911–915.
17. Shelby KS, Popham HJR (2009) Analysis of ESTs generated from immune-stimulated hemocytes of larval *Heliothis virescens*. Journal of Invertebrate Pathology 101: 86–95. doi: 10.1016/j.jip.2009.05.002 PMID: 19442669
18. Govind G, Mittapalli O, Griebel T, Allmann S, Bocker S, Baldwin IT (2010) Unbiased transcriptional comparisons of generalist and specialist herbivores feeding on progressively defenseless *Nicotiana attenuata* plants. PLOS One 5: e8735. doi: 10.1371/journal.pone.0008735 PMID: 20090945
19. Vogel H, Heidel A, Heckel DG, H, Groot AT (2010) Transcriptome analysis of the sex pheromone gland of the noctuid moth *Heliothis virescens*. BMC Genomics 11: 29. doi: 10.1186/1471-2164-11-29 PMID: 20074338
20. O'Neill ST, Dzurisin JDK, Carmichael RD, Lobo NF, Emrich SJ, Hellmann JJ (2010) Population-level transcriptome sequencing of nonmodel organisms *Erynnis propertius* and *Papilio zelicaon*. BMC Genomics 11: 310. doi: 10.1186/1471-2164-11-310 PMID: 20478048

21. Ewen-Campen B, Shaner N, Panfilio KA, Suzuki Y, Roth S, Extavour CG (2011) The maternal and early embryonic transcriptome of the milkweek bug *Oncopeltus fasciatus*. BMC Genomics 12: 61. doi: 10.1186/1471-2164-12-61 PMID: 21266083
22. Vera JC, Wheat CW, Fescemyer HW, Frilander MJ, Crawford DL, Hanski I (2008) Rapid transcriptome characterization for a nonmodel organism using 454 pyrosequencing. Molecular Ecology 17: 1636–1647. doi: 10.1111/j.1365-294X.2008.03666.x PMID: 18266620
23. Zhu H, Casselman A, Reppert SM (2008) Chasing migration genes: A brain expressed sequence tag resource for summer and migratory Monarch butterflies (*Danaus plexippus*). PLOS One 3: e1345. doi: 10.1371/journal.pone.0001345 PMID: 18183285
24. Nasonia Genome Working Group (2010) Functional and evolutionary insights from the genomes of three parasitoid *Nasonia* species. Science 327: 343–348. doi: 10.1126/science.1178028 PMID: 20075255
25. Honeybee Genome Sequencing Consortium (2007) Insights into social insects from the genome of the honeybee *Apis mellifera*. Nature 443: 931–949.
26. Xia Q, Guo Y, Zhang Z, Li D, Xuan Z, Li Z et al. (2009) Complete resequencing of 40 genomes reveals domestication events and genes in Silkworm (*Bombyx*). Science 326: 433–436. doi: 10.1126/science.1176620 PMID: 19713493
27. Cabrera AR, Donahue KV, Khalil KV, Scholl SMS, Opperman E, Sonenshine C, et al. (2011) New approach for the study of mite reproduction: The first transcriptome analysis of a mite, *Phytoseiulus persimilis* (Acari: Phytoseiidae). Journal of Insect Physiology 57: 52–61. doi: 10.1016/j.jinsphys.2010.09.006 PMID: 20888830
28. Margam VM, Coates BS, Bayles DO, Hellmich RL, Agunbiade T, Seufferheld MJ, et al. (2011) Transcriptome sequencing, and rapid development and application of SNP markers for the legume pod borer *Maruca vitrata* (Lepidoptera: Crambidae). PLOS One 6: e21388. doi: 10.1371/journal.pone.0021388 PMID: 21754987
29. Rotenberg D, Whitfield AE (2010) Analysis of expressed sequence tags for *Franliniella occidentalis*, the western flower thrips. Insect Molecular Biology 19: 537–551. doi: 10.1111/j.1365-2583.2010.01012.x PMID: 20522119
30. Bai X, Mamidala P, Rajarapu SP, Jones SC, Mittapalli O (2011) Transcriptomics of the Bed Bug (*Cimex lectularius*). PLOS One 6: e16336. doi: 10.1371/journal.pone.0016336 PMID: 21283830
31. Mittapalli O, Bai X, Mamidala P, Rajarapu SP, Bonello,P., Herms DA (2010) Tissue-specific transcriptomics of the exotic invasive insect pest emerald ash borer (*Agrilus planipennis*). PLOS One e13708.
32. Karatolos N, Pauchet Y, Wilkinson P, Chauhan R, Denholm I, Gorman K, et al. (2011) Pyrosequencing the transcriptome of the greenhouse whitefly, *Trialeurodes vaporariorum* reveals multiple transcripts encoding insecticide targets and detoxifying enzymes. BMC Genomics 12: 56. doi: 10.1186/1471-2164-12-56 PMID: 21261962
33. Wang X-W, Luan J-B, Li J-M, Bao Y-Y, Zhang C-X, Liu S-S (2010) *De novo* characterization of a whitefly transcriptome and analysis of its gene expression during development. BMC Genomics 11: 400. doi: 10.1186/1471-2164-11-400 PMID: 20573269
34. Xue J, Bao YY, Li BL, Cheng Y-B, Pen Z-Y, Liu H, et al. (2010) Transcriptome analysis of the brown planthopper *Nilaparvata lugens*. PLOS One 5: e14233. doi: 10.1371/journal.pone.0014233 PMID: 21151909
35. Bai X, Zhang W, Orantes L, Jun T-H, Mittapalli O, Rouf Mian MA (2010) Combining next-generation sequencing strategies for rapid molecular resource development from an invasive aphid species, *Aphis glycines*. PLOS One 5: e11370. doi: 10.1371/journal.pone.0011370 PMID: 20614011
36. Zhang F, H. G, Zheng H, Zhou T, Zhou Y, Wang S, et al. (2010) Massively parallel pyrosequencing-based transcriptome analyses of small brown planthopper (*Laodelphax striatellus*), a vector insect transmitting rice stripe virus (RSV). BMC Genomics 11: 303. doi: 10.1186/1471-2164-11-303 PMID: 20462456
37. Schwartz D, Robertson HM, Feder JL, Varala K, Hudson ME, Ragland GJ, et al. (2009) Sympatric ecological speciation meets pyrosequencing: sampling the transcriptome of the apple maggot *Rhagoletis pomonella*. BMC Genomics 10: 663.
38. Hahn DA, Ragland GJ, Shoemaker DD, Denlinger DL (2009) Gene discovery using massively parallel pyrosequencing to develop ESTs for the flesh fly *Sarcophaga crassipalpi*s. BMC Genomics 10: 234. doi: 10.1186/1471-2164-10-234 PMID: 19454017
39. Wang Y, Zhang H, Li H, Miao X (2011) Second-generation sequencing supply an effective way to screen RNAi targets in large scale for potential application in pest insect control. PLOS One 6: e18644. doi: 10.1371/journal.pone.0018644 PMID: 21494551

40. Grosse-Wilde E, Kuebler LS, Bucks S, Vogel H, Wicher D, Hansson BS (2011) Antennal transcriptome of *Manduca sexta*. Proceedings of the National Academy of Sciences USA 108: 7449–7454. doi: 10.1073/pnas.1017963108 PMID: 21498690

41. Vogel H, Altincicek B, Glockner G, Vilcinskas A (2011) A comprehensive transcriptome and immune-gene repertoire of the lepidopteran model host *Galleria mellonella*. BMC Genomics 12: 308. doi: 10.1186/1471-2164-12-308 PMID: 21663692

42. de la Paz Celorio-Mancera M, Courtiade J, Muck A, Heckel DG, Musser RO, Vogel H (2011) Sialome of a generalist lepidopteran herbivore: identification of transcripts and proteins from *Helicoverpa armigera* labial salivary glands. PLOS One 6: e26676. doi: 10.1371/journal.pone.0026676 PMID: 22046331

43. Mukherjee K, Altincicek B, Hain T, Domann E, Vilcinskas A, Hansson BS (2010) *Galleria mellonella* as a model system for studying *Listeria* pathogenesis. Applied and Environmental Microbiology 76: 310–317. doi: 10.1128/AEM.01301-09 PMID: 19897755

44. de la Paz Celorio-Mancera M, Heckel DG, Vogel H (2012) Transcriptional analysis of physiological pathways in a generalist herbivore: responses to different host plants and plant structures by the cotton bollworm, *Helicoverpa armigera*. Entomologia Experimentalis et Applicata 144: 123–133.

45. Groot AT, Classen A, Inglis O, Blanco CA, Lopez JR, Vargas AT, et al. (2011) Genetic differentiation across North America in the generalist moth *Heliothis virescens* and the specialist *H*. *subflexa*. Molecular Ecology 20: 2676–2692. doi: 10.1111/j.1365-294X.2011.05129.x PMID: 21615579

46. Terenius O, Papanicolaou A, Garbutt JS, Eleftherianos I, Huvenne H, Albrechtsen M, et al. (2010) RNA interference in Lepidoptera: an overview of successful and unsuccessful studies and implications for experimental design. Journal of Insect Physiology 57: 231–245. doi: 10.1016/j.jinsphys.2010.11.006 PMID: 21078327

47. Aronstein K, Oppert B, Lorenzen MD (2011) RNAi in agriculturaly-important arthropods. In: Grabowski P, editor. RNA Processing. Rijeka, Croatia: InTech. pp. 157–180.

48. Mao Y-B, Tao X-Y, Xue X-Y, Wang L-J, Chen X-Y (2011) Cotton plants expressing CYP6AE14 double-stranded RNA show enhanced resistance to bollworms. Transgenic Research 20: 655–673. doi: 10.1007/s11248-010-9449-7 PMID: 20949317

49. Xue X-Y, Mao Y-B, Tao X-Y, Huang Y-P, Chen X-Y (2012) New approaches to agricultural insect pest control based on RNA interference. In: Jockusch EL, editor. Advances in Insect Physiology. New York: Academic Press. pp. 73–118.

50. Clem RJ, Popham HJR, Shelby KS (2010) Antiviral responses in insects: Apoptosis and humoral responses. In: Asgari S, Johnson KN, editors. Insect Virology. Portland, OR: Caister Academic Press. pp. 383–404.

51. Shelby KS, Popham HJR (2012) RNA-Seq study of microbially induced hemocyte transcripts from larval *Heliothis virescens* (Lepidoptera: Noctuidae). Insects 3: 743–762. doi: 10.1007/s00442-011-2231-6 PMID: 22246471

52. Huang Y, Niu B, Gao Y, Fu L, Li W (2010) CD-HIT Suite: a web server for clustering and comparing biological sequences. Bioinformatics 26: 680–682. doi: 10.1093/bioinformatics/btq003 PMID: 20053844

53. Gotz S, Garcia-Gomez JM, Terol J, Williams TD, Naagaraj SH, Nueda MJ, et al. (2008) High-throughput functional annotation and data mining with the Blast2GO suite. Nucleic Acids Research 36: 3420–3435. doi: 10.1093/nar/gkn176 PMID: 18445632

54. Krueger CM, Degrugillier ME, Narang SK (1993) Size difference among 16S rRNA genes from endosymbiotic bacteria found in testes of *Heliothis virescens*, *H*. *subflexa*, (Lepidoptera: Noctuidae) and backcross sterile male moths. Florida Entomologist 76: 382–390.

55. Jiang H, Vilcinskas A, Kanost MR (2010) Immunity in Lepidopteran insects. Invertebrate Immunity. pp. 163–180.

56. Blander JM, Sander LE (2012) Beyond pattern recognition: five immune checkpoints for scaling the microbial threat. Nature Reviews in Immunology 12: 215–225.

57. Shelby KS, Popham HJR (2008) Cloning and characterization of the secreted hemocytic prophenoloxidases of *Heliothis virescens*. Archives of Insect Biochemistry and Physiology 69: 127–142. doi: 10.1002/arch.20274 PMID: 18839417

58. Shelby KS, Webb BA (1999) Polydnavirus-mediated suppression of insect immunity. Journal of Insect Physiology 45: 507–514. PMID: 12770335

59. Shelby KS, Adeyeye OA, Okot-Kotber BM, Webb BA (2000) Parasitism-linked block of host plasma melanization. Journal of Invertebrate Pathology 75: 218–225. PMID: 10753598

60. Breitenbach JE, Shelby KS, Popham HJ (2011) Baculovirus induced transcripts in hemocytes from the larvae of *Heliothis virescens*. Viruses 3: 2047–2064. doi: 10.3390/v3112047 PMID: 22163334

61. Vodovar N, Saleh M-C (2012) Of insects and viruses: The role of small RNAs in insect defense. In: Jockusch EL, editor. Advances in Insect Physiology. New York: Academic Press. pp. 1–36.

62. Falabella P, Riviello L, Pascale M, Di Lelio I, Tettamanti G, Grimaldi A, et al. (2012) Functional amyloids in insect immune response. Insect Biochemistry and Molecular Biology 42: 203–211. doi: 10.1016/j.ibmb.2011.11.011 PMID: 22207151

63. Bachman PM, Bolognesi R, Moar WJ, Mueller GM, Paradise MS, Ramaseshadri P, et al. (2013) Characterization of the spectrum of insecticidal activity of a double-stranded RNA with targeted activity against Western Corn Rootworm (*Diabrotica virgifera virgifera* LeConte). Transgenic Res 22: 1207–1222. doi: 10.1007/s11248-013-9716-5 PMID: 23748931

64. Wynant N, Duressas TF, Santos D, Van Duppen J, Proost P, Huybrechts R, et al. (2014) Lipophorins can adhere to dsRNA, bacteria and fungi present in the hemolymph of the desert locust: A role as general scavenger for pathogens in the open body cavity. Journal of Insect Physiology 64: 7–13. doi: 10.1016/j.jinsphys.2014.02.010 PMID: 24607637

65. Wynant N, Santos D, Van Wielendaele P, Vanden Broeck J (2014) Scavenger receptor-mediated endocytosis facilitates RNA interference in the desert locust, *Schistocerca gregaria*. Insect Molecular Biology 23: 320–329. doi: 10.1111/imb.12083 PMID: 24528536

66. Ulvila J, Hultmark D, Ramet M (2010) RNA silencing in the antiviral innate immune defense—Role of DEAD-box RNA helicases. Scandinavian Journal of Immunology 71: 146–158. doi: 10.1111/j.1365-3083.2009.02362.x PMID: 20415780

67. Terra WR, Ferreira C (1994) Insect digestive enzymes: properties, compartmentalization and function. Comp Biochem Physiol 109B: 1–62.

68. Claudianos C, Ranson H, Johnson RM, Biswas S, Schuler MA, Berenbaum MR, et al. (2006) A deficit of detoxification enzymes: pesticide sensitivity and environmental response in the honeybee. Insect Molecular Biology 15: 615–636. PMID: 17069637

69. Montella IR, Schama R, Valle D (2012) The classification of esterases: an important gene family involved in insecticide resistance—a review Memorias do Instituto Oswaldo Cruz 107: 437–449. PMID: 22666852

70. Adang MJ, Crickmore N, Jurat-Fuentes JL (2014) Diversity of *Bacillus thuringiensis* crystal toxins and mechanism of action. In: Dhadialla TS, Gill S, editors. Insect Midgut and Insecticidal Proteins. London: Academic Press. pp. 39–87.

71. Gahan LJ, Gould F, Heckel DG (2001) Identification of a gene associated with Bt resistance in *Heliothis virescens*. Science 293: 857–860. PMID: 11486086

72. Gahan LJ, Pauchet Y, Vogel H, Heckel DG (2010) An ABC transporter mutation is correlated with insect resistance to *Bacillus thuringiensis* Cry1Ac toxin. PLOS Genetics 6: e1001248. doi: 10.1371/journal.pgen.1001248 PMID: 21187898

73. Jurat-Fuentes JL, Karumbaiah L, Jakka SRK, Ning C, Liu C, Wu K, et al. (2011) Reduced levels of membrane-bound alkaline phosphatase are common to lepidopteran strains resistant to Cry toxins from *Bacillus thuringiensis*. PLOS One 6: e17606. doi: 10.1371/journal.pone.0017606 PMID: 21390253

74. Luo K, Sangadala S, Masson L, Mazza A, Brousseau R, Adang MJ (1997) The *Heliothis virescens* 170-kDa aminopeptidase functions as 'Receptor A' by mediating specific *Bacillus thuringiensis* Cry1A delta-endotoxin binding and pore formation. Insect Biochem Mol Biol 27: 735–743. PMID: 9443374

75. Banks DJ, Jurat-Fuentes JL, Dean DH, Adang MJ (2001) *Bacillus thuringiensis* Cry1Ac and Cry1Fa delta-endotoxin binding to a novel 110 kDa aminopeptidase in *Heliothis virescens* is not N-acetylgalactosamine mediated. Insect Biochemistry and Molecular Biology 31: 908–919.

76. Perera OP, Willis JD, Adang MJ, Jurat-Fuentes JL (2009) Cloning and characterization of the Cry1Ac-binding alkaline phosphatase (HvALP) from *Heliothis virescens*. Insect Biochemistry and Molecular Biology 39: 294–302. doi: 10.1016/j.ibmb.2009.01.006 PMID: 19552892

77. Jurat-Fuentes JL, Adang MJ (2004) Characterization of a Cry1Ac-receptor alkaline phosphatase in susceptible and resistant *Heliothis virescens* larvae. European Journal of Biochemistry 271: 3127–3135. PMID: 15265032

78. Gill SS, Cowles EA, Francis V (1995) Identification, isolation, and cloning of a *Bacillus thuringiensis* CryIAc toxin-binding protein from the midgut of the Lepidopteran insect *Heliothis virescens*. Journal of Biological Chemistry 270: 27277–27282. PMID: 7592988

79. Oltean DI, Pullikuth AK, Hyun-Ku Lee H-K, Gill SS (1999) Partial purification and characterization of *Bacillus thuringiensis* Cry1A toxin receptor A from *Heliothis virescens* and cloning of the corresponding cDNA Applied and Environmental Microbiology 65: 4760–4766. PMID: 10543783

80. Ning C, Wu K, Liu C, Gao Y, Furat-Fuentes JL, Gao X, et al. (2010) Characterization of a Cry1Ac toxin-binding alkaline phosphatase in the midgut from *Helicoverpa armigera* (Hübner) larvae. Journal of Insect Physiology 56: 666–672. doi: 10.1016/j.jinsphys.2010.02.003 PMID: 20170658

81. Baxter SW, Badenes-Perez FR, Morrison A, Vogel H, Crickmore N, Kain W, et al. (2011) Parallel evolution of *Bacillus thuringiensis* toxin resistance in lepidoptera. Genetics 189: 675–679. doi: 10.1534/genetics.111.130971 PMID: 21840855

82. Park Y, Gonzalez-Martinez RM, Navarro-Cerrillo G, Chakroun M, Kim Y, Ziarsolo P, et al. (2014) ABCC transporters mediate insect resistance to multiple Bt toxins revealed by bulk segregant analysis. BMC Biol 12: 46. doi: 10.1186/1741-7007-12-46 PMID: 24912445

83. Piggot CR, Ellar DJ (2007) Role of receptors in *Bacillus thuringiensis* crystal toxin activity. Microbiol Mol Biol Rev 71: 255–281. PMID: 17554045

84. Denolf P, Hendrick K, Van Damme J, Jansens S, Peferoen M, Degheele D, et al. (1997) Cloning and characterization of *Manduca sexta* and *Plutella xylostella* midgut aminopeptidase N enzymes related to *Bacillus thuringiensis* toxin-binding proteins. Eur J Biochem 248: 748–761. PMID: 9342226

85. Banks DJ, Hua G, Adang MJ (2003) Cloning of a *Heliothis virescens* 110 kDa aminopeptidase N and expression in *Drosophila* S2 cells. Insect Biochem Mol Biol 33: 499–508. PMID: 12706629

86. Crava C, Bel Y, Jakubowska A, Ferré J, Escriche B (2013) Midgut aminopeptidase N isoforms from *Ostrinia nubilalis*: Activity characterization and differential binding to Cry1Ab and Cry1Fa proteins from *Bacillus thuringiensis*. Insect Biochem Molec Biol 43: 924–935.

87. Jurat-Fuentes JL, Adang MJ (2006) Cry toxin mode of action in susceptible and resistant *Heliothis virescens* larvae. Journal of Invertebrate Pathology 92: 166–171. PMID: 16797583

88. Xie R, Zhuang M, Ross LS, Gomez I, Oltean DI, Bravo A, et al. (2005) Single amino acid mutations in the cadherin receptor from *Heliothis virescens* affect its toxin binding ability to Cry1A toxins. Journal of Biological Chemistry 280: 8416–8425. PMID: 15572369

89. Jurat-Fuentes JL, Gahan LJ, Gould FL, Heckel DG, Adang MJ (2004) The HevCaLP protein mediates binding specificity of the Cry1A class of *Bacillus thuringiensis* toxins in *Heliothis virescens*. Biochemistry 43: 14299–14305. PMID: 15518581

90. Fabrick JA, Ponnuraj J, Singh A, Tanwar RK, Unnithan GC, Yelich AJ, et al. (2014) Alternative splicing and highly variable cadherin transcripts associated with field-evolved resistance of pink bollworm to Bt cotton in India. PLoS One 9: e97900. doi: 10.1371/journal.pone.0097900 PMID: 24840729

91. Bel Y, Siqueira HA, Siegfried BD, Ferre J, Escriche B (2009) Variability in the cadherin gene in an *Ostrinia nubilalis* strain selected for Cry1Ab resistance. Insect Biochem Mol Biol 39: 218–223. doi: 10.1016/j.ibmb.2008.11.005 PMID: 19114103

92. Popham HJR, Grasela JJ, Goodman CL, McIntosh AH (2010) Baculovirus infection influences host gene expression in two established insect cell lines. Journal of Insect Physiology 56: 1237–1245. doi: 10.1016/j.jinsphys.2010.03.024 PMID: 20362582

93. Chan QWT, Melathopoulos AP, Pernal SF, Foster LJ (2009) The innate immune and systemic response in honey bees to a bacterial pathogen, *Paenibacillus larvae*. BMC Genomics 10: 387. doi: 10.1186/1471-2164-10-387 PMID: 19695106

94. Krishnamoorthy M, Jurat-Fuentes JL, McNall RJ, Andracht T, Adang MJ (2007) Identification of novel Cry1Ac binding proteins in midgut membranes from *Heliothis virescens* using proteomic analyses. Insect Biochemistry and Molecular Biology 37: 189–201. PMID: 17296494

95. Groot AT, Staudacher H, Barthel A, Inglis O, Schofl G, Santangelo RG, et al. (2013) One quantitative trait locus for intra- and interspecific variation in a sex pheromone. Molecular Ecology 22: 1065–1080. doi: 10.1111/mec.12171 PMID: 23294019

96. Groot AT, Estock ML, Horovitz JL, Hamilton J, Santangelo RG, Schal C, et al. (2009) QTL analysis of sex pheromone blend differences between two closely related moths: insights into divergence in biosynthetic pathways. Insect Biochemistry and Molecular Biology 39: 568–577. doi: 10.1016/j.ibmb.2009.05.002 PMID: 19477278

97. Yang GS, Stott JM, Smailus D, Barber SA, Balasundaram M, Marra MA, et al. (2005) High-throughput sequencing: a failure mode analysis. BMC Genomics 6: 2. PMID: 15631628

98. Breitenbach JE, Popham HJR (2013) Baculovirus replication induces the expression of heat shock proteins *in vivo* and *in vitro*. Archives of Virology 158: 1517–1522. doi: 10.1007/s00705-013-1640-8 PMID: 23443933

99. Marioni JC, Mason CE, Mane SM, Stephens M, Gilad Y (2008) RNA-seq: An assessment of technical reproducibility and comparison with gene expression arrays. Genome Research 18: 1509–1517. doi: 10.1101/gr.079558.108 PMID: 18550803

100. Conesa A, Gotz S (2008) Blast2GO: A comprehensive suite for functional analysis in plant genomics. International Journal of Plant Genomics 2008: 619832. doi: 10.1155/2008/619832 PMID: 18483572

101. Mortazavi A, Williams BA, McCue K, Schaeffer L, Wold B (2008) Mapping and quantifying mammalian transcriptomes by RNA-seq. Nature Methods 5: 621–628. doi: 10.1038/nmeth.1226 PMID: 18516045

102. Itoh M, Kanamori Y, Takao M, Eguchi M (1999) Cloning of soluble alkaline phosphatase cDNA and molecular basis of the polymorphic nature in alkaline phosphatase isozymes of *Bombyx mori* midgut. Insect Biochem Mol Biol 29: 121–129. PMID: 10196735

103. Itoh M, Takeda S, Yamamoto H, Izumi S, Tomino S, Eguchi M (1991) Cloning and sequence analysis of membrane-bound alkaline phosphatase cDNA of the silkworm, *Bombyx mori*. Biochim Biophys Acta 1129: 135–138. PMID: 1756175

104. Atsumi S, Miyamoto K, Yamamoto K, Narukawa J, Kawai S, Sezutsu H, et al. (2012) Single amino acid mutation in an ATP-binding cassette transporter gene causes resistance to Bt toxin Cry1Ab in the silkworm, *Bombyx mori*. Proc Natl Acad Sci U S A 109: E1591–1598. doi: 10.1073/pnas.1120698109 PMID: 22635270

105. Tanaka S, Miyamoto K, Noda H, Jurat-Fuentes JL, Yoshizawa Y, Endo H, et al. (2013) The ATP-binding cassette transporter subfamily C member 2 in *Bombyx mori* larvae is a functional receptor for Cry toxins from *Bacillus thuringiensis*. FEBS J 280: 1782–1794. doi: 10.1111/febs.12200 PMID: 23432933
